# Stereoselective Synthesis and Antiproliferative Activities of Tetrafunctional Diterpene Steviol Derivatives

**DOI:** 10.3390/ijms24021121

**Published:** 2023-01-06

**Authors:** Dorottya Bai, Zsuzsanna Schelz, Dóra Erdős, Anna K. Kis, Viktória Nagy, István Zupkó, György T. Balogh, Zsolt Szakonyi

**Affiliations:** 1 Interdisciplinary Excellence Center, Institute of Pharmaceutical Chemistry, University of Szeged, Eötvös utca 6, H-6720 Szeged, Hungary; 2 Department of Pharmacodynamics and Biopharmacy, University of Szeged, Eötvös utca 6, H-6720 Szeged, Hungary; 3 Department of Chemical and Environmental Process Engineering, Budapest University of Technology and Economics, Muegyetem rkp. 3, H-1111 Budapest, Hungary; 4 Interdisciplinary Centre of Natural Products, University of Szeged, Eötvös utca 6, H-6720 Szeged, Hungary

**Keywords:** diterpene, steviol, stereoselective, aminotriol, antiproliferative activity

## Abstract

A new family of diterpene-type aminotriol derivatives has been synthesised from stevioside in a stereoselective manner. The key intermediate spiro-epoxide was prepared through the methyl ester of the allilyc diol derived from steviol. The oxirane ring was opened with primary and secondary amines, providing a versatile library of aminotriols. The corresponding primary aminotriol was formed by palladium-catalysed hydrogenation, and an *N*,*O*-heterocyclic compound was synthesised in a regioselective reaction. All new compounds were characterised by 1D- and 2D-NMR techniques and HRMS measurements. In our in vitro investigations, we found that the aromatic *N*-substituted derivatives exhibited high inhibition of cell growth on human cancer cell lines (HeLa, SiHa, A2780, MCF-7 and MDA-MB-231). The antiproliferative activities were assayed by the MTT method. Furthermore, the introduction of an additional hydroxy group slightly increased the biological activity. The drug-likeness of the compounds was assessed by in silico and experimental physicochemical characterisations, completed by kinetic aqueous solubility and in vitro intestinal-specific parallel artificial membrane permeability assay (PAMPA-GI) measurements.

## 1. Introduction

Cancer is one of the most feared diseases and a leading cause of death all over the world; therefore, the development of anticancer agents is a major focus for scientists across the world [1,2]. In the past decade, chiral synthons, easily available either from their natural sources or by large-scale preparation, have received significant attention in organic synthesis due to their application as starting materials for asymmetric transformations as well as auxiliaries or chiral catalysts [3,4,5,6]. Because of their coordination capacity and high affinity through their polar functional groups towards biogenic chiral building blocks of cells, aminodiols and aminotriols are of increasing importance, not only as building blocks but also because of their significant biological activities. For example, pactamycin, the most structurally intricate aminocyclopentitol antibiotic, displays potent antiproliferative properties [7], whereas the immunosuppressant antibiotic myriocin [8,9] and penaresdin A and B were identified as potent actomyosin ATP-ase activators [10]. A large majority of these compounds are derived from commercially available monoterpenes, such as (–)-isopulegol, α- or β-pinene [6,11], while only a handful of chiral sources of diterpenes are commercially available in a large scale [12,13,14]. Among them, stevioside (**I**) is a frequently used source of bioactive diterpenoid derivatives since it can be isolated at an industrial scale from *Stevia rebaudiana* and it can be easily transformed to its aglycons, steviol and isosteviol [15,16,17,18].

**Figure 1:**
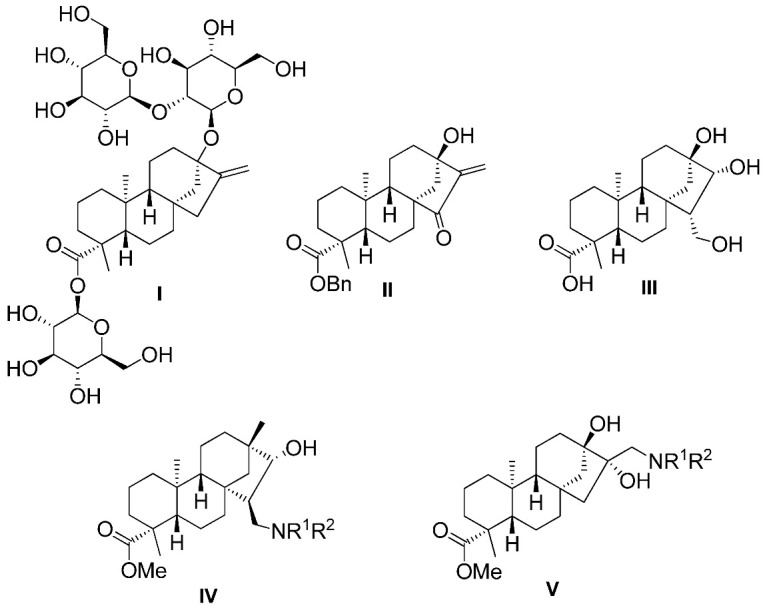
Bioactive compounds with steviol- and isosteviol-type skeleton derived from stevioside.

Stereoselective transformations of both steviol and isosteviol, affording reactive intermediates (**II**, **III**) and aminoalcohols (**IV**), as well as aminodiols (**V**) with antiproliferative activity, were studied previously (Figure 1) [15,17,19,20]. The outstanding pharmacological effects of the *N*-substituted derivatives raised our curiosity about this class of compounds. Therefore, our aim was to expand the aforementioned topic by introducing a third hydroxy function to study its effect on the antiproliferative activity of a library of novel aminotriols obtained from stevioside (**I**).

In the present study, we report the synthesis, physicochemical characterization and classification of a novel series of chiral tetrafunctional compounds, such as 2-aminomethyl-1,2,3-triol derivatives, starting from steviol. In addition, we planned to investigate their antiproliferative activities on human cancer cell lines and interpret the influence of an assortment of amine functions on their bioactivity, further developing our previous studies in this field.

## 2. Results and Discussion

### 2.1. Synthesis of Key Intermediate Spiro-Epoxide ***4b***

**Scheme 1:**
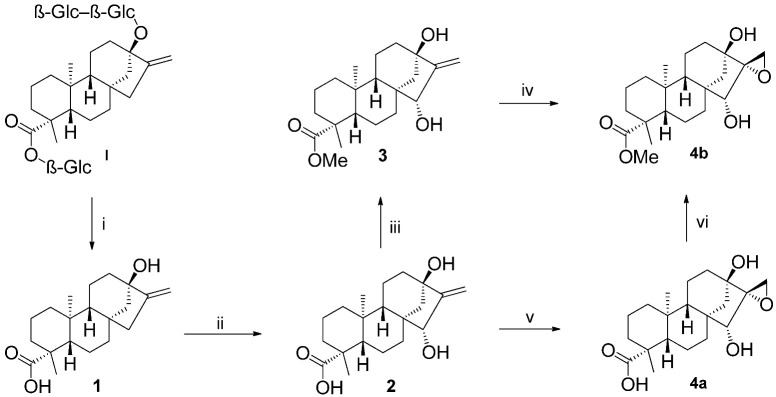
Chemo- and stereoselective synthesis of key intermediate spiro-epoxide **4b** from stevioside. (i) (a) NaIO_4_, H_2_O, 16 h, 25 °C; (b) KOH, H_2_O, 1 h, 100 °C, 57%. (ii) SeO_2_, *t*-BuOOH, dry THF, 1 day, 25 °C, 68%. (iii) CH_2_N_2_, Et_2_O, 2 min, 25 °C, 83%. (iv) *t*-BuOOH, VO(acac)_2_, dry toluene, 30 min, 0 °C, 1 h, 25 °C, 88%. (v) *m*-CPBA, dichloromethane, 24 h, 25 °C, 66%. (vi) CH_2_N_2_, Et_2_O, 2 min, 25 °C, 81%.

Starting from commercially available natural glycoside stevioside (**I**, obtained from Molar Chemicals Ltd, Halásztelek, Hungary, steviol (**1**) was synthesised in two steps in accordance with the methods described in the literature [21,22]. Allylic hydroxylation of compound **1** was accomplished by the application of selenium(IV) dioxide and tert-butyl hydroperoxide in dry THF [23]. The reaction was found to be stereoselective for the 15-α-OH isomer (**2**), as described in the literature [24,25]. The first attempt at the esterification of **2** was carried out with iodomethane, affording a low yield. In contrast, diazomethane was found to be a more efficient reagent, resulting in methyl ester **3** in a matter of minutes without a cyclopropanation side reaction (Scheme 1) [21]. Switching of the last two steps was also considered but it was dismissed because of the observation of low yields.

The epoxidation of methyl ester **3** with t-BuOOH as an oxidising agent was catalysed by vanadyl acetylacetonate (VO(acac)_2_). The change in oxidation state of vanadium (V(IV)→V(V)) throughout the reaction could be monitored by the colour of the mixture, which enabled tracking the completion of the process [26]. To determine the stereochemistry of spiro-epoxide **4b** provided by the stereospecific reaction, the compound was synthesised in an alternative pathway as well. As described in the literature, treatment of **2** with meta-chloroperoxybenzoic acid in dichloromethane gave derivative **4a** with known stereochemistry (Scheme 1) [25]. The ester synthesis was carried out with diazomethane, and the resulting product, according to the NMR spectra, proved to possess the same structure as that prepared using t-BuOOH (**4b**). Considering both the reaction time and the observed yields, we decided to continue working with the vanadium-catalysed method.

### 2.2. Synthesis of Aminotriol Derivatives ***5***–***17***

**Scheme 2:**
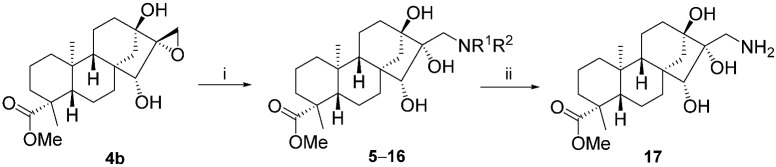
Stereoselective synthesis of aminotriols **5**–**17**. (i) HNR^1^R^2^, LiClO_4_, CH_3_CN, 2 days, 25 °C, 45–96% (ii) Pd/C, H_2_ (1 atm), MeOH, 1 day, 25 °C, 41%.

The nucleophilic addition of amines to epoxyalcohols is an elegant way for the synthesis of a versatile collection of aminoalcohols, and it has been previously reported in a number of studies by our research group [27,28,29]. Following the described method, the oxirane ring of epoxide **4b** was opened with a series of primary and secondary amines in the presence of LiClO_4_ as a Lewis acid catalyst (Scheme 2). The coordination of the lithium ion to the epoxide oxygen is presumed to be increasing the electrophilic character of the ring against the nucleophilic attack of the amine. Therefore, activating the compound toward ring opening not only improves the reaction time and yield, but it also enhances the stereoselectivity [30,31]. The preparation of novel aminotriols was accomplished with moderate to excellent yields, as shown in Table 1. The secondary amine N-benzylmethylamine was found to be less efficient for the synthesis, and the resulting derivative (**6**) showed lower bioactivity in our in vitro pharmacological study as well. Furthermore, compound **5** was subjected to debenzylation by hydrogenolysis over Pd/C to obtain primary aminotriol **17** in a moderate yield (Scheme 2).

**Scheme 3:**
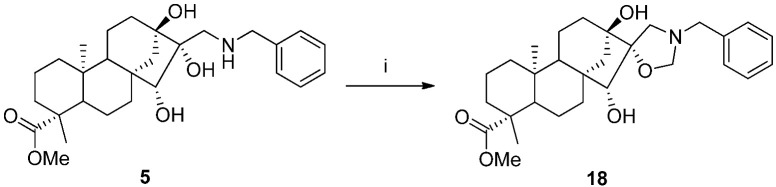
Ring closure of aminotriol **5** with formaldehyde. (i) 35% HCHO, Et_2_O, 1 h, 25 °C, 88%.

In earlier studies, remarkable cytotoxic activity expressed by steviol-based oxazolidine derivatives on human cancer cell lines was observed [32,33]. Intrigued by our previous results, we decided to synthesise the heterocyclic counterpart of compound **5** and determine the regioselectivity of ring closure. Treatment of aminotriol **5** with aqueous formaldehyde at room temperature gave spiro-oxazolidine **18** in an exclusive manner in a highly regioselective reaction (Scheme 3).

**Figure 2:**
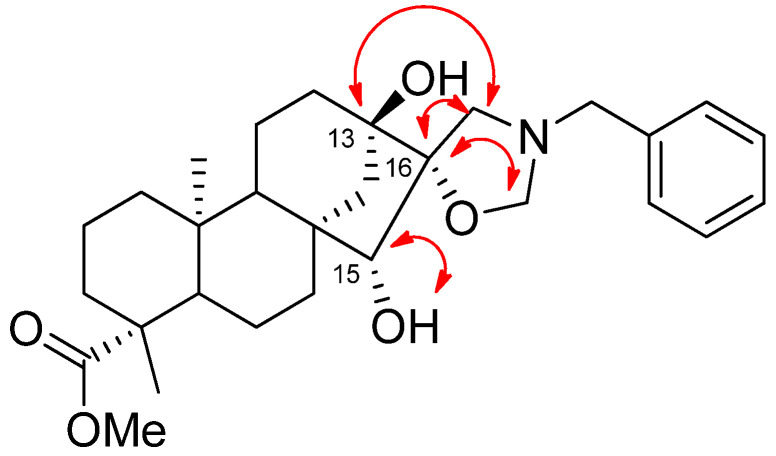
Determination of the structure of derivative **18** by HMBC.

The ^1^H NMR spectrum in DMSO-d_6_ clearly shows that 15-α-OH exists as a doublet and does not participate in ring closure. Determination of the structure was performed by studying the 2D-NMR data of **18**. Cross-coupling could be observed in the HMBC spectrum between the hydrogens carried by the carbon atom of the heterocycle bonded to both the nitrogen and the oxygen, and to C_16_, but not with C_13_ and C_15_ (Figure 2).

### 2.3. Drug-Likeness Properties of Steviol-Based Aminotriols Using in Silico and Experimental Physicochemical Parameters

The physicochemical classification and drug-likeness evaluation of the steviol derivatives were investigated at two levels using in silico and in vitro tests. The predicted values of Lipinski’s rule of five (Ro5) [34] and two additional parameters (pK_a_, topological polar surface area: TPSA) for estimation of drug absorption are presented in Table 2. In the study of steviol derivatives, the Ro5 violation occurred at two levels. In the case of benzyl derivatives, the molecular weight (MW) of **10**, **11**, while the lipophilicity (logP) of **15**, **16** slightly exceeded the drugability limits defined by Ro5. A higher level of Ro5 violation could be identified in the case of the three naphthyl derivatives (**13**–**15**), as their MW and calculated logP values exceeded the specified rule of thumb threshold. The acid-base character of steviol derivatives was also evaluated based on the predicted pK_a_ values. The steviol (**2**) starting compound has an acidic character (pK_a_ = 4.6) due to its free carboxylic acid group, while the steviol methyl ester derivative **3** and the spiro-epoxide derivative (**4b**) that can be derived from it have a neutral character. The other steviol-derived aminotriols (**5**–**18**) have a basic character of almost the same strength (pK_a_ = 9.2–9.6) with some characteristic differences. Thus, the basicity of the debenzylated primary aminotriol (**17**: pK_a_ = 10.2) increased, and the basicity of the dialkylated aminotriol derivative (**6**: pK_a_ = 8.6) decreased compared to compound **5** due to the steric effect of the incorporated methyl group. A greater decrease in the basic character can be identified for the spiro-oxazolidine derivative (**18**: pK_a_ = 5.2). It is also important to mention that the TPSA value of all tested compounds is less than 140 Å^2^, which corresponds to the oral bioavailability rule defined by Veber et al. [35].

Kinetic aqueous solubility and in vitro intestinal effective permeability (using PAMPA-GI system [36]) of steviol derivatives were also determined (Table 3). Regarding the kinetic solubility values, only compounds **9**, **16** and **12**–**14** have moderate and poor aqueous solubility, respectively. This result shows a good correlation with the high log*P* value of the naphthyl derivatives. The intestinal-specific permeability values (P_e_) show that only four steviol derivatives (**2**, **5**, **7** and **8**) have a P_e_ value in the moderate category. The other derivatives (**15** and **16**) had poor permeability or could not be detected with the developed HPLC-MS method on the acceptor side of the PAMPA system. In the latter case, it is worth dividing the compounds into two types on the basis of their membrane retention value (MR%). In the case of **12**–**14**, the inhibited membrane permeability can be explained by the increased membrane partition (MR > 90%) or hydrophobic character (see log*P* values), while the hydrophilic character of **17**, **18** (MR < 5%). Regarding the experimental data, it is also worth mentioning that in the case of the **5**, **6** and **18** derivatives, the decrease in the basicity of the aminotriols was accompanied by a decrease in the effective permeability. In the case of compounds **5** and **6**, the decrease in basicity was also associated with a decrease in kinetic solubility.

### 2.4. In Vitro Antiproliferative Studies of Steviol-Based Aminotriols and Structure-Activity Relationship

**Figure 3:**
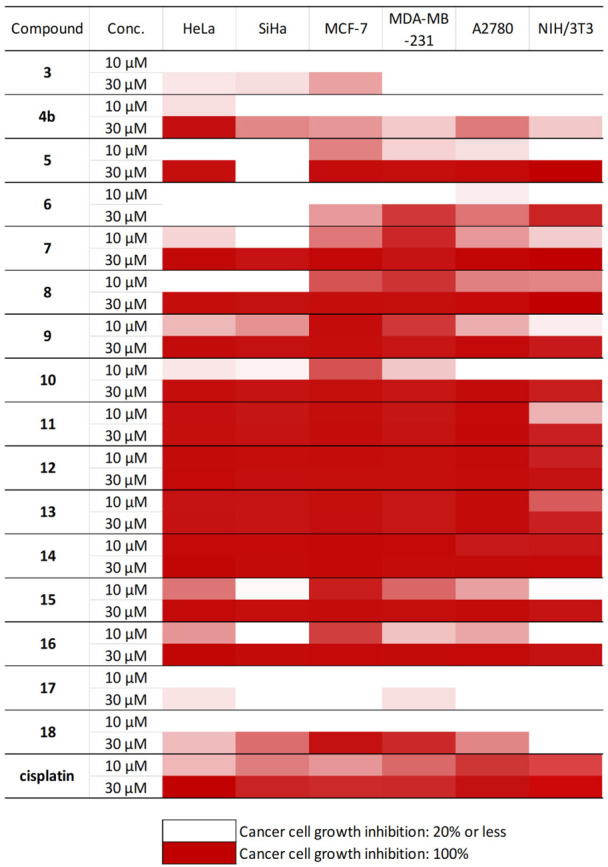
Antiproliferative properties of the prepared steviol analogues against cancer cells and NIH/3T3 fibroblasts.

The in vitro antiproliferative activities of the synthesised aminotriols **5**–**18** against a panel of different human cancer cell lines of gynecological origin, including cervical (SiHA and HeLa), breast (MCF7 and MDA-MB-231), ovary (A2780) cancers and NIH/3T3 healthy fibroblasts, were assayed by the MTT method. Cisplatin, a clinically applied anticancer agent was used as a reference compound, and the results are summarised in Figure 3 and Table S1 in the Supplementary Materials.

Based on the results of in vitro viability assays, it can be concluded that SiHa cells from cervix cancer seem less sensitive. However, the utilised cancer panel detected no substantial cell line selective action. In addition, some compounds (**11**–**14**) exhibited more pronounced antiproliferative activity than the reference agent cisplatin, and non-cancerous fibroblast cells were damaged in the same concentration range, indicating a general cytotoxic property of the tested substances.

The in vitro pharmacological assay showed elevated but non-selective inhibition of cell growth for compounds **11**–**14** in 10 µM concentration on all tumour cell lines studied (Figure 3). This result shows a good agreement with the lipophilicity values presented in Table 2 and with the risk of promiscuity introduced by Leeson and Springthorpe [37] and Waring [38], since derivatives **11**–**14** have increased lipophilicity. Furthermore, in the case of **12**–**14**, the risk is also supported by the increased membrane retention (Table 3) identified by the PAMPA-GI study. Selectivity for MCF-7 cells increased slightly in the case of the heterocyclic derivative **18** in 30 µM, with moderate activity on the SiHa, MDA-MB-231 and A2780 lines. Compounds **10** and **16** exhibited higher selectivity on the MCF-7 cell line at lower concentrations, with the latter also showing a moderate inhibition of HeLa cell growth. On the MDA-MB-231 cell line, derivatives **7** and **8** displayed noteworthy selectivity, followed by slightly weaker action on MCF-7 and A2780. A strong and selective antiproliferative effect was found regarding compounds **9** and **15** on MCF-7, with decent activity on the MDA-MB-231 line. Additionally, **9** exerted modest inhibition on SiHa cell line, while **15** exerted modest inhibition on HeLa and A2780. Derivatives **4b**, **5** and **6** executed significant activity in higher concentrations only, with **4b** expressing potent inhibition on the HeLa line and **6** having a slight selectivity for MDA-MB-231 cells. In the cases of **2**, **3** and **17,** any notable effect was not detectable.

Analysis of the results led us to believe that the presence of the N-benzyl substituent at the amino function is required for the cytotoxic effect in the case of aminotriols too, similar to that noted previously on steviol-derived aminodiols [18]. Based on recent data, the naphthyl rings, while increasing the activity, lower the selectivity for a specific cell line. Substituents, such as methoxy and fluorine in para-position of the benzene ring, favour the MCF-7 cells regarding the inhibition of cell growth. The α-alkyl groups seem to be beneficial for the selectivity; furthermore, the number of carbon atoms has influence on the affinity towards specific types of cells. However, when paired with the p-fluorine function, these properties are lost. The spiro-heterocycle lowers the strength of action, but the selectiveness observed holds potential for the compound for future investigations. Considering the antiproliferative activity profile of the studied steviol derivatives against NIH/3T3 healthy cell lines, the most selective aminotriols (**9**, **10**, **15** and **16**) performed best on the MCF-7 breast cancer cell line, which indicates group selectivity. In addition, a smaller but also selective antiproliferative effect was found on the other breast cancer cell line (MDA-MB-231) in the case of derivatives **9**, **15** and **18**.

Summarising the antiproliferative activity and the in silico (Table 2) and early ADME physicochemical properties (Table 3), compounds **9**, **10** and **16** can be considered as primary in vivo preclinical candidates, while derivatives **15** and **18** can be secondary candidates for further studies.

## 3. Materials and Methods

### 3.1. General Methods

Commercially available reagents were used as obtained from suppliers (Et_2_O, dichloromethane, MeOH, EtOAc, n-hexane: Novochem Co., Ltd., 1089 Budapest, Hungary, Orczy út 6.; toluene, MeCN: Merck Ltd., Budapest, Hungary and DMSO-d_6_, CDCl_3_: VWR International Ltd., Debrecen, Hungary), while solvents were dried according to standard procedures. Chromatographic separations and monitoring of reactions were carried out on Merck Kieselgel 60 (Merck Ltd., Budapest, Hungary). HRMS flow injection analysis was performed with a Thermo Scientific Q Exactive Plus hybrid quadrupole-Orbitrap (Thermo Fisher Scientific, Waltham, MA, USA) mass spectrometer coupled to a Waters Acquity I-Class UPLC™ (Waters, Manchester, UK). Melting points were determined on a Kofler apparatus (Nagema, Dresden, Germany). Optical rotations were measured in MeOH at 20 °C with a Perkin-Elmer 341 polarimeter (PerkinElmer Inc., Shelton, CT, USA). ^1^H-, ^13^C *J*-MOD and ^19^F-NMR spectra were recorded on a Bruker Avance DRX 500 spectrometer (Bruker Biospin, Karlsruhe, Baden-Württemberg, Germany) [500 MHz (^1^H), 125 MHz (^13^C *J*-MOD), and 470 MHz (^19^F) δ = 0 (TMS)]. Chemical shifts are expressed in ppm (δ) relative to TMS as an internal reference. *J* values are given in Hz. All ^1^H-, ^13^C *J*-MOD-, ^19^F-NMR, COSY, NOESY, 2D-HMBC and 2D-HMQC spectra are available in the Supporting Information file. Physicochemical parameters (Table 2) were calculated by Percepta, Version: v2020 Build 3382 (ACD/Labs, Toronto, ON, Canada). Available online: https://www.acdlabs.com/products/percepta/ (accessed on 18 June 2020).

### 3.2. Starting Materials

Natural glycoside, stevioside **I** was obtained from Molar Chemicals Ltd., Halásztelek, Hungary. Preparation of steviol **1** was accomplished according to the literature method from commercially available stevioside, and its spectroscopic data were the same as those reported therein [39].

The synthesis of compound **3** was carried out according to literature methods. Its physical and chemical properties and spectroscopic data were similar to those described in the literature [40,41]. ^1^H, ^13^C *J*-MOD, ^19^F, COSY, NOESY, HSQC and HMBC NMR spectra of the new compounds are available in the Supplementary Materials.

#### 3.2.1. (4*R*,6a*R*,7*R*,9*S*,11b*S*)-Methyl 7,9-Dihydroxy-4,11b-dimethyl-8-methylenetetradecahydro-6a,9-methanocyclohepta[a]naphthalene-4-carboxylate (**3**)

2.00 g (5.98 mmol) **2** was dissolved in Et_2_O (200 mL) and CH_2_N_2_ in Et_2_O was added dropwise while the solution was stirred at room temperature. The solvent was evaporated as soon as the mixture turned a permanent yellow colour. The resulting crude product was purified by column chromatography on silica gel with *n*-hexane/EtOAc = 1:9. Yield: 1.64 g (83%); white crystals; m.p.: 253–255 °C; [*α*]_D_^20^ = −87 (*c* 0.0433 MeOH); ^1^H-NMR (500 MHz, DMSO-d_6_) δ (ppm): 0.76 (s, 3H), 0.77–0.82 (m, 1H), 0.85 (d, 1H, *J* = 8.2 Hz), 0.96–1.02 (m, 1H), 1.04 (d, 1H, *J* = 12.3 Hz), 1.12 (s, 3H), 1.22–1.29 (m, 2H), 1.31–1.38 (m, 2H), 1.45 (d, 1H, *J* = 10.4 Hz), 1.56–1.64 (m, 4H), 1.70–1.76 (m, 2H), 1.77–1.82 (m, 2H), 2.05 (d, 1H, *J* = 13.2 Hz), 3.57 (s, 3H), 3.59 (d, 1H, *J* = 5.4 Hz), 4.40 (d, 1H, *J* = 5.9 Hz), 4.63 (s, 1H), 5.05 (d, 2H, *J* = 9.1 Hz); ^13^C-NMR (125 MHz, DMSO-d_6_) δ (ppm): 15.7 (CH_3_), 19.2 (CH_2_), 20.1 (CH_2_), 21.3 (CH_2_), 28.7 (CH_3_), 35.4 (CH_2_), 37.9 (CH_2_), 39.4 (C_q_), 40.6 (CH_2_), 43.3 (CH_2_), 43.7 (C_q_), 45.7 (C_q_), 51.4 (CH_3_), 52.5 (CH), 56.7 (CH), 78.1 (C_q_), 80.6 (CH), 107.1 (CH_2_), 161.6 (C_q_), 177.5 (C=O). HRMS (ESI+): *m/z* calcd. for C_21_H_31_O_3_^+^ [(M-H_2_O) + H]^+^ 331.2268; found 331.2267 (mass error Δ_m_ = −0.22 ppm).

#### 3.2.2. (2’*R*,4*R*,6a*R*,7*R*,9*S*,11b*S*)-Methyl 7,9-Dihydroxy-4,11b-dimethyldodecahydro-1H-spiro[6a,9-methanocyclohepta[a]naphthalene-8,2’-oxirane]-4-carboxylate (**4b**)

An emerald green solution of **3** (1 g, 2.87 mmol) and VO(acac)_2_ (10 mg) in dry toluene (100 mL) was stirred at 0 °C for 30 min. A solution of *t*-BuOOH (2 mL) in dry toluene (20 mL) was added dropwise to the mixture after it had dried on Na_2_SO_4_ and been filtered. The colour of the solution changed to a deep red during the addition, and after stirring for 1 h, it faded to orange. A saturated NaHCO_3_ solution (20 mL) was added to the mixture, then it was extracted with toluene (3 × 30 mL), and the organic layer was washed with brine before it was dried (Na_2_SO_4_), filtered, and concentrated. The resulting crude product was purified by column chromatography on silica gel with n-hexane/EtOAc 1:9. Yield: 0.92 g (88%); white crystals; m.p.: 182–185 °C; [*α*]_D_^20^ = −67(*c* 0.0467 MeOH); ^1^H-NMR (500 MHz, DMSO-d_6_) δ (ppm): 0.77 (s, 3H), 0.80–0.84 (m, 1H), 0.91 (d, 1H, *J* = 7.3 Hz), 0.97–1.04 (m, 1H), 1.06 (d, 1H, *J* = 12.3 Hz), 1.12 (s, 3H), 1.26–1.32 (m, 1H), 1.34–1.40 (m, 2H), 1.50–1.63 (m, 4H), 1.67–1.76 (m, 3H), 0.78–0.84 (m, 3H), 2.05 (d, 1H, *J* = 13.2 Hz), 2.72 (d, 1H, *J* = 5.5 Hz), 2.82 (d, 1H, *J* = 5.5 Hz), 3.30 (d, 1H, *J* = 4.1 Hz), 3.58 (s, 3H), 3.61 (d, 1H, *J* = 4.1 Hz), 4.02 (s, 1H); ^13^C-NMR (125 MHz, DMSO-d_6_) δ (ppm): 15.9 (CH_3_), 19.2 (CH_2_), 19.4 (CH_2_), 21.3 (CH_2_), 28.7 (CH_3_), 35.2 (CH_2_), 36.7 (CH_2_), 37.9 (CH_2_), 39.4 (C_q_), 40.5 (CH_2_), 42.7 (CH_2_), 43.7 (C_q_), 45.7 (C_q_), 47.6 (CH_2_), 51.5 (CH_3_), 52.1 (CH), 56.5 (CH), 66.8 (C_q_), 73.6 (C_q_), 79.4 (CH), 177.5 (C=O). HRMS (ESI+): *m/z* calcd. for C_21_H_33_O_5_^+^ [M + H]^+^ 365.2323; found 365.2322 (mass error Δ_m_ = −0.14 ppm).

### 3.3. General Procedure for Preparation of Aminotriols with Primary and Secondary Amines

LiClO_4_ (58.4 mg, 0.54 mmol) and one (**5**, **9**–**10**, **14**–**16**) or two equivalents (**6**–**8**, **11**–**13**) of the appropriate amine were added to a solution of 4**b** (100 mg, 0.27 mmol) in dry MeCN (8 mL). The mixture was stirred for two days at room temperature (**5**–**11** and **14**–**16**) or at reflux (**12**–**13**). After completion of the reactions, water (10 mL) was added, followed by extraction with CH_2_Cl_2_ (3 × 15 mL). The organic phases were dried (Na_2_SO_4_) and evaporated to dryness. The crude product was purified by column chromatography on silica gel with CHCl_3_/MeOH = 19:1.

#### 3.3.1. (4*R*,6a*R*,7*R*,8*R*,9*S*,11b*S*)-Methyl 8-((Benzylamino)methyl)-7,8,9-trihydroxy-4,11b-dimethyltetradecahydro-6a,9-methanocyclohepta[a]naphthalene-4-carboxylate (**5**)

The reaction was accomplished with benzylamine, as described in the general procedure. Yield: 118 mg (93%); white crystals; m.p.: 119–122 °C; [*α*]_D_^20^ = −33 (*c* 0.05 MeOH); ^1^H-NMR (500 MHz, DMSO-d_6_) δ (ppm): 0.72 (s, 3H), 0.76–0.81 (m, 1H), 0.88 (d, 1H, *J* = 8.3 Hz), 0.97–1.02 (m, 1H), 1.04 (d, 1H, *J* = 11.6 Hz), 1.11 (s, 3H), 1.24–1.29 (m, 1H), 1.33–1.54 (m, 7H), 1.56 (s, 2H), 1.58–1.64 (m, 1H), 1.71–1.81 (m, 3H), 2.03 (d, 1H, *J* = 12.7 Hz), 2.86 (s, 1H), 3.31 (s, 2H), 3.36 (s, 1H), 3.56 (s, 3H), 4.07 (q, 2H, *J* = 13.3 Hz, 13.8 Hz), 5.05 (s, 1H), 7.37 (t, 1H, J = 7.1 Hz), 7.40 (t, 2H, *J* = 7.3 Hz), 7.49 (d, 2H, *J* = 7.3 Hz); ^13^C-NMR (125 MHz, DMSO-d_6_) δ (ppm): 15.4 (CH_3_), 19.0 (CH_2_), 19.1 (CH_2_), 21.4 (CH_2_), 28.6 (CH_3_), 33.2 (CH_2_), 35.5 (CH_2_), 37.9 (CH_2_), 39.3 (C_q_), 40.3 (CH_2_), 42.2 (CH_2_), 43.6 (C_q_), 45.9 (C_q_), 50.4 (CH_2_), 52.0 (CH_2_), 51.5 (CH_3_), 53.3 (CH), 56.3 (CH), 75.9 (C_q_), 78.7 (C_q_), 80.9 (CH), 129.0 (3xCH), 130.1 (2xCH), 134.7 (C_q_), 177.5 (C=O). HRMS (ESI+): *m/z* calcd. for C_28_H_42_NO_5_^+^ [M + H]^+^ 472.3056; found 472.3057 (mass error Δ_m_ = 0.11 ppm).

#### 3.3.2. (4*R*,6a*R*,7*R*,8*R*,9*S*,11b*S*)-Methyl 8-((Benzyl(methyl)amino)methyl)-7,8,9-trihydroxy-4,11b-dimethyltetradecahydro-6a,9-methanocyclohepta[a]naphthalene-4-carboxylate (**6**)

The reaction was accomplished with *N*-benzylmethylamine, as described in the general procedure. Yield: 59 mg (45%); white crystals; m.p.: 162–166 °C; [*α*]_D_^20^ = −57 (*c* 0.05 MeOH); ^1^H-NMR (500 MHz, DMSO-d_6_) δ (ppm): 0.72 (s, 3H), 0.74–0.80 (m, 1H), 0.85 (d, 1H, *J* = 8.4 Hz), 0.96–1.00 (m, 1H), 1.02 (d, 1H, *J* = 11.5 Hz), 1.11 (s, 3H), 1.23–1.29 (m, 2H), 1.33–1.38 (m, 1H), 1.39–1.44 (m, 2H), 1.44–1.57 (m, 5H), 1.71–1.81 (m, 3H), 2.03 (d, 1H, *J* = 12.8 Hz), 2.22 (s, 3H), 2.45–2.49 (m, 1H, overlapped with DMSO-d_6_), 2.62 (d, 1H, *J* = 13.2 Hz), 3.22 (d, 1H, *J* = 7.4 Hz), 3.30 (s, 1H), 3.56 (s, 3H), 3.60 (q, 2H, *J* = 8.4 Hz, 12.9 Hz, overlapped with hydrogens of the esteric methyl group), 3.90 (s, 1H), 4.11–4.18 (m, 1H), 4.81 (s, 1H), 7.22–7.27 (m, 1H), 7.29–7.32 (m, 2H), 7.32 (s, 2H); ^13^C-NMR (125 MHz, DMSO-d_6_) δ (ppm): 15.5 (CH_3_), 19.0 (CH_2_), 19.1 (CH_2_), 21.5 (CH_2_), 28.6 (CH_3_), 32.8 (CH_2_), 35.8 (CH_2_), 37.9 (CH_2_), 39.3 (C_q_), 40.3 (CH_2_), 42.7 (CH_2_), 43.60 (C_q_), 43.64 (CH_3_), 45.8 (C_q_), 51.5 (CH_3_), 53.2 (CH), 56.5 (CH), 59.5 (CH_2_), 63.2 (CH_2_), 76.6 (C_q_), 78.5 (C_q_), 83.1 (CH), 127.4 (CH), 128.6 (2xCH), 129.5 (2xCH), 139.5 (C_q_), 177.5 (C=O). HRMS (ESI+): *m/z* calcd. for C_29_H_44_NO_5_^+^ [M + H]^+^ 486.3214; found 486.3216 (mass error Δ_m_ = 0.41 ppm).

#### 3.3.3. (4*R*,6a*R*,7*R*,8*R*,9*S*,11b*S*)-Methyl 7,8,9-Trihydroxy-4,11b-dimethyl-8-((((*R*)-1-phenylethyl)amino)methyl)tetradecahydro-6a,9-methanocyclohepta[a]naphthalene-4-carboxylate (**7**)

The reaction was accomplished with *R*-(+)-α-methylbenzylamine, as described in the general procedure. Yield: 77 mg (59%); white crystals; m.p.: 84–87 °C; [*α*]_D_^20^ = −38 (*c* 0.0667 MeOH); ^1^H-NMR (500 MHz, DMSO-d_6_) δ (ppm): 0.70 (s, 3H), 0.73–0.78 (m, 1H), 0.81 (d, 1H, *J* = 8.1 Hz), 0.95–0.99 (m, 1H), 1.01 (d, 1H, *J* = 12.2 Hz), 1.10 (s, 3H), 1.14–1.21 (m, 1H), 1.24 (s, 1H), 1.28 (d, 3H, *J* = 5.5 Hz), 1.32–1.37 (m, 2H), 1.38–1.43 (m, 1H), 1.47 (s, 1H), 1.50 (s, 3H), 1.53 (s, 1H), 1.55–1.69 (m, 1H), 1.70–1.80 (m, 3H), 2.02 (d, 1H, *J* = 13.2 Hz), 2.37 (d, 1H, *J* = 11.6 Hz), 2.57 (d, 1H, *J* = 11.3 Hz), 3.18 (s, 1H), 3.32 (s, 1H), 3.55 (s, 3H), 3.75 (s, 1H), 4.46 (s, 1H), 4.61 (s, 1H), 7.21 (t, 1H, *J* = 6.9 Hz), 7.31 (t, 2H, *J* = 7.3 Hz), 7.36 (d, 2H, *J* = 7.3 Hz); ^13^C-NMR (125 MHz, DMSO-d_6_) δ (ppm): 15.4 (CH_3_), 19.1 (CH_2_), 19.2 (CH_2_), 21.4 (CH_2_), 24.6 (CH_3_), 28.6 (CH_3_), 33.4 (CH_2_), 35.7 (CH_2_), 37.9 (CH_2_), 39.3 (C_q_), 40.3 (CH_2_), 42.5 (CH_2_), 43.6 (C_q_), 45.8 (C_q_), 50.5 (CH_2_), 51.5 (CH_3_), 53.3 (CH), 56.4 (CH), 58.6 (CH), 77.0 (C_q_), 78.6 (C_q_), 81.8 (CH), 127.1 (3xCH), 128.7 (2xCH), 145.8 (C_q_), 177.5 (C=O). HRMS (ESI+): *m/z* calcd. for C_29_H_44_NO_5_^+^ [M + H]^+^ 486.3214; found 486.3216 (mass error Δ_m_ = 0.41 ppm).

#### 3.3.4. (4*R*,6a*R*,7*R*,8*R*,9*S*,11b*S*)-Methyl 7,8,9-Trihydroxy-4,11b-dimethyl-8-((((*S*)-1-phenylethyl)amino)methyl)tetradecahydro-6a,9-methanocyclohepta[a]naphthalene-4-carboxylate (**8**)

The reaction was accomplished with *S*-(–)-α-methylbenzylamine, as described in the general procedure. Yield: 79 mg (60%); white crystals; m.p.: 157–161 °C; [*α*]_D_^20^ = −27 (*c* 0.05 MeOH); ^1^H-NMR (500 MHz, DMSO-d_6_) δ (ppm): 0.71 (s, 3H), 0.74–0.78 (m, 1H), 0.84 (d, 1H, *J* = 8.3 Hz), 0.95–1.00 (m, 1H), 1.02 (d, 1H, *J* = 12.0 Hz), 1.10 (s, 3H), 1.24–1.26 (m, 1H), 1.26–1.32 (m, 4H), 1.34–1.40 (m, 3H), 1.48 (s, 1H), 1.50 (s, 3H), 1.52–1.58 (m, 2H), 1.73 (d, 2H, *J* = 11.7 Hz), 1.78 (d, 1H, *J* = 13.3 Hz), 2.03 (d, 1H, *J* = 13.3 Hz), 2.50–2.52 (m, 2H, overlapped with DMSO-d_6_), 3.20 (s, 1H, overlapped with H_2_O), 3.28 (s, 1H, overlapped with H_2_O), 3.55 (s, 3H), 3.78 (s, 1H), 4.45 (s, 2H), 7.20–7.26 (m, 1H), 7.29–7.39 (m, 4H); ^13^C-NMR (125 MHz, DMSO-d_6_) δ (ppm): 15.4 (CH_3_), 19.1 (CH_2_), 19.2 (CH_2_), 21.4 (CH_2_), 24.0 (CH_3_), 28.6 (CH_3_), 33.3 (CH_2_), 35.7 (CH_2_), 37.9 (CH_2_), 39.3 (C_q_), 40.3 (CH_2_), 42.5 (CH_2_), 43.6 (C_q_), 45.8 (C_q_), 50.3 (CH_2_), 51.5 (CH_3_), 53.3 (CH), 56.4 (CH), 58.5 (CH), 76.8 (C_q_), 78.6 (C_q_), 81.9 (CH), 127.2 (3xCH), 128.8 (2xCH), 144.8 (C_q_), 177.5 (C=O). HRMS (ESI+): *m/z* calcd. for C_29_H_44_NO_5_^+^ [M + H]^+^ 486.3214; found 486.3214 (mass error Δ_m_ = 0.0 ppm).

#### 3.3.5. (4*R*,6a*R*,7*R*,8*R*,9*S*,11b*S*)-Methyl 8-(((4-Fluorobenzyl)amino)methyl)-7,8,9-trihydroxy-4,11b-dimethyltetradecahydro-6a,9-methanocyclohepta[a]naphthalene-4-carboxylate (**9**)

The reaction was accomplished with 4-fluorobenzylamine, as described in the general procedure. Yield: 126 mg (95%); white crystals; m.p.: 195–197 °C; [*α*]_D_^20^ = −47 (*c* 0.08 MeOH); ^1^H-NMR (500 MHz, DMSO-d_6_) δ (ppm): 0.73 (s, 3H), 0.75–0.81 (m, 1H), 0.87 (d, 1H, *J* = 7.7 Hz), 0.97–1.02 (m, 1H), 1.03 (d, 1H, *J* = 11.8 Hz), 1.11 (s, 3H), 1.22–1.28 (m, 1H), 1.31–1.48 (m, 5H), 1.48–1.55 (m, 4H), 1.60 (d, 1H, *J* = 13.9 Hz), 1.72–1.81 (m, 3H), 2.04 (d, 1H, *J* = 12.8 Hz), 2.55 (d, 1H, *J* = 12.3 Hz), 2.65 (d, 1H, *J* = 11.8 Hz), 3.22 (s, 1H), 3.57 (s, 3H), 3.72 (s, 2H), 4.02 (s, 1H), 4.51 (s, 2H), 7.13 (t, 2H, *J* = 8.4 Hz), 7.37 (t, 2H, *J* = 6.8 Hz); ^13^C-NMR (125 MHz, DMSO-d_6_) δ (ppm): 15.4 (CH_3_), 19.1 (CH_2_), 19.3 (CH_2_), 21.5 (CH_2_), 28.6 (CH_3_), 33.4 (CH_2_), 35.7 (CH_2_), 37.9 (CH_2_), 39.3 (C_q_), 40.3 (CH_2_), 42.6 (CH_2_), 43.6 (C_q_), 45.8 (C_q_), 51.5 (CH_3_), 51.7 (CH_2_), 53.0 (CH_2_), 53.3 (CH), 56.4 (CH), 77.1 (C_q_), 78.6 (C_q_), 82.1 (CH), 115.2 (CH), 115.3 (CH), 130.2 (CH), 130.3 (CH), 137.3 (C_q_), 160.6 (C_q-F_), 162.5 (C_q-F_), 177.5 (C=O). ^19^F-NMR (470 MHz, DMSO-d_6_) δ (ppm): -116.6 (C_q-F_). HRMS (ESI+): *m/z* calcd. for C_28_H_41_FNO_5_^+^ [M + H]^+^ 490.2963; found 490.2958 (mass error Δ_m_ = −1.08 ppm).

#### 3.3.6. (4*R*,6a*R*,7*R*,8*R*,9*S*,11b*S*)-Methyl 7,8,9-Trihydroxy-8-(((4-methoxybenzyl)amino)methyl)-4,11b-dimethyltetradecahydro-6a,9-methanocyclohepta[a]naphthalene-4-carboxylate (**10**)

The reaction was accomplished with 4-methoxybenzylamine, as described in the general procedure. Yield: 130 mg (96%); white crystals; m.p.: 161–163 °C; [*α*]_D_^20^ = −52 (*c* 0.0233 MeOH); ^1^H-NMR (500 MHz, DMSO-d_6_) δ (ppm): 0.72 (s, 3H), 0.75–0.81 (m, 1H), 0.86 (d, 1H, *J* = 7.8 Hz), 0.96–1.01 (m, 1H), 1.02 (d, 1H, *J* = 11.7 Hz), 1.11 (s, 3H), 1.22–1.27 (m, 1H), 1.31–1.47 (m, 5H), 1.48 (s, 1H), 1.51 (d, 2H, *J* = 5.4 Hz), 1.54 (s, 1H), 1.56–1.61 (m, 1H), 1.71–1.80 (m, 3H), 2.03 (d, 1H, *J* = 12.7 Hz), 2.54 (d, 1H, *J* = 12.2 Hz), 2.64 (d, 1H, *J* = 11.7 Hz), 3.20 (s, 1H), 3.56 (s, 3H), 3.64 (q, 2H, *J* = 4.4 Hz, 13.2 Hz), 3.73 (s, 3H), 4.08 (s, 1H), 4.45 (s, 2H), 6.86 (d, 2H, *J* = 7.8 Hz), 7.22 (d, 2H, *J* = 7.8 Hz); ^13^C-NMR (125 MHz, DMSO-d_6_) δ (ppm): 15.4 (CH_3_), 19.1 (CH_2_), 19.3 (CH_2_), 21.5 (CH_2_), 28.6 (CH_3_), 33.4 (CH_2_), 35.7 (CH_2_), 37.9 (CH_2_), 39.3 (C_q_), 40.3 (CH_2_), 42.6 (CH_2_), 43.6 (C_q_), 45.8 (C_q_), 51.5 (CH_3_), 51.7 (CH_2_), 53.3 (CH_2_), 55.5 (CH_3_), 56.4 (CH), 77.1 (C_q_), 78.6 (C_q_), 82.2 (CH), 114.0 (2xCH), 129.6 (2xCH), 133.1 (C_q_), 158.5 (C_q_), 177.5 (C=O). HRMS (ESI+): *m/z* calcd. for C_29_H_44_NO_6_^+^ [M + H]^+^ 502.3163; found 502.3156 (mass error Δ_m_ = −1.42 ppm).

#### 3.3.7. (4*R*,4a*S*,6a*R*,7*R*,8*R*,11a*S*,11b*S*)-Methyl 8-((((*R*)-1-(4-Fluorophenyl)ethyl)amino)methyl)-7,8,9-trihydroxy-4,11b-dimethyltetradecahydro-6a,9-methanocyclohepta[a]naphthalene-4-carboxylate (**11**)

The reaction was accomplished with (*R*)-4-fluoro-α-methylbenzylamine, as described in the general procedure. Yield: 68 mg (50%); white crystals; m.p.: 63–67 °C; [*α*]_D_^20^ = −28 (*c* 0.04 MeOH); ^1^H-NMR (500 MHz, DMSO-d_6_) δ (ppm): 0.71 (s, 3H), 0.73–0.78 (m, 1H), 0.82 (d, 1H, *J* = 8.8 Hz), 0.95–0.99 (m, 1H), 1.01 (d, 1H, *J* = 12.7 Hz), 1.10 (s, 3H), 1.24 (s, 1H), 1.26 (d, 3H, *J* = 5.9 Hz), 1.31–1.45 (m, 4H), 1.48 (s, 1H), 1.51 (s, 3H), 1.52–1.58 (m, 2H), 1.70–1.80 (m, 3H), 2.03 (d, 1H, *J* = 13.2 Hz), 2.36 (d, 1H, *J* = 11.5 Hz), 2.57 (d, 1H, *J* = 11.5 Hz), 3.17 (s, 1H), 3.55 (s, 3H), 3.76 (s, 1H), 4.02 (s, 1H), 4.51 (s, 2H), 7.12 (t, 2H, *J* = 8.7 Hz), 7.39 (t, 1H, *J* = 6.8 Hz); ^13^C-NMR (125 MHz, DMSO-d_6_) δ (ppm): 15.4 (CH_3_), 19.1 (CH_2_), 19.2 (CH_2_), 21.4 (CH_2_), 24.4 (CH_3_), 28.6 (CH_3_), 33.4 (CH_2_), 35.7 (CH_2_), 37.9 (CH_2_), 39.4 (C_q_), 40.4 (CH_2_), 42.6 (CH_2_), 43.6 (C_q_), 45.8 (C_q_), 50.4 (CH_2_), 51.4 (CH_3_), 53.4 (CH), 56.5 (CH), 57.9 (CH), 77.1 (C_q_), 78.6 (C_q_), 82.0 (CH), 115.3 (CH), 115.4 (CH), 128.9 (2xCH), 142.0 (C_q_), 160.3 (C_q-F_), 162.7 (C_q-F_), 177.5 (C=O). ^19^F-NMR (470 MHz, DMSO-d_6_) δ (ppm): -116.4 (C_q-F_). HRMS (ESI+): *m/z* calcd. for C_29_H_43_FNO_5_^+^ [M + H]^+^ 504.3120; found 504.3118 (mass error Δ_m_ = −0.35 ppm).

#### 3.3.8. (4*R*,6a*R*,7*R*,8*R*,9*S*,11b*S*)-Methyl 7,8,9-Trihydroxy-4,11b-dimethyl-8-((((*R*)-1-(naphthalen-2-yl)ethyl)amino)methyl)tetradecahydro-6a,9-methanocyclohepta[a]naphthalene-4-carboxylate (**12**)

The reaction was accomplished with (*R*)-1-(2-naphthyl)ethylamine, as described in the general procedure. Yield: 87 mg (60%); white crystals; m.p.: 93–95 °C; [*α*]_D_^20^ = +60 (*c* 0.04 MeOH); ^1^H-NMR (500 MHz, DMSO-d_6_) δ (ppm): 0.68 (s, 3H), 0.71 (s, 1H), 0.79 (d, 1H, *J* = 8.5 Hz), 0.90–0.98 (m, 1H), 0.99 (d, 1H, *J* = 12.4 Hz), 1.10 (s, 3H), 1.12–1.19 (m, 1H), 1.22–1.28 (m, 1H), 1.29–1.35 (m, 2H), 1.36 (d, 3H, *J* = 5.9 Hz), 1.39–1.47 (m, 2H), 1.49 (s, 1H), 1.51 (s, 2H), 1.55 (s, 1H), 1.62–1.73 (m, 2H), 1.77 (d, 1H, *J* = 12.4 Hz), 2.02 (d, 1H, *J* = 12.4 Hz), 2.42 (d, 1H, *J* = 11.7 Hz), 2.63 (d, 1H, *J* = 11.7 Hz), 3.23 (s, 1H), 3.34 (s, 1H), 3.55 (s, 3H), 3.93 (d, 1H, *J* = 5.2 Hz), 4.10–4.49 (m, 1H), 4.67 (s, 1H), 7.47 (q, 2H, *J* = 7.0 Hz), 7.58 (d, 1H, *J* = 8.4 Hz), 7.75–7.94 (m, 4H); ^13^C-NMR (125 MHz, DMSO-d_6_) δ (ppm): 15.3 (CH_3_), 19.0 (CH_2_), 19.2 (CH_2_), 21.4 (CH_2_), 24.6 (CH_3_), 28.6 (CH_3_), 33.4 (CH_2_), 35.7 (CH_2_), 37.9 (CH_2_), 39.3 (C_q_), 40.3 (CH_2_), 42.6 (CH_2_), 43.6 (C_q_), 45.8 (C_q_), 50.6 (CH_2_), 51.5 (CH_3_), 53.3 (CH), 56.3 (CH), 58.7 (CH), 77.1 (C_q_), 78.6 (C_q_), 81.9 (CH), 125.5 (CH), 125.9 (CH), 126.4 (2xCH), 127.97 (CH), 127.98 (CH), 128.4 (CH), 132.8 (C_q_), 133.5 (C_q_), 143.1 (C_q_), 177.5 (C=O). HRMS (ESI+): *m/z* calcd. for C_33_H_46_NO_5_^+^ [M + H]^+^ 536.3371; found 536.3369 (mass error Δ_m_ = −0.28 ppm).

#### 3.3.9. (4*R*,6a*R*,7*R*,8*R*,9*S*,11b*S*)-Methyl 7,8,9-Trihydroxy-4,11b-dimethyl-8-((((*S*)-1-(naphthalen-2-yl)ethyl)amino)methyl)tetradecahydro-6a,9-methanocyclohepta[a]naphthalene-4-carboxylate (**13**)

The reaction was accomplished with (*S*)-1-(2-naphthyl)ethylamine, as described in the general procedure. Yield: 79 mg (55%); white crystals; m.p.: 81–83 °C; [*α*]_D_^20^ = −40 (*c* 0.09 MeOH); ^1^H-NMR (500 MHz, DMSO-d_6_) δ (ppm): 0.69 (s, 3H), 0.71–0.76 (m, 1H), 0.83 (d, 1H, *J* = 8.5 Hz), 0.93–0.99 (m, 1H), 1.00 (d, 1H, *J* = 12.1 Hz), 1.10 (s, 3H), 1.21–1.27 (m, 2H), 1.28–1.35 (m, 2H), 1.38 (d, 5H, *J* = 6.7 Hz), 1.48 (s, 1H), 1.50 (s, 3H), 1.52–1.57 (m, 1H), 1.69 (d, 2H, *J* = 11.2 Hz), 1.78 (d, 1H, *J* = 13.9 Hz), 2.02 (d, 1H, *J* = 12.6 Hz), 2.51–2.57 (m, 2H, overlapped with DMSO-d_6_), 3.22 (s, 1H), 3.40 (s, 1H, overlapped with H_2_O), 3.55 (s, 3H), 3.95 (s, 1H), 4.57 (s, 2H), 7.48 (q, 2H, *J* = 6.6 Hz), 7.56 (d, 1H, *J* = 8.5 Hz), 7.81 (s, 1H), 7.85–7.90 (m, 3H); ^13^C-NMR (125 MHz, DMSO-d_6_) δ (ppm): 15.4 (CH_3_), 19.0 (CH_2_), 19.3 (CH_2_), 21.4 (CH_2_), 24.0 (CH_3_), 28.6 (CH_3_), 33.3 (CH_2_), 35.7 (CH_2_), 37.9 (CH_2_), 39.3 (C_q_), 40.3 (CH_2_), 42.5 (CH_2_), 43.6 (C_q_), 45.8 (C_q_), 50.6 (CH_2_), 51.5 (CH_3_), 53.3 (CH), 56.4 (CH), 58.6 (CH), 76.9 (C_q_), 78.6 (C_q_), 81.9 (CH), 125.4 (CH), 126.0 (CH), 126.5 (2xCH), 127.9 (CH), 128.0 (CH), 128.4 (CH), 132.8 (C_q_), 133.4 (C_q_), 143.1 (C_q_), 177.5 (C=O). HRMS (ESI+): *m/z* calcd. for C_33_H_46_NO_5_^+^ [M + H]^+^ 536.3371; found 536.3370 (mass error Δ_m_ = −0.09 ppm).

#### 3.3.10. (4*R*,6a*R*,7*R*,8*R*,9*S*,11b*S*)-Methyl 7,8,9-Trihydroxy-4,11b-dimethyl-8-(((naphthalen-1-ylmethyl)amino)methyl)tetradecahydro-6a,9-methanocyclohepta[a]naphthalene-4-carboxylate (**14**)

The reaction was accomplished with 1-naphtalenemethylamine, as described in the general procedure. Yield: 85 mg (60%); white crystals; m.p.: 138–140 °C; [*α*]_D_^20^ = −47 (*c* 0.07 MeOH); ^1^H-NMR (500 MHz, DMSO-d_6_) δ (ppm): 0.72 (s, 3H), 0.74–0.80 (m, 1H), 0.88 (s, 1H), 0.96–1.00 (m, 1H), 1.02 (d, 1H, *J* = 12.2 Hz), 1.11 (s, 3H), 1.16–1.32 (m, 2H), 1.37 (s, 1H), 1.39–1.47 (m, 3H), 1.48 (s, 1H), 1.52 (s, 3H), 1.60 (d, 1H, *J* = 7.5 Hz), 1.74 (s, 1H), 1.77 (s, 1H), 1.80 (s, 1H), 2.03 (d, 1H, *J* = 12.2 Hz), 2.74 (d, 1H, *J* = 12.2 Hz), 2.82 (d, 1H, *J* = 12.2 Hz), 3.26 (s, 1H), 3.56 (s, 3H), 3.88–4.14 (m, 1H), 4.21 (q, 2H, *J* = 13.2 Hz, 14.1 Hz), 4.49 (s, 2H), 7.46 (t, 1H, *J* = 7.3 Hz), 7.49–7.57 (m, 3H), 7.82 (d, 1H, *J* = 8.1 Hz), 7.92 (d, 1H, *J* = 7.7 Hz), 8.17 (d, 1H, *J* = 8.1 Hz); ^13^C-NMR (125 MHz, DMSO-d_6_) δ (ppm): 15.4 (CH_3_), 19.1 (CH_2_), 19.3 (CH_2_), 21.5 (CH_2_), 28.6 (CH_3_), 33.4 (CH_2_), 35.7 (CH_2_), 37.9 (CH_2_), 39.3 (C_q_), 40.3 (CH_2_), 42.6 (CH_2_), 43.6 (C_q_), 45.9 (C_q_), 51.4 (CH_3_), 51.5 (CH_2_), 52.5 (CH_2_), 53.3 (CH), 56.4 (CH), 77.2 (C_q_), 78.7 (C_q_), 82.1 (CH), 124.4 (CH), 125.9 (CH), 126.1 (CH), 126.3 (CH), 126.4 (CH), 127.8 (CH), 128.9 (CH), 131.9 (C_q_), 133.8 (C_q_), 136.5 (C_q_), 177.5 (C=O). HRMS (ESI+): *m/z* calcd. for C_32_H_44_NO_5_^+^ [M + H]^+^ 522.3214; found 522.3216 (mass error Δ_m_ = 0.38 ppm).

#### 3.3.11. (4*R*,6a*R*,7*R*,8*R*,9*S*,11b*S*)-Methyl 7,8,9-Trihydroxy-4,11b-dimethyl-8-((((*R*)-1-phenylpropyl)amino)methyl)tetradecahydro-6a,9-methanocyclohepta[a]naphthalene-4-carboxylate (**15**)

The reaction was accomplished with (*R*)-(+)-α-ethylbenzylamine, as described in the general procedure. Yield: 108 mg (80%); white crystals; m.p.: 138–141 °C; [[*α*]_D_^20^ = −15 (*c* 0.1267 MeOH); ^1^H-NMR (500 MHz, DMSO-d_6_) δ (ppm): 0.69 (s, 3H), 0.72 (s, 1H), 0.76 (t, 3H, *J* = 7.2 Hz), 0.79 (d, 1H, *J* = 8.5 Hz), 0.93–0.99 (m, 1H), 1.00 (d, 1H, *J* = 12.4 Hz), 1.10 (s, 3H), 1.11–1.18 (m, 1H), 1.22–1.25 (m, 1H), 1.32–1.42 (m, 3H), 1.47 (s, 1H), 1.50 (s, 3H), 1.51–1.59 (m, 2H), 1.61–1.81 (m, 5H), 2.02 (d, 1H, *J* = 13.5 Hz), 2.34 (d, 1H, *J* = 11.8 Hz), 2.45- 2.49 (m, 1H, overlapped with DMSO-d_6_), 3.13 (s, 1H), 3.44 (s, 1H), 3.55 (s, 3H), 4.06 (s, 1H), 4.50 (s, 2H), 7.21 (t, 1H, *J* = 4.3 Hz), 7.30 (d, 4H, *J* = 4.0 Hz); ^13^C-NMR (125 MHz, DMSO-d_6_) δ (ppm): 11.2 (CH_3_), 15.4 (CH_3_), 19.0 (CH_2_), 19.2 (CH_2_), 21.4 (CH_2_), 28.6 (CH_3_), 31.1 (CH_2_), 33.6 (CH_2_), 35.7 (CH_2_), 37.9 (CH_2_), 39.3 (C_q_), 40.3 (CH_2_), 42.5 (CH_2_), 43.6 (C_q_), 45.8 (C_q_), 50.7 (CH_2_), 51.5 (CH_3_), 53.3 (CH), 56.4 (CH), 65.3 (CH), 77.2 (C_q_), 78.6 (C_q_), 81.9 (CH), 127.2 (CH), 127.6 (2xCH), 128.6 (2xCH), 144.7 (C_q_), 177.5 (C=O). HRMS (ESI+): *m/z* calcd. for C_30_H_46_NO_5_^+^ [M + H]^+^ 500.3371; found 500.3369 (mass error Δ_m_ = −0.30 ppm).

#### 3.3.12. (4*R*,6a*R*,7*R*,8*R*,9*S*,11b*S*)-Methyl 7,8,9-Trihydroxy-4,11b-dimethyl-8-((((*S*)-1-phenylpropyl)amino)methyl)tetradecahydro-6a,9-methanocyclohepta[a]naphthalene-4-carboxylate (**16**)

The reaction was accomplished with *S*-(-)-α-ethylbenzylamine, as described in the general procedure. Yield: 88 mg (65%); white crystals; m.p.: 140–143 °C; [*α*]_D_^20^ = +126 (*c* 0.1 MeOH); ^1^H-NMR (500 MHz, DMSO-d_6_) δ (ppm): 0.70 (s, 3H), 0.73 (t, 4H, *J* = 7.3 Hz), 0.77 (s, 1H), 0.82 (d, 1H, *J* = 8.5 Hz), 0.94–0.99 (m, 1H), 1.00 (d, 1H, *J* = 12.0 Hz), 1.10 (s, 3H), 1.21–1.27 (m, 2H), 1.32–1.38 (m, 3H), 1.47 (s, 1H), 1.50 (s, 3H), 1.51–1.55 (m, 2H), 1.64–1.69 (m, 1H), 1.72 (d, 2H, *J* = 12.5 Hz), 1.78 (d, 1H, *J* = 13.6 Hz), 2.02 (d, 1H, *J* = 13.1 Hz), 2.40 (d, 1H, *J* = 12.0 Hz), 2.46 (d, 1H, *J* = 12.0 Hz), 3.14 (s, 1H), 3.44 (s, 1H), 3.55 (s, 3H), 3.97 (s, 1H), 4.39 (s, 1H), 4.51 (s, 1H), 7.21 (t, 1H, *J* = 6.8 Hz), 7.27 (d, 2H, *J* = 7.3 Hz), 7.31 (t, 2H, *J* = 7.3 Hz); ^13^C-NMR (125 MHz, DMSO-d_6_) δ (ppm): 11.2 (CH_3_), 15.4 (CH_3_), 19.1 (CH_2_), 19.3 (CH_2_), 21.4 (CH_2_), 28.6 (CH_3_), 30.8 (CH_2_), 33.3 (CH_2_), 35.7 (CH_2_), 37.9 (CH_2_), 39.3 (C_q_), 40.3 (CH_2_), 42.6 (CH_2_), 43.6 (C_q_), 45.8 (C_q_), 50.4 (CH_2_), 51.5 (CH_3_), 53.3 (CH), 56.4 (CH), 64.9 (CH), 76.9 (C_q_), 78.6 (C_q_), 82.3 (CH), 127.2 (CH), 127.6 (2xCH), 128.6 (2xCH), 144.8 (C_q_), 177.5 (C=O). HRMS (ESI+): *m/z* calcd. for C_30_H_46_NO_5_^+^ [M + H]^+^ 500.3371; found 500.3371 (mass error Δ_m_ = 0.10 ppm).

#### 3.3.13. (4*R*,6a*R*,7*R*,8*R*,9*S*,11b*S*)-Methyl 8-(Aminomethyl)-7,8,9-trihydroxy-4,11b-dimethyltetradecahydro-6a,9-methanocyclohepta[a]naphthalene-4-carboxylate (**17**)

80 mg (0.21 mmol) of **5** was dissolved in MeOH (22 mL) and added to a suspension of palladium-on-carbon (Pd/C, 0.01 g) in MeOH (5 mL). The mixture was stirred at room temperature for 24 h under a H_2_ atmosphere (1 atm), then filtered through a Celite pad, and the resulting solution was evaporated to dryness. The product was purified by recrystallisation in Et_2_O, resulting in primary aminotriol **17**. Yield: 33 mg (41%); white crystals; m.p.: 152–155 °C; [*α*]_D_^20^ = −41 (*c* 0.08 MeOH); ^1^H-NMR (500 MHz, DMSO-d_6_) δ (ppm): 0.74 (s, 3H), 0.77–0.83 (m, 1H), 0.90 (d, 1H, *J* = 7.4 Hz), 0.97–1.02 (m, 1H), 1.05 (d, 1H, *J* = 11.8 Hz), 1.11 (s, 3H), 1.24–1.30 (m, 1H), 1.37 (d, 1H, *J* = 13.6 Hz), 1.43–1.48 (m, 1H), 1.49–1.56 (m, 4H), 1.58 (s, 2H), 1.65 (d, 1H, *J* = 10.1 Hz), 1.72–1.83 (m, 3H), 2.04 (d, 1H, *J* = 13.8 Hz), 2.83 (q, 2H, *J* = 12.8 Hz, 14.9 Hz), 3.09–3.34 (m, 2H), 3.36 (s, 1H), 3.37–3.49 (m, 1H), 3.57 (s, 3H), 5.75 (s, 2H); ^13^C-NMR (125 MHz, DMSO-d_6_) δ (ppm): 15.4 (CH_3_), 18.9 (CH_2_), 19.1 (CH_2_), 21.4 (CH_2_), 28.6 (CH_3_), 33.2 (CH_2_), 35.4 (CH_2_), 37.9 (CH_2_), 39.3 (C_q_), 40.3 (CH_2_), 42.4 (CH_2_), 42.9 (CH_2_), 43.6 (C_q_), 45.8 (C_q_), 51.5 (CH_3_), 53.3 (CH), 56.3 (CH), 75.7 (C_q_), 78.6 (C_q_), 80.5 (CH), 177.5 (C=O). HRMS (ESI+): *m/z* calcd. for C_21_H_36_NO_5_^+^ [M + H]^+^ 382.2588; found 382.2587 (mass error Δ_m_ = −0.26 ppm).

#### 3.3.14. (4*R*,5’*R*,6a*R*,7*R*,9*S*,11b*S*)-Methyl 3’-Benzyl-7,9-dihydroxy-4,11b-dimethyldodecahydro-1H-spiro [6a,9-methanocyclohepta[a]naphthalene-8,5’-oxazolidine]-4-carboxylate (**18**)

To a solution of aminotriol **5** (70 mg, 0.15 mmol) in Et_2_O (15 mL) 3.7 mL of aqueous formaldehyde (35%) was added, and the mixture was stirred at room temperature for 1 h. After the completion of the reaction to make the mixture alkaline, 10% aqueous KOH (10 mL) was added, and the extraction followed with Et_2_O (3 × 50 mL). The organic phases were dried (Na_2_SO_4_) and the solvent was evaporated before crude product **18** was purified by column chromatography on silica gel with *n*-hexane/EtOAc 1:9. Yield: 64 mg (88%); white crystals; m.p.: 123–125 °C; [*α*]_D_^20^ = +76 (*c* 0.05 MeOH); ^1^H-NMR (500 MHz, DMSO-d_6_) δ (ppm): 0.73 (s, 3H), 0.75–0.80 (m, 1H), 0.86 (d, 1H, *J* = 7.8 Hz), 0.96–1.01 (m, 1H), 1.04 (d, 1H, *J* = 12.3 Hz), 1.11 (s, 3H), 1.21–1.31 (m, 3H), 1.35 (d, 1H, *J* = 14.3 Hz), 1.44 (d, 3H, *J* = 10.4 Hz), 1.53 (s, 1H), 1.56 (s, 1H), 1.61 (d, 1H, *J* = 14.3 Hz), 1.68 (d, 1H, *J* = 11.0 Hz), 1.74 (s, 1H), 1.77 (d, 1H, *J* = 11.0 Hz), 2.03 (d, 1H, *J* = 13.0 Hz), 2.97 (q, 2H, *J* = 11.7 Hz, 16.2 Hz), 3.24 (d, 1H, *J* = 8.4 Hz), 3.56 (s, 3H), 3.73 (d, 1H, *J* = 13.7 Hz), 3.74 (d, 1H, *J* = 7.2 Hz), 3.79 (d, 1H, *J* = 13.0 Hz), 4.23 (s, 1H), 4.26 (d, 1H, *J* = 5.2 Hz), 4.46 (d, 1H, *J* = 5.2 Hz), 7.25 (s, 1H), 7.31 (s, 4H); ^13^C-NMR (125 MHz, DMSO-d_6_) δ (ppm): 15.7 (CH_3_), 19.1 (CH_2_), 19.2 (CH_2_), 21.5 (CH_2_), 28.6 (CH_3_), 33.8 (CH_2_), 35.7 (CH_2_), 37.9 (CH_2_), 39.3 (C_q_), 40.3 (CH_2_), 43.4 (CH_2_), 43.6 (C_q_), 45.8 (C_q_), 51.5 (CH_3_), 52.9 (CH), 56.5 (CH), 56.9 (CH_2_), 57.6 (CH_2_), 77.9 (C_q_), 85.8 (C_q_), 85.9 (CH), 87.8 (CH_2_), 127.3 (CH), 128.7 (2xCH), 129.0 (2xCH), 139.9 (C_q_), 177.6 (C=O). HRMS (ESI+): *m/z* calcd. for C_29_H_42_NO_5_^+^ [M + H]^+^ 484.3057; found 484.3058 (mass error Δ_m_ = 0.10 ppm).

### 3.4. Analytical Method for Physicochemical Investigations

Filtrates and calibration solutions (5–500 μM) of the kinetic solubility study and starting donor and acceptor solutions of the PAMPA-GI study were investigated by HPLC-DAD-MS. LC-MS analysis was performed using a Waters 2795 HPLC with a Waters 2487 DAD detector coupled with a Micromass Quattro Ultima TMPt quadrupol mass spectrometer equipped with an ESI source. Measurements were performed on a Waters XSelect C18 (150 × 4.6 mm; 3.5 μm) column with a 1.0 mL/h eluent flow rate and the temperature set at 40 °C. The composition of eluent A was MilliQ water with 0.1% formic acid, and that of eluent B was an acetonitrile and MilliQ water mixture (95:5, *v*/*v*), also with 0.1% formic acid. The following linear gradient program was used: 0–6 min, 10–100% B; this was held for 3 min, then from 9.01 min on, back to the starting composition of 10% B–90% A. The injection volume was 10 μL for each sample. Chromatographic profiles were registered at dual wavelength, 220 and 240 nm. The mass spectrometer was used in negative ion mode for samples **2**, **3** and **4b**, and in positive ion mode for other samples. The operating parameters of the MS were: Mass range of m/z 150–700. Nitrogen gas flow rate was 350 L/h, temperature of 350 °C and pressure of 6 bar. The quadrupol temperature was 120 °C, and the capillary voltage was 2.5 kV. Fragmentor’s voltage of 60 V.

### 3.5. Kinetic Solubility and PAMPA-GI Measurements

Intestinal (GI) permeability was determined by using an intestinal-specific parallel artificial membrane permeability assay (PAMPA-GI [36]]). Briefly, kinetic solubility was performed with 5% DMSO as a cosolvent in phosphate buffered saline (PBS, 0.01 M phosphate buffer pH 7.4) at 37 °C for 2 h using 96-well Multi-Screen HTS-PCF Filter Plates (Merck Ltd., Budapest, Hungary). All solutions were filtered by MultiScreen HTS Vacuum Manifold (Merck Ltd., Budapest, Hungary) after the incubation period. The target concentration was 500 μM for every sample investigated. The intestinal-specific effective permeability of the samples was determined by PAMPA-GI. To prepare starting solutions for a 96-well donor plate, 15 μLs of the 10 mM DMSO stock solution of investigation compounds and 285 μL pH 6.5 phosphate buffer solution were mixed. These solutions were left in a shaking incubator (300 rpm) at 25 °C for 1 h. Meanwhile, a 96-well acceptor plate was filled with 300 μL of pH 7.4 PBS buffer solution containing 5% DMSO. The intestinal-specific artificial membrane was prepared the following way: the hydrophobic filter material of the donor plate was coated with a mixture of phosphatidylcholine/cholesterol = 2:1 (5 μL, 4 % (*w*/*v*) in dodecane). After that, all donor wells were filled with 150 μL of the starting donor solutions, and they were carefully placed on the acceptor plate and incubated at 37 °C for 4 h. After the end of the incubation period the donor and acceptor plates were separated carefully. A total of 150 μL of the donor, acceptor and the starting donor solutions were sampled and analysed with HPLC-DAD-MS. The effective permeability and membrane retention of drugs were calculated using the following equations:

$$P_{e}=\frac{-2.303}{A\cdot \left(t-\tau_{ss}\right)}\cdot \frac{1}{1+r_{v}}\cdot lg\left[-r_{v}+\left(\frac{1+r_{v}}{1-MR}\right)\cdot \right]\frac{C_{D}\left(t\right)}{C_{D}\left(0\right)}$$

$$MR=1-\frac{C_{D}\left(t\right)}{C_{D}\left(0\right)}-\frac{V_{A}\cdot C_{A}\left(t\right)}{V_{D}\cdot C_{D}\left(0\right)}$$

where P_e_ is the effective permeability coefficient (cm/s), A is the filter area (0.3 cm^2^), V_D_ and V_A_ are the volumes in the donor (0.15 cm^3^) and acceptor phase (0.3 cm^3^), t is the incubation time (s), τ_SS_ is the time to reach steady-state (s), C_D_(t) is the concentration of the compound in the donor phase at time point t (mol/cm^3^), C_D_(0) is the concentration of the compound in the donor phase at time point zero (mol/cm^3^), r_v_ is the aqueous compartment volume ratio (V_D_/V_A_), MR is the membrane retention factor.

### 3.6. Determination of the Antiproliferative Activities

The growth-inhibitory effects of the presented terpenes were determined by a standard MTT [3-(4,5-dimethylthiazol2-yl)-2,5-diphenyltetrazolium bromide] assay against four human cancer cell lines (cervical cancer HeLa and SiHa cell lines, breast cancer MCF-7 and MDA-MB-231 and ovarian cancer A2780) [42]. Murine embryonal fibroblast cells (NIH/3T3) were used to determine selectivity. All cell lines were purchased from the European Collection of Cell Cultures (Salisbury, UK). The cells were cultivated in Eagle’s minimal essential medium supplemented with 10% fetal bovine serum, 1% non-essential amino acids, and 1% Antibiotic-Antimycotic complex (penicillin, streptomycin, amphotericin B) at 37 °C in a humidified atmosphere containing 5% CO_2_. All media and supplements were purchased from Lonza Group Ltd. (Basel, Switzerland). Cancer cells were seeded into 96-well plates (5000 cells/well) after an overnight incubation, and the test compounds were added at two different concentrations (10 μM and 30 μM) and incubated for another 72 h under cell-culturing conditions. Finally, 20 μL of a 5 mg/mL MTT solution was added to each well, and the contents were incubated for a further 4 h. The medium was removed, and the precipitated formazan crystals were dissolved in DMSO by shaking at 37 °C for 60 min. The absorbance was measured at 545 nm by using a microplate reader (SPECTROStar Nano, BMG Labtech, Offenburg, Germany). Clinically utilised anticancer agent cisplatin (Ebewe GmbH, Unterach, Austria) was included as a reference molecule. Calculations were performed using the GraphPad Prism 9 software (GraphPad Software Inc., San Diego, CA, USA).

## 4. Conclusions

In summary, a library of new steviol derivatives has been prepared by stereoselective transformations with moderate to excellent yields, and their physicochemical characterisation and antiproliferative activities against five human tumour cell lines (HeLa, SiHa, A2780, MCF-7 and MDA-MB-231) have been investigated. Using commercially available stevioside as starting material, a novel series of steviol-based derivatives carrying aminotriol and oxazolidine moieties was synthesised using spiro-epoxide methyl ester as the key intermediate. Based on the in vitro pharmacological studies, the resulting aminotriols exhibit a significant cytostatic effect on human cancer cell lines. The investigation further confirmed the essential role of the *N*-benzyl substituent at the amino function, displaying antiproliferative properties and showed that substituents such as the naphthyl and the *p*-fluorophenyl groups are beneficial for the increased activity. Selectivity for the breast cancer cell lines (MCF-7, MDA-MB-231) was detectable in the case of *p*-fluorobenzyl-, *p*-methoxybenzyl- and α-ethylbezyl-functionalised compounds and a spiro-oxazolidine derivative. Further optimisation of the *N*-substituent and examination of the ester functional group on the A ring may lead to pharmacologically valuable compounds.

## Figures and Tables

**Table 1:** Stereoselective syntheses of steviol-derived aminotriols.

Entry	Product	R^1^	R^2^	Yield [%]
1	**5**	H	benzyl	93
2	**6**	Me	benzyl	45
3	**7**	H	(*R*)-α-methylbenzyl	59
4	**8**	H	(*S*)-α-methylbenzyl	60
5	**9**	H	4-fluorobenzyl	95
6	**10**	H	4-methoxybenzyl	96
7	**11**	H	(*R*)-4-fluoro-α-methylbenzyl	50
8	**12**	H	(*R*)-1-(2-naphthyl)ethyl	60
9	**13**	H	(*S*)-1-(2-naphthyl)ethyl	55
10	**14**	H	1-naphthylmethyl	60
11	**15**	H	(*R*)-(+)-α-ethylbenzyl	80
12	**16**	H	(*S*)-(-)-α-ethylbenzyl	65

**Table 2:** Predicted physicochemical parameters of steviol-based aminotriols using the ACD/Labs Percepta software package. MW: molecular weight, TPSA: topological polar surface area. Lipinski’s rule of 5 (Ro5) for estimation of drug-like properties.

Product	MW ^a^	pKa,*_base_*/pKa,*_acid_*	log*P^a^*/log*D*_7.4_	TPSA ${Å}^{2}$	Lipinski Ro5 Violation ^a^
**2**	334	-/4.6	3.2/0.4	77.8	No
**3**	348	-/-	3.8/3.8	66.8	No
**4b**	364	-/-	2.6/2.6	79.3	No
**5**	472	9.5/-	4.5/2.8	99	No
**6**	486	8.6/-	4.8/4.0	90.2	No
**7**	486	9.6/-	4.8/3.1	99	No
**8**	486	9.6/-	4.8/3.1	99	No
**9**	490	9.5/-	4.4/2.8	99	No
**10**	502	9.9/-	4.3/2.3	108.3	Moderate: Mw
**11**	504	9.6/-	4.8/3.2	99	Moderate: Mw
**12**	536	9.3/-	5.7/4.3	99	High: Mw, logP
**13**	536	9.3/-	5.7/4.3	99	High: Mw, logP
**14**	522	9.2/-	5.5/4.2	99	High: Mw, logP
**15**	500	9.6/-	5.2/3.5	99	Moderate: logP
**16**	500	9.6/-	5.2/3.5	99	Moderate: logP
**17**	382	10.2/-	2.3/-0.1	113	No
**18**	484	5.2/-	4.1/4.1	79.2	No

^a^ Lipinski’s Ro5 violation for molecular weight (MW > 500, moderate risk: light grey, high risk: dark grey) and lipophilicity (logP > 5, moderate risk: light grey, high risk: dark grey).

**Table 3:** Experimental physicochemical characterisation of steviol-based aminotriols. P_e_: effective permeability, MR: membrane retention. Kinetic aqueous solubility, P_e_ and MR values of PAMPA-GI measurements represent mean ± S.E.M, *n* = 3.

Products	Kinetic Solubility ^a^ (μM)	PAMPA-GI	
		P_e_ ^b^ (10^−7^ cm/s)	MR ^c^ (%)
**2**	443.4 ± 35.7	5.2 ± 0.2	6.5 ± 0.7
**3**	ND ^d^	ND ^d^	ND ^d^
**4b**	ND ^d^	ND ^d^	ND ^d^
**5**	290.8 ± 35.5	6.4 ± 0.3	29.8 ± 0.3
**6**	114.3 ± 16.0	2.3 ± 0.8	67.3 ± 8.6
**7**	181.6 ± 3.2	6.4 ± 0.7	28.8 ± 5.8
**8**	131.3 ± 2.2	5.8 ± 0.1	42.6 ± 3.9
**9**	42.1 ± 10.8	4.0 ± 0.4	63.8 ± 4.9
**10**	142.9 ± 26.8	3.7 ± 1.1	6.1 ± 1.1
**11**	145.4 ± 14.1	4.8 ± 0.5	22.4 ± 2.0
**12**	13.3 ± 0.9	ND ^e^	92.6 ± 2.1
**13**	11.9 ± 1.8	ND ^e^	92.6 ± 1.1
**14**	18.9 ± 2.4	ND ^e^	94.2 ± 2.0
**15**	145.0 ± 28.2	1.4 ± 0.4	46.8 ± 3.3
**16**	89.9 ± 8.5	2.5 ± 0.9	44.6 ± 4.2
**17**	535.4 ± 38.4	ND ^e^	1.5 ± 0.3
**18**	321.1 ± 88.5	ND ^e^	2.7 ± 0.4

^a^ After 2 h, at 37 °C in PBS, pH 7.4; classification for kinetic solubility (μM): good (non-greyed ≥ 100) moderate (100 > light grey ≥ 40), poor (dark grey < 40), ^b^ Gradient pH system (pH_donor_6.5→pH_acceptor_7.4) after 4 h, at 37 °C; classification for PAMPA-GI permeability, P_e_ (10^−6^ cm/s): medium (non-greyed ≥ 5), low (5 > light grey ≥ 3), very-low (dark grey < 3), ^c^ Increased lipid membrane retantion (MR% > 90), indicating a strong interaction between the active substance and the artificial lipid membrane, ^d^ ND: not detectable (3 and 4b compounds have no chromophoric group, and these compounds were also not ionised in either positive or negative ESI mode), ^e^ ND: not determined (the compound cannot be detected in the acceptor side).

## Data Availability

Data sharing is not applicable to this article.

## Supplementary Materials

The supporting information can be downloaded at: https://www.mdpi.com/article/10.3390/ijms24021121/s1.
