# Supplementary material for: Stereoselective Synthesis and Antiproliferative Activities of Tetrafunctional Diterpene Steviol Derivatives
Source: Int J Mol Sci. 2023 Jan 6;24(2):1121. doi: 10.3390/ijms24021121 (PMC9861858; doi:10.3390/ijms24021121)

Supporting Informations

Contents

Investigation of antiproliferative activity of aminotriols S3
^{1}H, ^{13}C, ^{19}F NMR, COSY, NOESY, HSQC, HMBC spectra of new compounds S4 - 101
HRMS spectra of new compounds S102 - 117

Table S1 *In vitro* antiproliferative activity of the prepared compounds

Comp.	Conc. (µM)	Growth inhibition (%) ± SEM[1]					
		HeLa	SiHa	MCF-7	MDA-MB-231	A2780	NIH/3T3
**3**	10	<20[2]	<20	<20	<20	<20	<20
	30	27.92 ± 2.30	30.73 ± 2.71	49.20 ± 2.19	<20	<20	<20
**4b**	10	30.61 ± 1.09	<20	<20	<20	<20	<20
	30	95.35 ± 0.11	57.98 ± 2.66	53.11 ± 2.20	36.70 ± 1.62	61.99 ± 0.78	37.17 ± 0.71
**5**	10	<20	<20	59.60 ± 2.73	34.59± 2.98	30.35± 2.48	<20
	30	95.77 ± 0.26	<20	96.42 ± 0.27	95.80 ± 0.63	97.15 ± 0.21	101.1 ± 0.43
**6**	10	<20	<20	<20	<20	25.73 ± 2.70	<20
	30	<20	<20	51.71 ± 2.54	82.56 ± 0.69	63.73 ± 1.98	89.32 ± 2.62
**7**	10	33.68 ± 0.93	<20	62.79 ± 1.05	88.05 ± 0.59	52.85 ± 1.83	35.79 ± 4.12
	30	97.76 ± 0.25	94.56 ± 0.54	97.69 ± 0.38	94.56 ± 0.63	98.14 ± 0.36	100.50 ± 0.37
**8**	10	<20	<20	73.99 ± 2.14	84.31 ± 2.78	59.85 ± 3.03	58.81 ± 1.41
	30	96.19 ± 0.25	95.09 ± 0.69	96.04 ± 0.32	95.62 ± 0.92	97.45 ± 0.36	103.20 ± 0.33
**9**	10	42.54 ± 1.42	54.60 ± 3.42	96.40 ± 0.47	83.03 ± 1.71	45.60 ± 3.13	25.90 ± 0.94
	30	96.80 ± 0.74	95.17 ± 0.37	96.19 ± 0.65	93.63 ± 0.36	96.97 ± 0.29	92.44 ± 1.01
**10**	10	27.79 ± 1.05	24.10 ± 1.39	74.32 ± 2.62	37.79 ± 2.27	<20	<20
	30	96.06 ± 0.93	94.64 ± 0.50	95.66 ± 0.76	93.42 ± 0.56	96.79 ± 0.25	90.93 ± 1.17
**11**	10	95.41 ± 1.08	93.66 ± 0.85	96.23 ± 0.59	93.66 ± 0.59	97.06 ± 0.30	44.42 ± 0.70
	30	95.75 ± 1.11	94.59 ± 0.49	96.27 ± 0.65	94.31 ± 0.51	97.51 ± 0.22	90.55 ± 1.16
**12**	10	96.82 ± 0.56	96.08 ± 0.60	96.35 ± 0.36	95.84 ± 0.29	96.68 ± 0.36	90.07 ± 0.51
	30	96.94 ± 0.49	95.49 ± 0.86	95.87 ± 0.38	95.62 ± 0.34	96.38 ± 0.30	94.67 ± 0.24
**13**	10	94.50 ± 1.24	93.78 ± 1.81	95.74 ± 0.64	93.33 ± 0.29	96.64 ± 0.35	72.02 ± 1.81
	30	94.91 ± 1.29	93.77 ± 0.41	95.44 ± 0.85	93.21 ± 0.46	96.79 ± 0.20	90.55 ± 1.31
**14**	10	97.20 ± 0.92	97.24 ± 0.39	97.57 ± 0.19	97.41 ± 0.21	92.19 ± 3.33	93.25 ± 0.51
	30	98.14 ± 0.36	96.70 ± 0.65	97.66 ± 0.28	96.46 ± 0.34	96.81 ± 0.23	97.34 ± 0.26
**15**	10	63.56 ± 1.54	22.35 ± 1.42	90.95 ± 1.52	68.30 ± 2.86	50.02 ± 2.99	20.97 ± 0.96
	30	97.25 ± 0.50	95.67 ± 0.76	96.47 ± 0.26	95.63 ± 0.45	96.64 ± 0.24	94.23 ± 0.31
**16**	10	53.54 ± 1.99	<20	80.66 ± 2.37	39.29 ± 1.99	48.34 ± 2.17	<20
	30	98.33 ± 0.32	96.73 ± 0.65	97.10 ± 0.23	96.65 ± 0.37	96.99 ± 0.19	95.12 ± 0.41
**17**	10	<20	<20	<20	<20	<20	<20
	30	28.87 ± 2.72	<20	<20	30.39 ± 2.04	<20	<20
**18**	10	<20	<20	<20	<20	<20	<20
	30	41.12 ± 1.95	66.08 ± 2.45	95.18 ± 0.35	87.67 ± 0.91	58.51 ± 3.18	<20

[1]: Mean ± SEM values from two determinations with 5 parallel wells in each.

[2]: Cancer cell growth inhibition values less than 20% were considered negligible and are not given numerically

^{1}H-NMR of compound (4*R*,6a*R*,7*R*,9*S*,11b*S*)-methyl 7,9-dihydroxy-4,11b-dimethyl-8-methylenetetradecahydro-6a,9-methanocyclohepta[a]naphthalene-4-carboxylate **(3)**

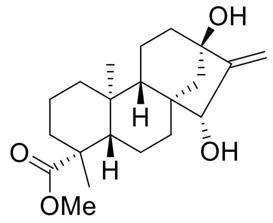

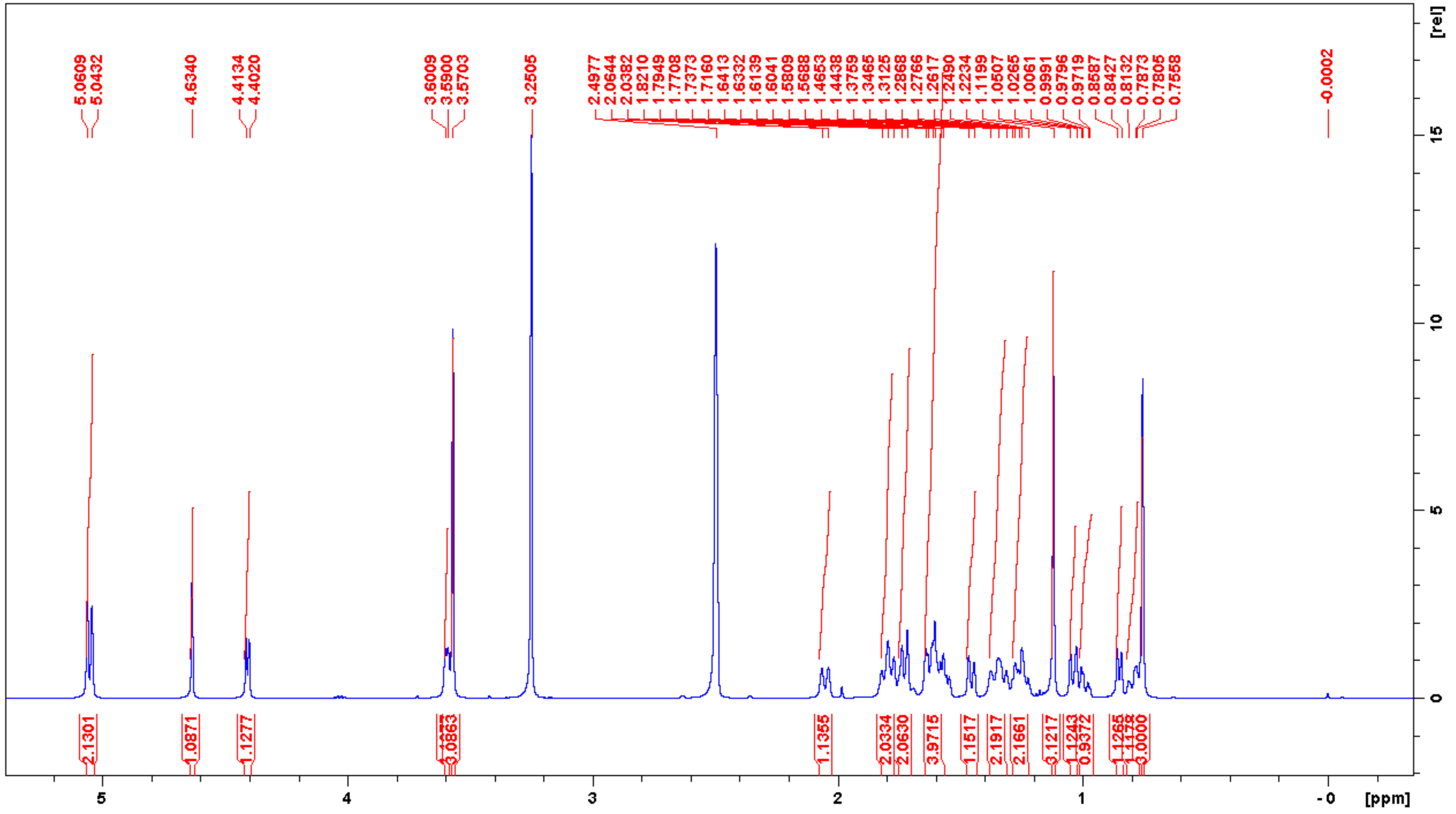

^{13}C-NMR of compound (4*R*,6a*R*,7*R*,9*S*,11b*S*)-methyl 7,9-dihydroxy-4,11b-dimethyl-8-methylenetetradecahydro-6a,9-methanocyclohepta[a]naphthalene-4-carboxylate **(3)**

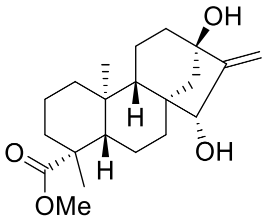

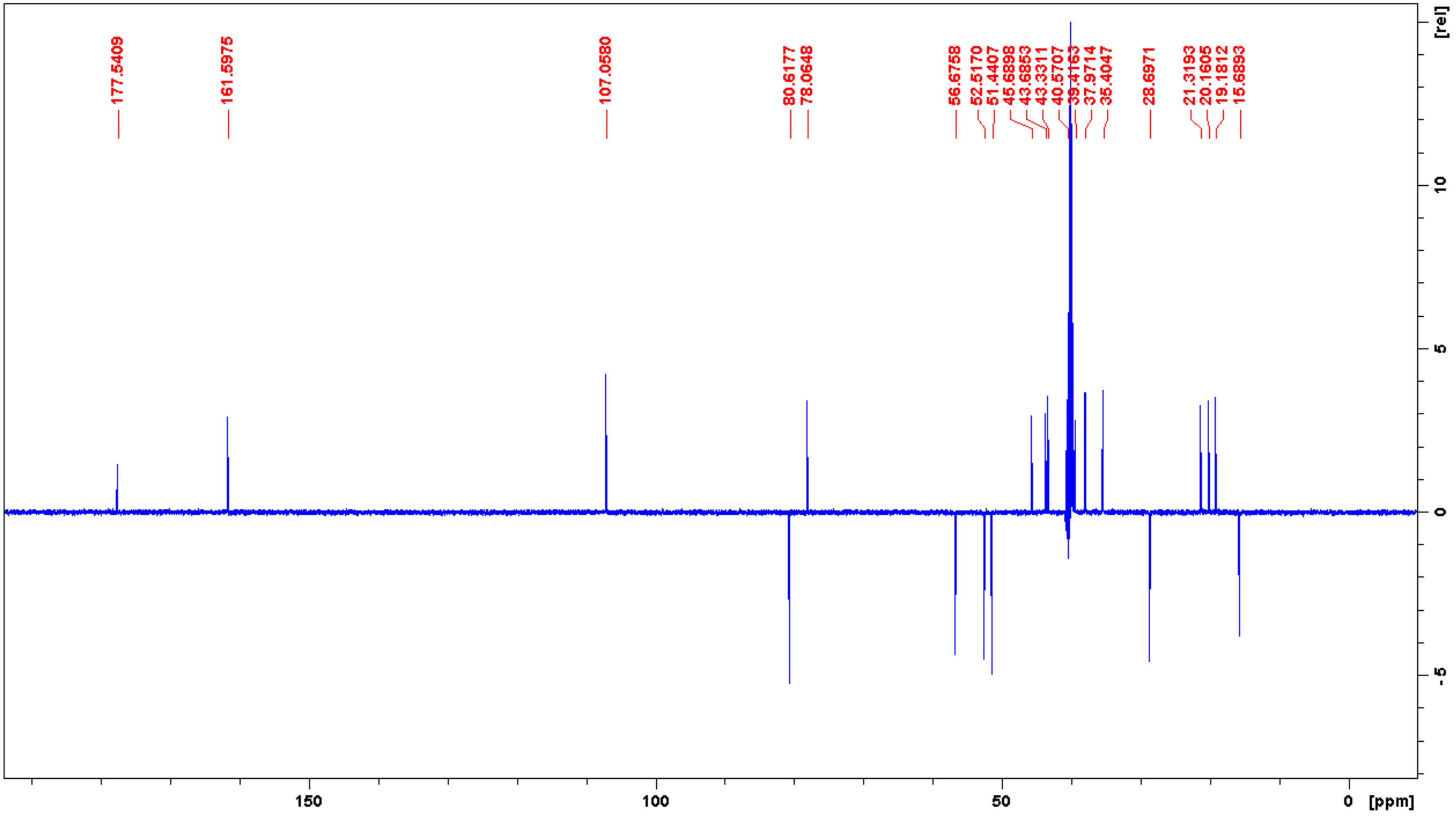

COSY of compound (4*R*,6a*R*,7*R*,9*S*,11b*S*)-methyl 7,9-dihydroxy-4,11b-dimethyl-8-methylenetetradecahydro-6a,9-methanocyclohepta[a]naphthalene-4-carboxylate **(3)**

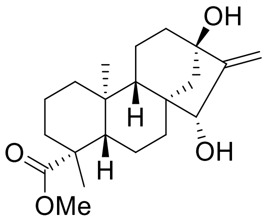

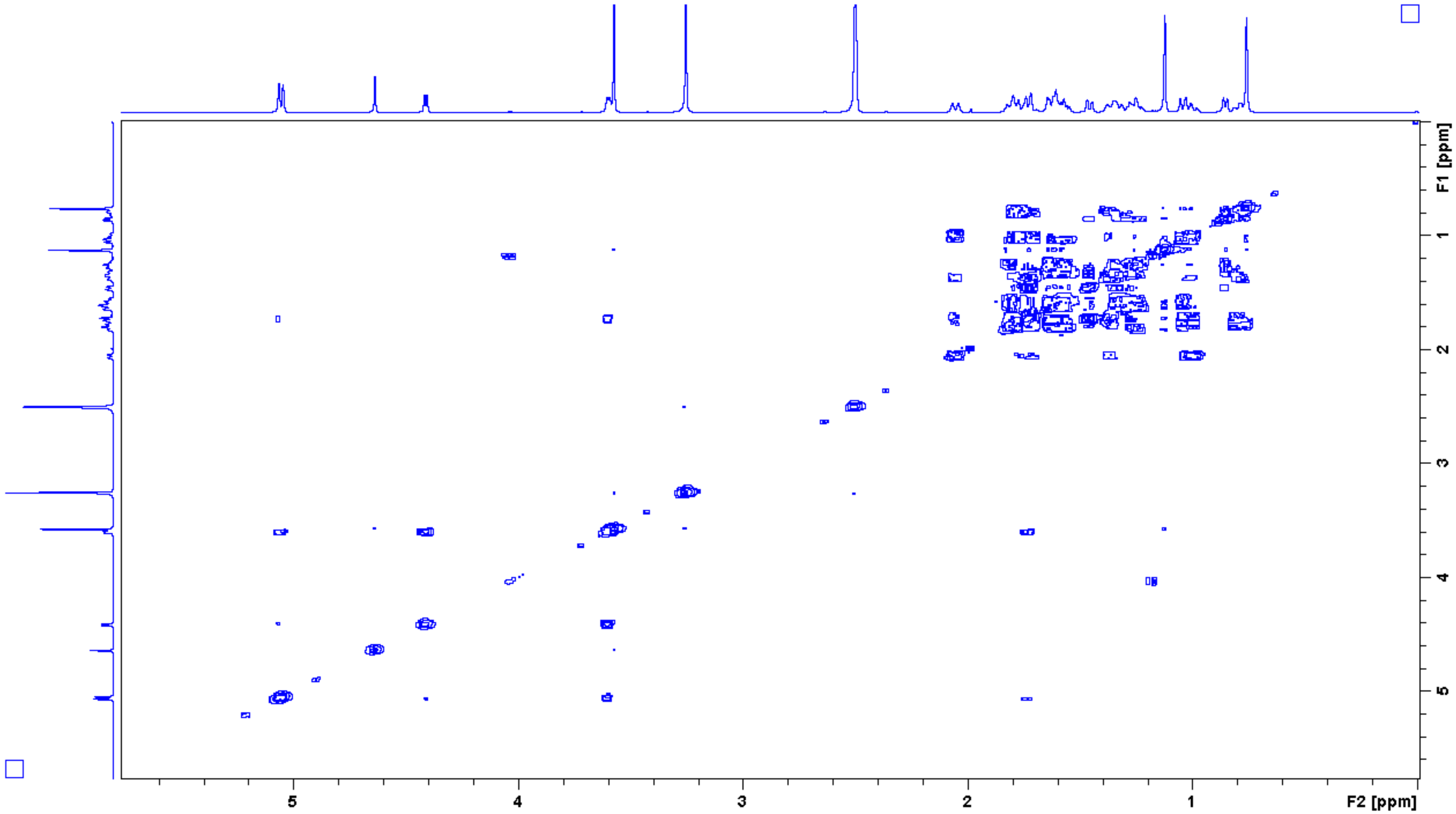

NOESY of compound (4*R*,6a*R*,7*R*,9*S*,11b*S*)-methyl 7,9-dihydroxy-4,11b-dimethyl-8-methylenetetradecahydro-6a,9-methanocyclohepta[a]naphthalene-4-carboxylate **(3)**

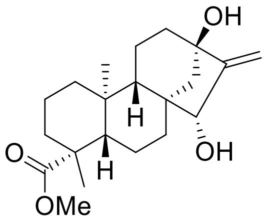

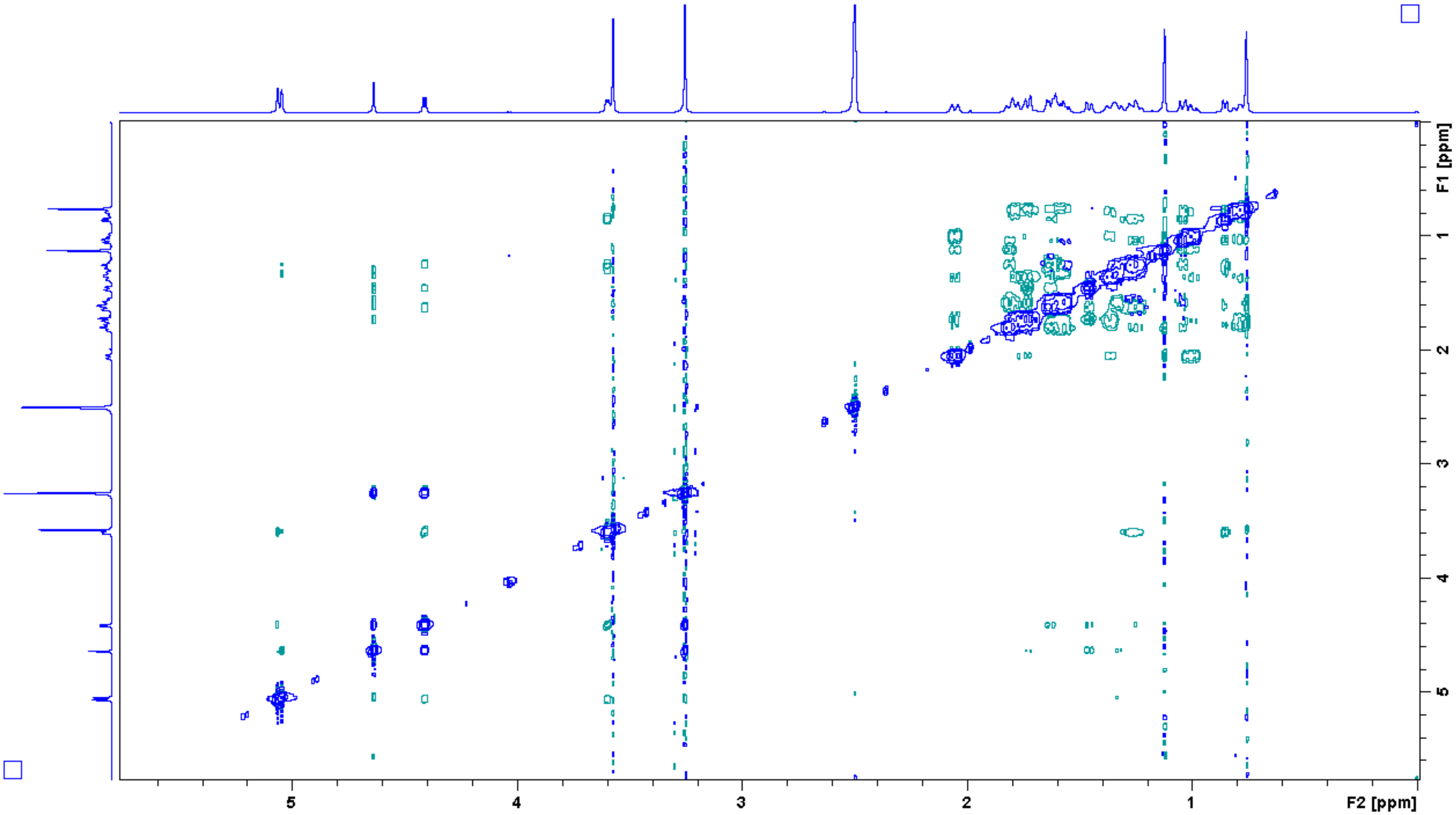

HSQC of compound (4*R*,6a*R*,7*R*,9*S*,11b*S*)-methyl 7,9-dihydroxy-4,11b-dimethyl-8-methylenetetradecahydro-6a,9-methanocyclohepta[a]naphthalene-4-carboxylate **(3)**

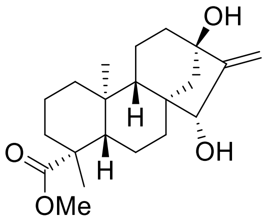

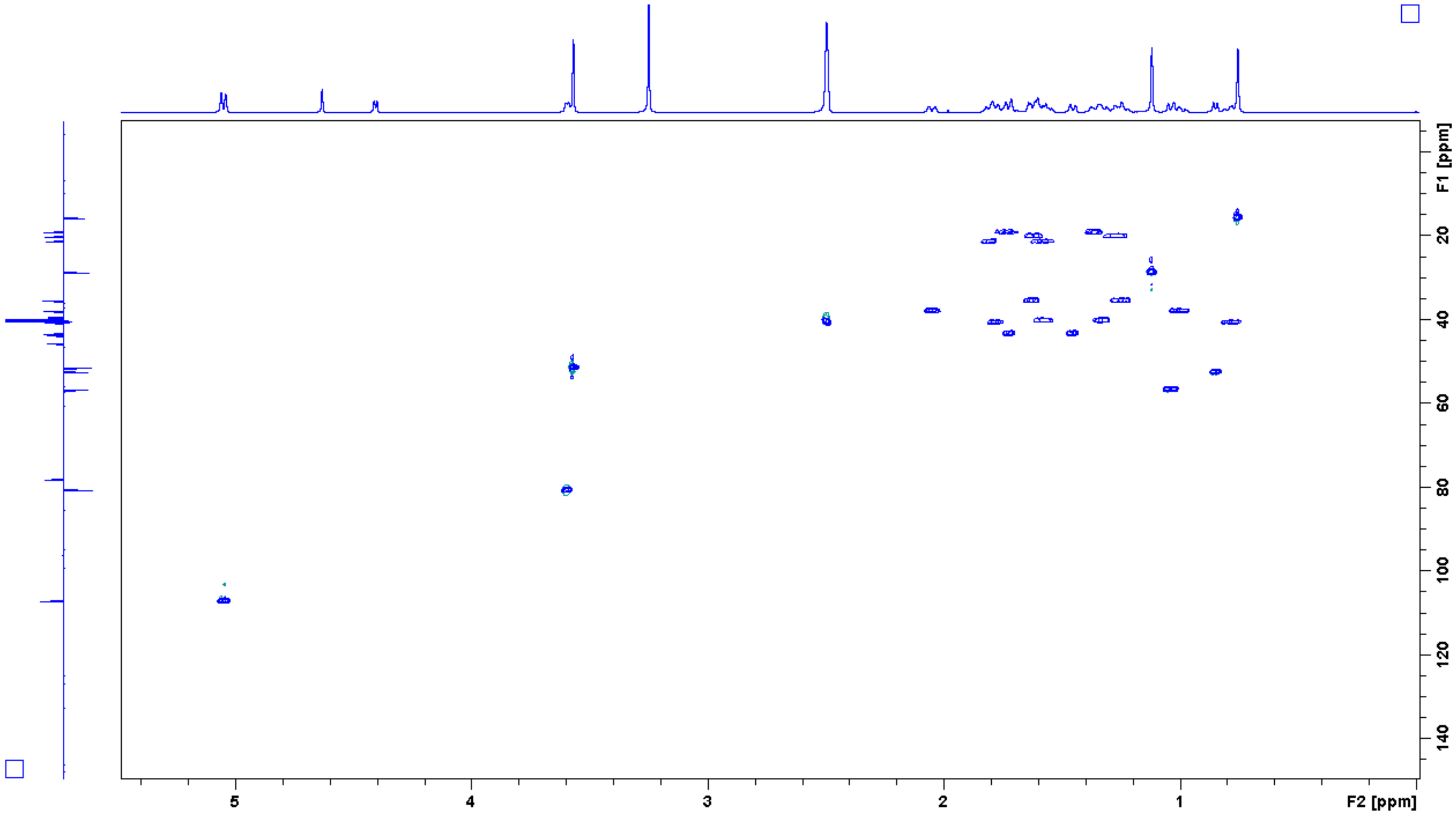

HMBC of compound (4*R*,6a*R*,7*R*,9*S*,11b*S*)-methyl 7,9-dihydroxy-4,11b-dimethyl-8-methylenetetradecahydro-6a,9-methanocyclohepta[a]naphthalene-4-carboxylate **(3)**

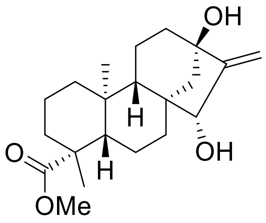

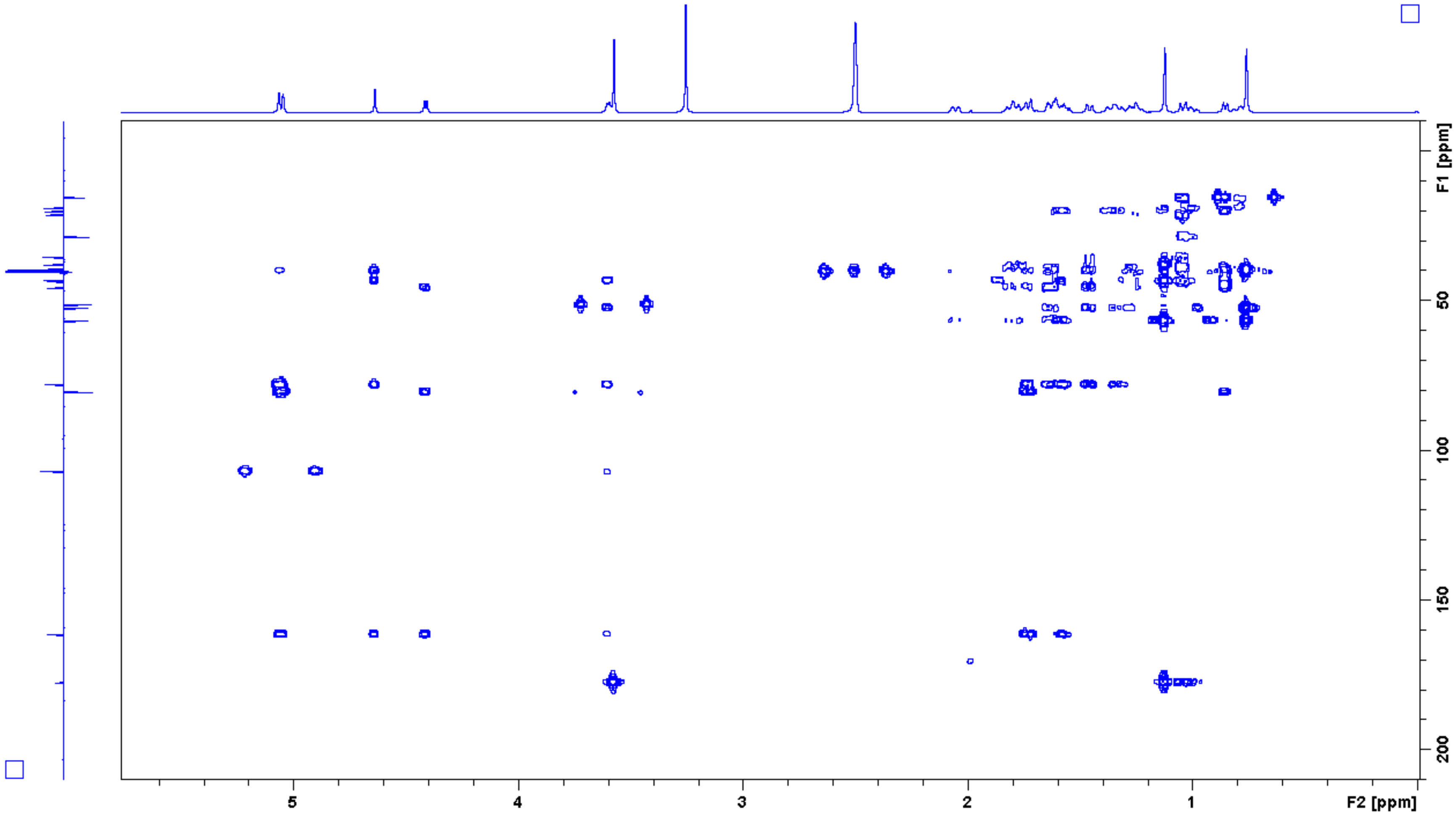

^{1}H-NMR of compound (2'*R*,4*R*,6a*R*,7*R*,9*S*,11b*S*)-methyl 7,9-dihydroxy-4,11b-dimethyldodecahydro-1H-spiro[6a,9-methanocyclohepta[a]naphthalene-8,2'-oxirane]-4-carboxylate **(4b)**

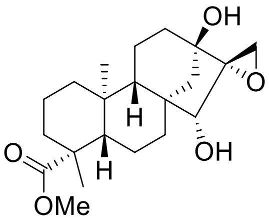

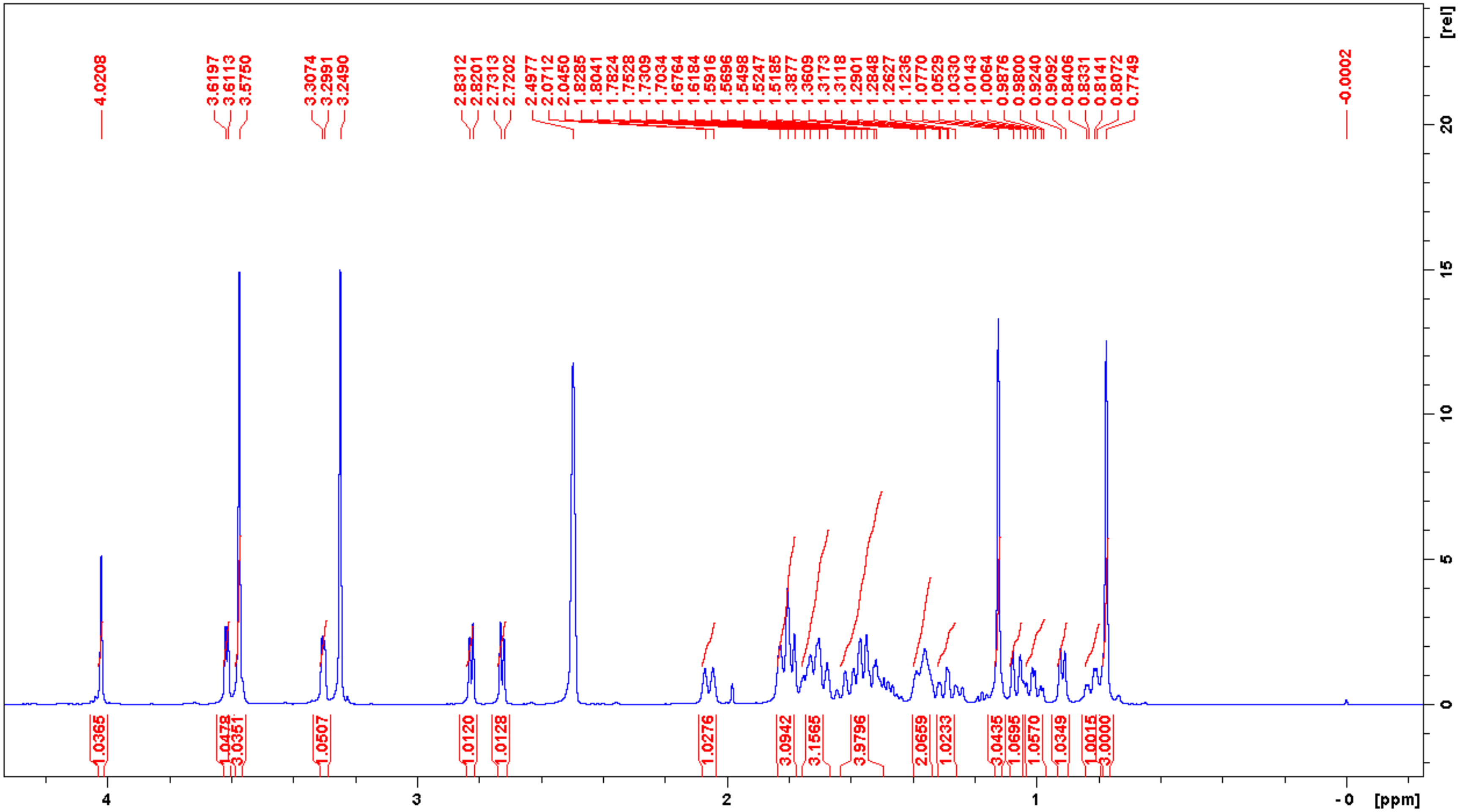

^{13}C-NMR of compound (2'*R*,4*R*,6a*R*,7*R*,9*S*,11b*S*)-methyl 7,9-dihydroxy-4,11b-dimethyldodecahydro-1H-spiro[6a,9-methanocyclohepta[a]naphthalene-8,2'-oxirane]-4-carboxylate **(4b)**

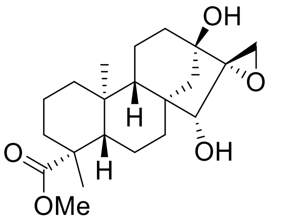

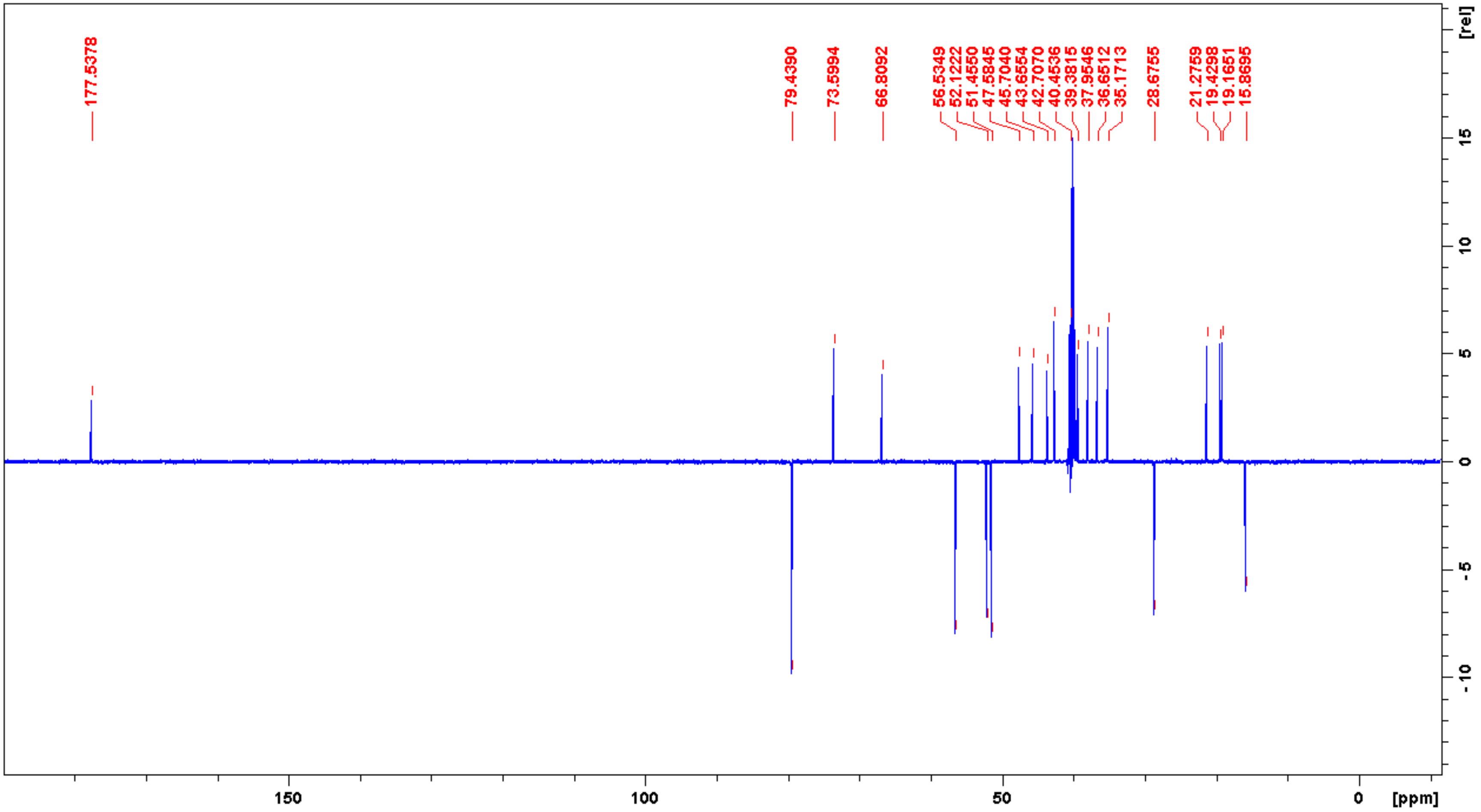

COSY of compound (2'*R*,4*R*,6a*R*,7*R*,9*S*,11b*S*)-methyl 7,9-dihydroxy-4,11b-dimethyldodecahydro-1H-spiro[6a,9-methanocyclohepta[a]naphthalene-8,2'-oxirane]-4-carboxylate **(4b)**

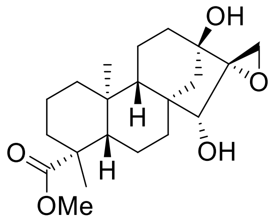

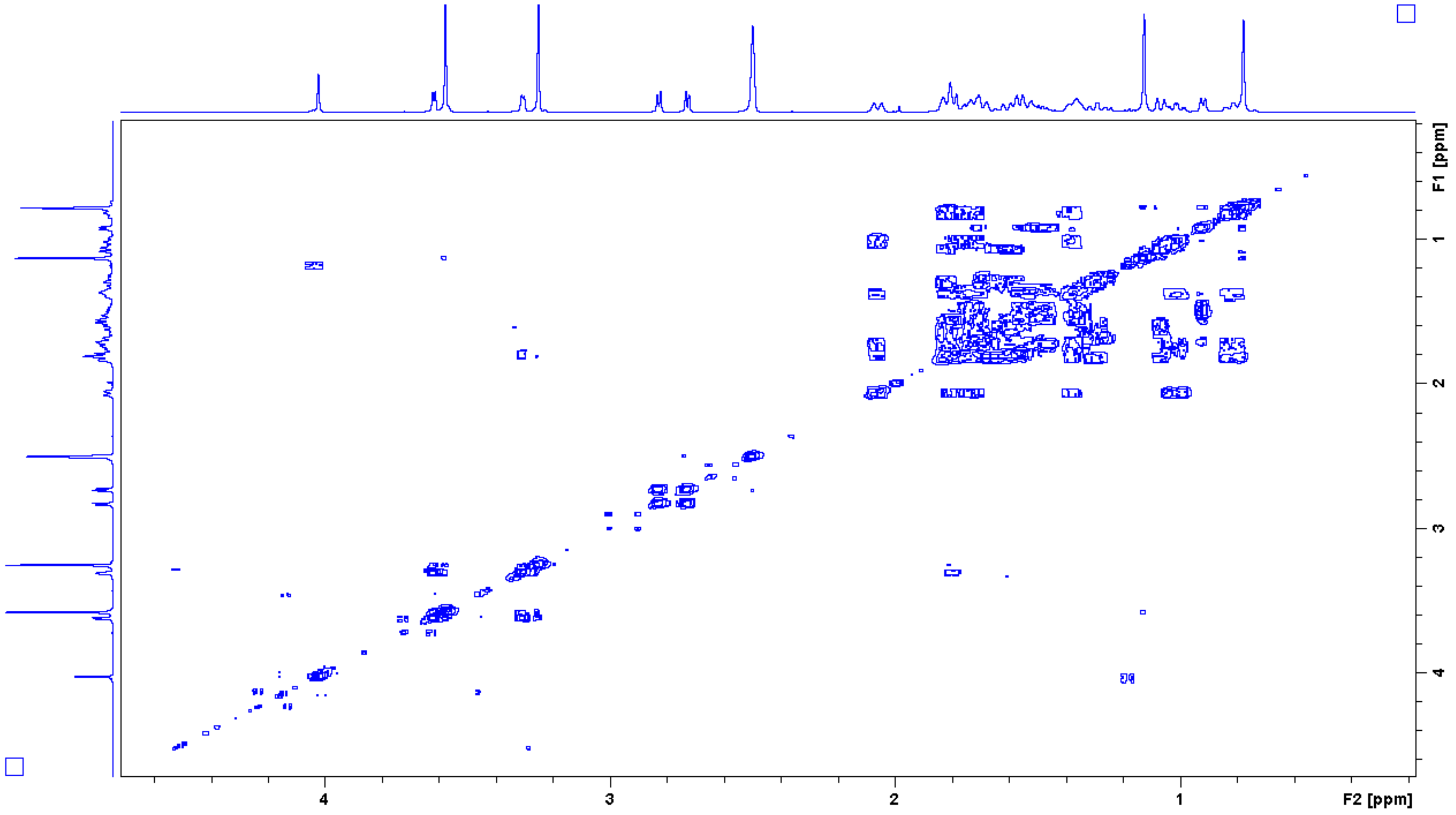

NOESY of compound (2'*R*,4*R*,6a*R*,7*R*,9*S*,11b*S*)-methyl 7,9-dihydroxy-4,11b-dimethyldodecahydro-1H-spiro[6a,9-methanocyclohepta[a]naphthalene-8,2'-oxirane]-4-carboxylate **(4b)**

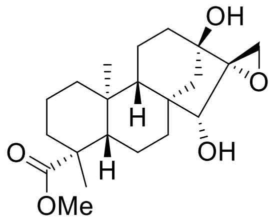

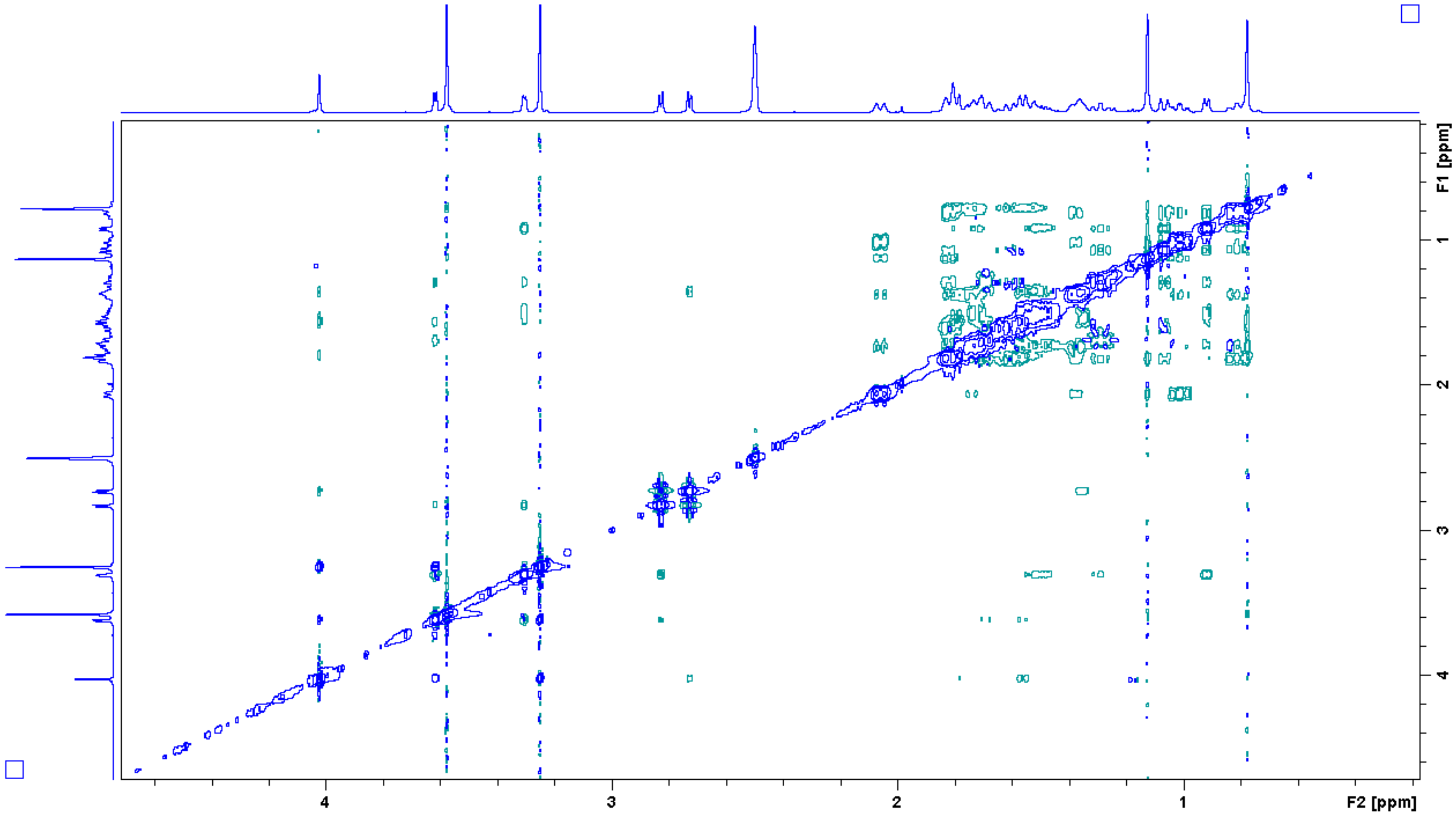

HSQC of compound (2'*R*,4*R*,6a*R*,7*R*,9*S*,11b*S*)-methyl 7,9-dihydroxy-4,11b-dimethyldodecahydro-1H-spiro[6a,9-methanocyclohepta[a]naphthalene-8,2'-oxirane]-4-carboxylate **(4b)**

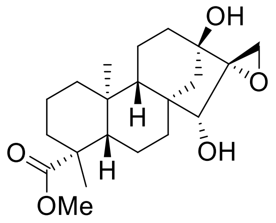

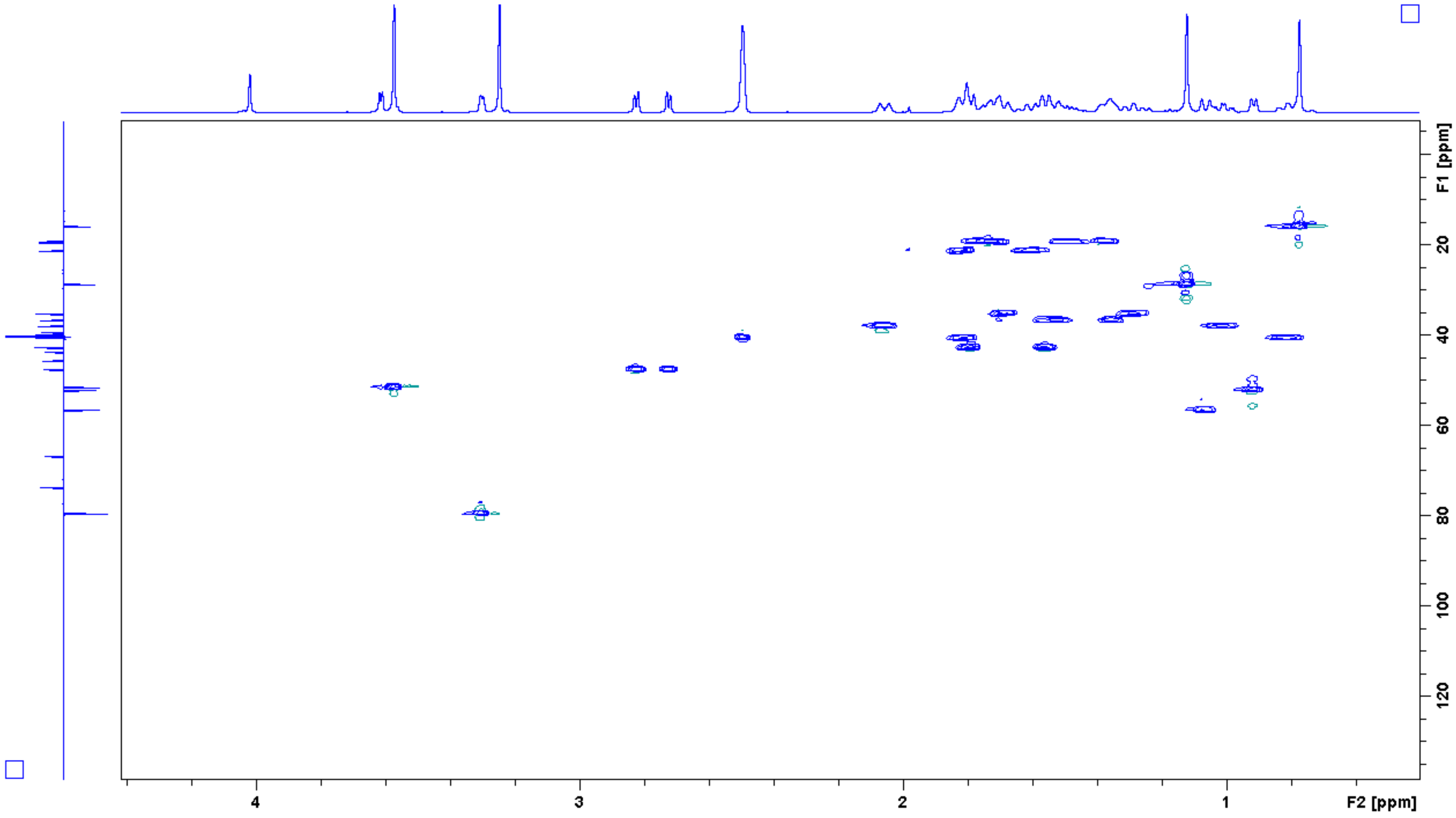

HMBC of compound (2'*R*,4*R*,6a*R*,7*R*,9*S*,11b*S*)-methyl 7,9-dihydroxy-4,11b-dimethyldodecahydro-1H-spiro[6a,9-methanocyclohepta[a]naphthalene-8,2'-oxirane]-4-carboxylate **(4b)**

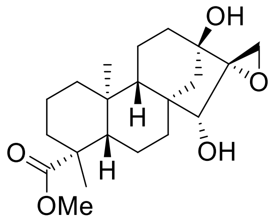

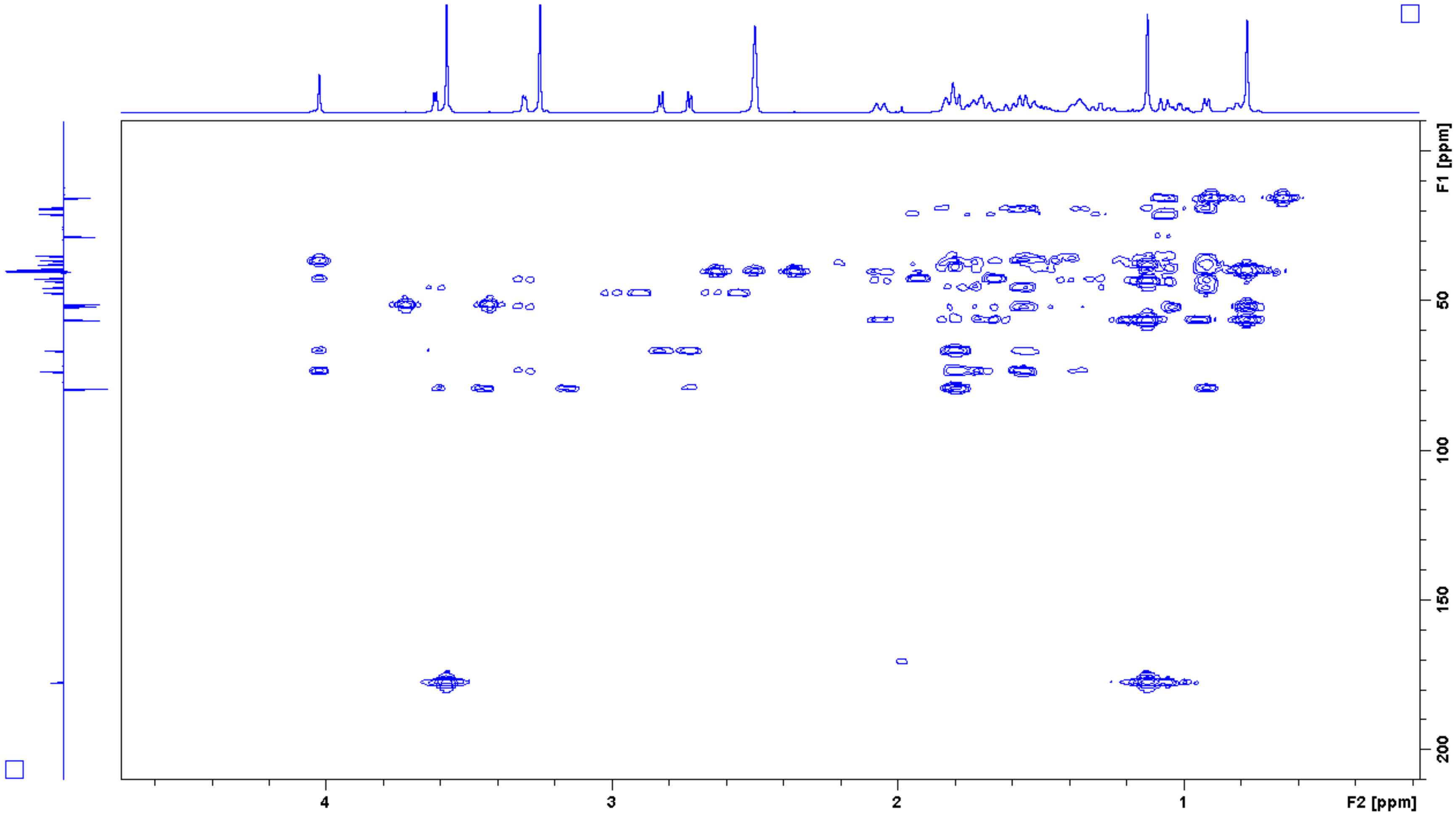

^{1}H-NMR of compound (4*R*,6a*R*,7*R*,8*R*,9*S*,11b*S*)-methyl 8-((benzylamino)methyl)-7,8,9-trihydroxy-4,11b-dimethyltetradecahydro-6a,9-methanocyclohepta[a]naphthalene-4-carboxylate **(5)**

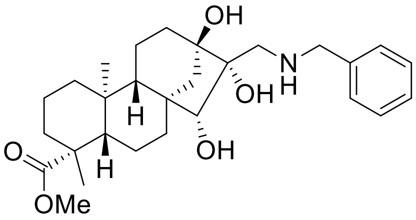

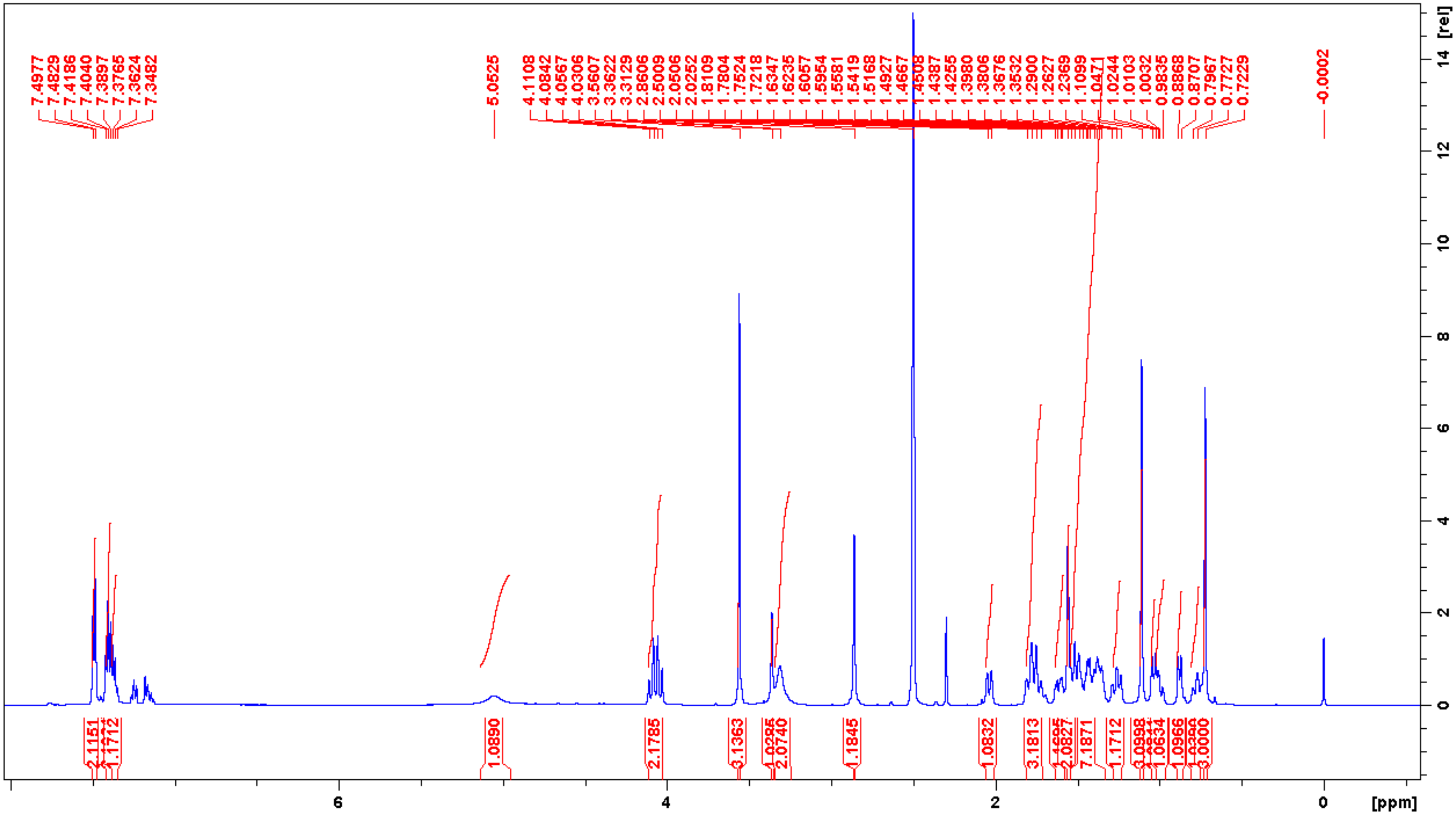

^{13}C-NMR of compound (4*R*,6a*R*,7*R*,8*R*,9*S*,11b*S*)-methyl 8-((benzylamino)methyl)-7,8,9-trihydroxy-4,11b-dimethyltetradecahydro-6a,9-methanocyclohepta[a]naphthalene-4-carboxylate **(5)**

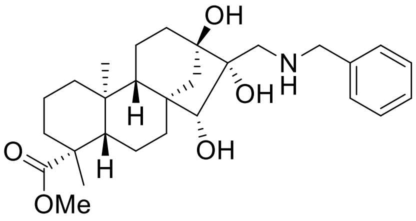

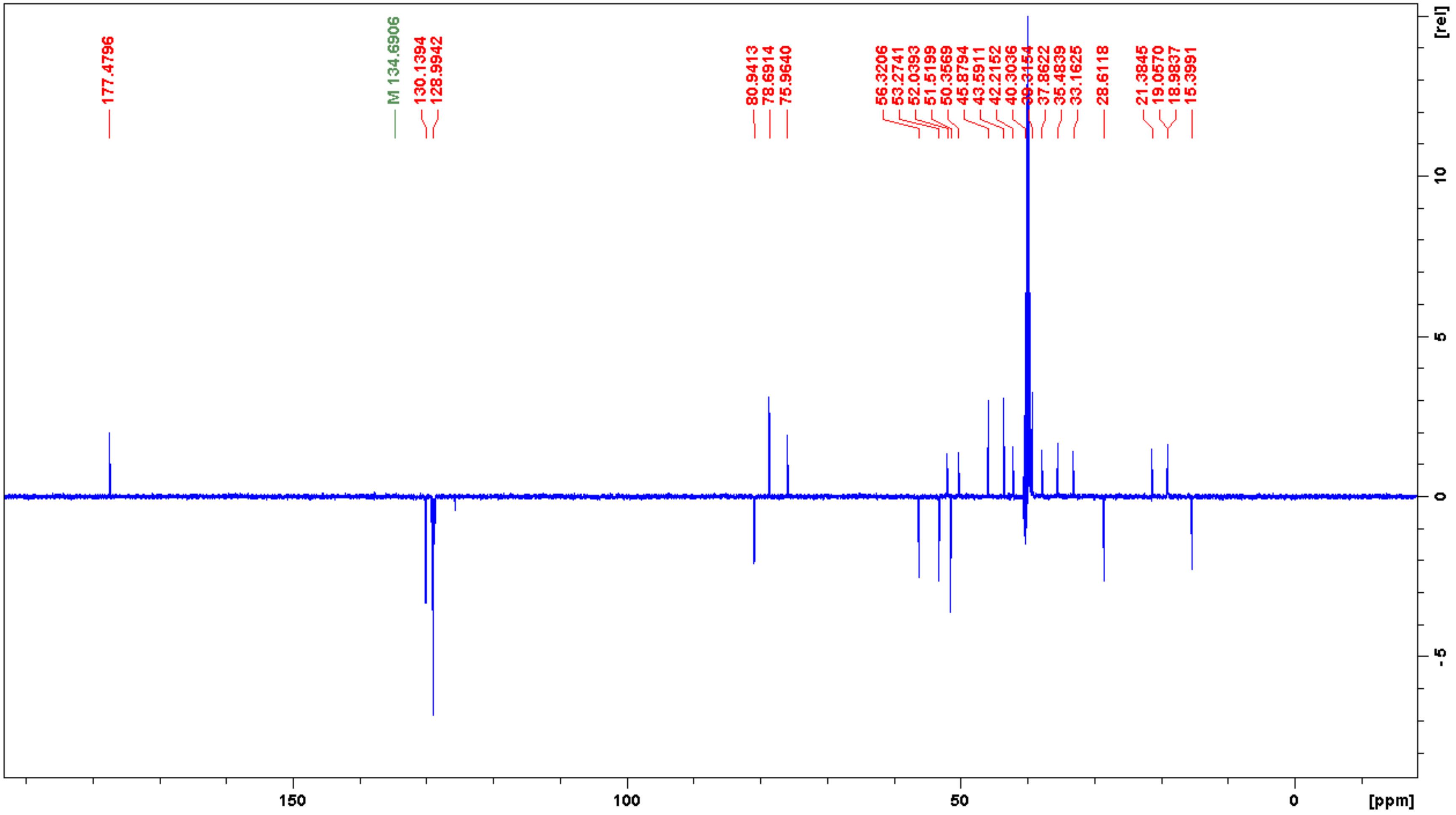

COSY of compound (4*R*,6a*R*,7*R*,8*R*,9*S*,11b*S*)-methyl 8-((benzylamino)methyl)-7,8,9-trihydroxy-4,11b-dimethyltetradecahydro-6a,9-methanocyclohepta[a]naphthalene-4-carboxylate **(5)**

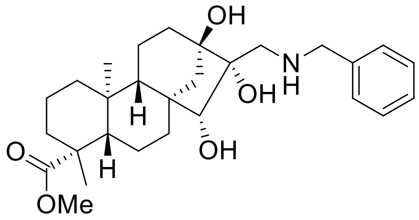

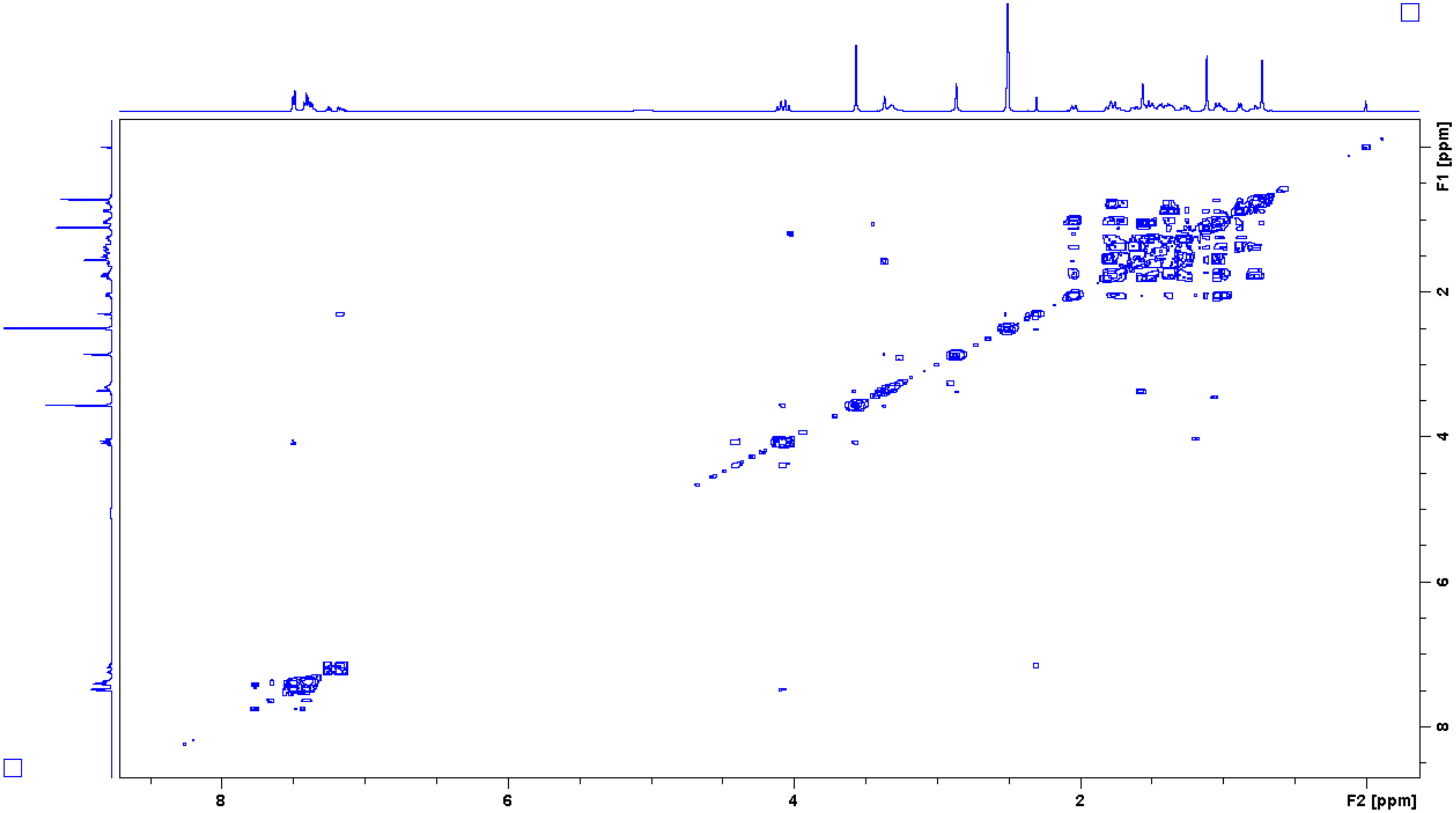

NOESY of compound (4*R*,6a*R*,7*R*,8*R*,9*S*,11b*S*)-methyl 8-((benzylamino)methyl)-7,8,9-trihydroxy-4,11b-dimethyltetradecahydro-6a,9-methanocyclohepta[a]naphthalene-4-carboxylate **(5)**

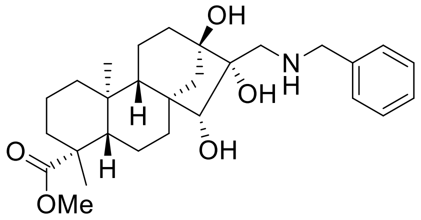

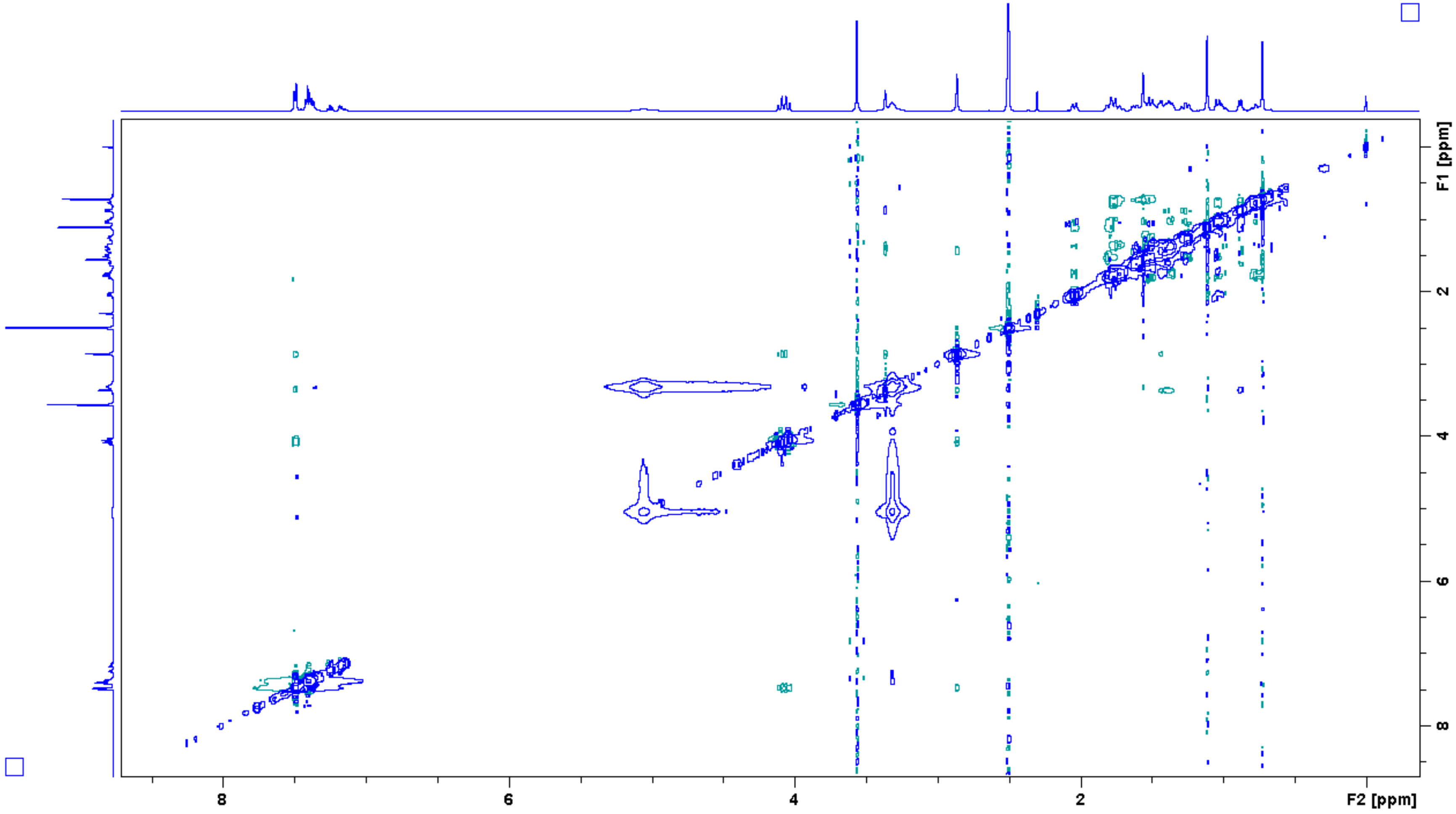

HSQC of compound (4*R*,6a*R*,7*R*,8*R*,9*S*,11b*S*)-methyl 8-((benzylamino)methyl)-7,8,9-trihydroxy-4,11b-dimethyltetradecahydro-6a,9-methanocyclohepta[a]naphthalene-4-carboxylate **(5)**

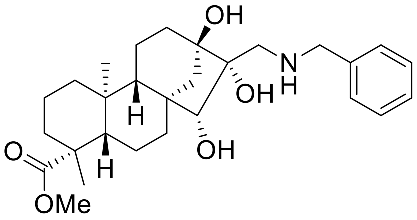

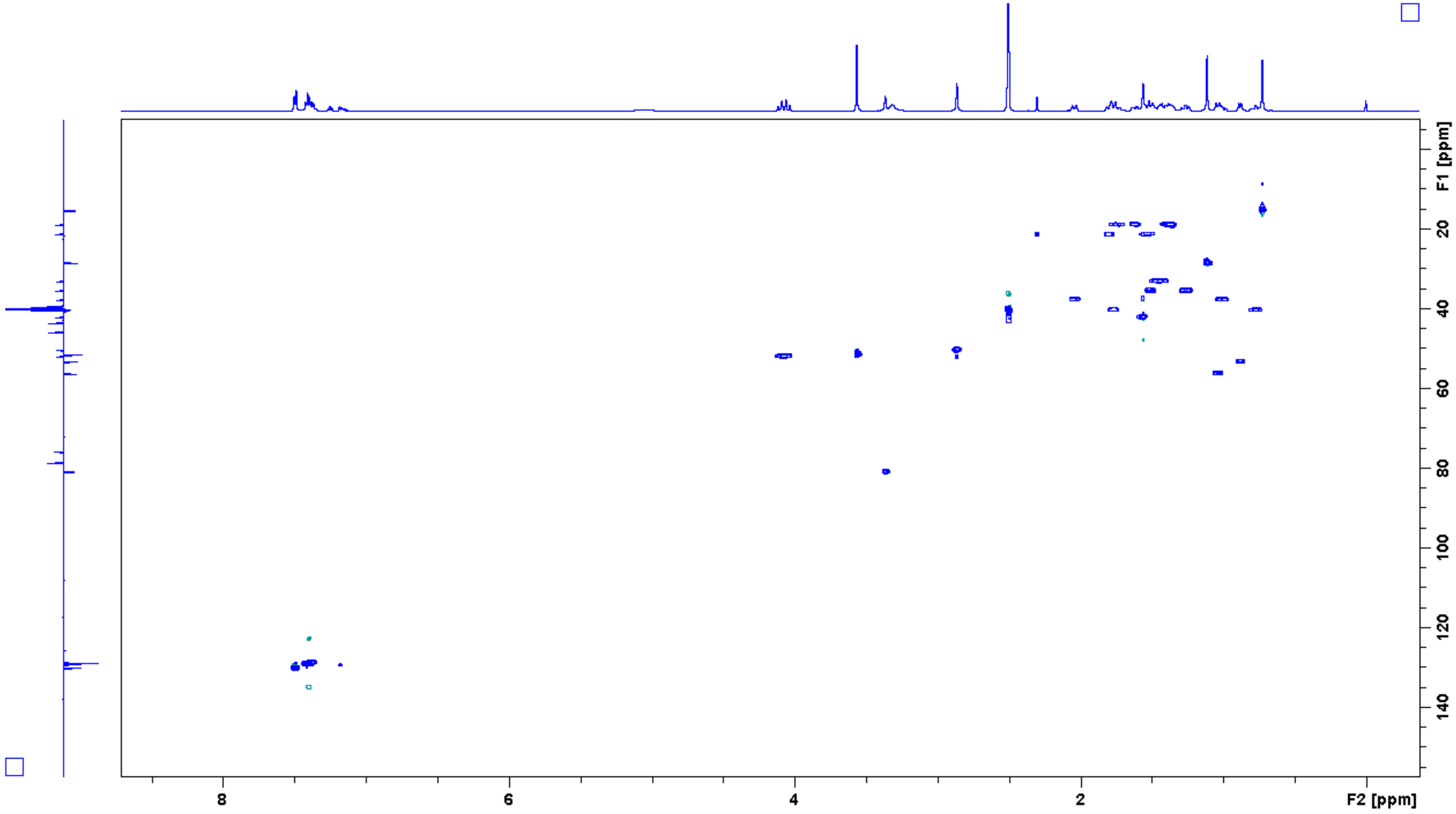

HMBC of compound (4*R*,6a*R*,7*R*,8*R*,9*S*,11b*S*)-methyl 8-((benzylamino)methyl)-7,8,9-trihydroxy-4,11b-dimethyltetradecahydro-6a,9-methanocyclohepta[a]naphthalene-4-carboxylate **(5)**

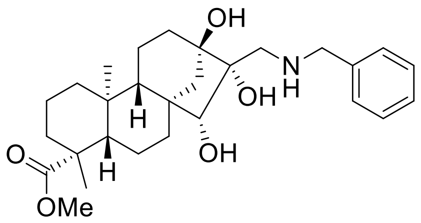

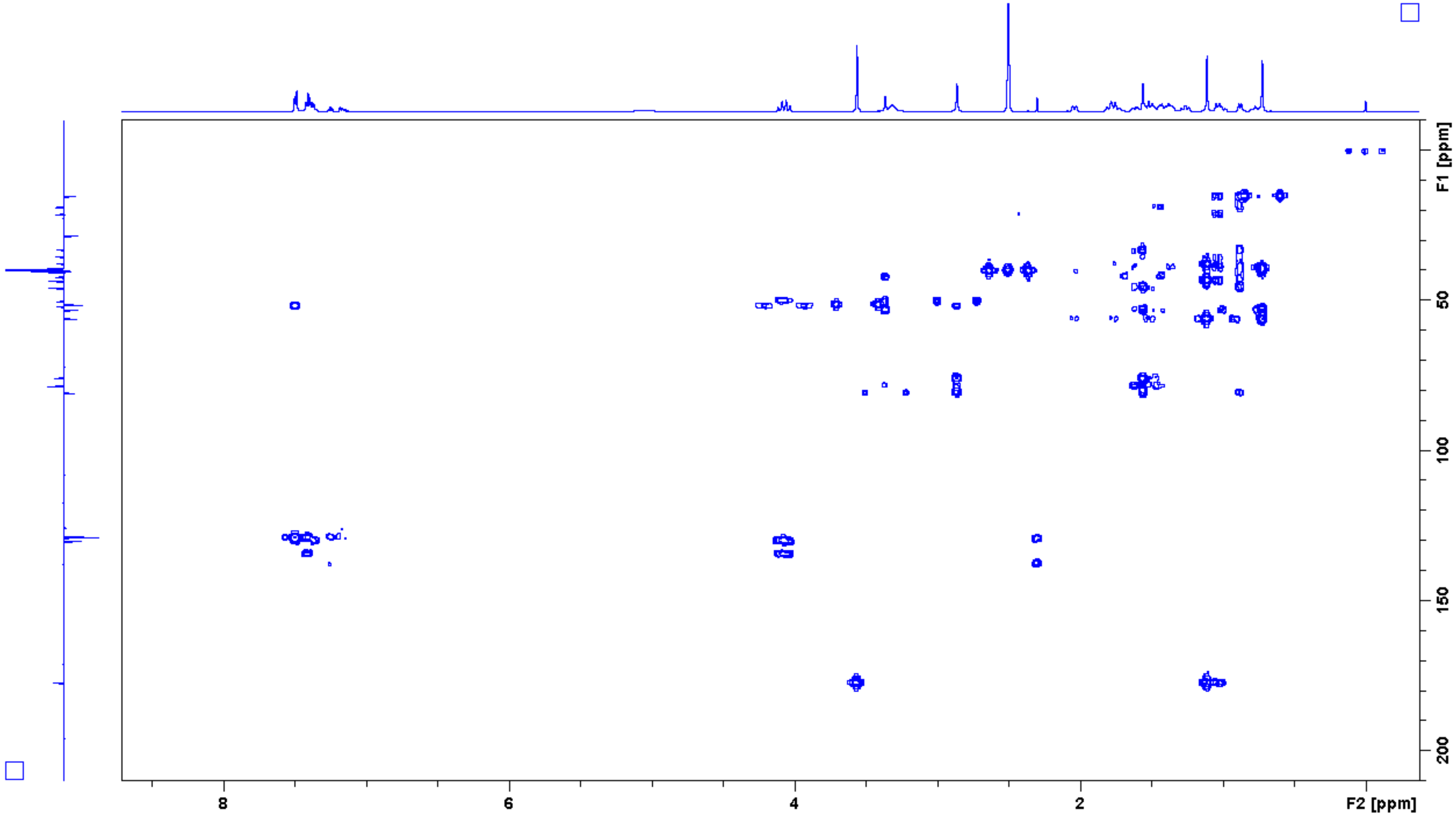

^{1}H-NMR of compound (4*R*,6a*R*,7*R*,8*R*,9*S*,11b*S*)-methyl 8-((benzyl(methyl)amino)methyl)-7,8,9-trihydroxy-4,11b-dimethyltetradecahydro-6a,9-methanocyclohepta[a]naphthalene-4-carboxylate **(6)**

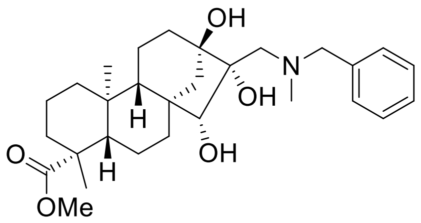

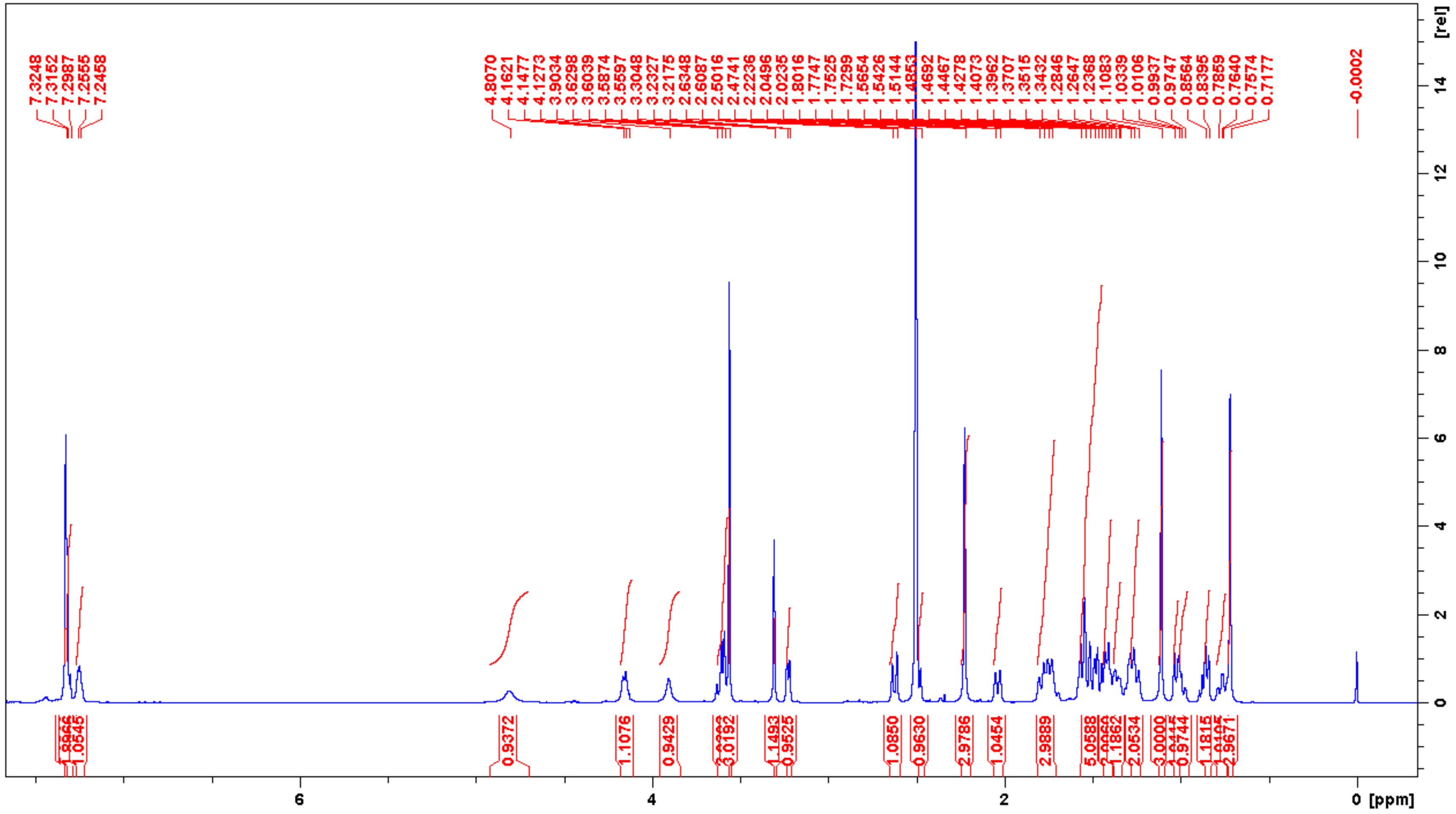

^{13}C-NMR of compound (4*R*,6a*R*,7*R*,8*R*,9*S*,11b*S*)-methyl 8-((benzyl(methyl)amino)methyl)-7,8,9-trihydroxy-4,11b-dimethyltetradecahydro-6a,9-methanocyclohepta[a]naphthalene-4-carboxylate **(6)**

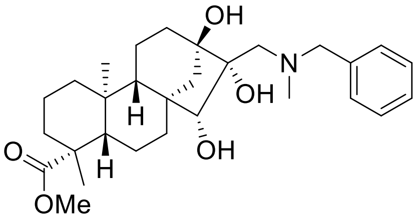

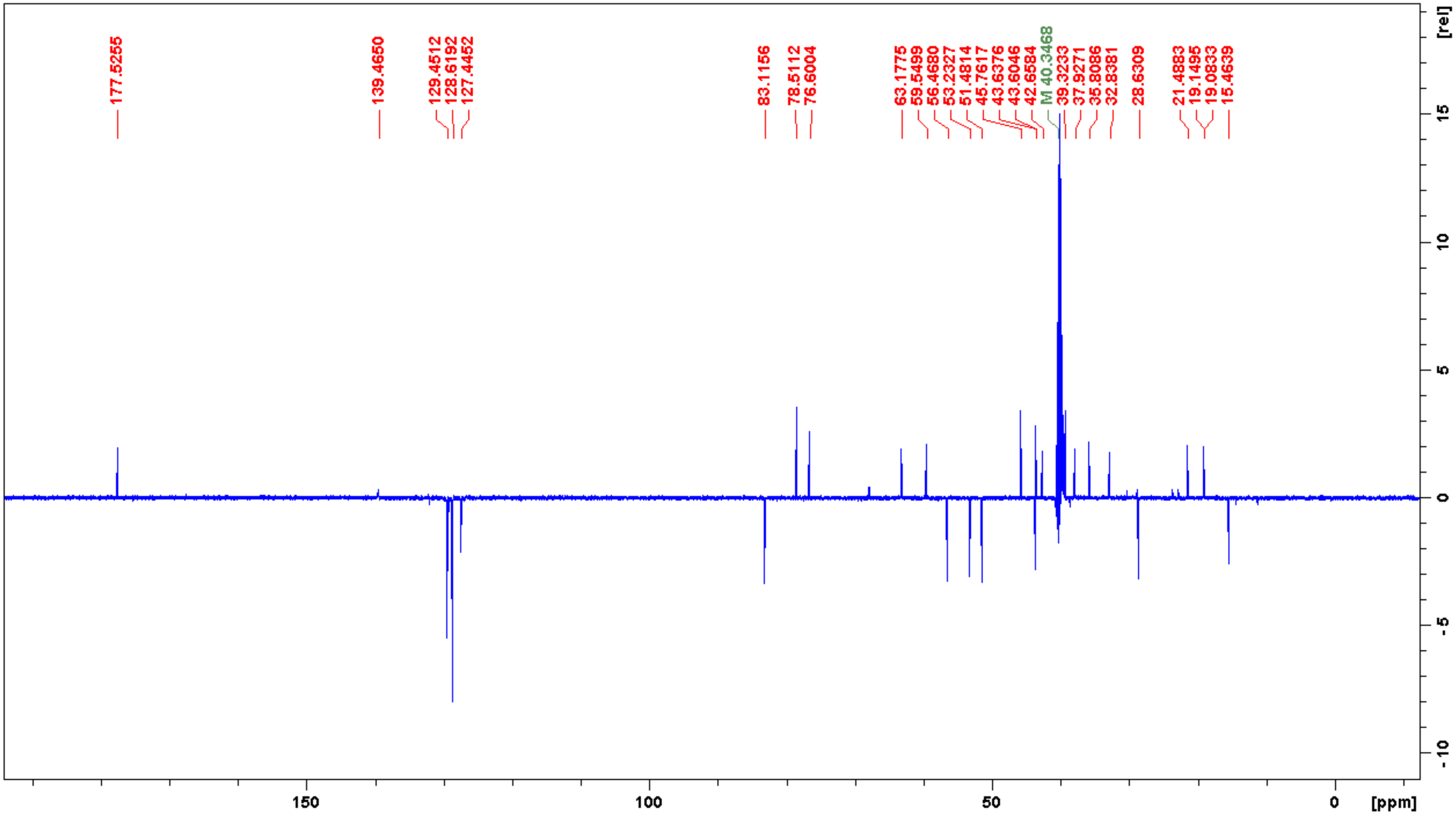

COSY of compound (4*R*,6a*R*,7*R*,8*R*,9*S*,11b*S*)-methyl 8-((benzyl(methyl)amino)methyl)-7,8,9-trihydroxy-4,11b-dimethyltetradecahydro-6a,9-methanocyclohepta[a]naphthalene-4-carboxylate **(6)**

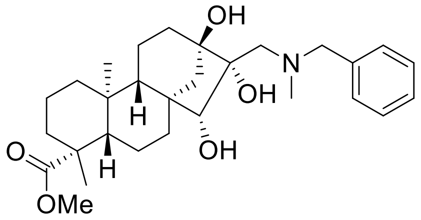

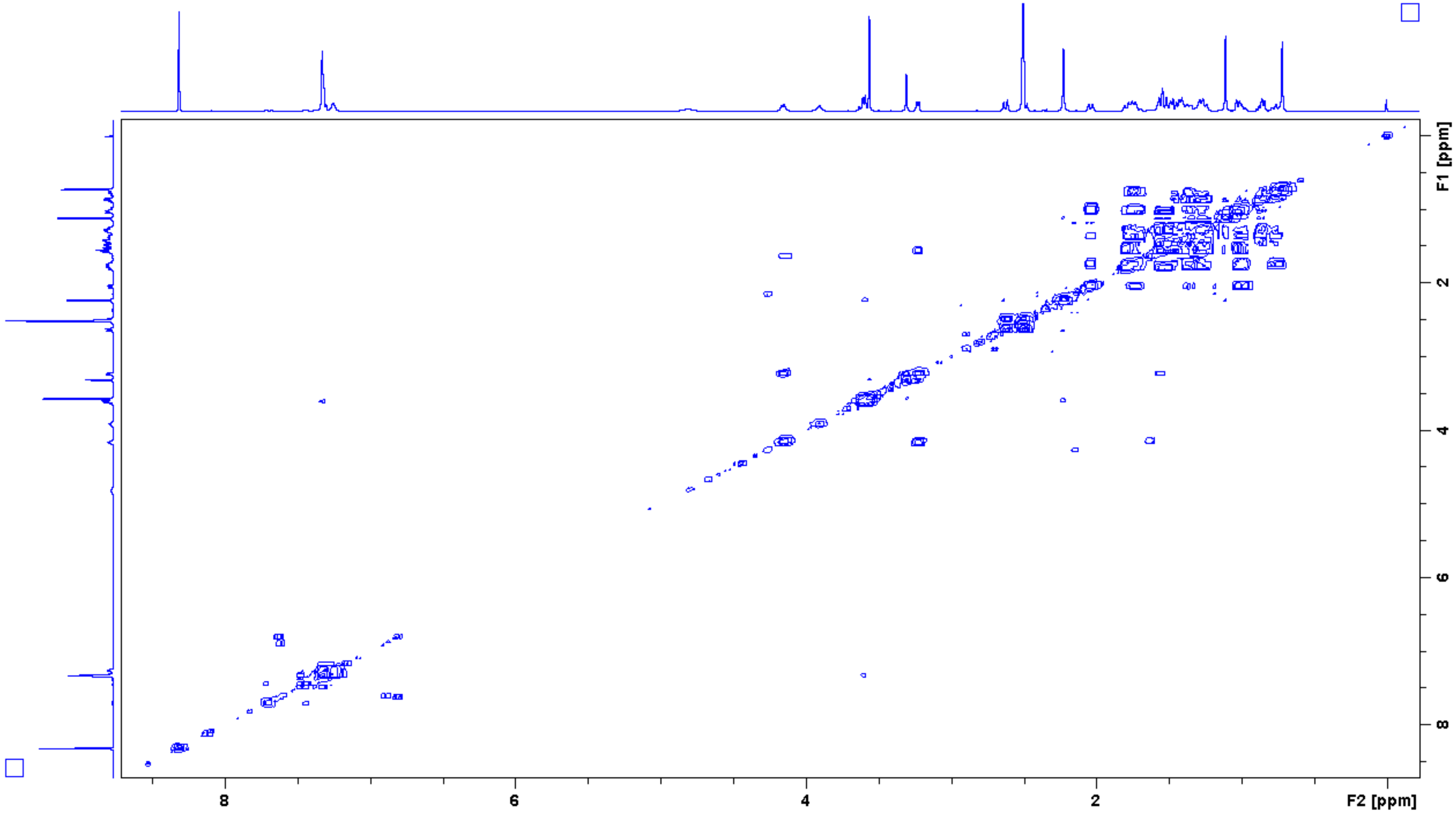

NOESY of compound (4*R*,6a*R*,7*R*,8*R*,9*S*,11b*S*)-methyl 8-((benzyl(methyl)amino)methyl)-7,8,9-trihydroxy-4,11b-dimethyltetradecahydro-6a,9-methanocyclohepta[a]naphthalene-4-carboxylate **(6)**

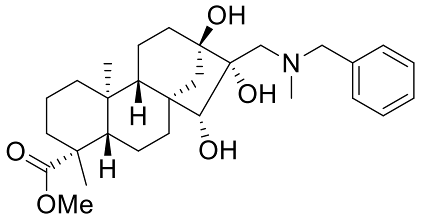

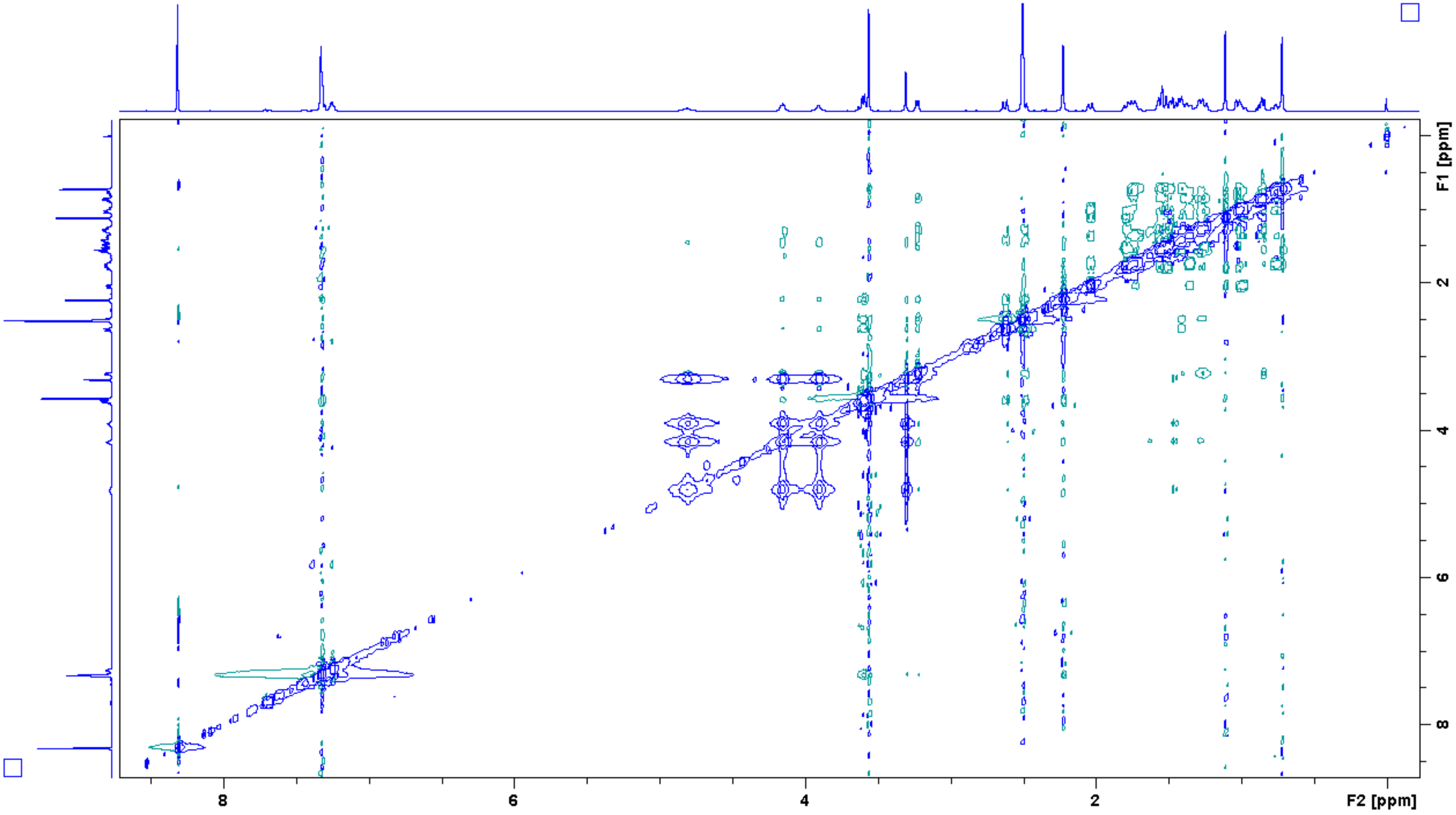

HSQC of compound (4*R*,6a*R*,7*R*,8*R*,9*S*,11b*S*)-methyl 8-((benzyl(methyl)amino)methyl)-7,8,9-trihydroxy-4,11b-dimethyltetradecahydro-6a,9-methanocyclohepta[a]naphthalene-4-carboxylate **(6)**

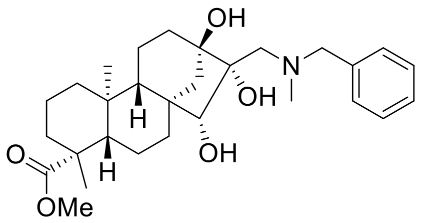

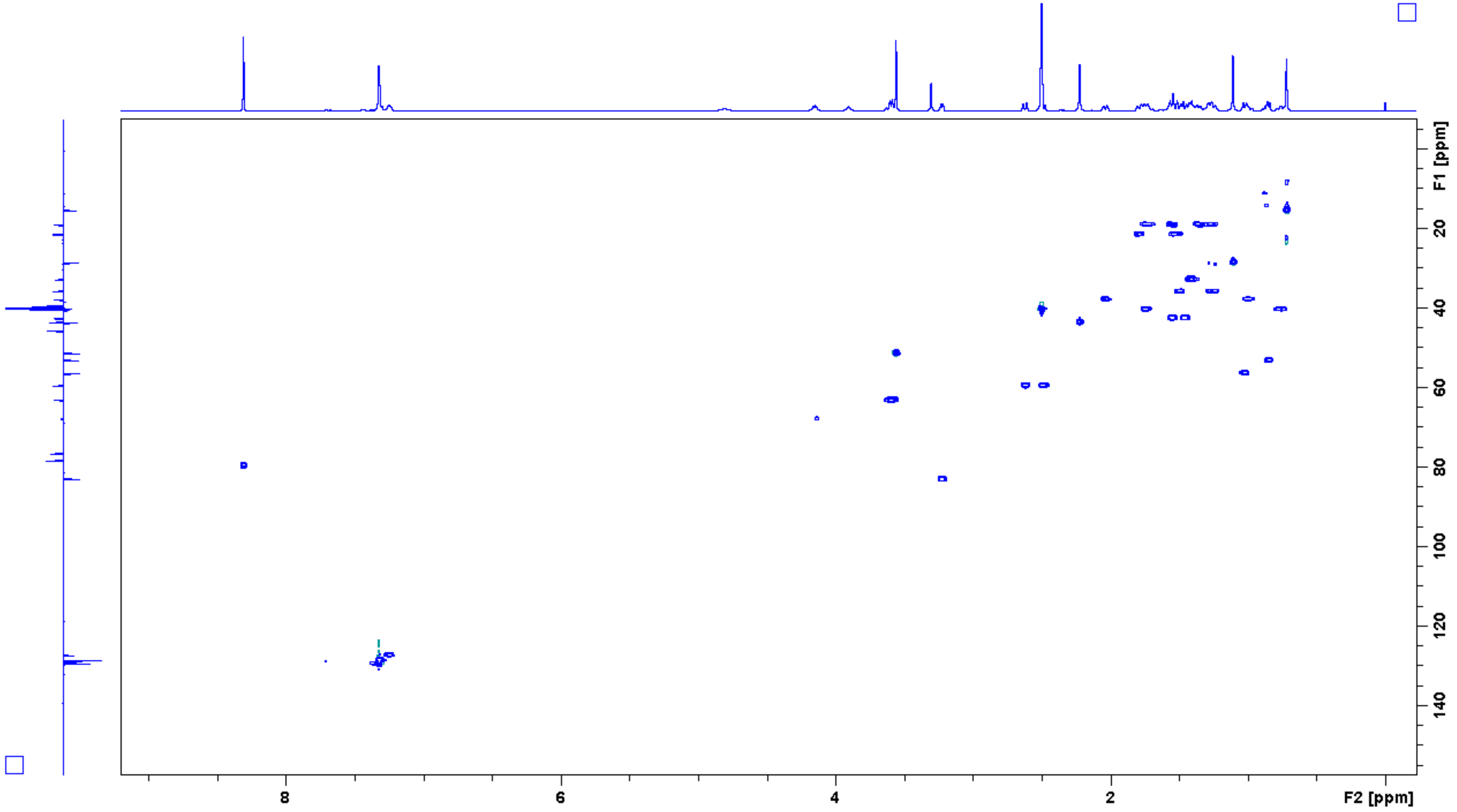

HMBC of compound (4*R*,6a*R*,7*R*,8*R*,9*S*,11b*S*)-methyl 8-((benzyl(methyl)amino)methyl)-7,8,9-trihydroxy-4,11b-dimethyltetradecahydro-6a,9-methanocyclohepta[a]naphthalene-4-carboxylate **(6)**

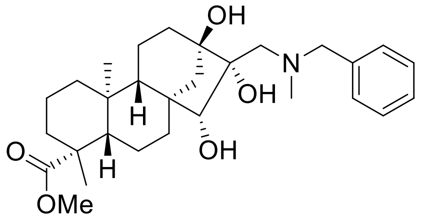

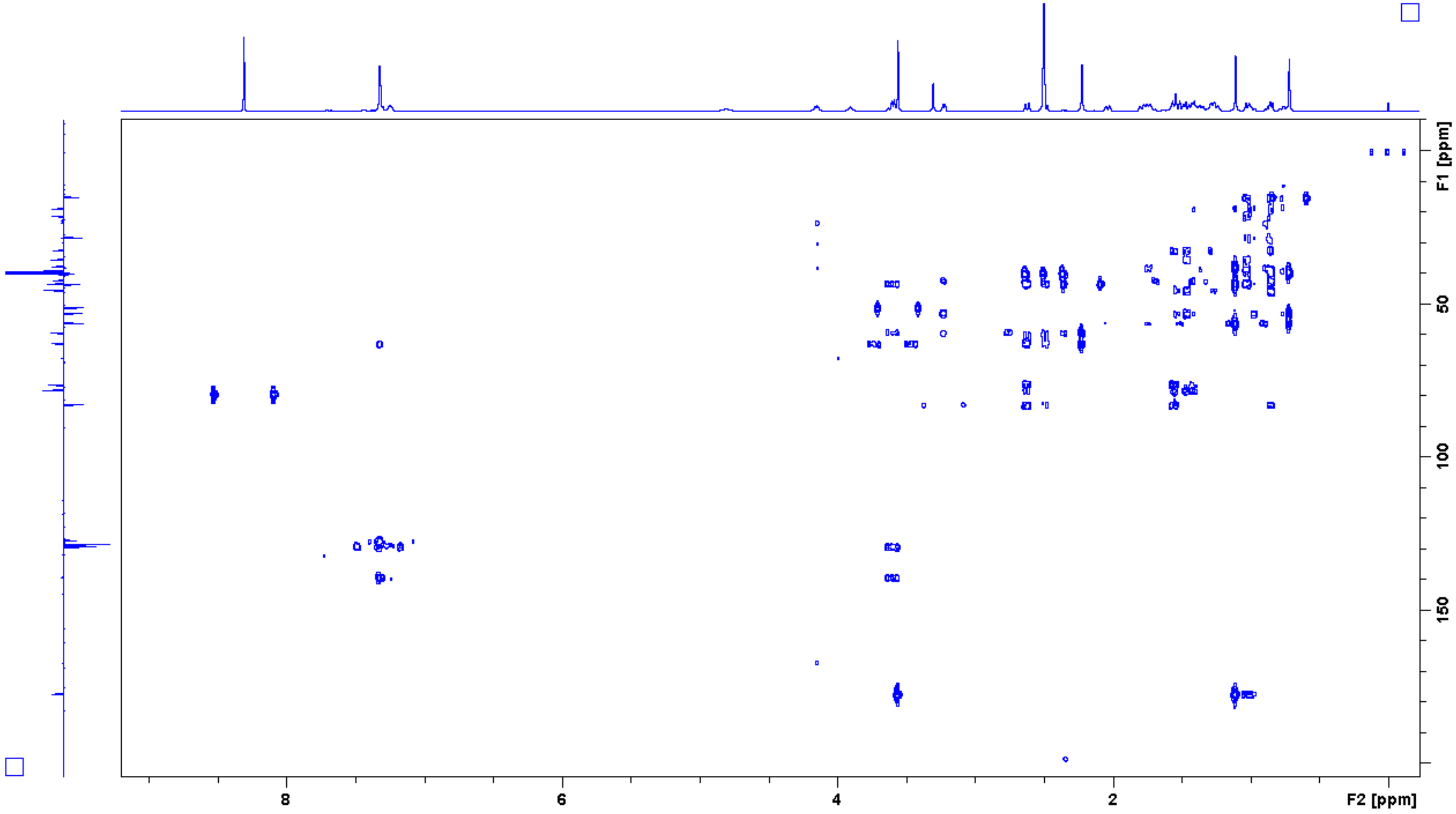

^{1}H-NMR of compound (4*R*,6a*R*,7*R*,8*R*,9*S*,11b*S*)-methyl 7,8,9-trihydroxy-4,11b-dimethyl-8-((((*R*)-1-phenylethyl)amino)methyl)tetradecahydro-6a,9-methanocyclohepta[a]naphthalene-4-carboxylate **(7)**

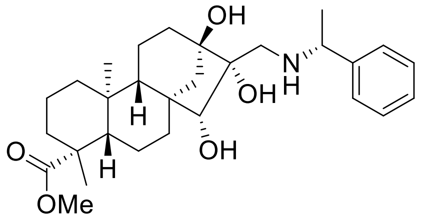

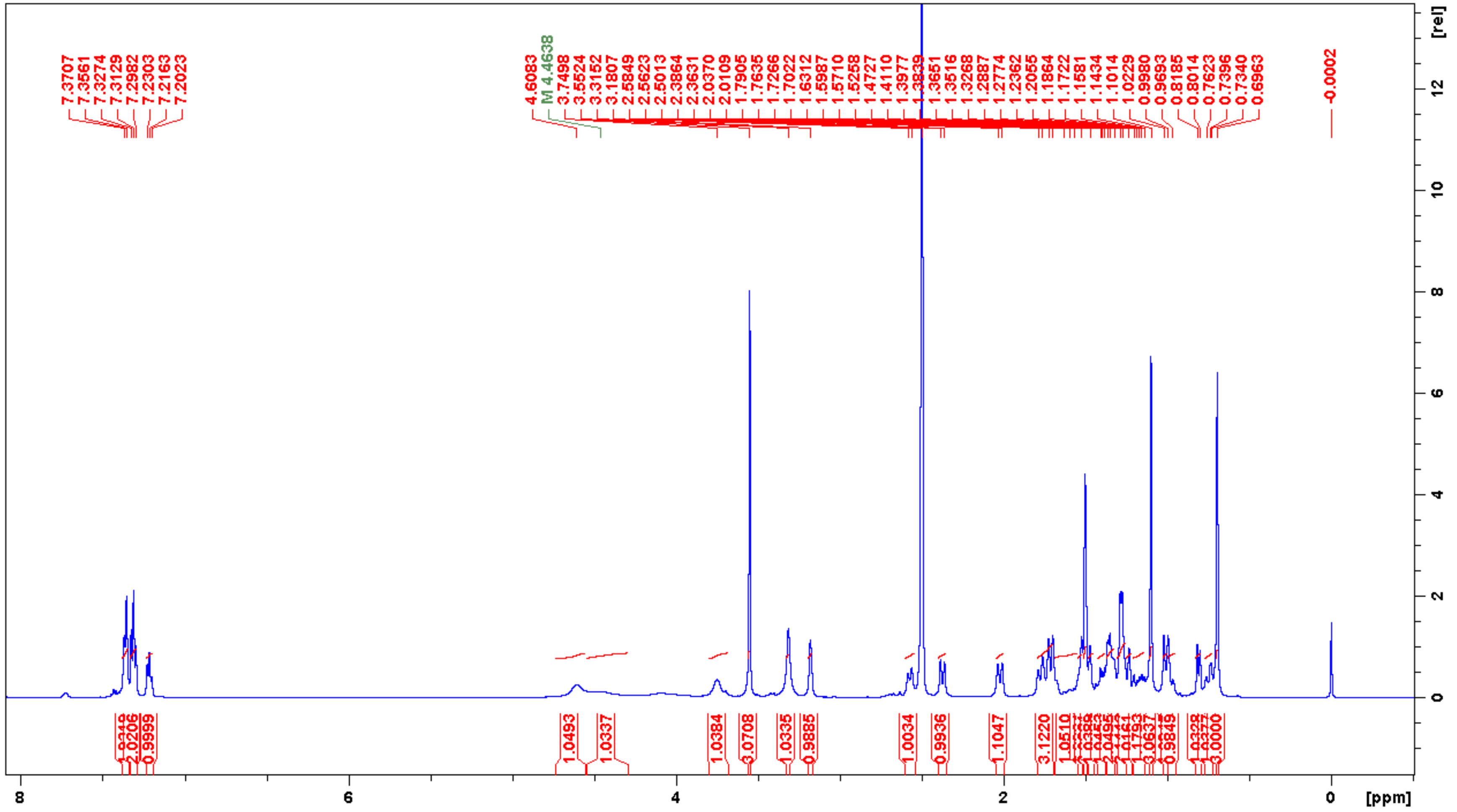

^{13}C-NMR of compound (4*R*,6a*R*,7*R*,8*R*,9*S*,11b*S*)-methyl 7,8,9-trihydroxy-4,11b-dimethyl-8-((((*R*)-1-phenylethyl)amino)methyl)tetradecahydro-6a,9-methanocyclohepta[a]naphthalene-4-carboxylate **(7)**

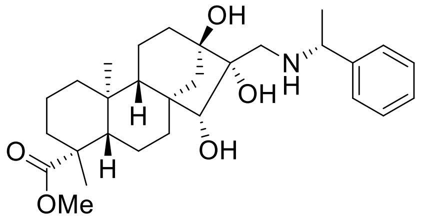

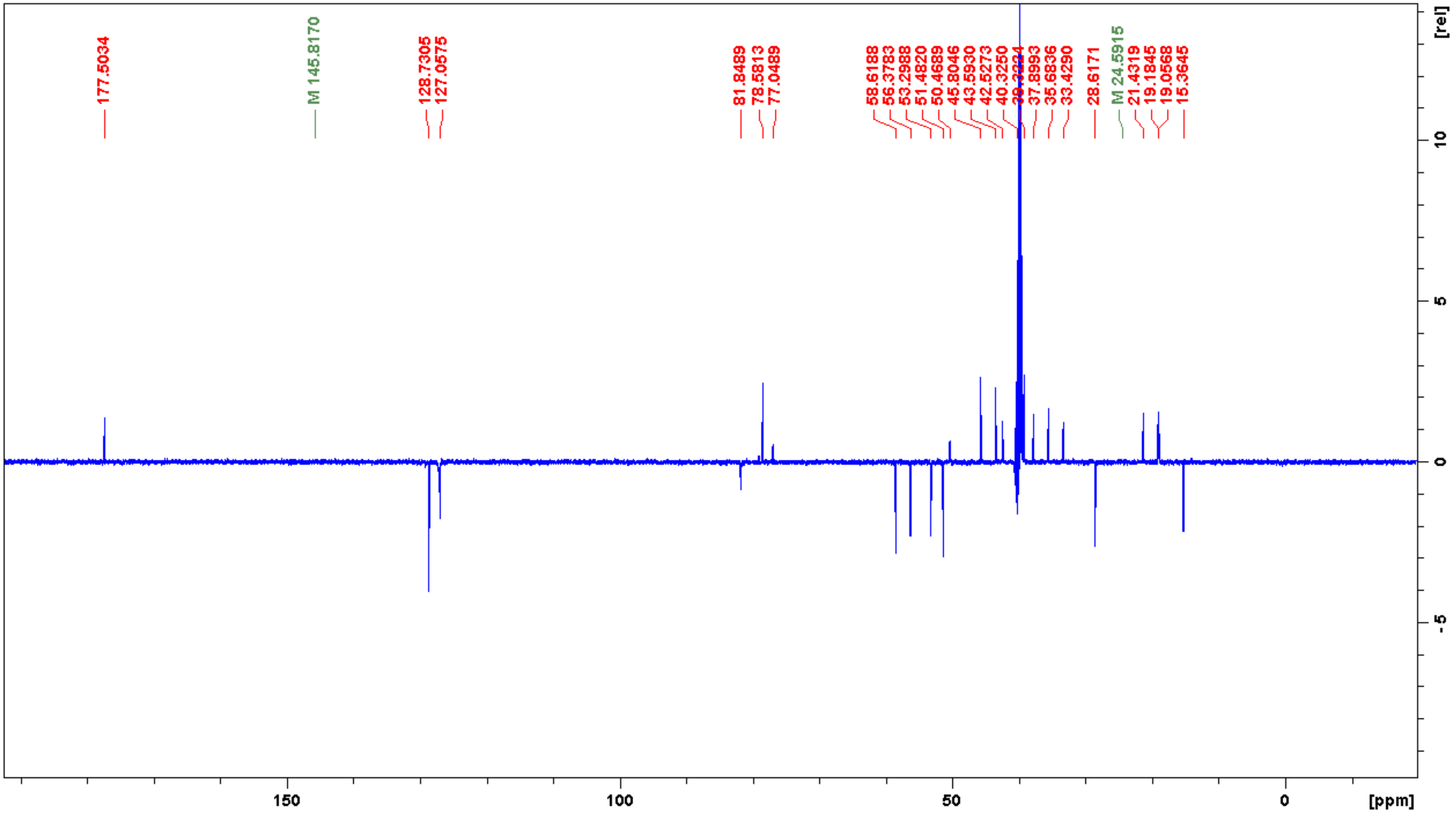

COSY of compound(4*R*,6a*R*,7*R*,8*R*,9*S*,11b*S*)-methyl 7,8,9-trihydroxy-4,11b-dimethyl-8-((((*R*)-1-phenylethyl)amino)methyl)tetradecahydro-6a,9-methanocyclohepta[a]naphthalene-4-carboxylate **(7)**

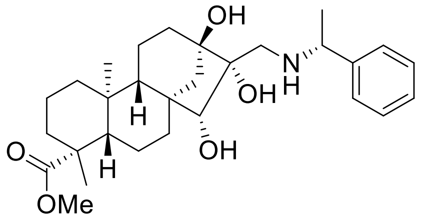

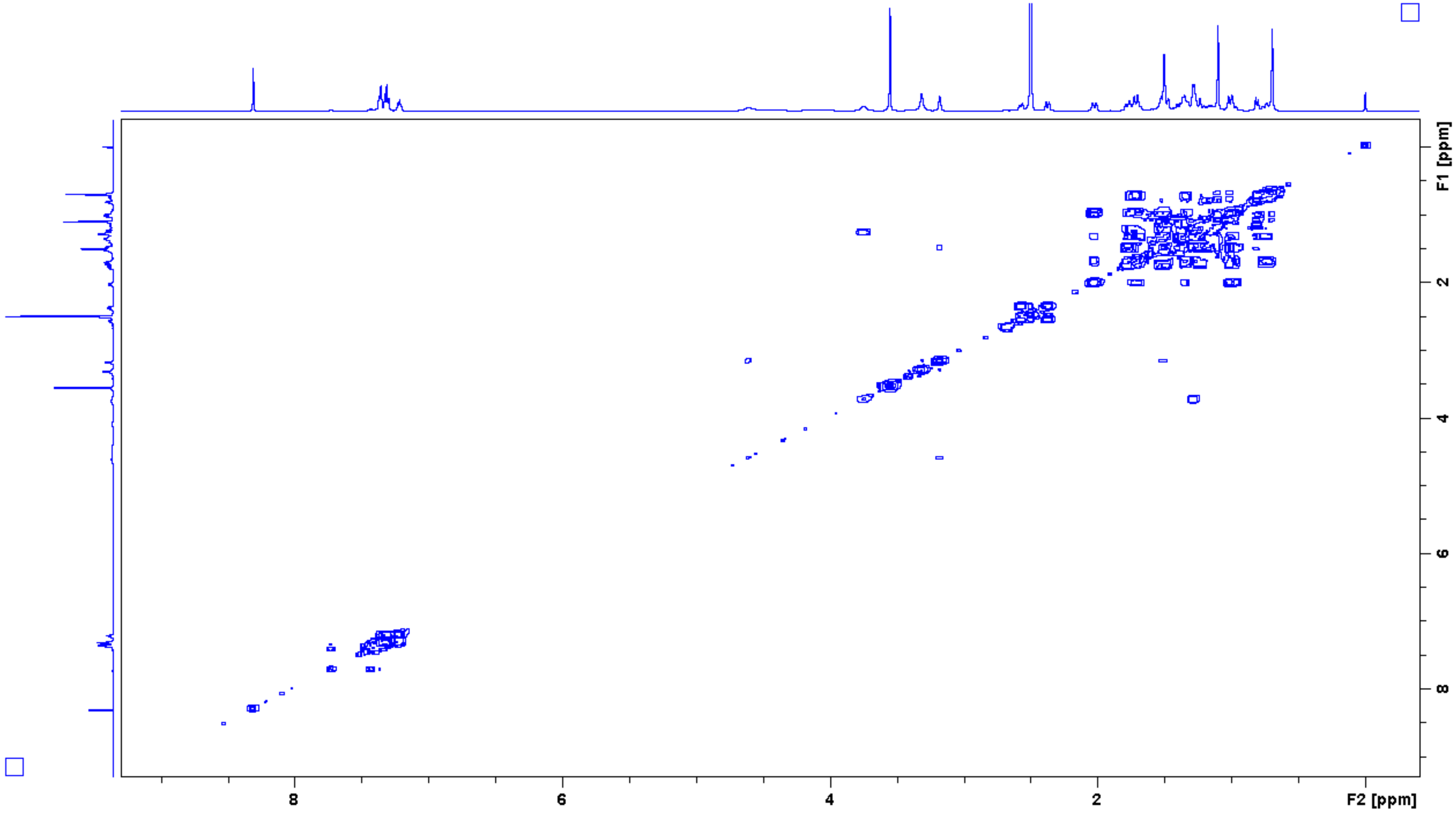

NOESY of compound (4*R*,6a*R*,7*R*,8*R*,9*S*,11b*S*)-methyl 7,8,9-trihydroxy-4,11b-dimethyl-8-((((*R*)-1-phenylethyl)amino)methyl)tetradecahydro-6a,9-methanocyclohepta[a]naphthalene-4-carboxylate **(7)**

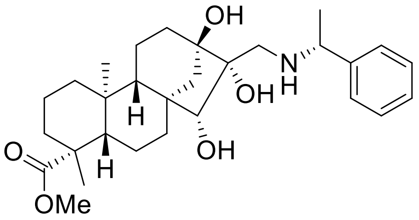

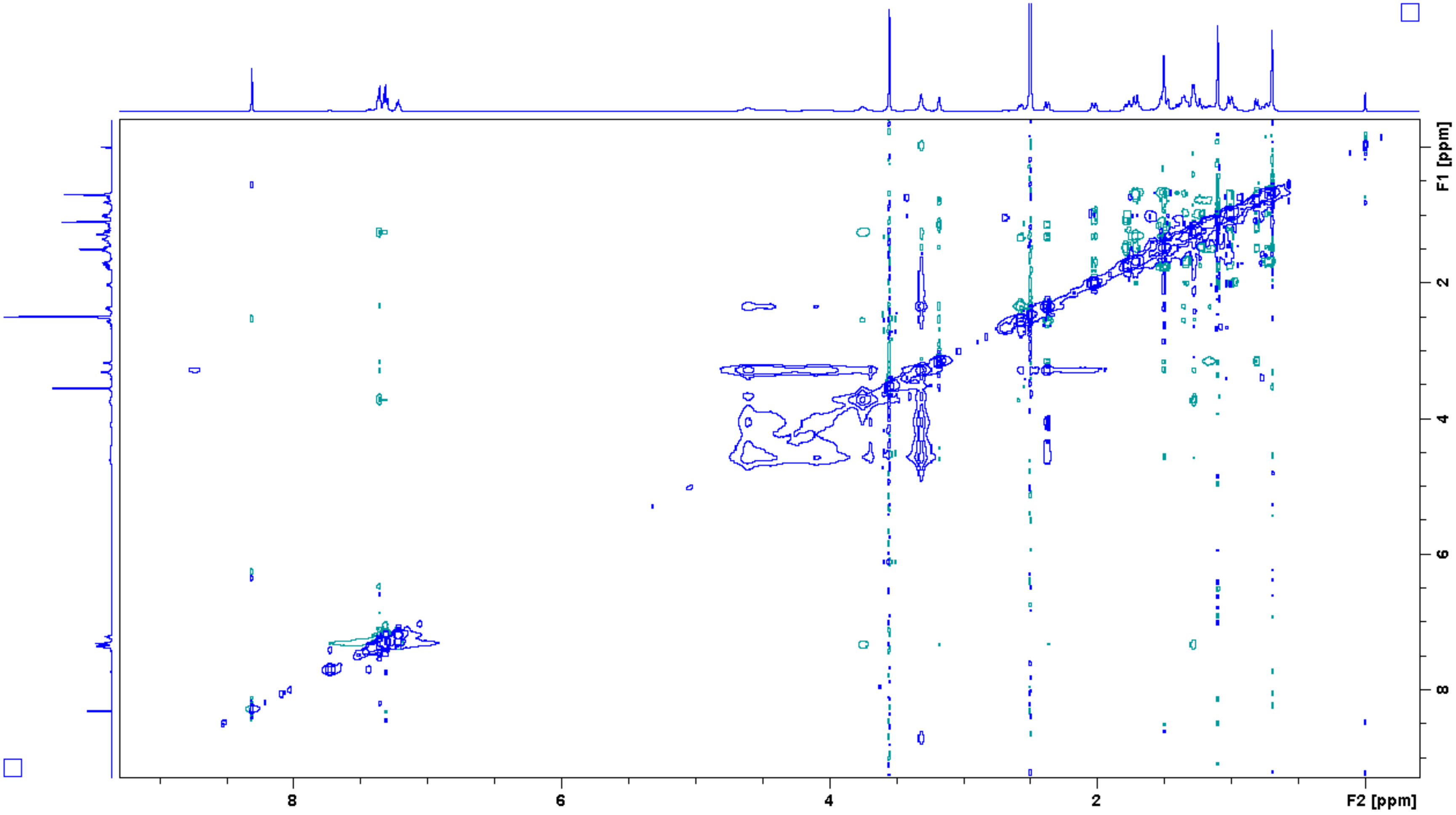

HSQC of compound (4*R*,6a*R*,7*R*,8*R*,9*S*,11b*S*)-methyl 7,8,9-trihydroxy-4,11b-dimethyl-8-((((*R*)-1-phenylethyl)amino)methyl)tetradecahydro-6a,9-methanocyclohepta[a]naphthalene-4-carboxylate **(7)**

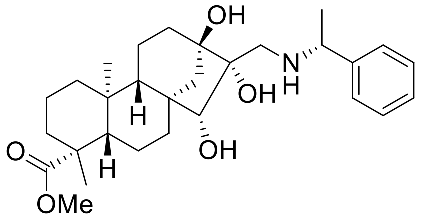

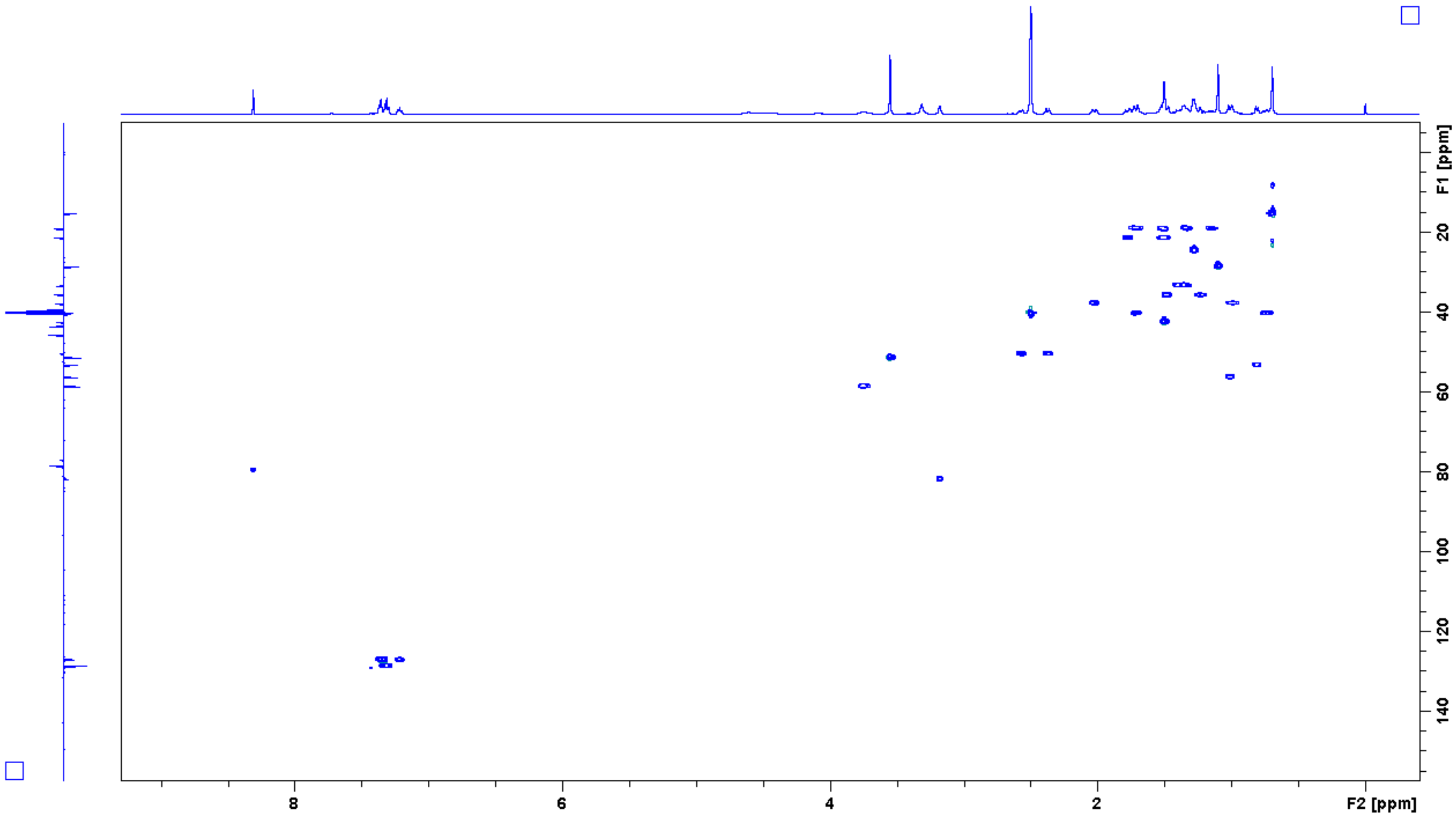

HMBC of compound (4*R*,6a*R*,7*R*,8*R*,9*S*,11b*S*)-methyl 7,8,9-trihydroxy-4,11b-dimethyl-8-((((*R*)-1-phenylethyl)amino)methyl)tetradecahydro-6a,9-methanocyclohepta[a]naphthalene-4-carboxylate **(7)**

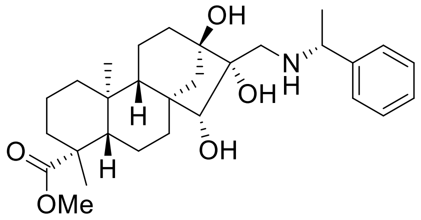

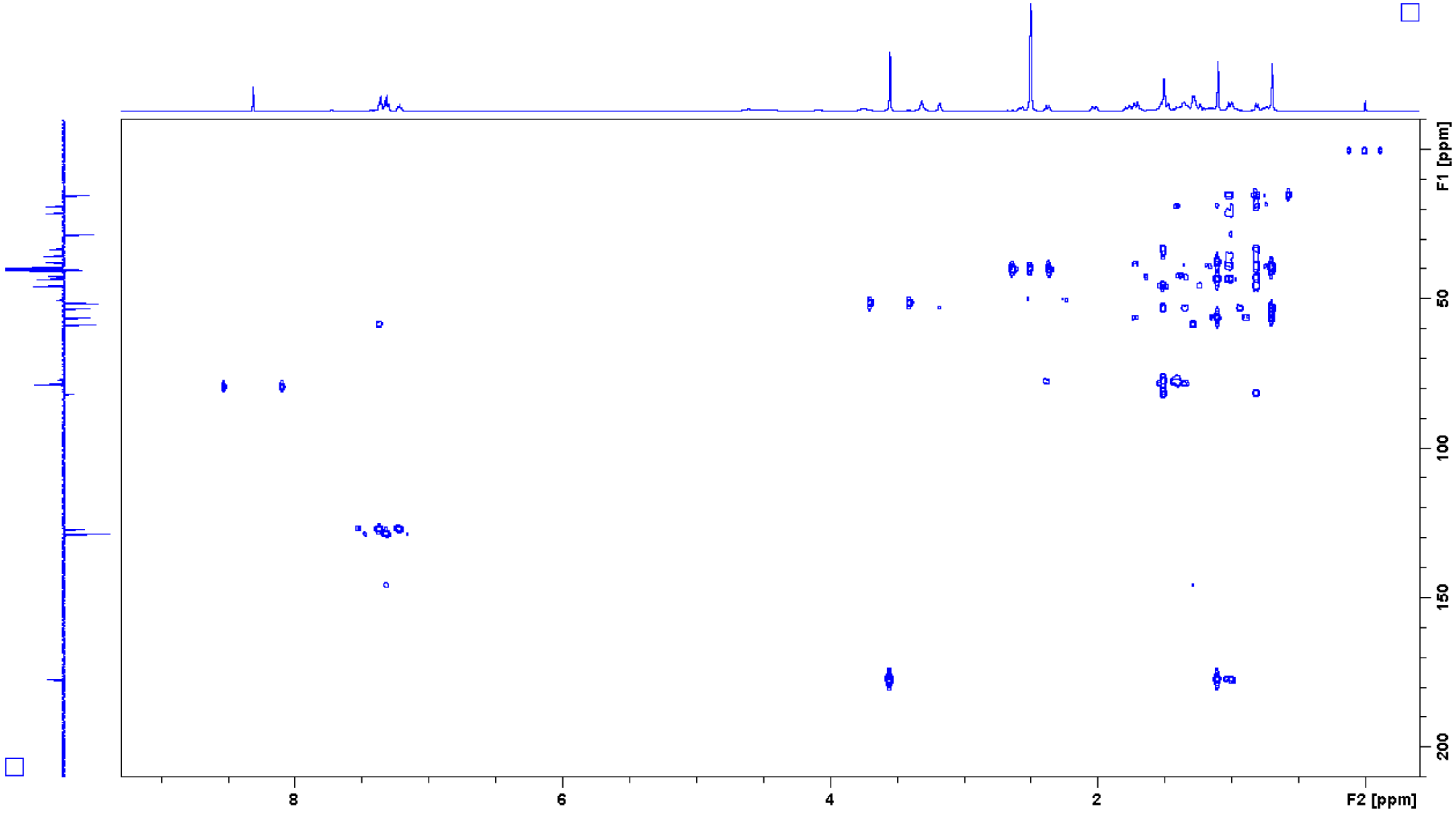

^{1}H-NMR of compound (4*R*,6a*R*,7*R*,8*R*,9*S*,11b*S*)-methyl 7,8,9-trihydroxy-4,11b-dimethyl-8-((((*S*)-1-phenylethyl)amino)methyl)tetradecahydro-6a,9-methanocyclohepta[a]naphthalene-4-carboxylate **(8)**

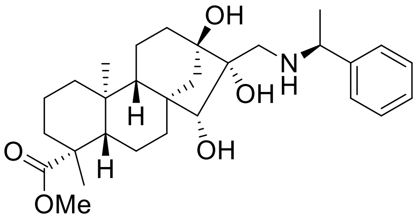

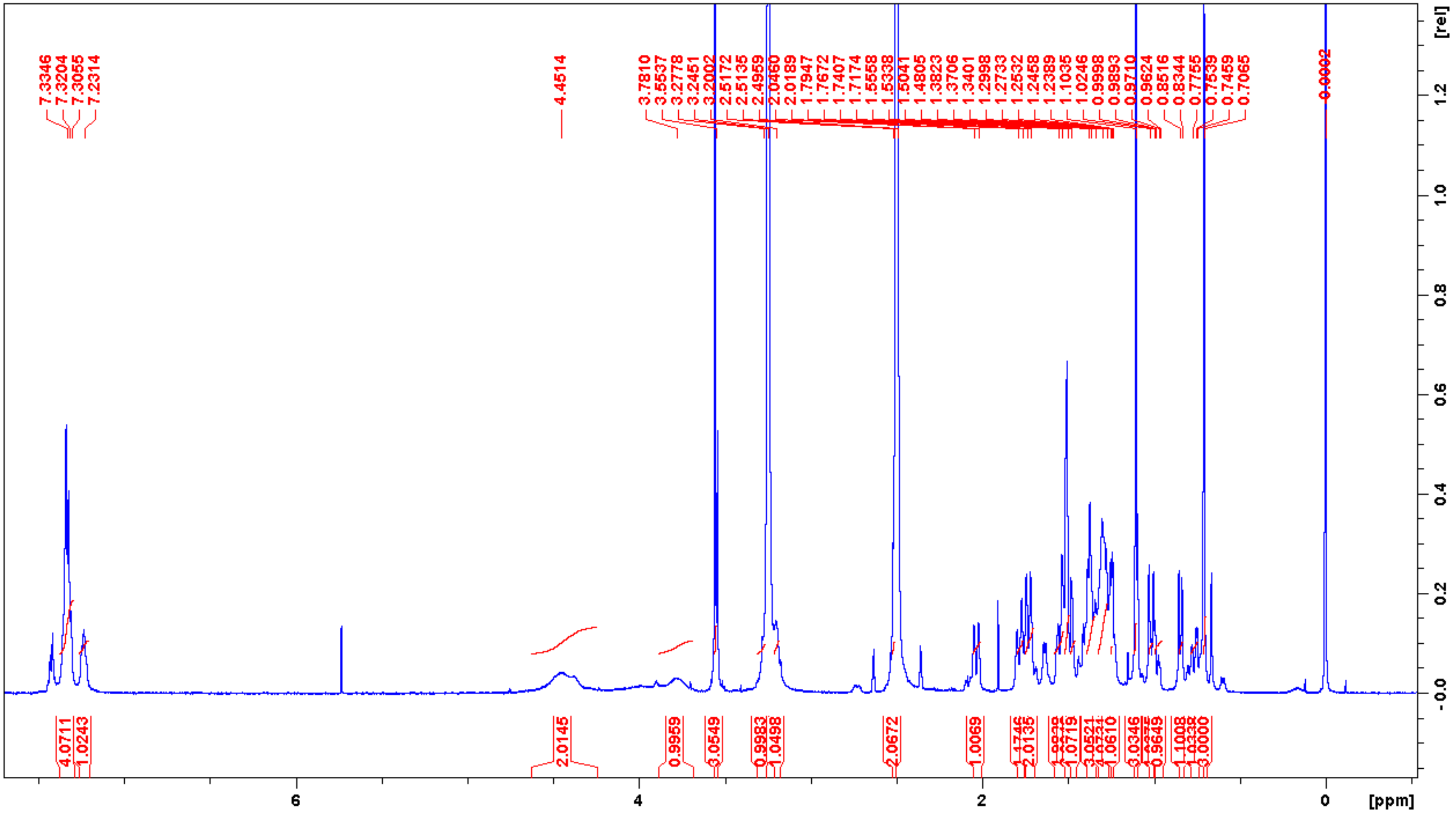

^{13}C-NMR of compound (4*R*,6a*R*,7*R*,8*R*,9*S*,11b*S*)-methyl 7,8,9-trihydroxy-4,11b-dimethyl-8-((((*S*)-1-phenylethyl)amino)methyl)tetradecahydro-6a,9-methanocyclohepta[a]naphthalene-4-carboxylate **(8)**

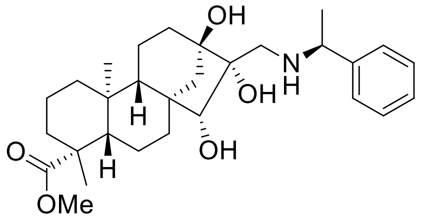

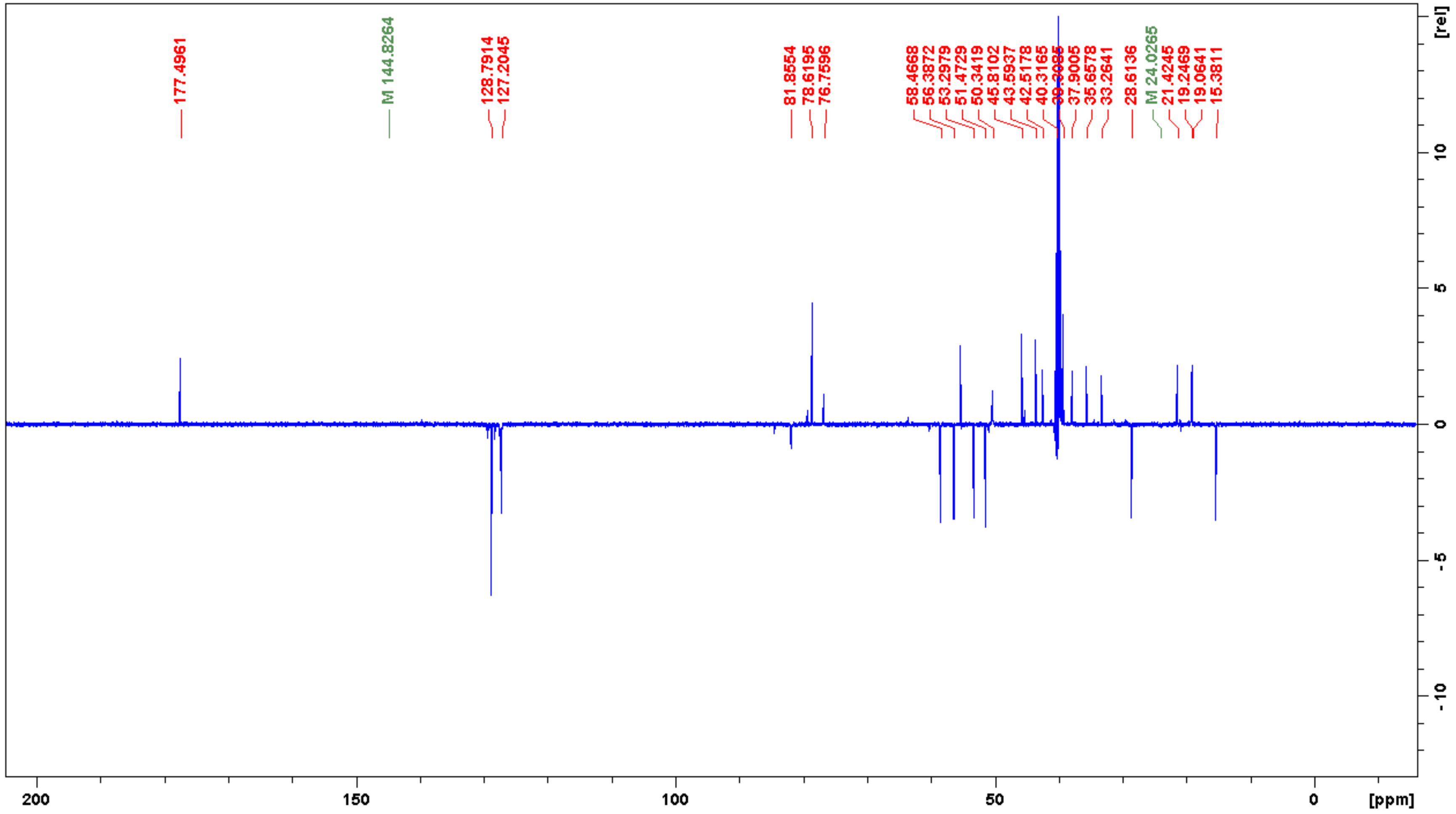

COSY of compound (4*R*,6a*R*,7*R*,8*R*,9*S*,11b*S*)-methyl 7,8,9-trihydroxy-4,11b-dimethyl-8-((((*S*)-1-phenylethyl)amino)methyl)tetradecahydro-6a,9-methanocyclohepta[a]naphthalene-4-carboxylate **(8)**

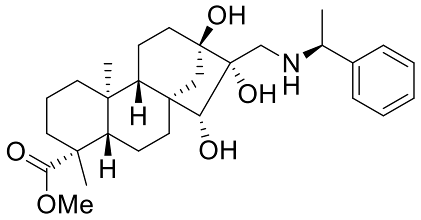

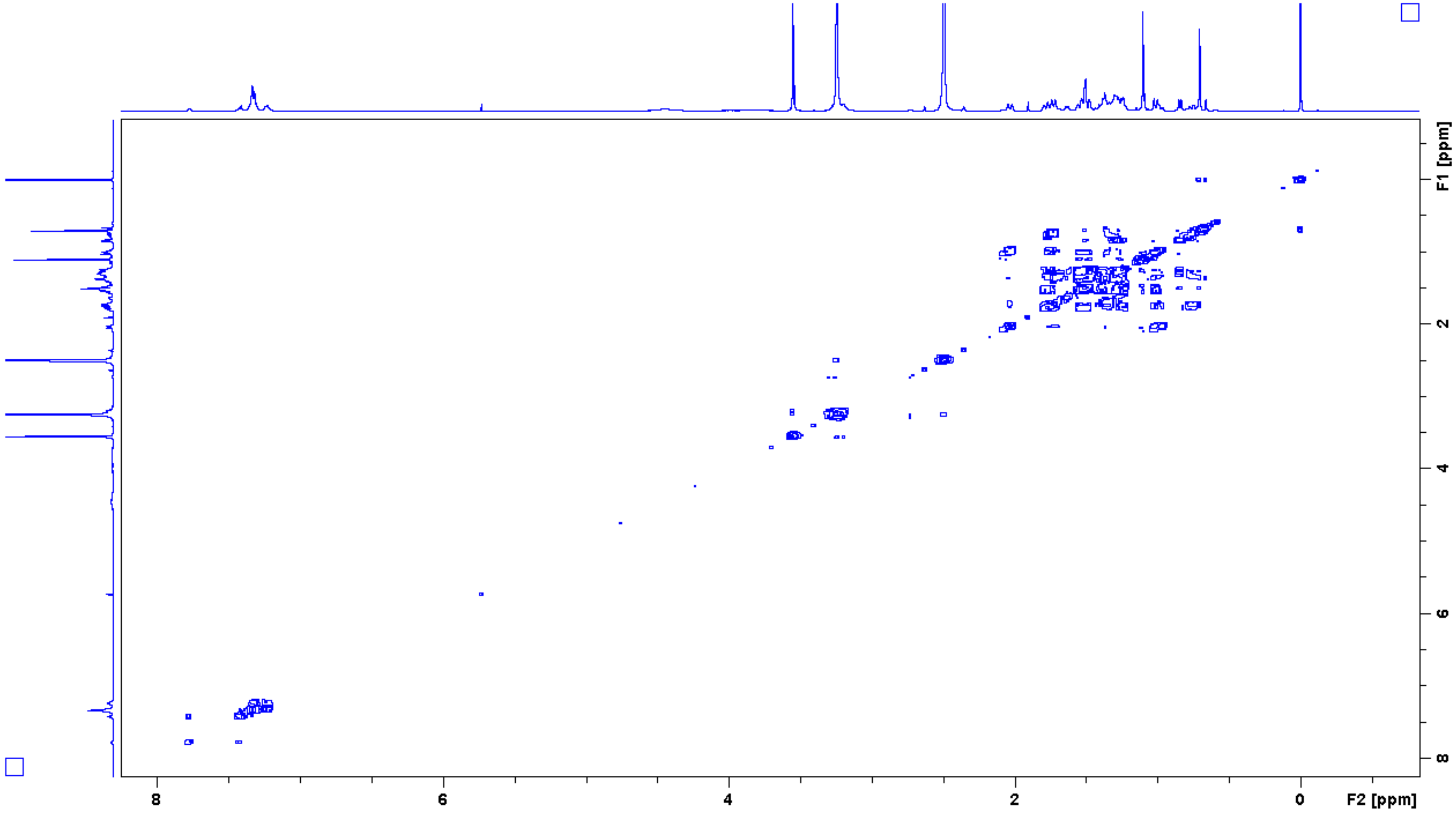

NOESY of compound (4*R*,6a*R*,7*R*,8*R*,9*S*,11b*S*)-methyl 7,8,9-trihydroxy-4,11b-dimethyl-8-((((*S*)-1-phenylethyl)amino)methyl)tetradecahydro-6a,9-methanocyclohepta[a]naphthalene-4-carboxylate **(8)**

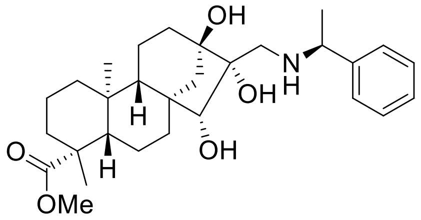

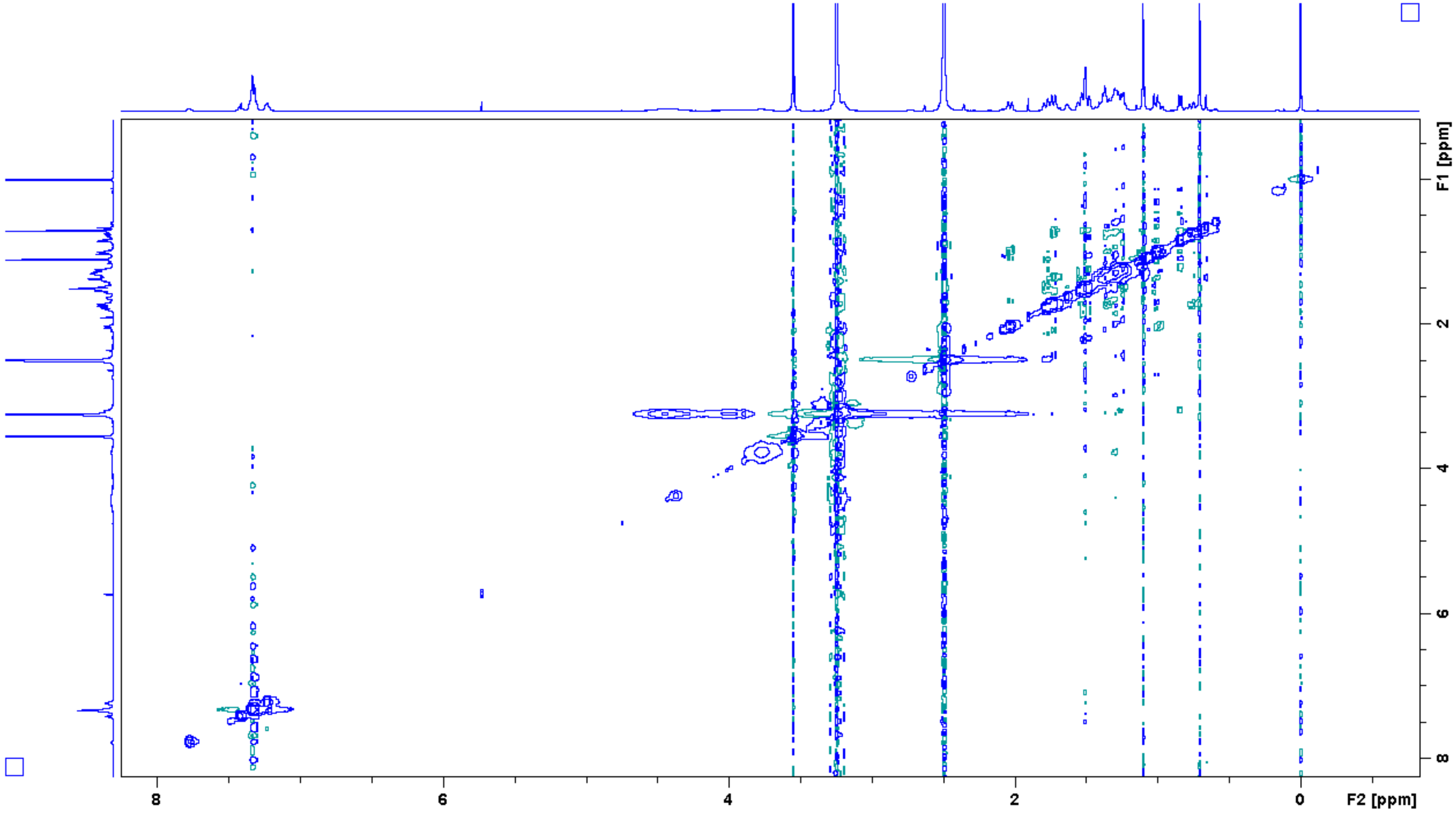

HSQC of compound (4*R*,6a*R*,7*R*,8*R*,9*S*,11b*S*)-methyl 7,8,9-trihydroxy-4,11b-dimethyl-8-((((*S*)-1-phenylethyl)amino)methyl)tetradecahydro-6a,9-methanocyclohepta[a]naphthalene-4-carboxylate **(8)**

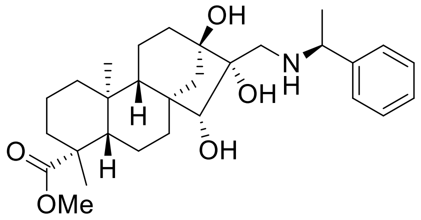

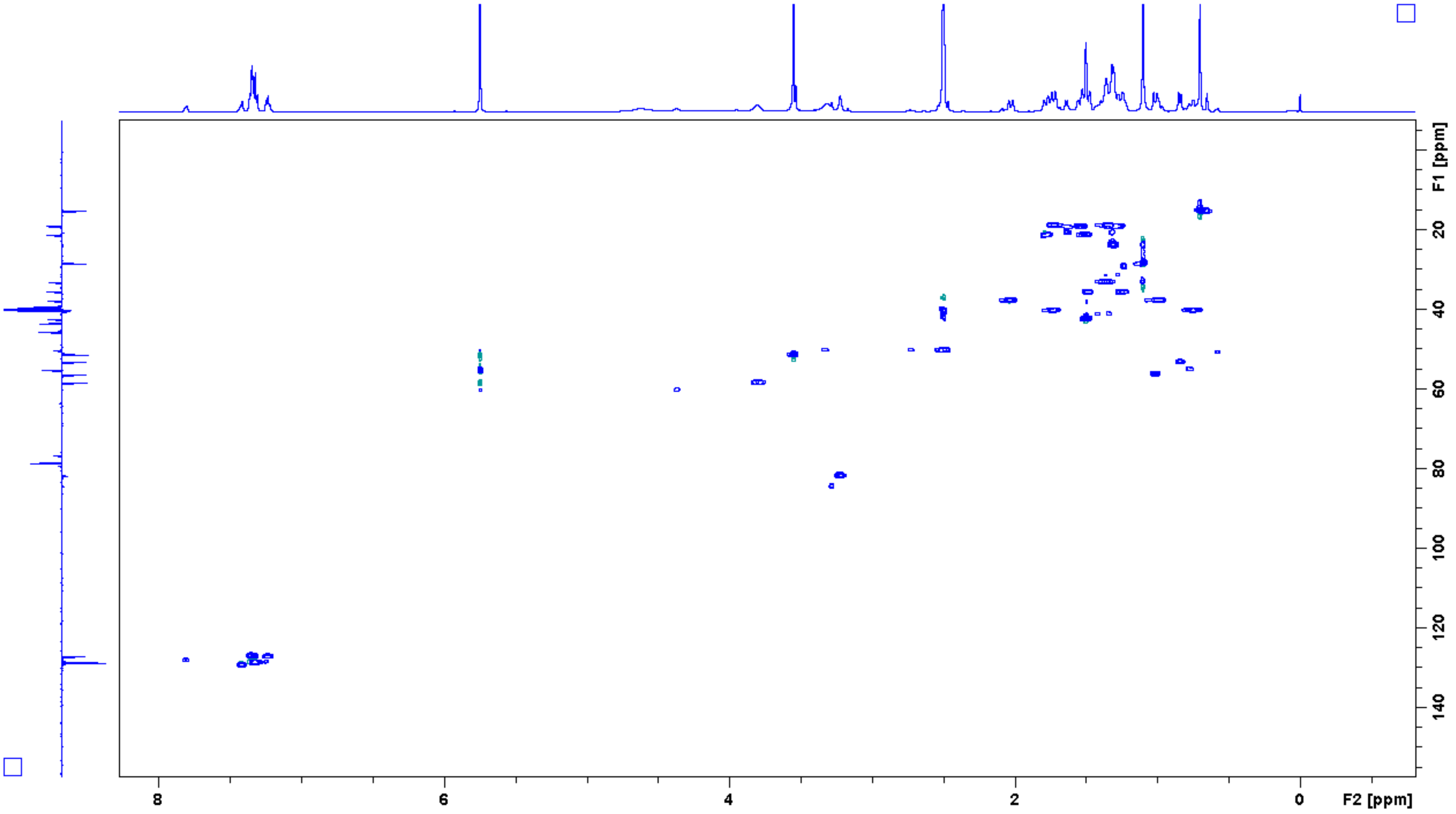

HMBC of compound (4*R*,6a*R*,7*R*,8*R*,9*S*,11b*S*)-methyl 7,8,9-trihydroxy-4,11b-dimethyl-8-((((*S*)-1-phenylethyl)amino)methyl)tetradecahydro-6a,9-methanocyclohepta[a]naphthalene-4-carboxylate **(8)**

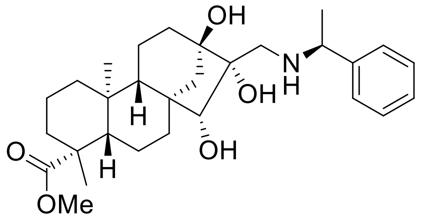

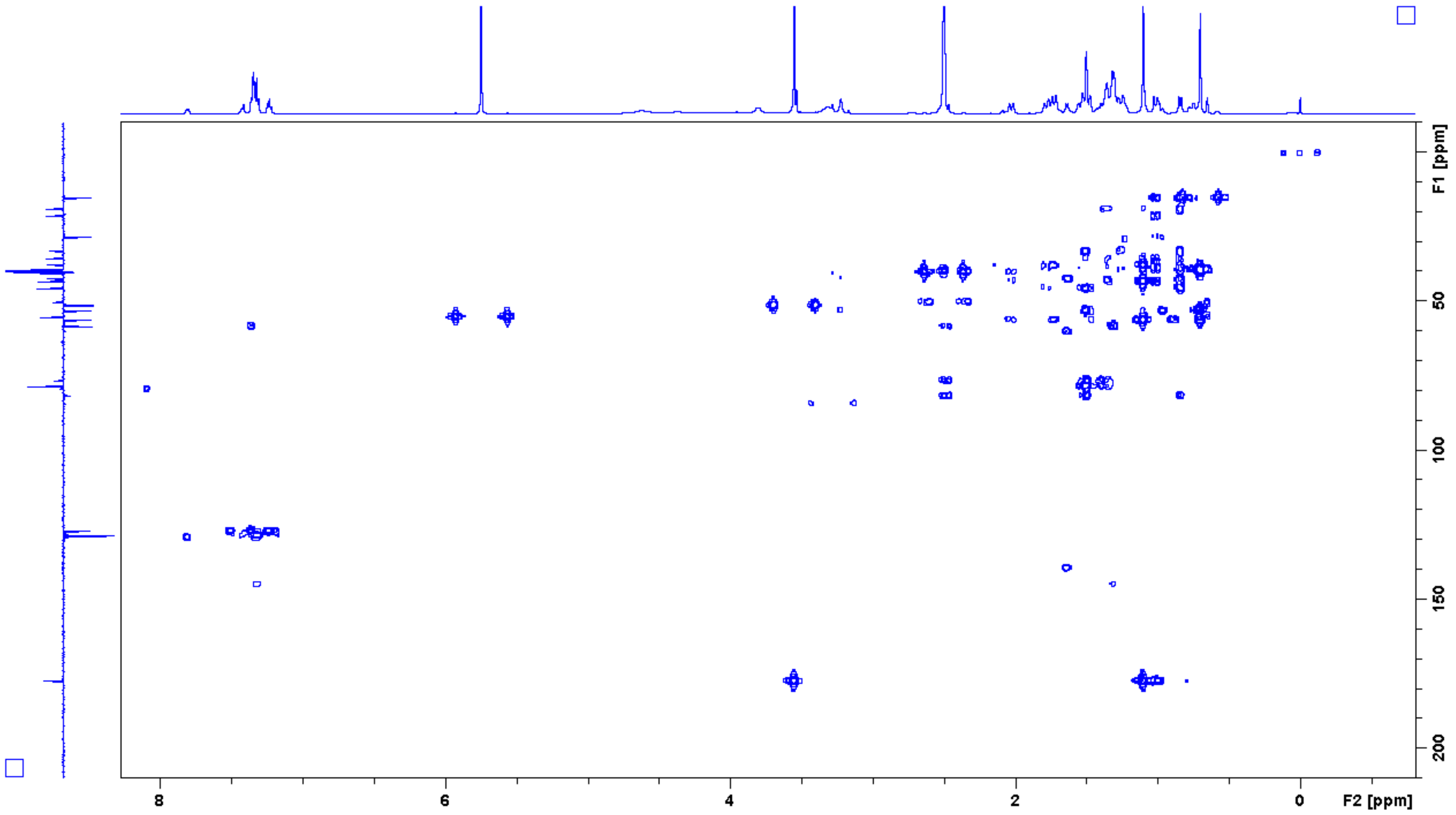

^{1}H-NMR of compound (4*R*,6a*R*,7*R*,8*R*,9*S*,11b*S*)-methyl 8-(((4-fluorobenzyl)amino)methyl)-7,8,9-trihydroxy-4,11b-dimethyltetradecahydro-6a,9-methanocyclohepta[a]naphthalene-4-carboxylate **(9)**

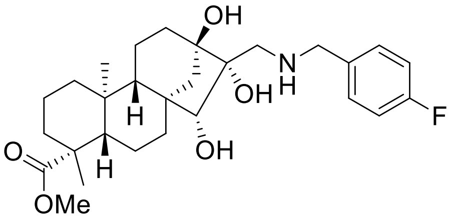

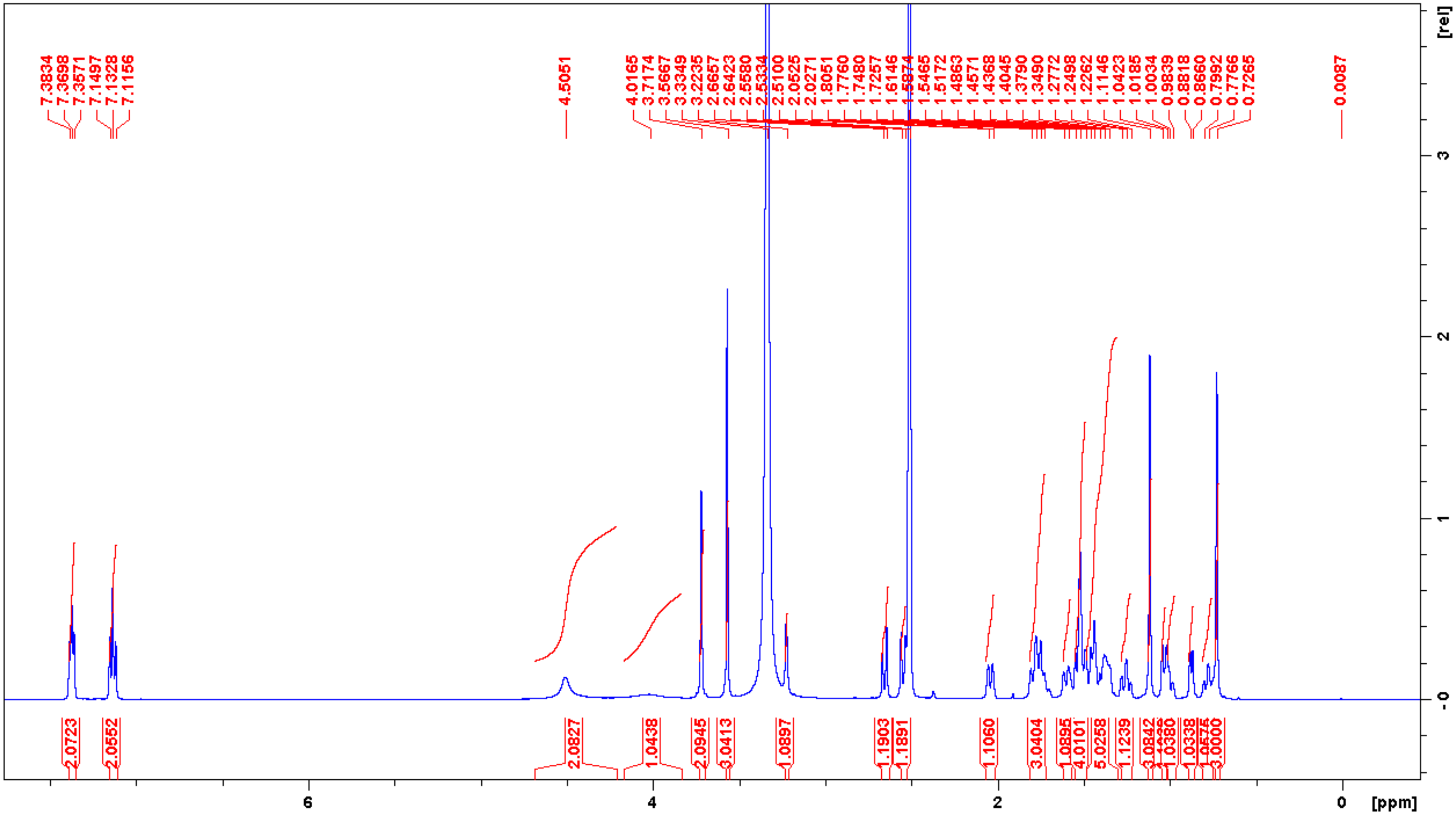

^{13}C-NMR of compound (4*R*,6a*R*,7*R*,8*R*,9*S*,11b*S*)-methyl 8-(((4-fluorobenzyl)amino)methyl)-7,8,9-trihydroxy-4,11b-dimethyltetradecahydro-6a,9-methanocyclohepta[a]naphthalene-4-carboxylate **(9)**

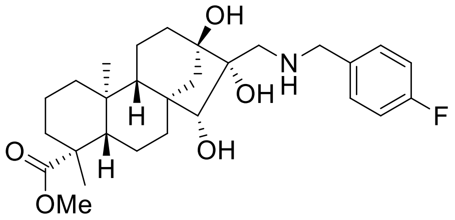

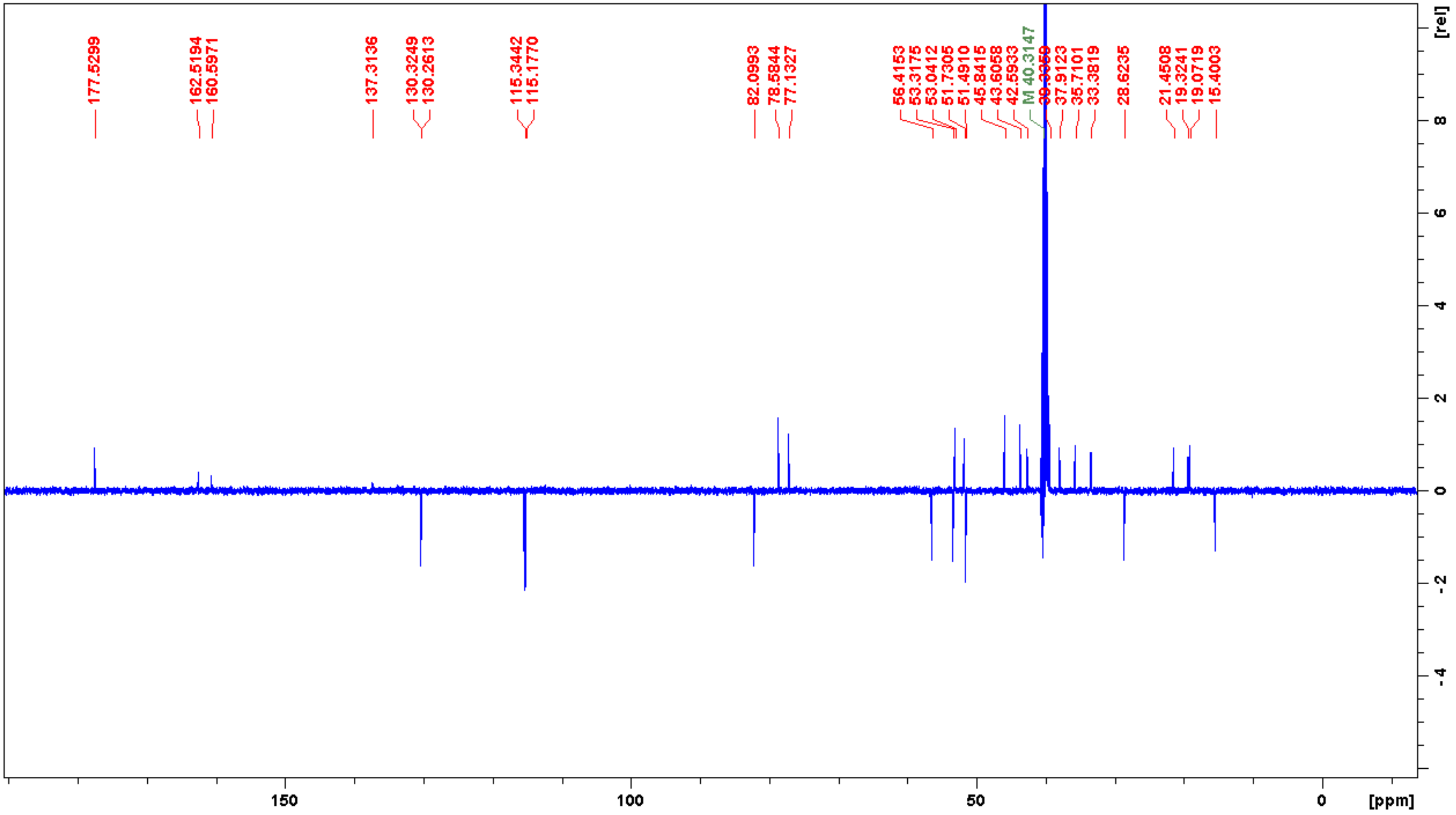

COSY of compound (4*R*,6a*R*,7*R*,8*R*,9*S*,11b*S*)-methyl 8-(((4-fluorobenzyl)amino)methyl)-7,8,9-trihydroxy-4,11b-dimethyltetradecahydro-6a,9-methanocyclohepta[a]naphthalene-4-carboxylate **(9)**

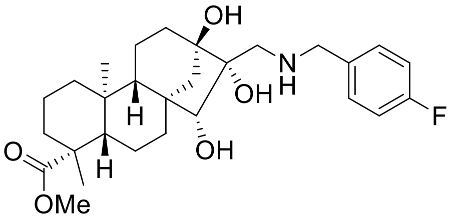

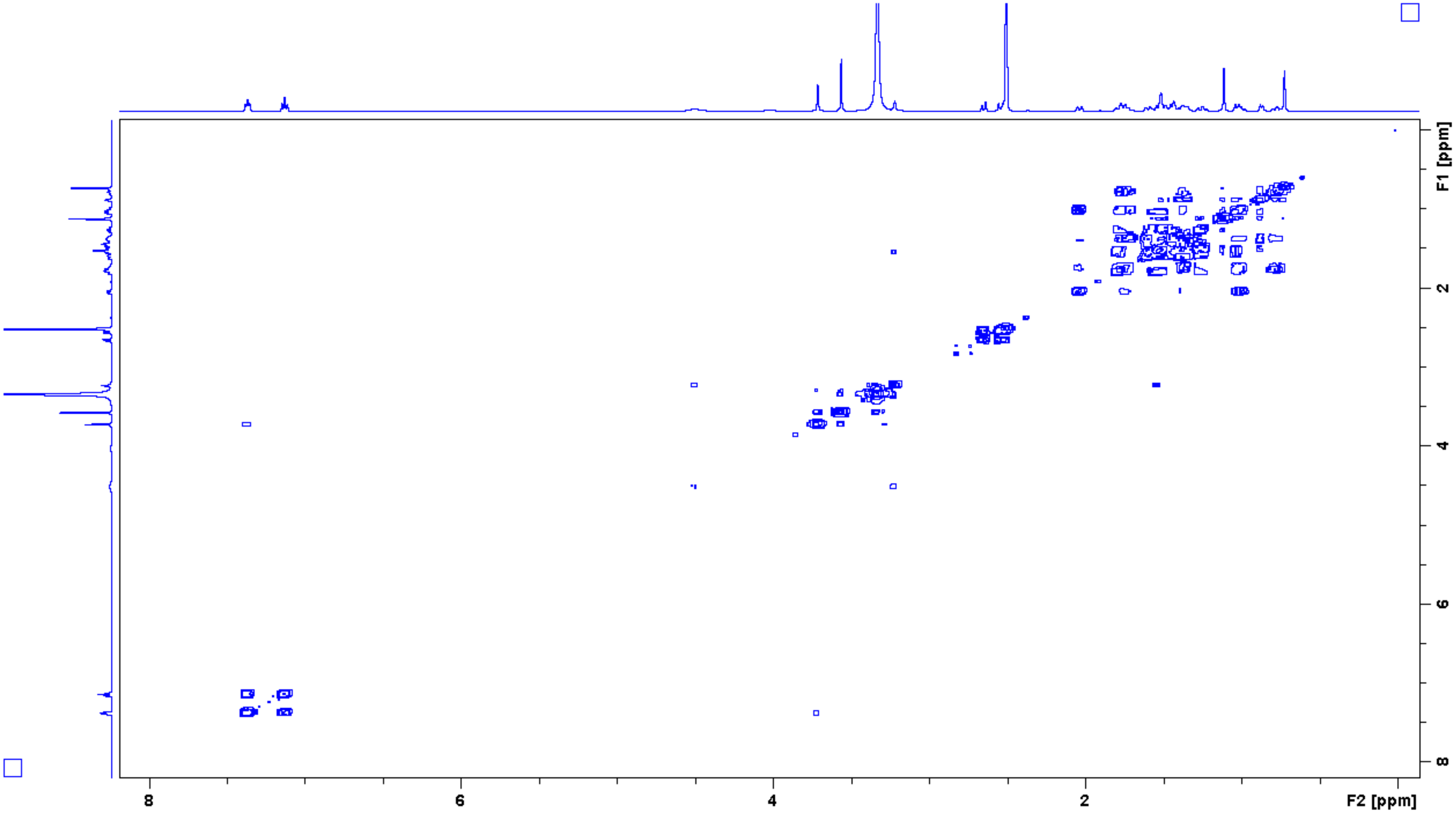

NOESY of compound (4*R*,6a*R*,7*R*,8*R*,9*S*,11b*S*)-methyl 8-(((4-fluorobenzyl)amino)methyl)-7,8,9-trihydroxy-4,11b-dimethyltetradecahydro-6a,9-methanocyclohepta[a]naphthalene-4-carboxylate **(9)**

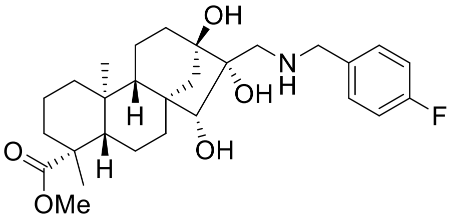

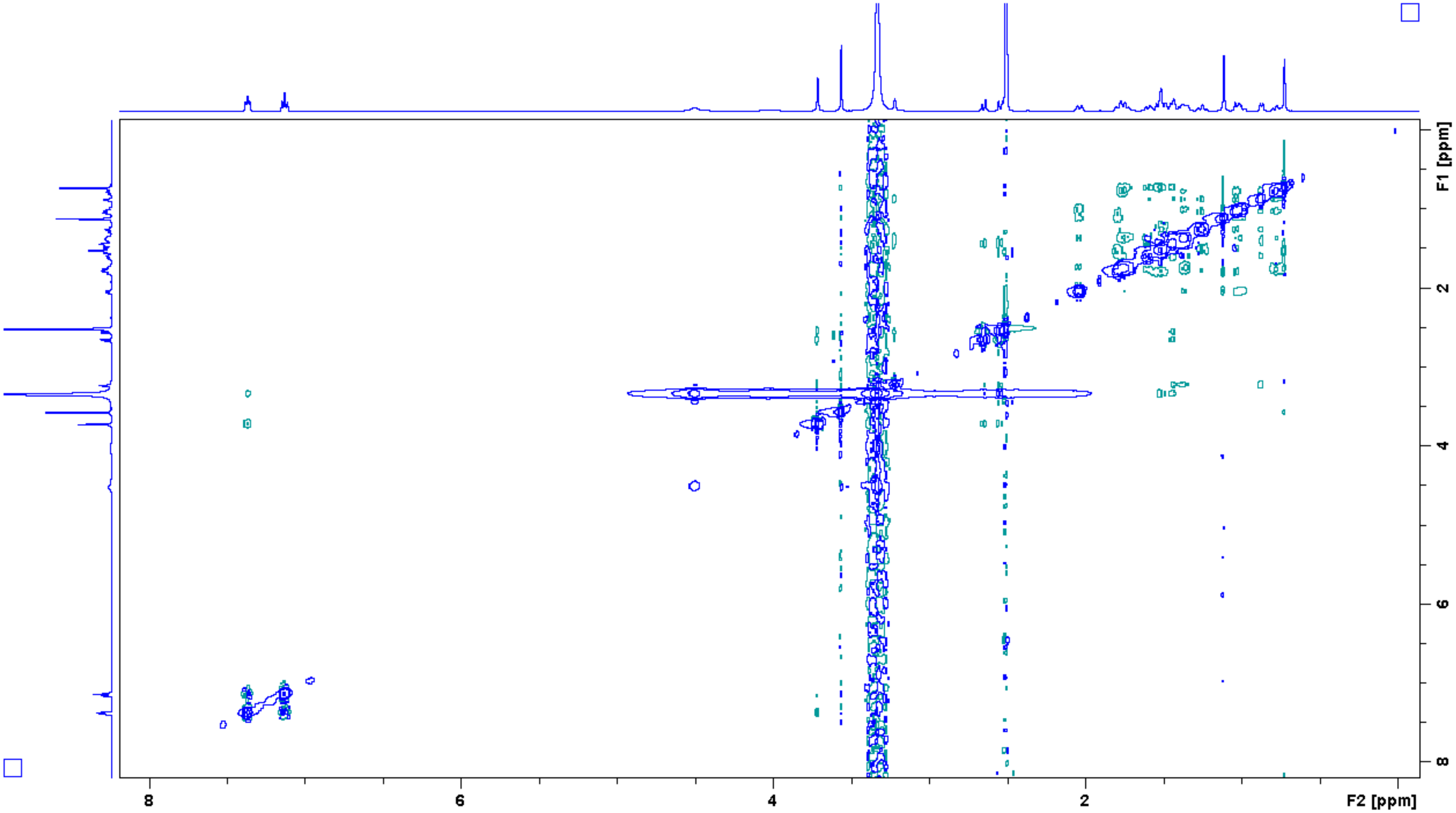

HSQC of compound (4*R*,6a*R*,7*R*,8*R*,9*S*,11b*S*)-methyl 8-(((4-fluorobenzyl)amino)methyl)-7,8,9-trihydroxy-4,11b-dimethyltetradecahydro-6a,9-methanocyclohepta[a]naphthalene-4-carboxylate **(9)**

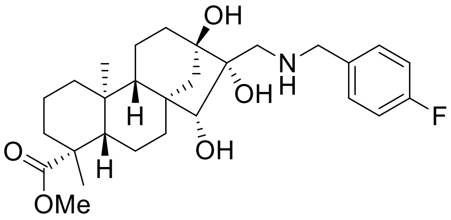

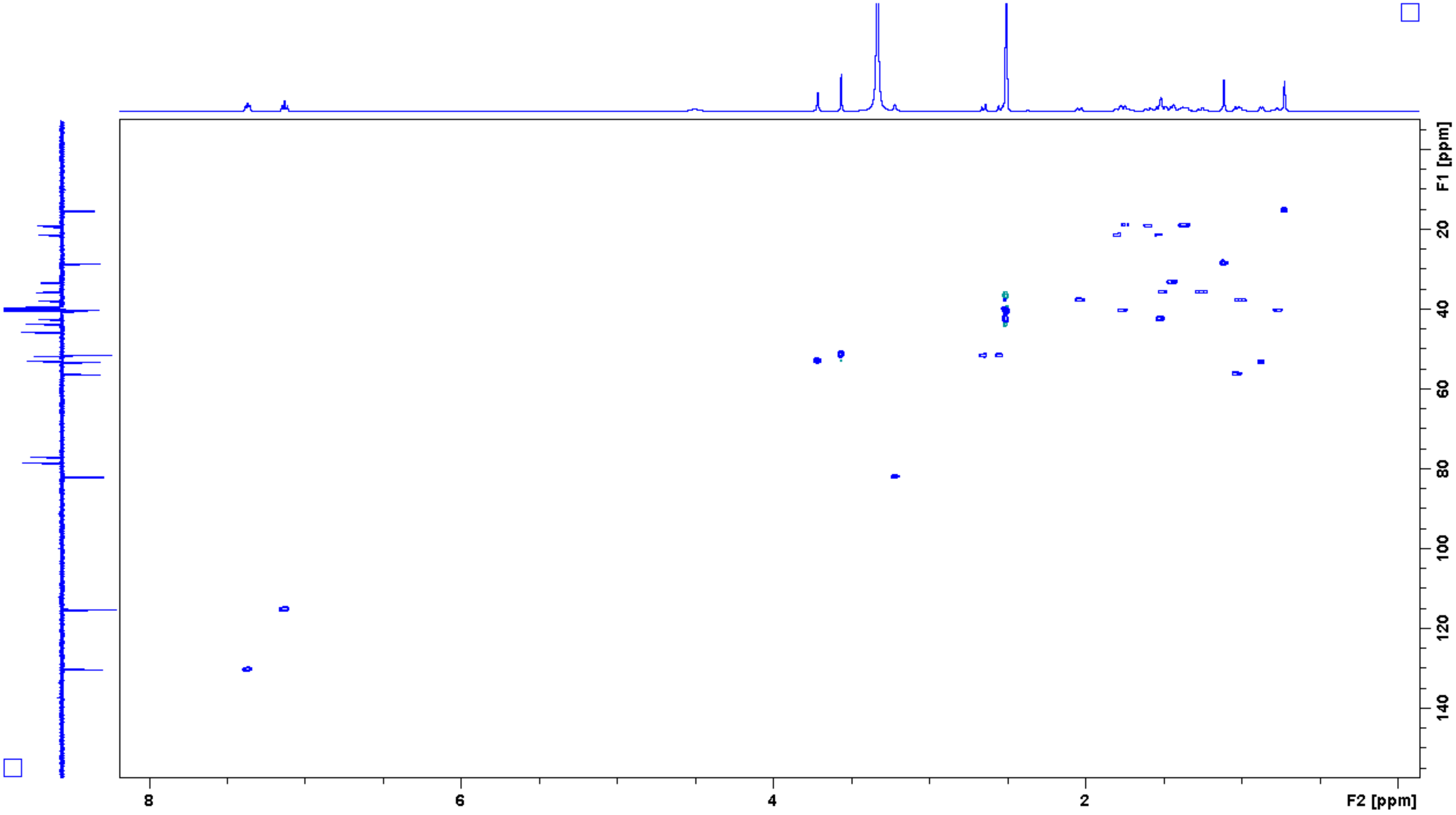

HMBC of compound (4*R*,6a*R*,7*R*,8*R*,9*S*,11b*S*)-methyl 8-(((4-fluorobenzyl)amino)methyl)-7,8,9-trihydroxy-4,11b-dimethyltetradecahydro-6a,9-methanocyclohepta[a]naphthalene-4-carboxylate **(9)**

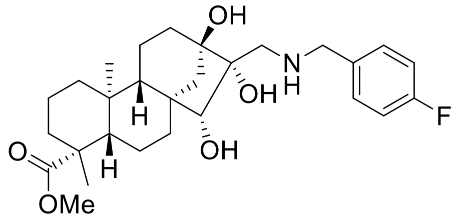

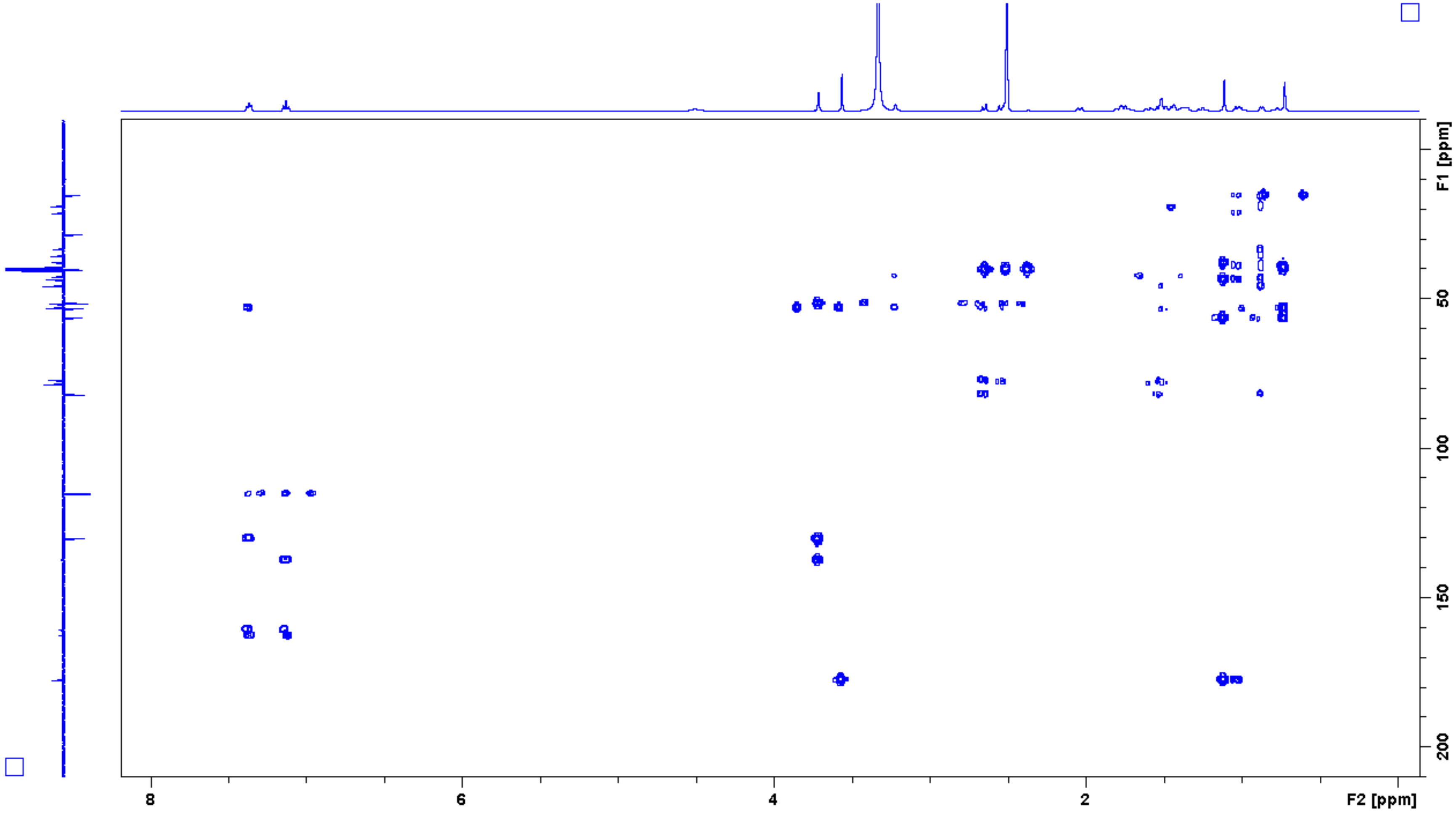

^{19}F-NMR of compound (4*R*,6a*R*,7*R*,8*R*,9*S*,11b*S*)-methyl 8-(((4-fluorobenzyl)amino)methyl)-7,8,9-trihydroxy-4,11b-dimethyltetradecahydro-6a,9-methanocyclohepta[a]naphthalene-4-carboxylate **(9)**

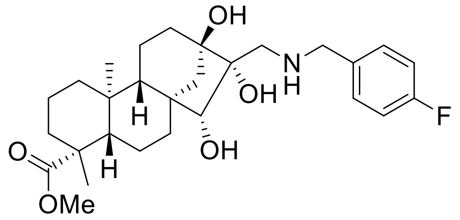

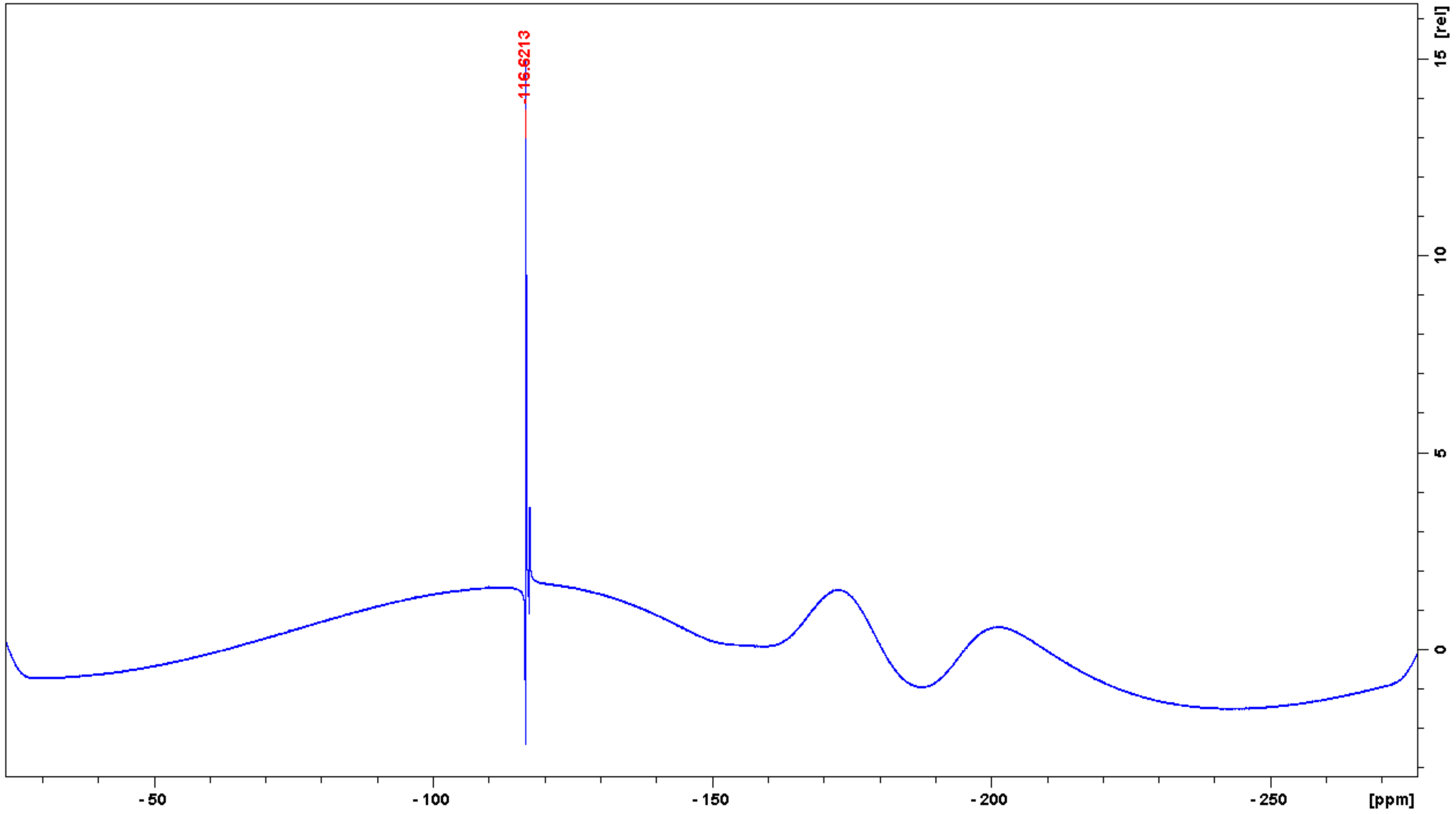

^{1}H-NMR of compound (4*R*,6a*R*,7*R*,8*R*,9*S*,11b*S*)-methyl 7,8,9-trihydroxy-8-(((4-methoxybenzyl)amino)methyl)-4,11b-dimethyltetradecahydro-6a,9-methanocyclohepta[a]naphthalene-4-carboxylate **(10)**

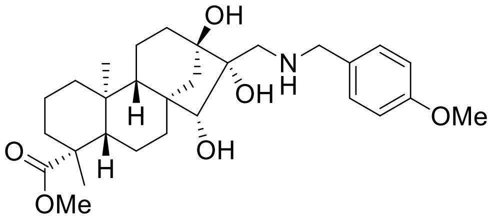

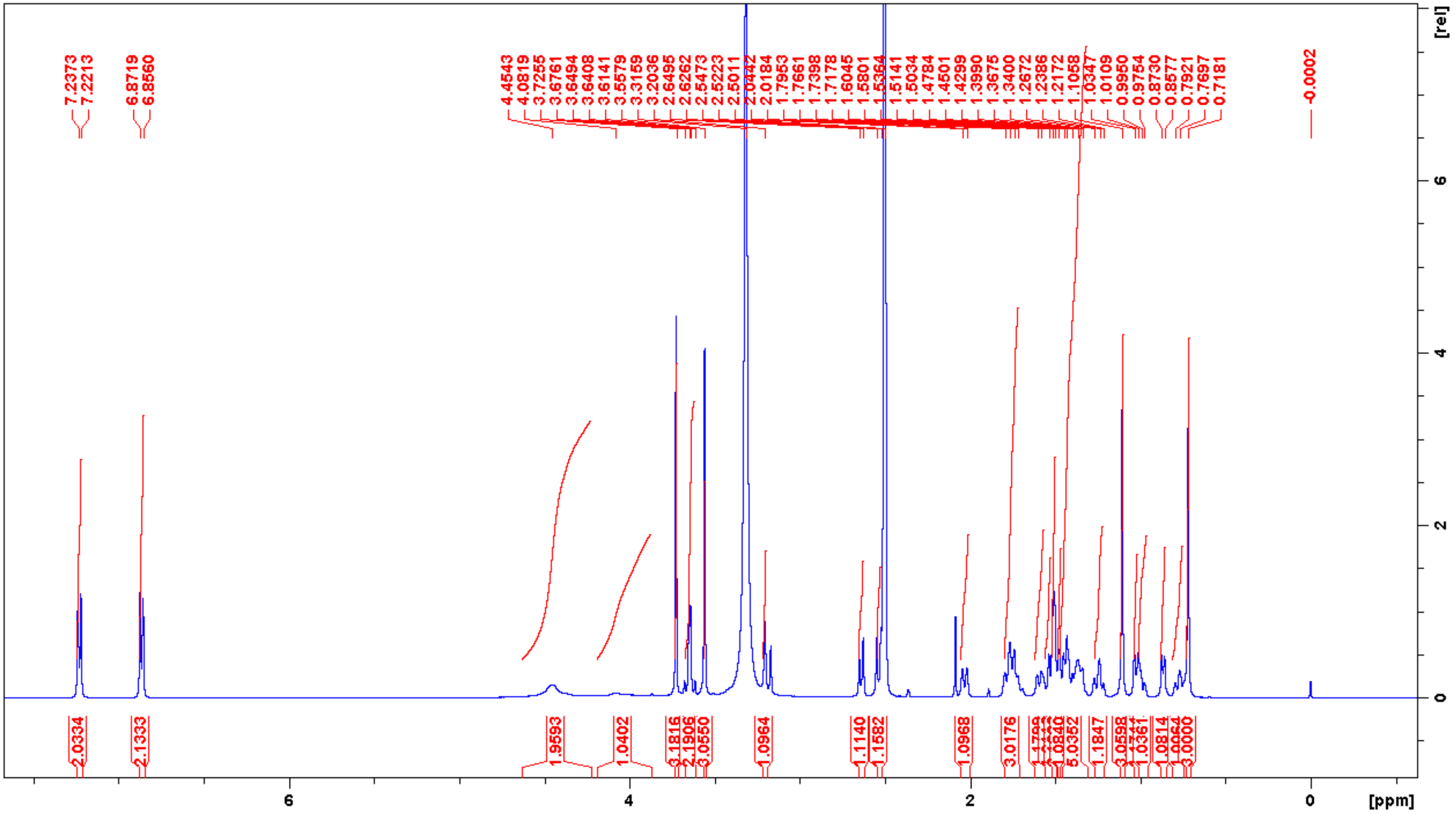

^{13}C-NMR of compound (4*R*,6a*R*,7*R*,8*R*,9*S*,11b*S*)-methyl 7,8,9-trihydroxy-8-(((4-methoxybenzyl)amino)methyl)-4,11b-dimethyltetradecahydro-6a,9-methanocyclohepta[a]naphthalene-4-carboxylate **(10)**

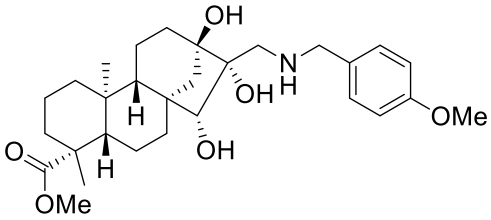

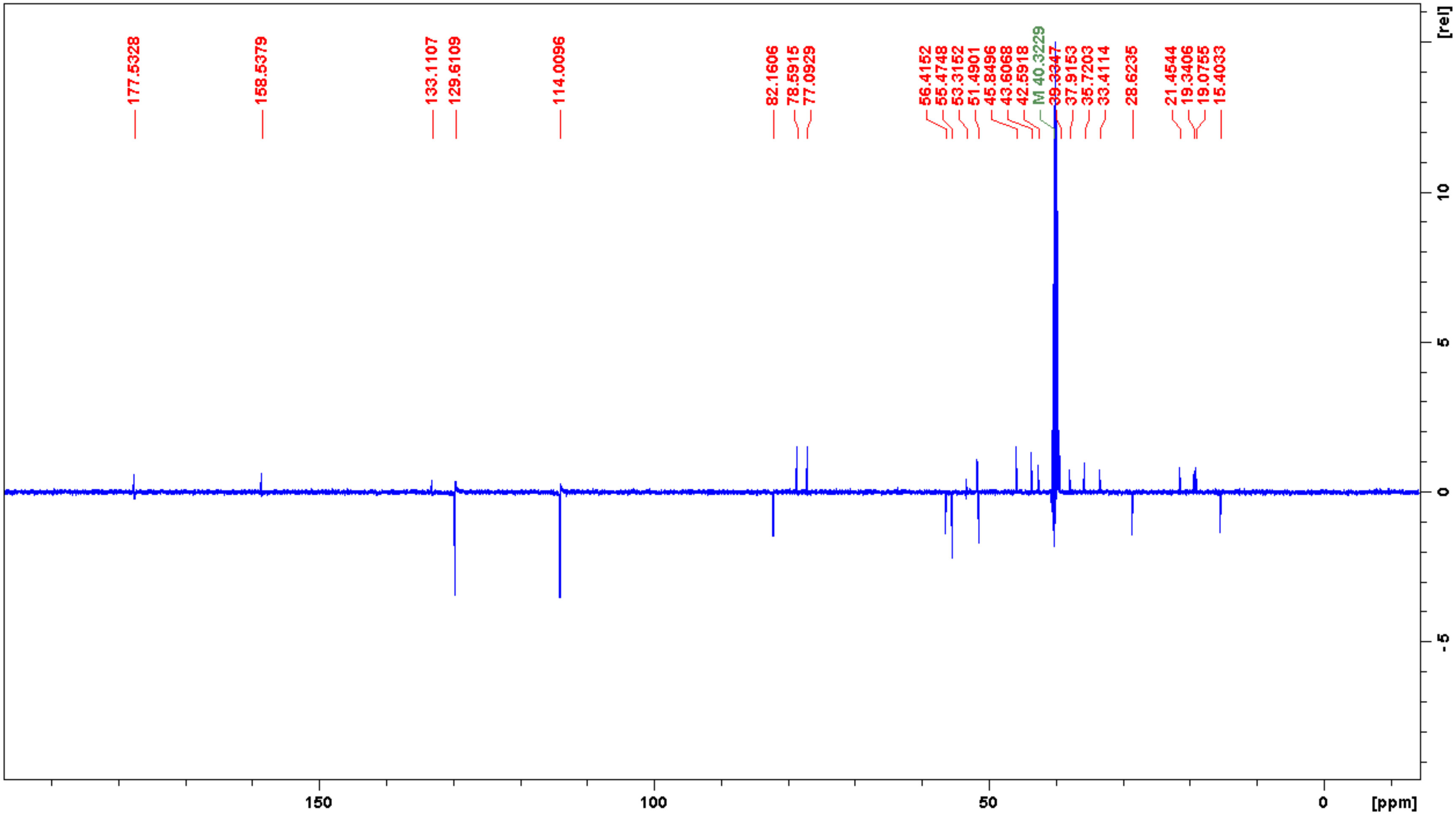

COSY of compound (4*R*,6a*R*,7*R*,8*R*,9*S*,11b*S*)-methyl 7,8,9-trihydroxy-8-(((4-methoxybenzyl)amino)methyl)-4,11b-dimethyltetradecahydro-6a,9-methanocyclohepta[a]naphthalene-4-carboxylate **(10)**

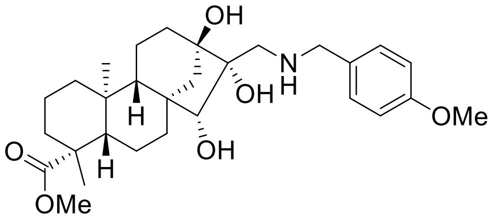

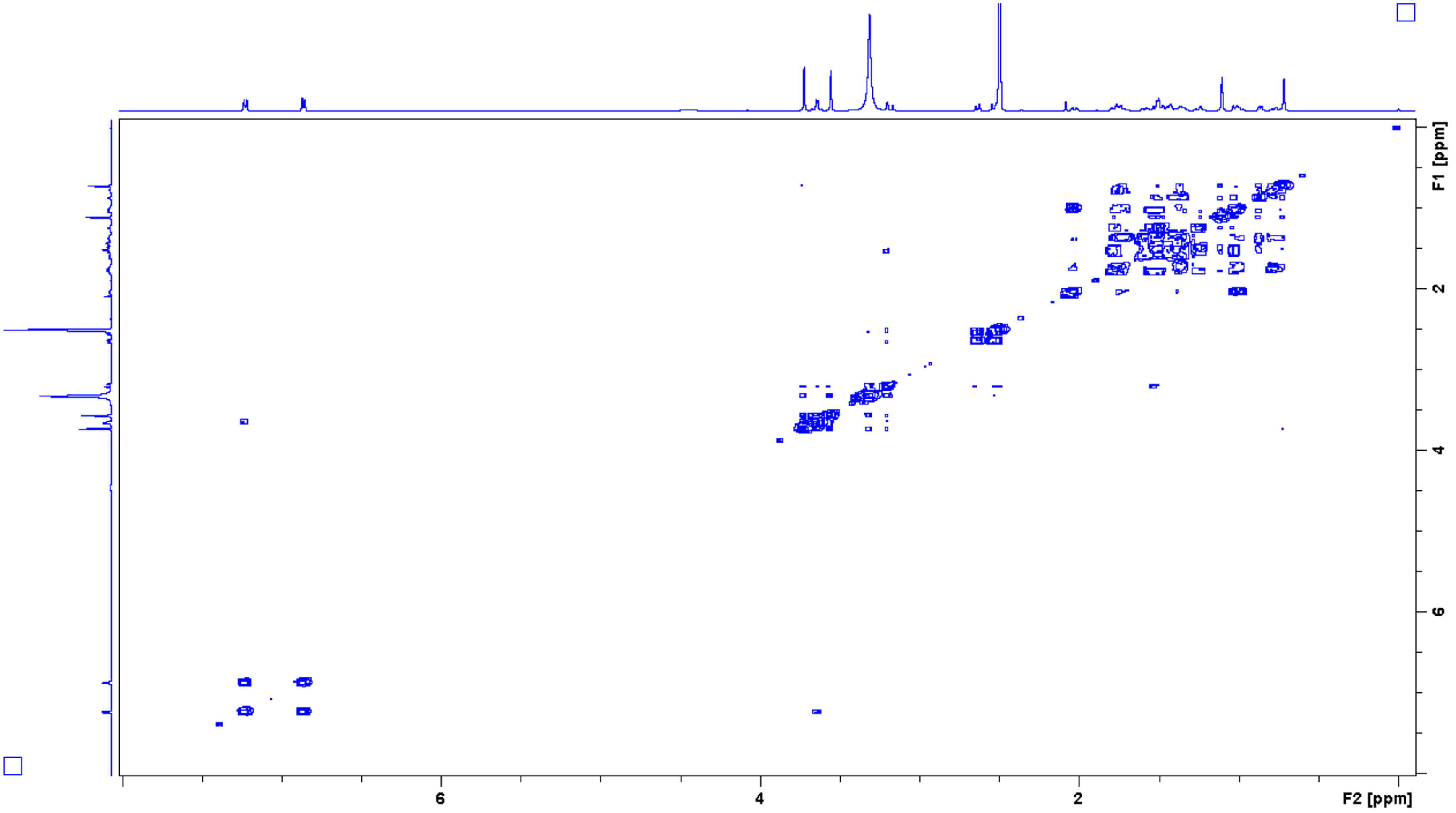

NOESY of compound (4*R*,6a*R*,7*R*,8*R*,9*S*,11b*S*)-methyl 7,8,9-trihydroxy-8-(((4-methoxybenzyl)amino)methyl)-4,11b-dimethyltetradecahydro-6a,9-methanocyclohepta[a]naphthalene-4-carboxylate **(10)**

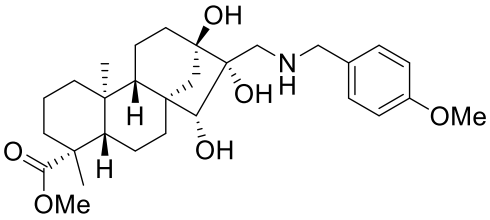

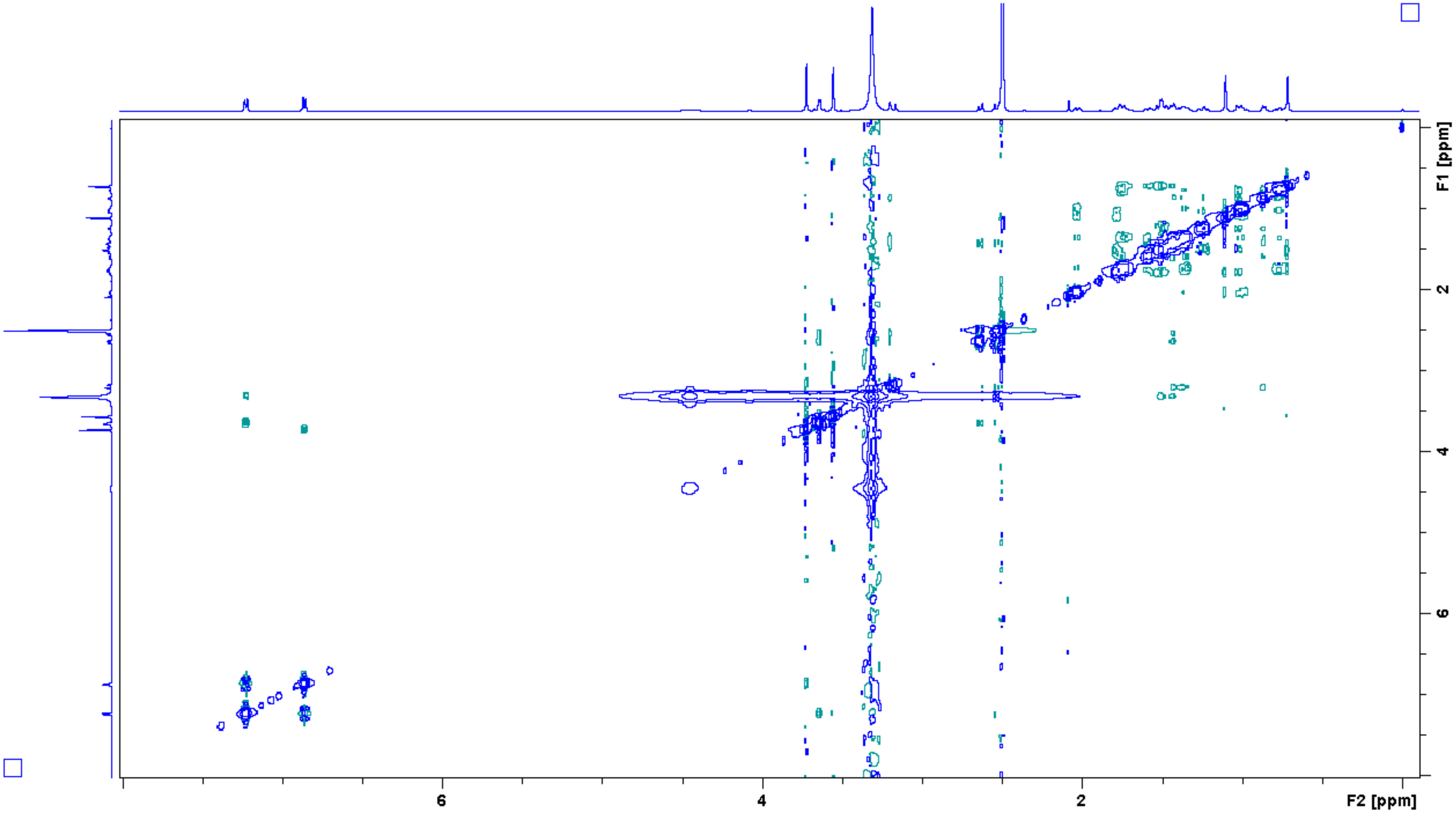

HSQC of compound (4*R*,6a*R*,7*R*,8*R*,9*S*,11b*S*)-methyl 7,8,9-trihydroxy-8-(((4-methoxybenzyl)amino)methyl)-4,11b-dimethyltetradecahydro-6a,9-methanocyclohepta[a]naphthalene-4-carboxylate **(10)**

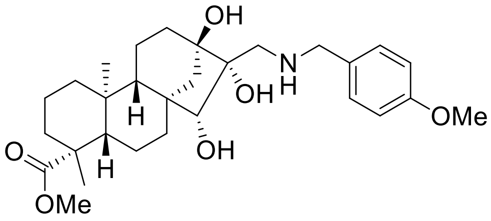

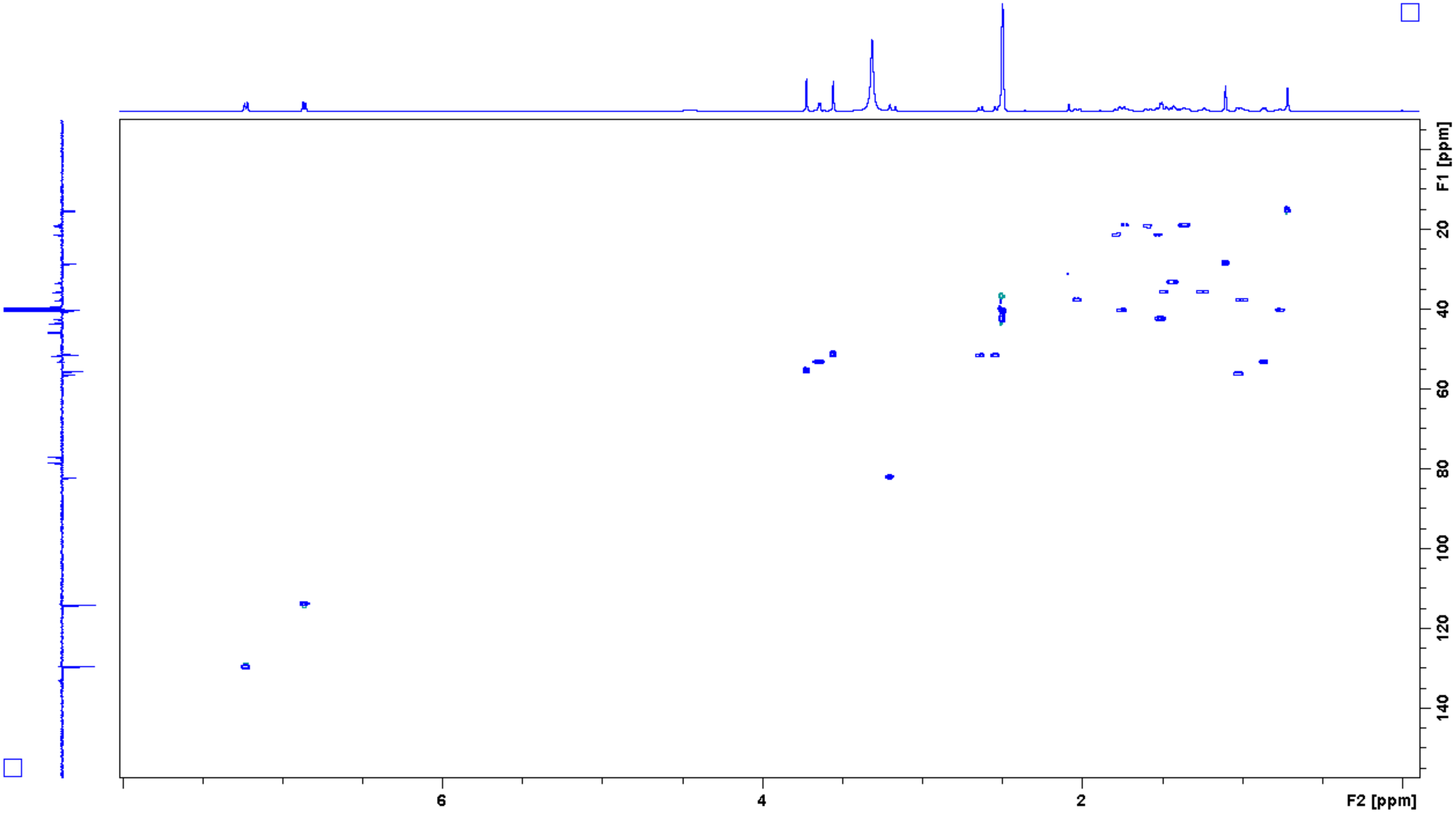

HMBC of compound (4*R*,6a*R*,7*R*,8*R*,9*S*,11b*S*)-methyl 7,8,9-trihydroxy-8-(((4-methoxybenzyl)amino)methyl)-4,11b-dimethyltetradecahydro-6a,9-methanocyclohepta[a]naphthalene-4-carboxylate **(10)**

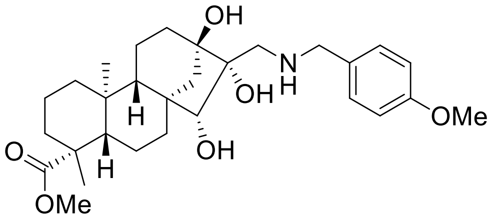

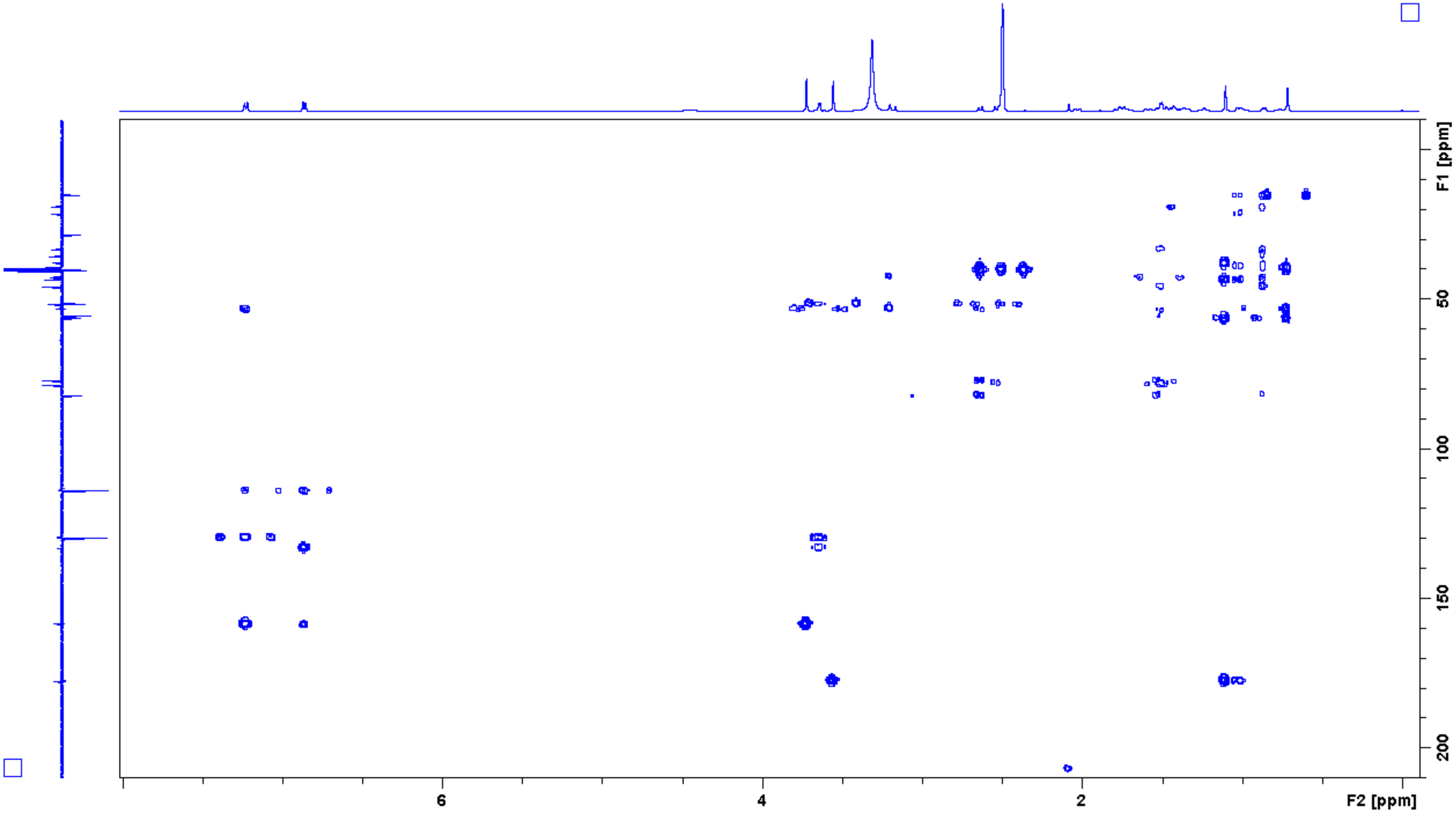

^{1}H-NMR of compound (4*R*,4a*S*,6a*R*,7*R*,8*R*,11a*S*,11b*S*)-methyl 8-((((*R*)-1-(4-fluorophenyl)ethyl)amino)methyl)-7,8,9-trihydroxy-4,11b-dimethyltetradecahydro-6a,9-methanocyclohepta[a]naphthalene-4-carboxylate **(11)**

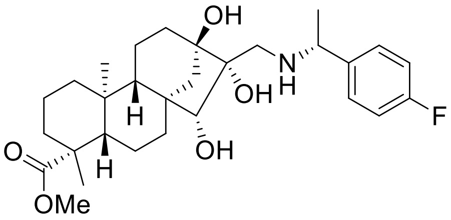

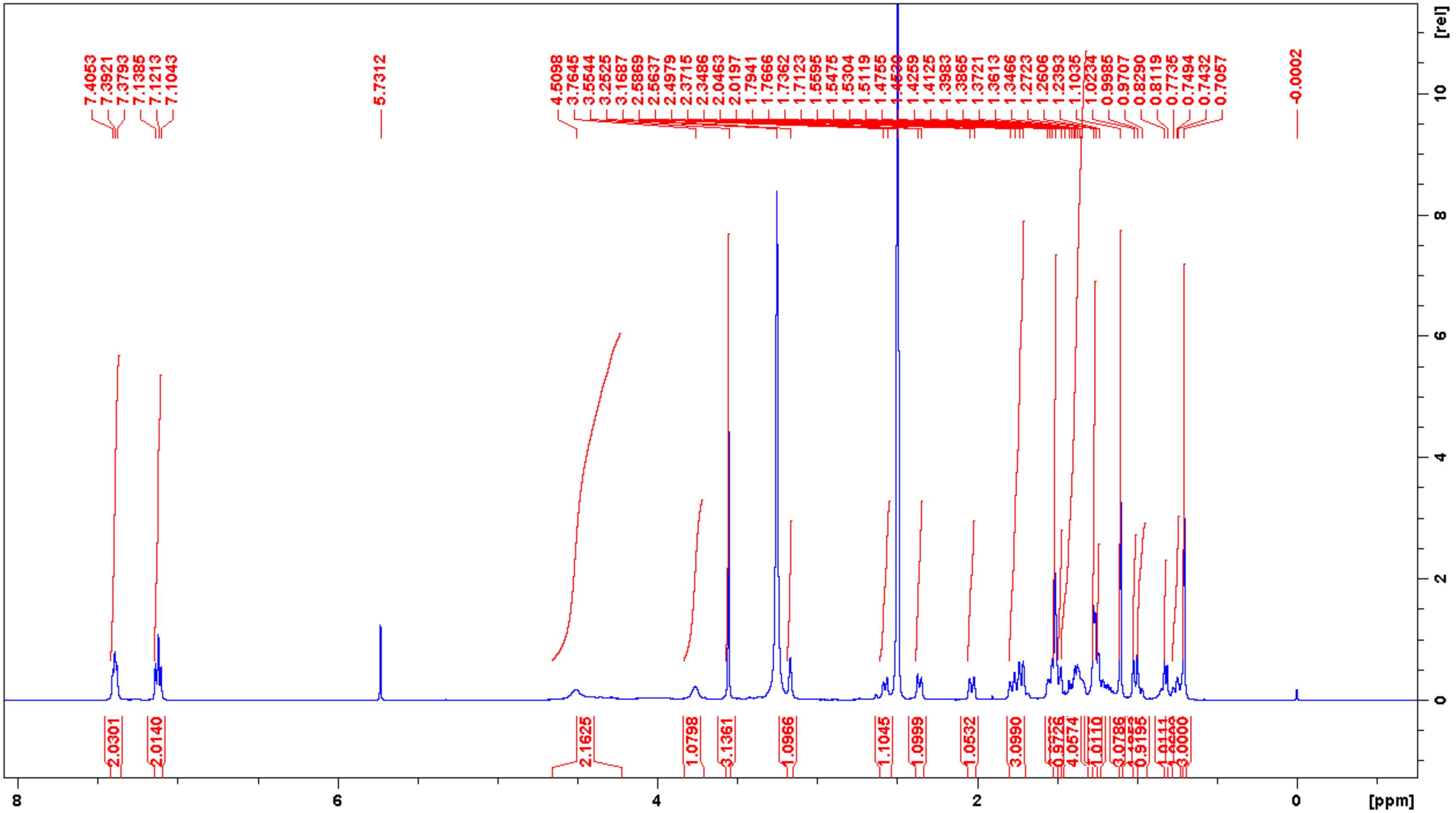

^{13}C-NMR of compound (4*R*,4a*S*,6a*R*,7*R*,8*R*,11a*S*,11b*S*)-methyl 8-((((*R*)-1-(4-fluorophenyl)ethyl)amino)methyl)-7,8,9-trihydroxy-4,11b-dimethyltetradecahydro-6a,9-methanocyclohepta[a]naphthalene-4-carboxylate **(11)**

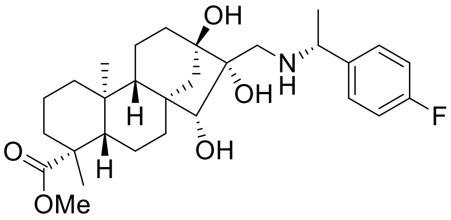

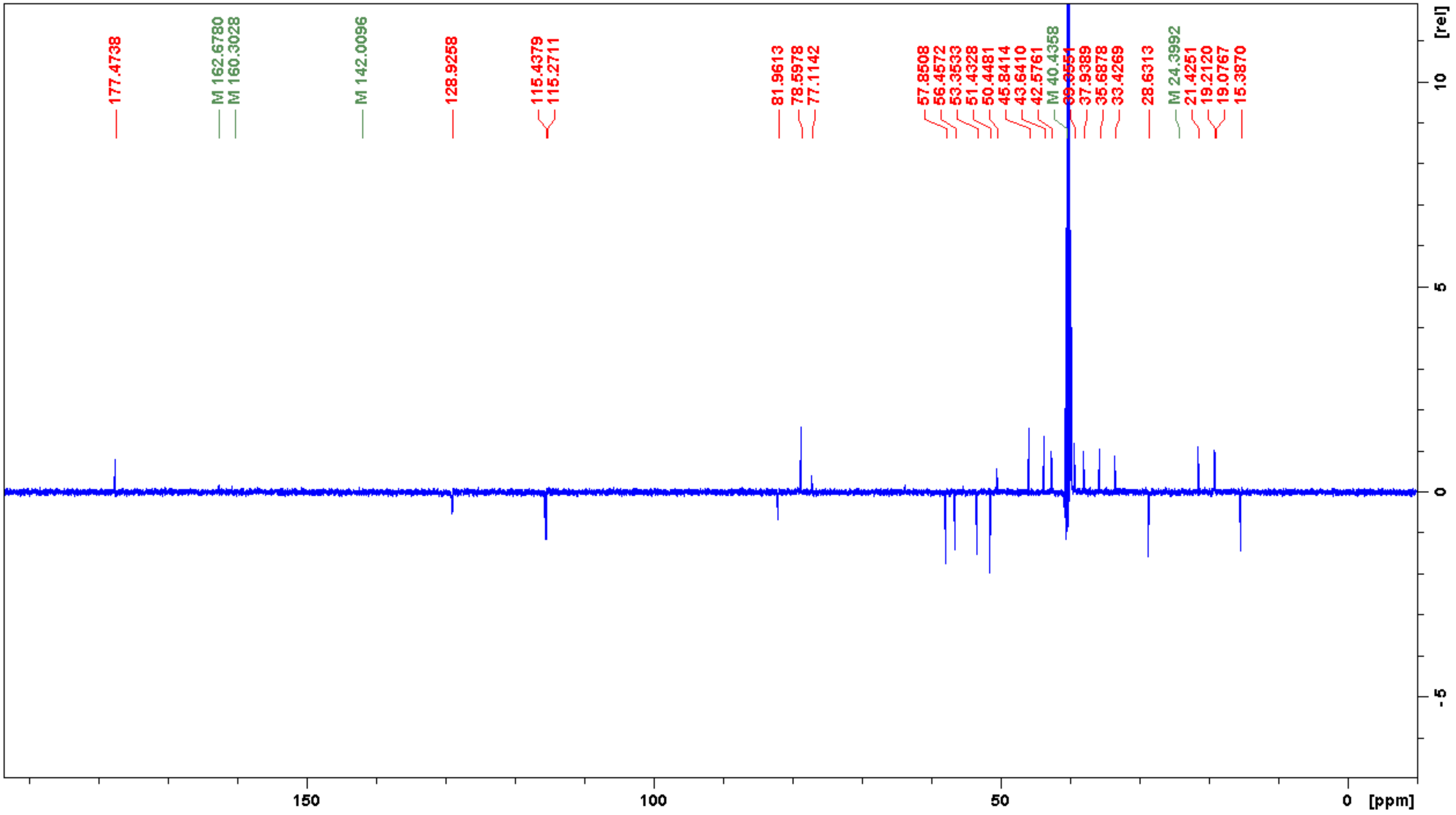

COSY of compound (4*R*,4a*S*,6a*R*,7*R*,8*R*,11a*S*,11b*S*)-methyl 8-((((*R*)-1-(4-fluorophenyl)ethyl)amino)methyl)-7,8,9-trihydroxy-4,11b-dimethyltetradecahydro-6a,9-methanocyclohepta[a]naphthalene-4-carboxylate **(11)**

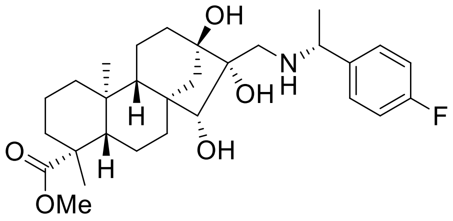

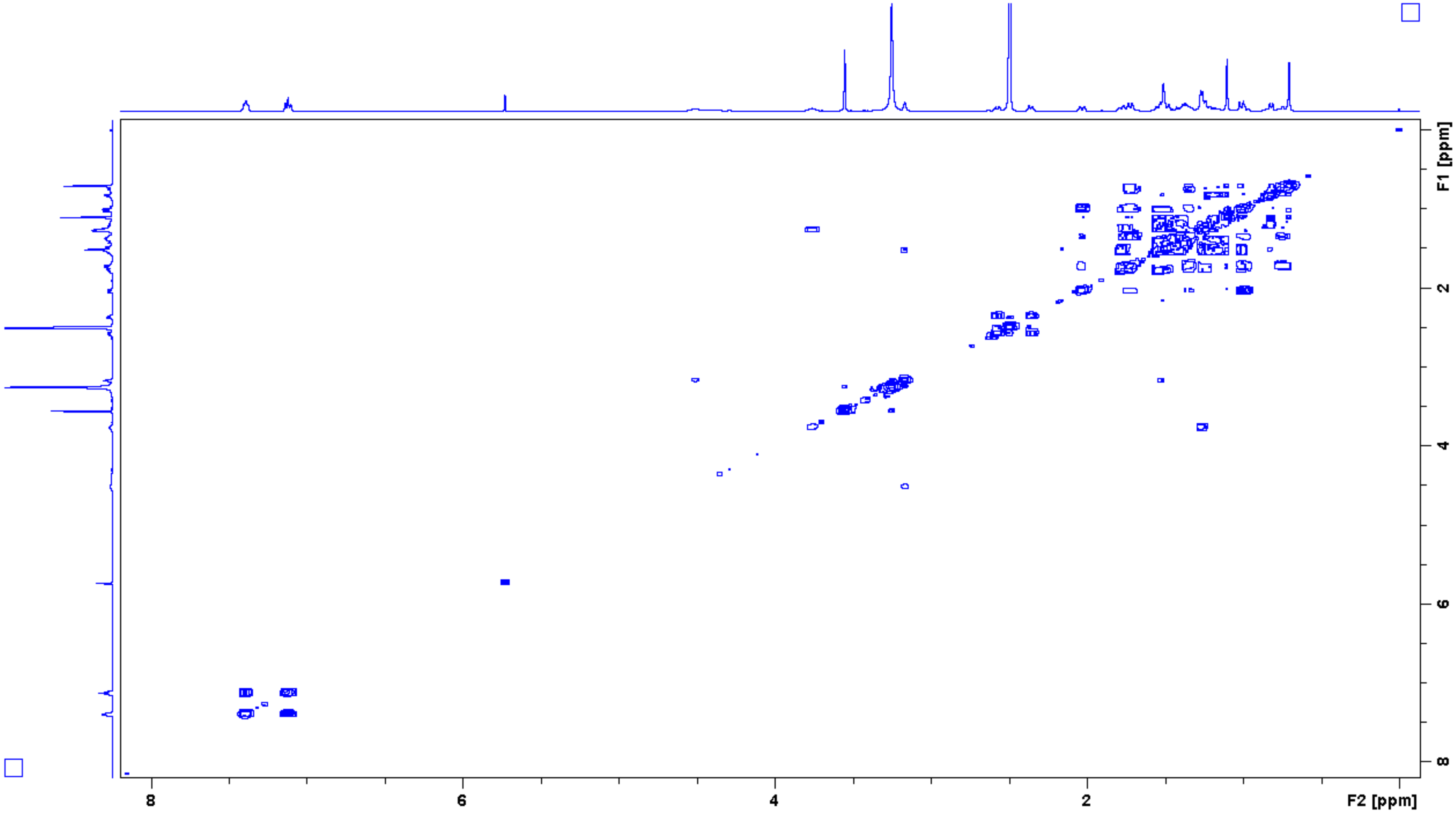

NOESY of compound (4*R*,4a*S*,6a*R*,7*R*,8*R*,11a*S*,11b*S*)-methyl 8-((((*R*)-1-(4-fluorophenyl)ethyl)amino)methyl)-7,8,9-trihydroxy-4,11b-dimethyltetradecahydro-6a,9-methanocyclohepta[a]naphthalene-4-carboxylate **(11)**

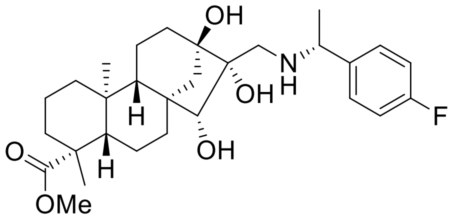

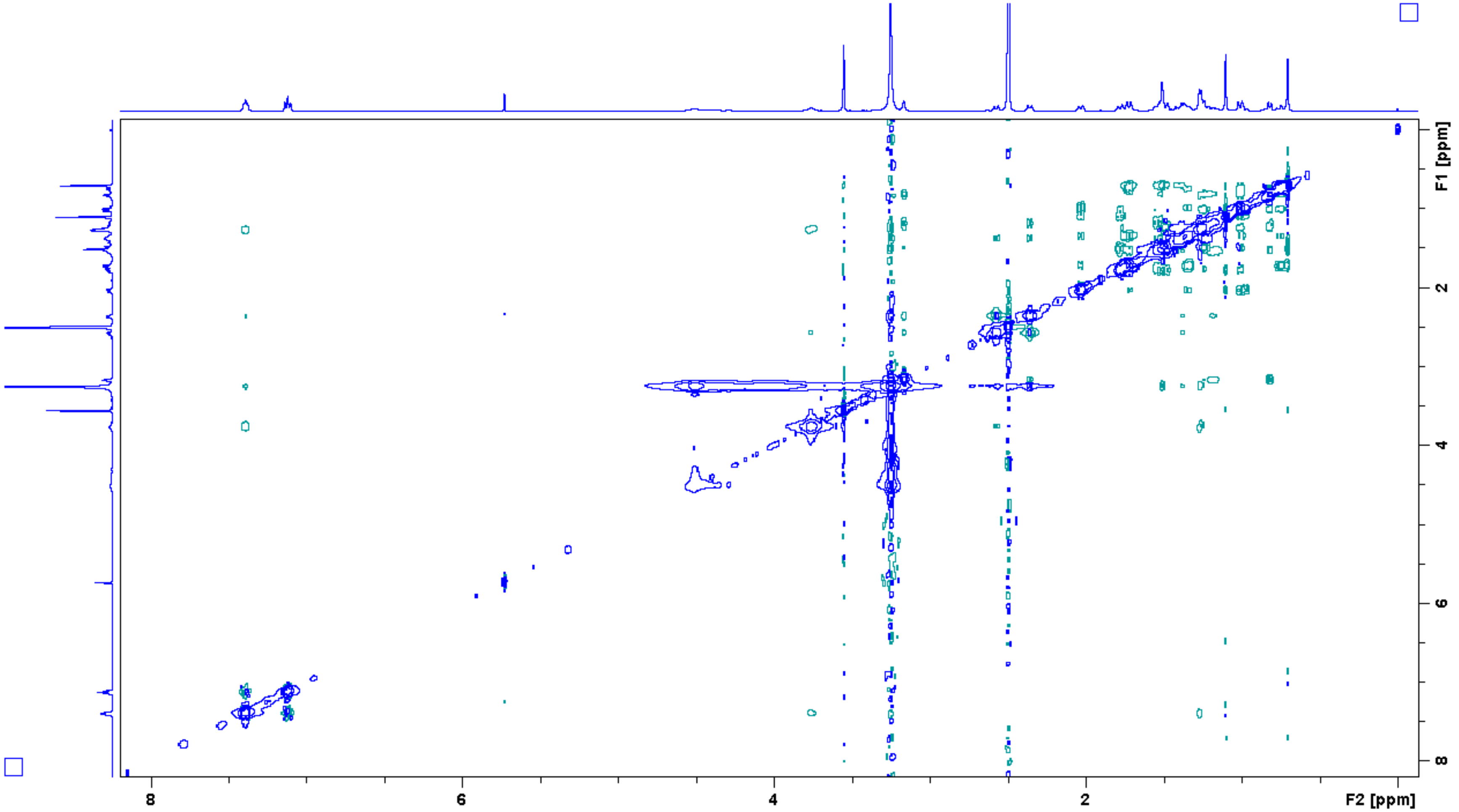

HSQC of compound (4*R*,4a*S*,6a*R*,7*R*,8*R*,11a*S*,11b*S*)-methyl 8-((((*R*)-1-(4-fluorophenyl)ethyl)amino)methyl)-7,8,9-trihydroxy-4,11b-dimethyltetradecahydro-6a,9-methanocyclohepta[a]naphthalene-4-carboxylate **(11)**

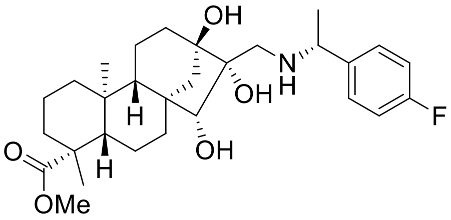

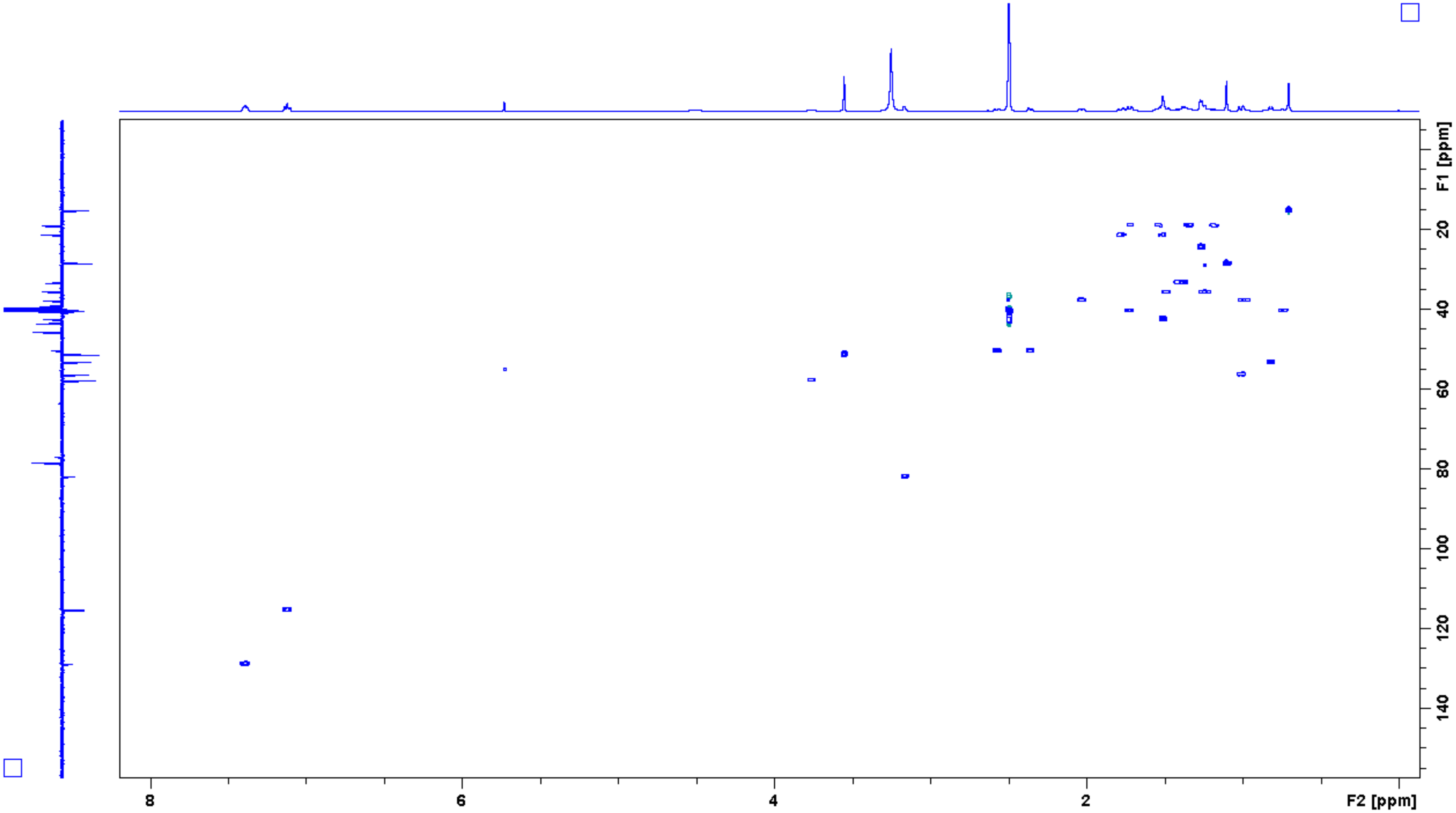

HMBC of compound (4*R*,4a*S*,6a*R*,7*R*,8*R*,11a*S*,11b*S*)-methyl 8-((((*R*)-1-(4-fluorophenyl)ethyl)amino)methyl)-7,8,9-trihydroxy-4,11b-dimethyltetradecahydro-6a,9-methanocyclohepta[a]naphthalene-4-carboxylate **(11)**

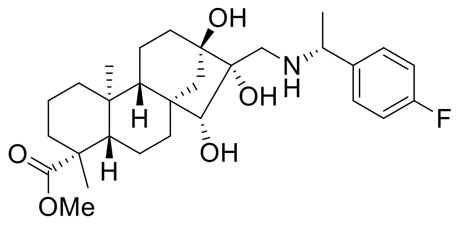

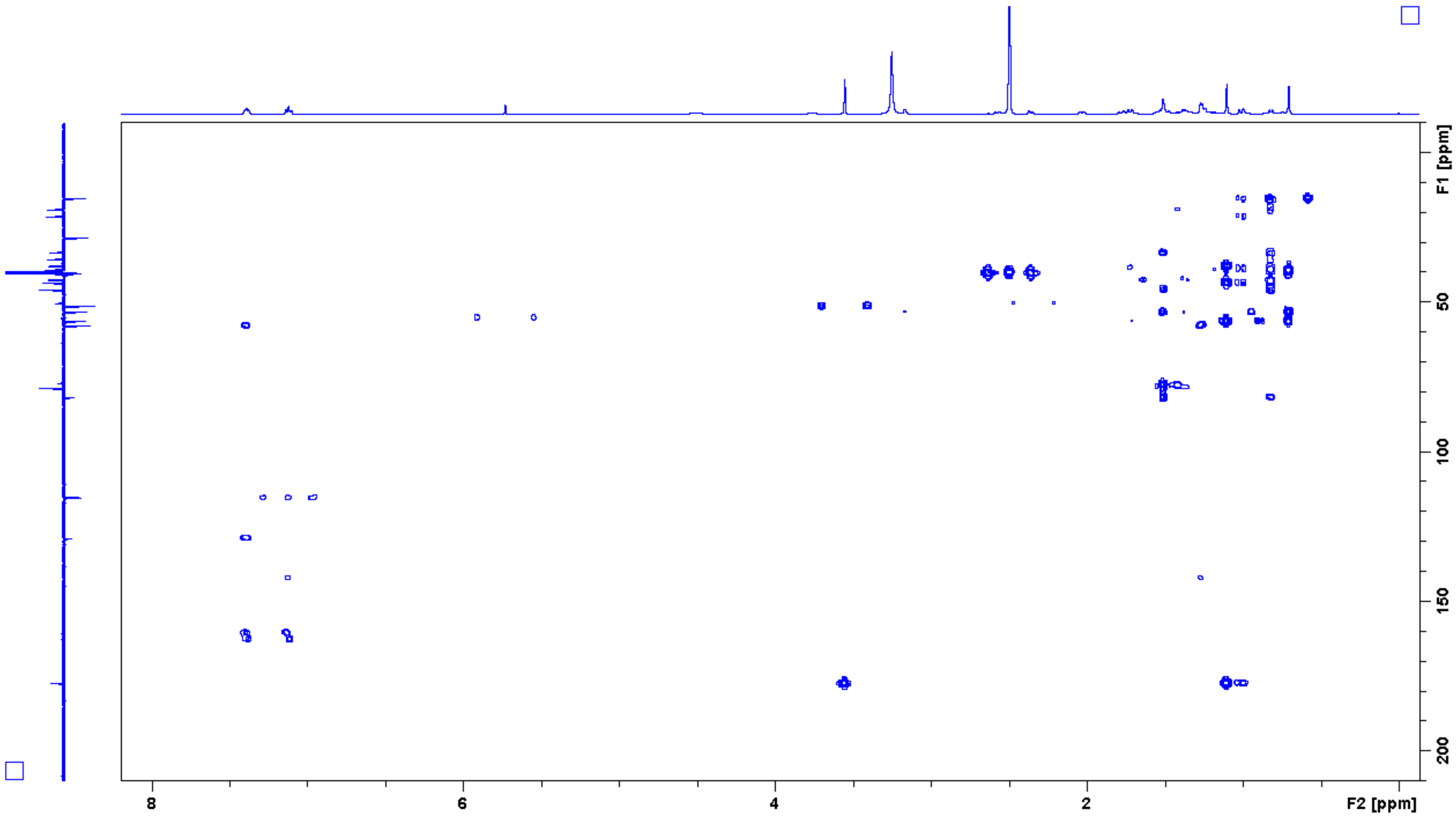

^{19}F-NMR of compound (4*R*,4a*S*,6a*R*,7*R*,8*R*,11a*S*,11b*S*)-methyl 8-((((*R*)-1-(4-fluorophenyl)ethyl)amino)methyl)-7,8,9-trihydroxy-4,11b-dimethyltetradecahydro-6a,9-methanocyclohepta[a]naphthalene-4-carboxylate **(11)**

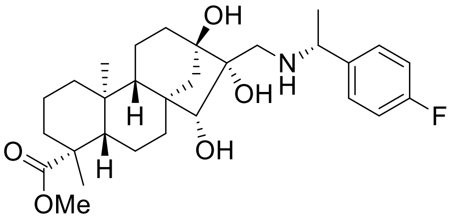

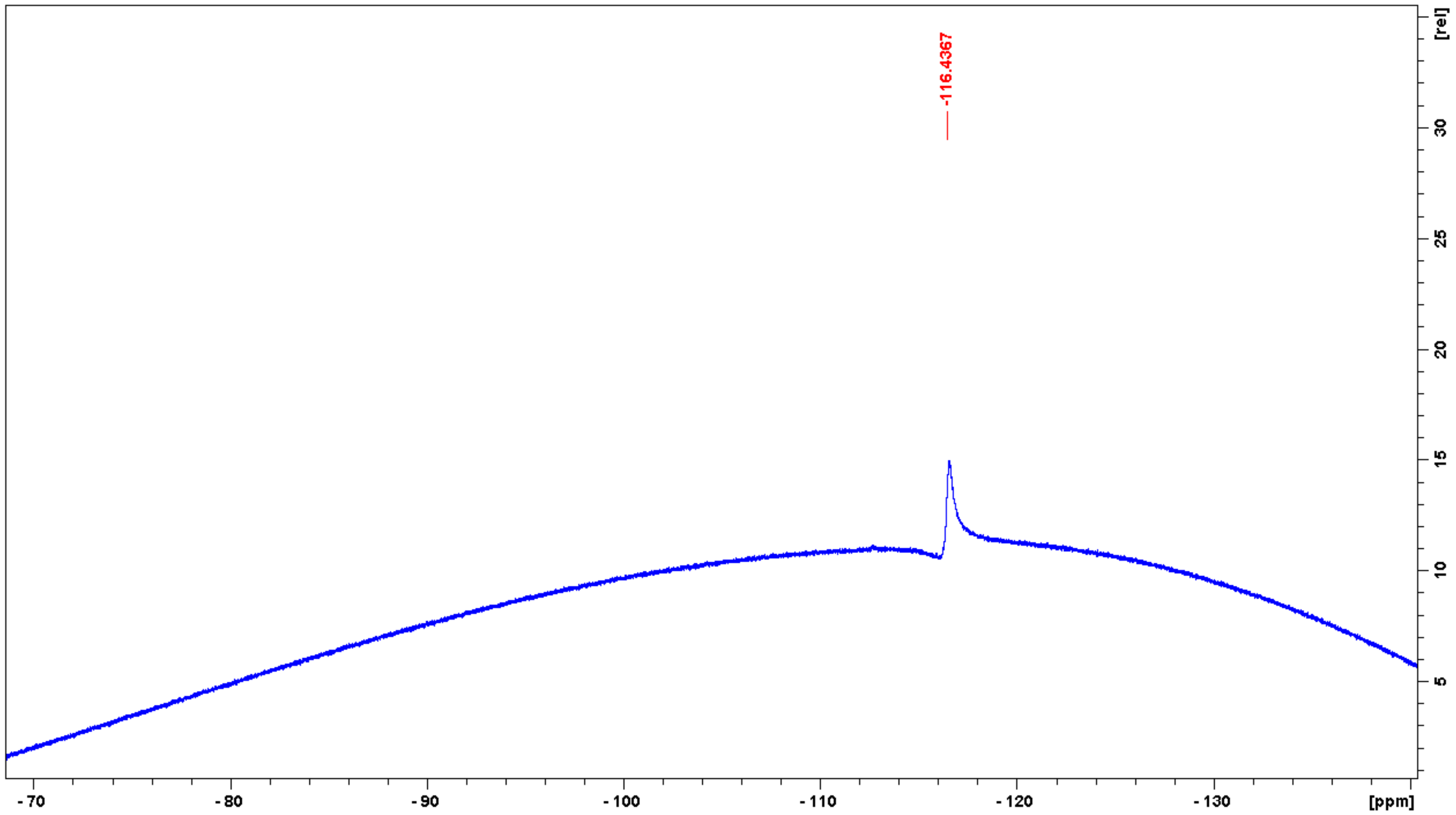

^{1}H-NMR of compound (4*R*,6a*R*,7*R*,8*R*,9*S*,11b*S*)-methyl 7,8,9-trihydroxy-4,11b-dimethyl-8-((((*R*)-1-(naphthalen-2-yl)ethyl)amino)methyl)tetradecahydro-6a,9-methanocyclohepta[a]naphthalene-4-carboxylate **(12)**

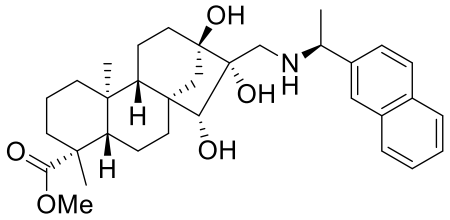

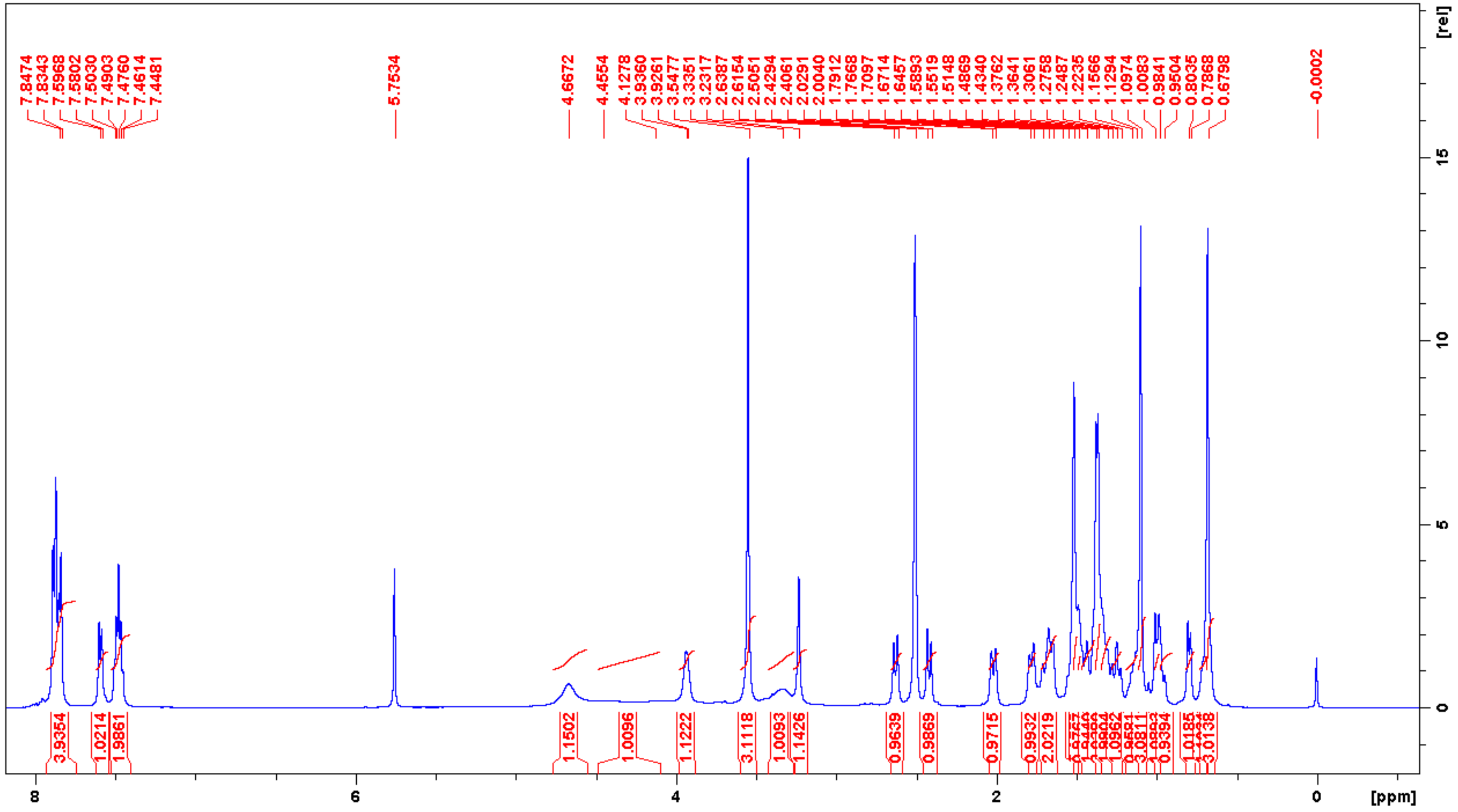

^{13}C-NMR of compound (4*R*,6a*R*,7*R*,8*R*,9*S*,11b*S*)-methyl 7,8,9-trihydroxy-4,11b-dimethyl-8-((((*R*)-1-(naphthalen-2-yl)ethyl)amino)methyl)tetradecahydro-6a,9-methanocyclohepta[a]naphthalene-4-carboxylate **(12)**

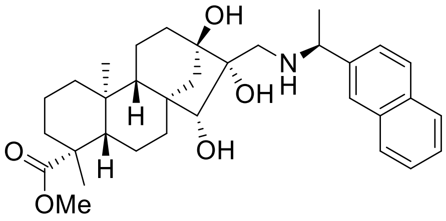

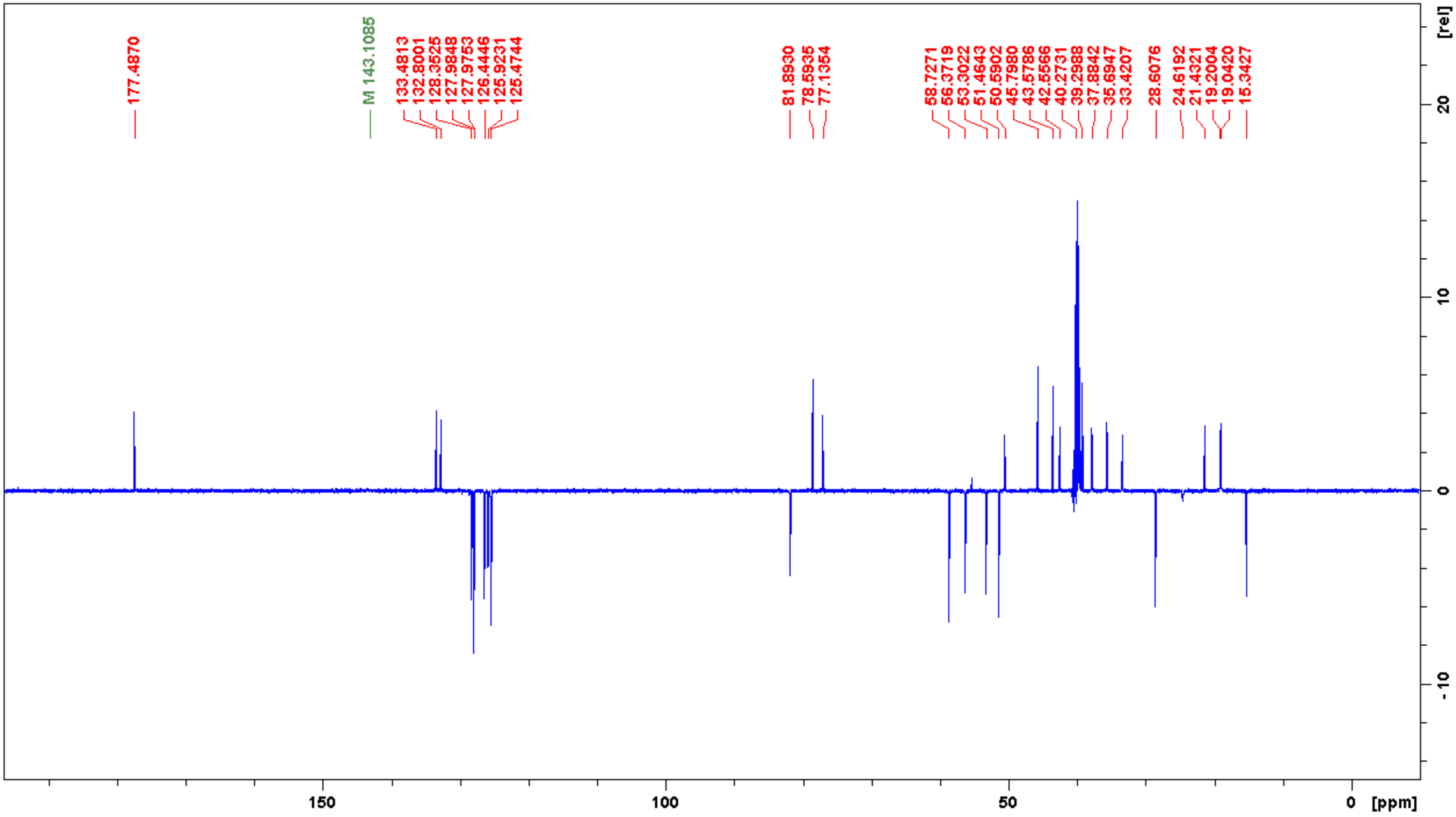

COSY of compound (4*R*,6a*R*,7*R*,8*R*,9*S*,11b*S*)-methyl 7,8,9-trihydroxy-4,11b-dimethyl-8-((((*R*)-1-(naphthalen-2-yl)ethyl)amino)methyl)tetradecahydro-6a,9-methanocyclohepta[a]naphthalene-4-carboxylate **(12)**

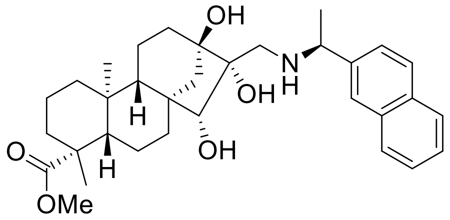

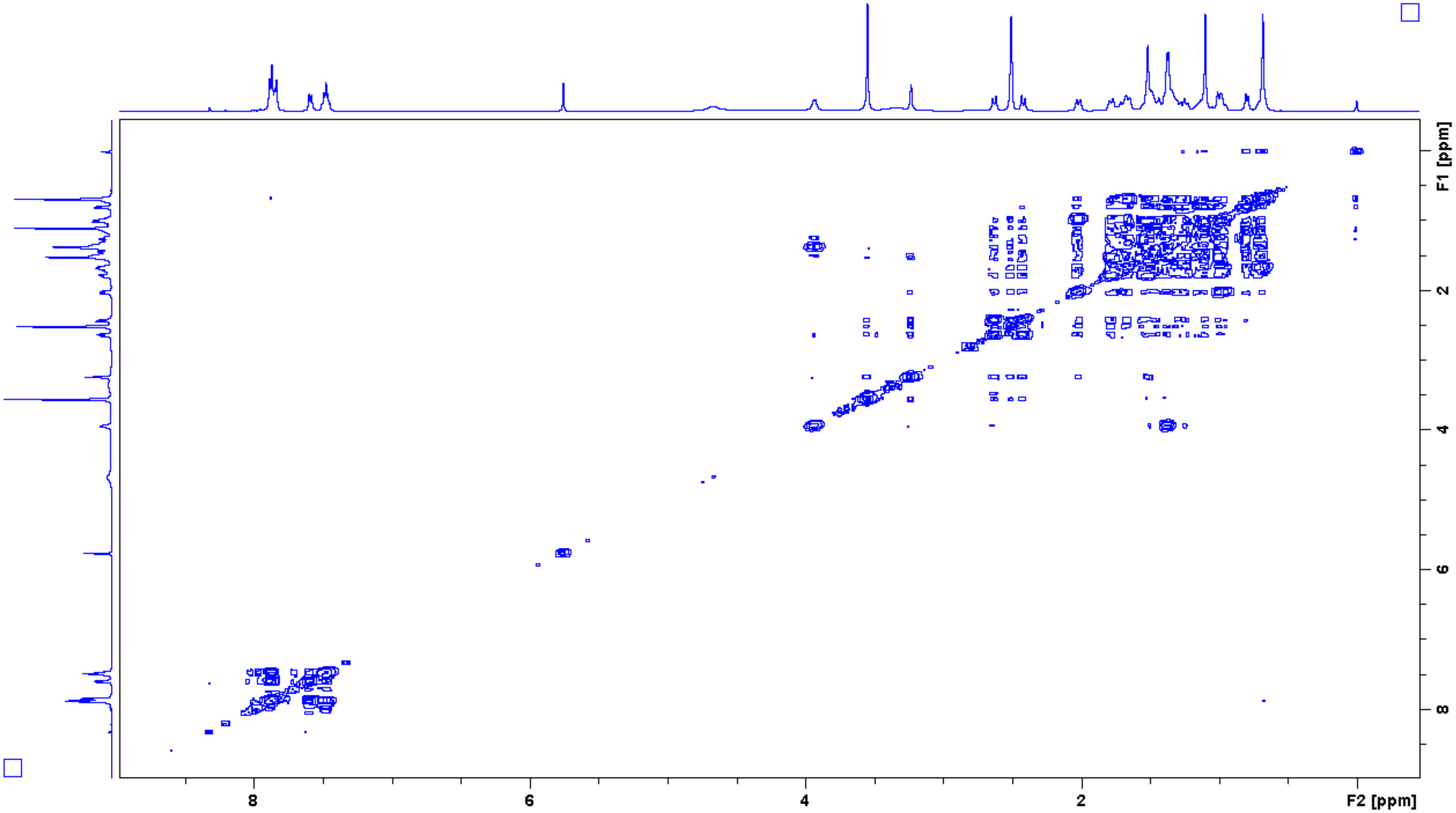

NOESY of compound (4*R*,6a*R*,7*R*,8*R*,9*S*,11b*S*)-methyl 7,8,9-trihydroxy-4,11b-dimethyl-8-((((*R*)-1-(naphthalen-2-yl)ethyl)amino)methyl)tetradecahydro-6a,9-methanocyclohepta[a]naphthalene-4-carboxylate **(12)**

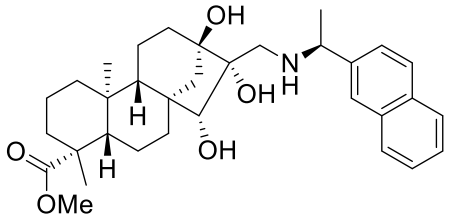

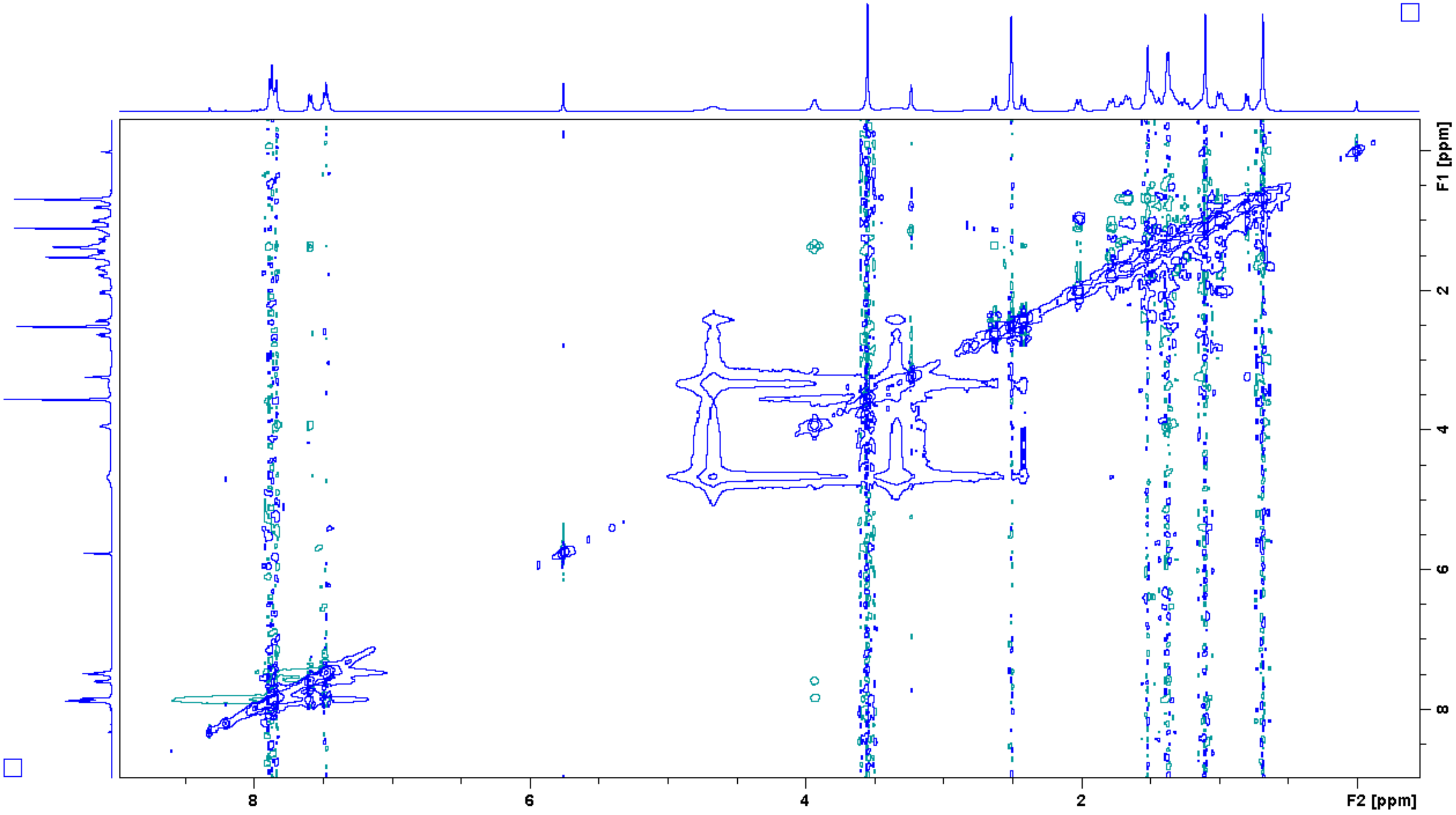

HSQC of compound (4*R*,6a*R*,7*R*,8*R*,9*S*,11b*S*)-methyl 7,8,9-trihydroxy-4,11b-dimethyl-8-((((*R*)-1-(naphthalen-2-yl)ethyl)amino)methyl)tetradecahydro-6a,9-methanocyclohepta[a]naphthalene-4-carboxylate **(12)**

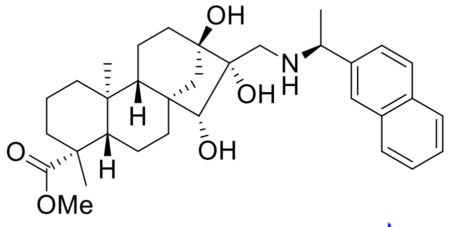

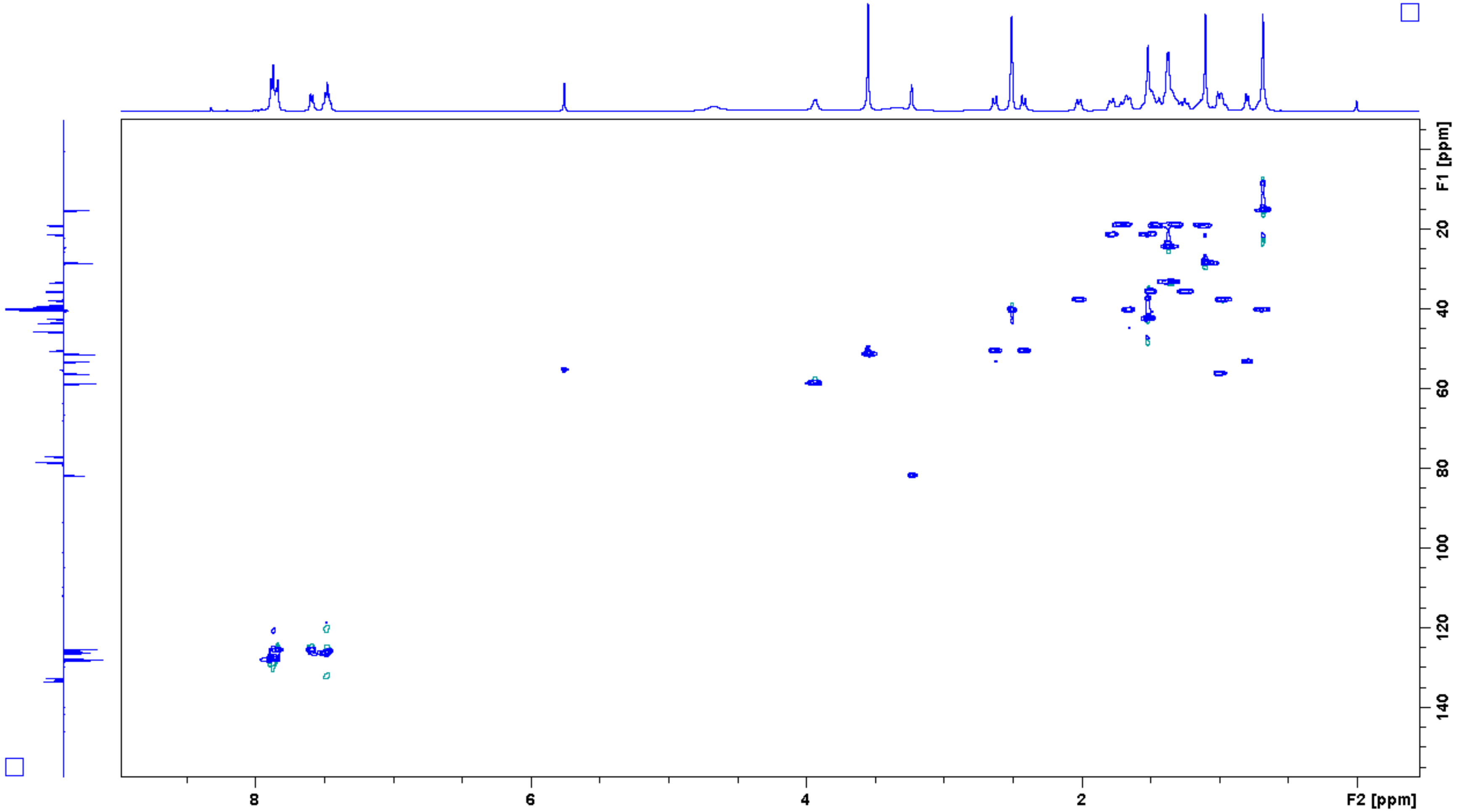

HMBC of compound (4*R*,6a*R*,7*R*,8*R*,9*S*,11b*S*)-methyl 7,8,9-trihydroxy-4,11b-dimethyl-8-((((*R*)-1-(naphthalen-2-yl)ethyl)amino)methyl)tetradecahydro-6a,9-methanocyclohepta[a]naphthalene-4-carboxylate **(12)**

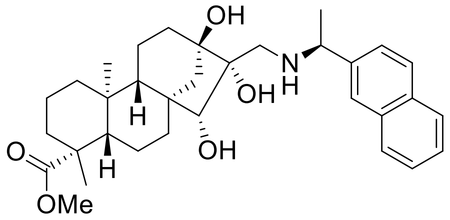

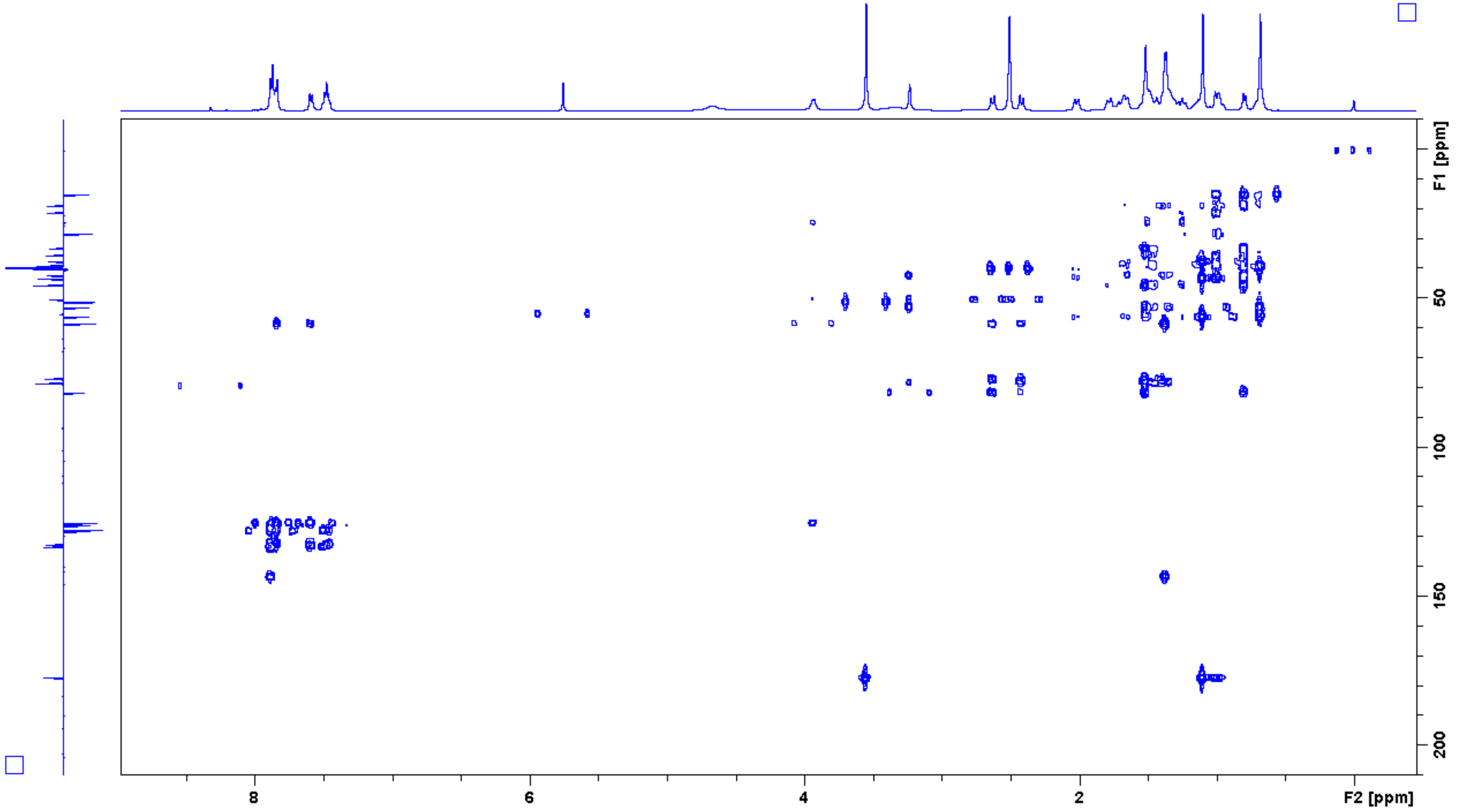

^{1}H-NMR of compound (4*R*,6a*R*,7*R*,8*R*,9*S*,11b*S*)-methyl 7,8,9-trihydroxy-4,11b-dimethyl-8-((((*S*)-1-(naphthalen-2-yl)ethyl)amino)methyl)tetradecahydro-6a,9-methanocyclohepta[a]naphthalene-4-carboxylate **(13)**

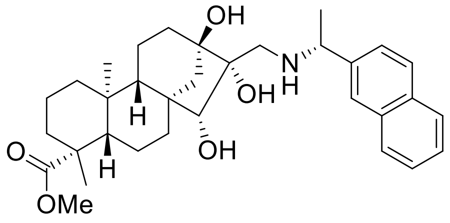

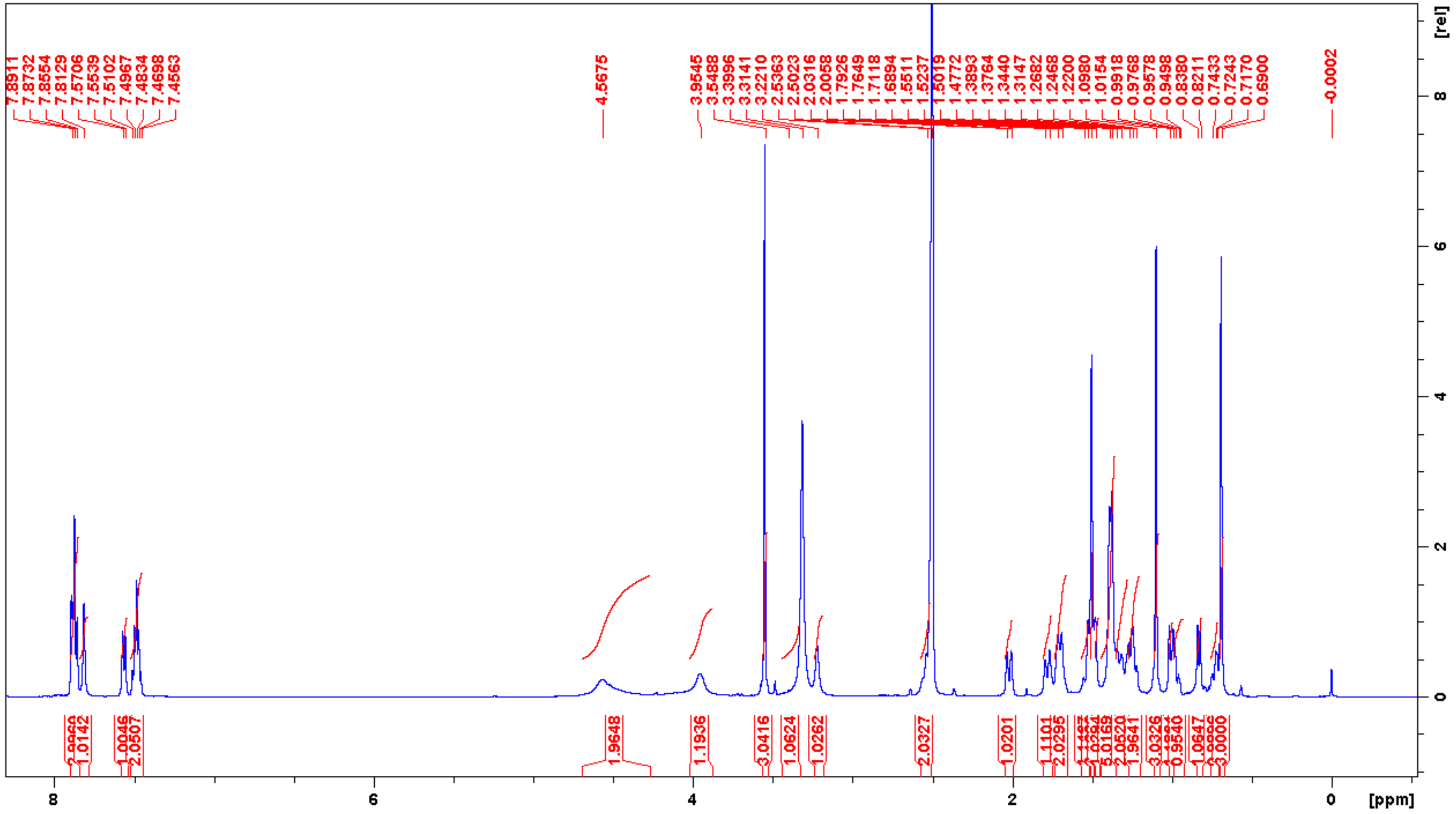

^{13}C-NMR of compound (4*R*,6a*R*,7*R*,8*R*,9*S*,11b*S*)-methyl 7,8,9-trihydroxy-4,11b-dimethyl-8-((((*S*)-1-(naphthalen-2-yl)ethyl)amino)methyl)tetradecahydro-6a,9-methanocyclohepta[a]naphthalene-4-carboxylate **(13)**

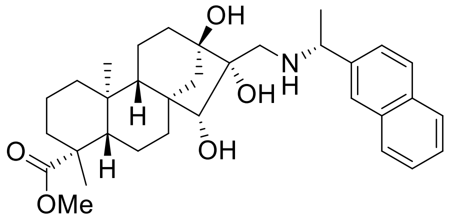

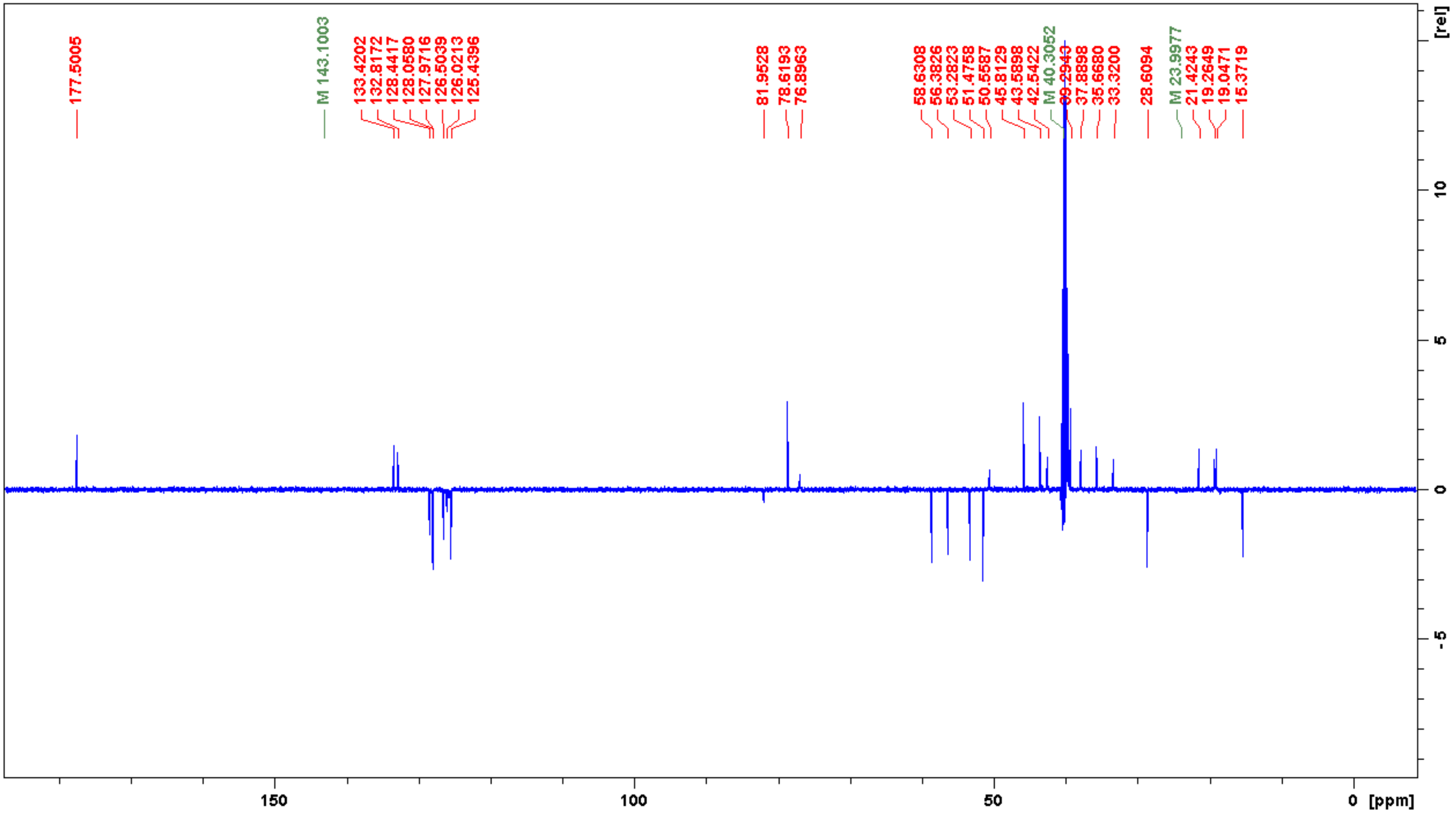

COSY of compound (4*R*,6a*R*,7*R*,8*R*,9*S*,11b*S*)-methyl 7,8,9-trihydroxy-4,11b-dimethyl-8-((((*S*)-1-(naphthalen-2-yl)ethyl)amino)methyl)tetradecahydro-6a,9-methanocyclohepta[a]naphthalene-4-carboxylate **(13)**

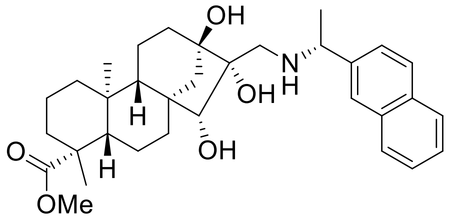

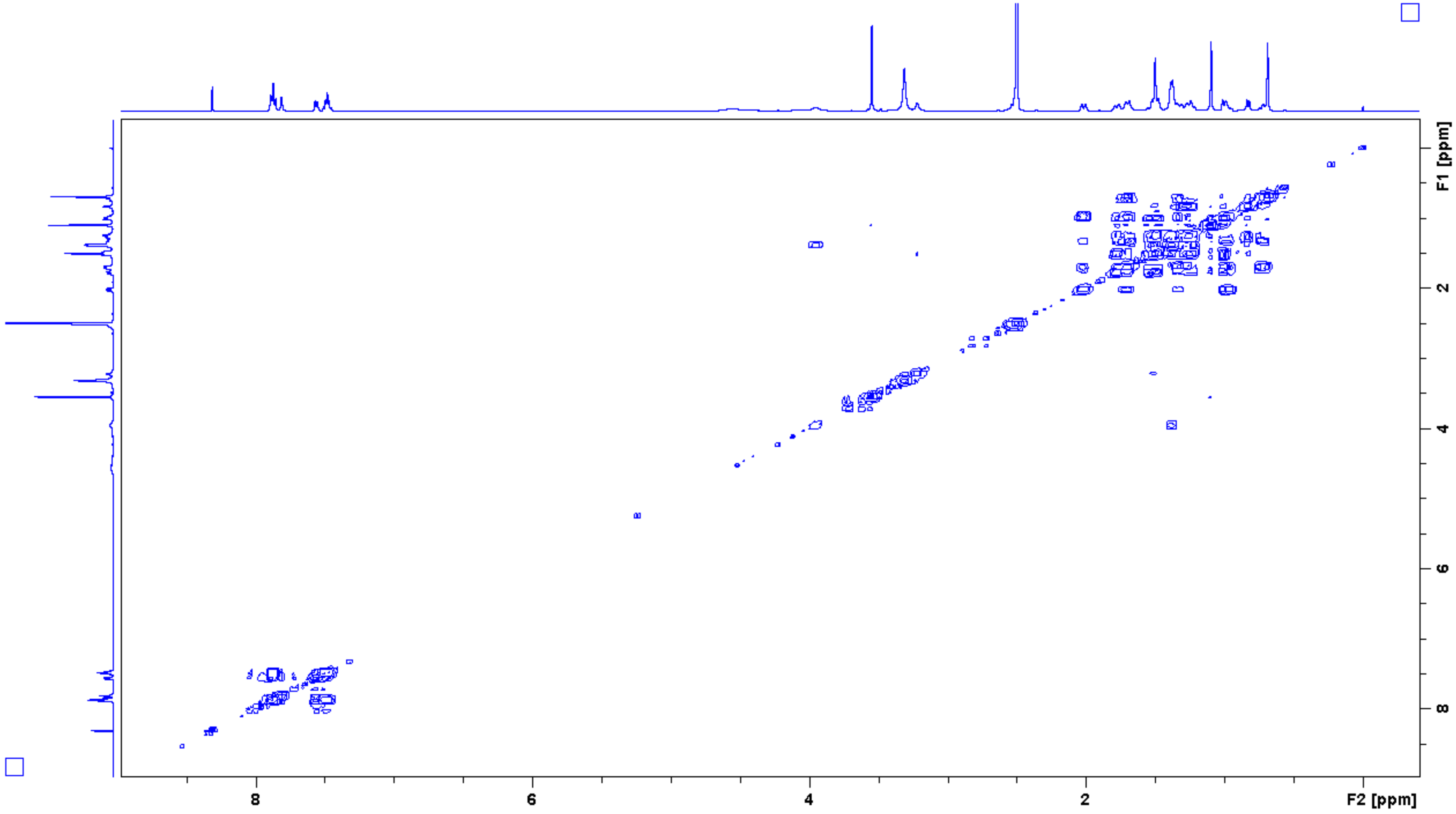

NOESY of compound (4*R*,6a*R*,7*R*,8*R*,9*S*,11b*S*)-methyl 7,8,9-trihydroxy-4,11b-dimethyl-8-((((*S*)-1-(naphthalen-2-yl)ethyl)amino)methyl)tetradecahydro-6a,9-methanocyclohepta[a]naphthalene-4-carboxylate **(13)**

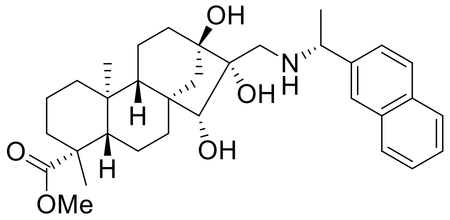

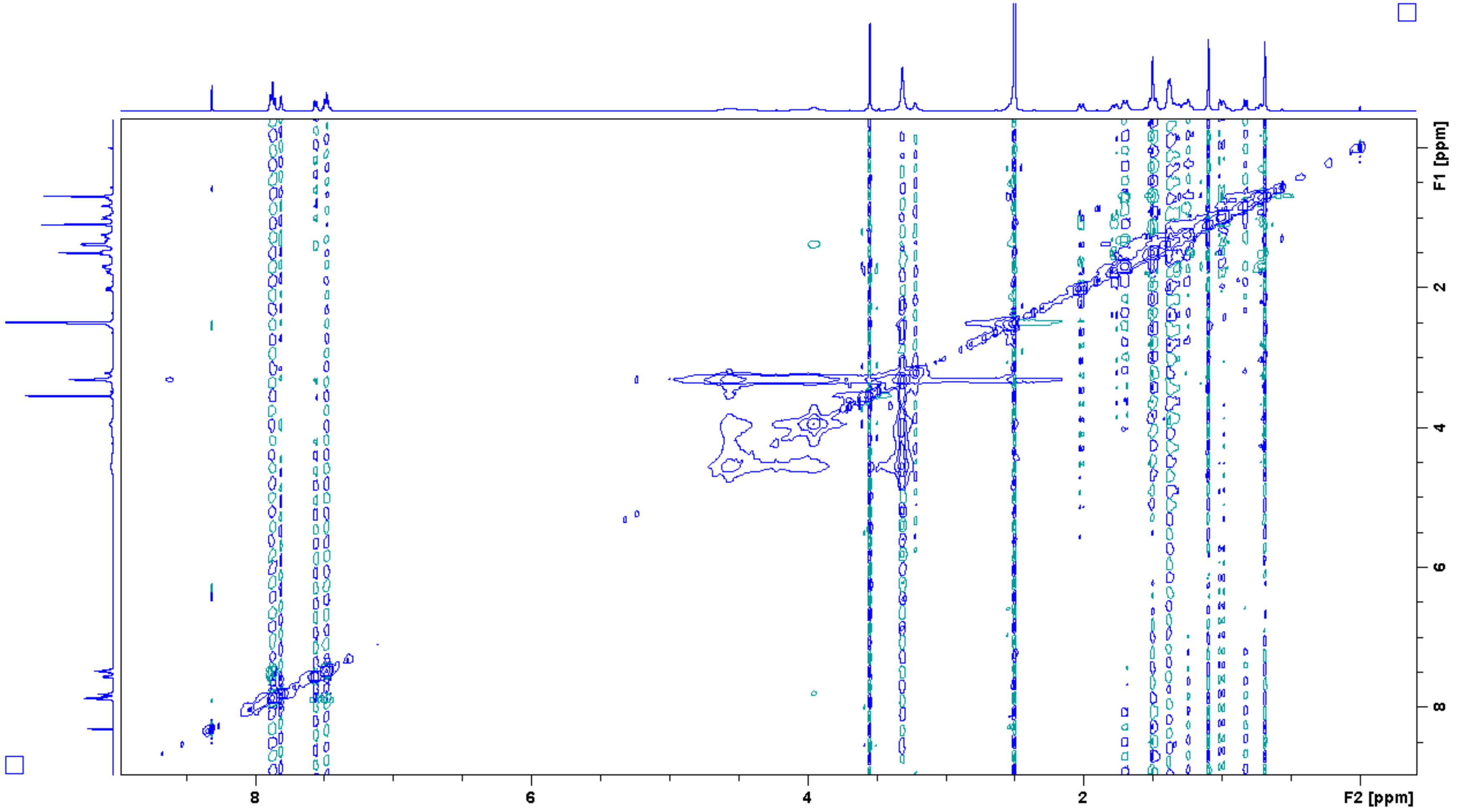

HSQC of compound (4*R*,6a*R*,7*R*,8*R*,9*S*,11b*S*)-methyl 7,8,9-trihydroxy-4,11b-dimethyl-8-((((*S*)-1-(naphthalen-2-yl)ethyl)amino)methyl)tetradecahydro-6a,9-methanocyclohepta[a]naphthalene-4-carboxylate **(13)**

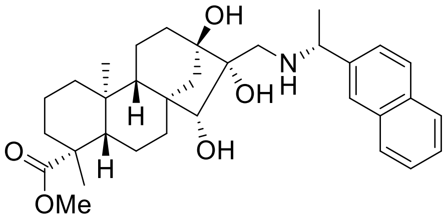

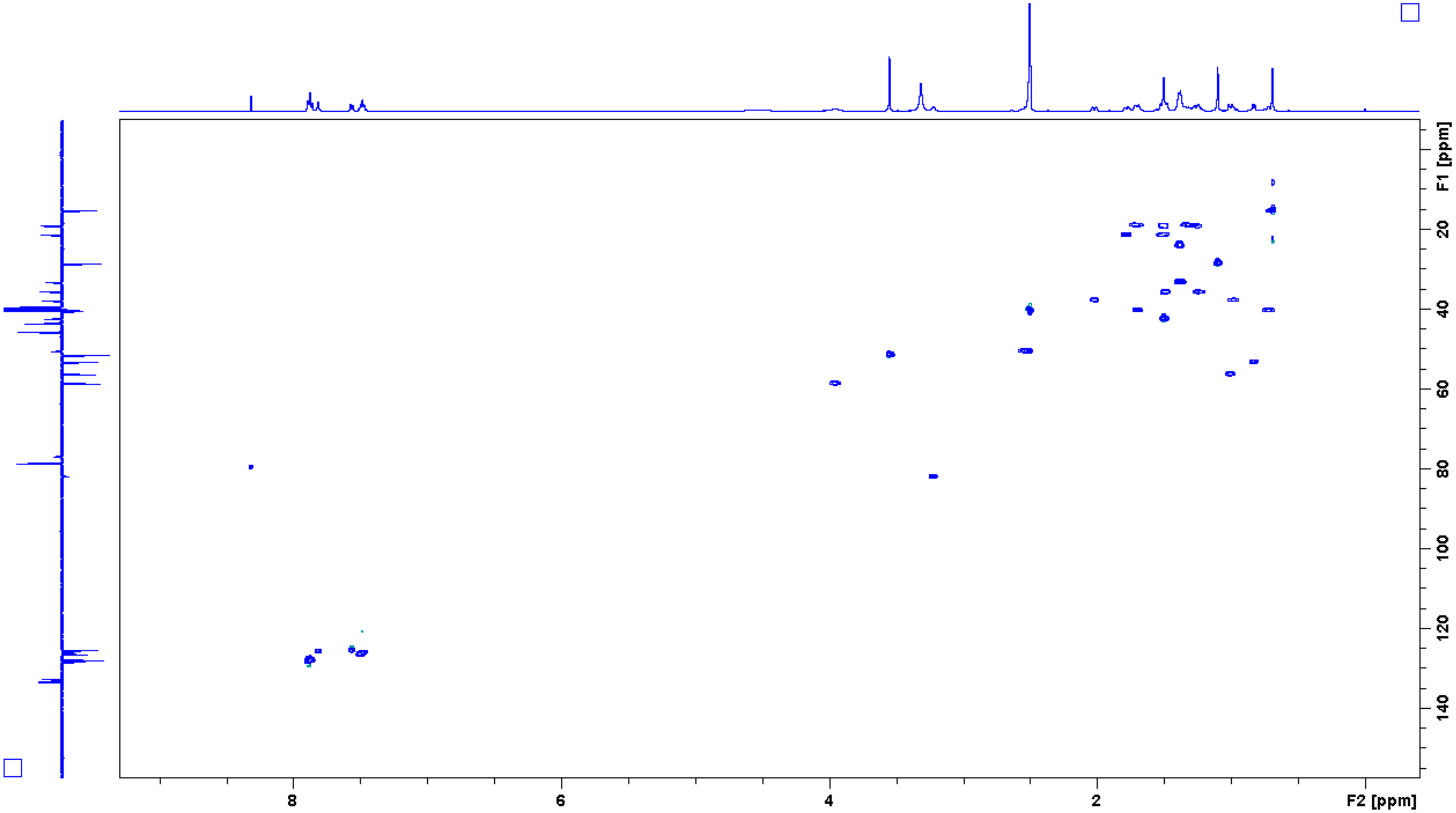

HMBC of compound (4*R*,6a*R*,7*R*,8*R*,9*S*,11b*S*)-methyl 7,8,9-trihydroxy-4,11b-dimethyl-8-((((*S*)-1-(naphthalen-2-yl)ethyl)amino)methyl)tetradecahydro-6a,9-methanocyclohepta[a]naphthalene-4-carboxylate **(13)**

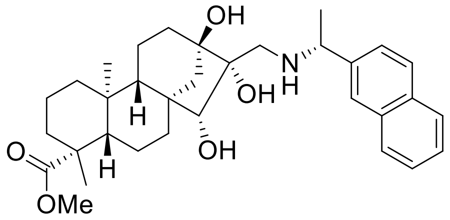

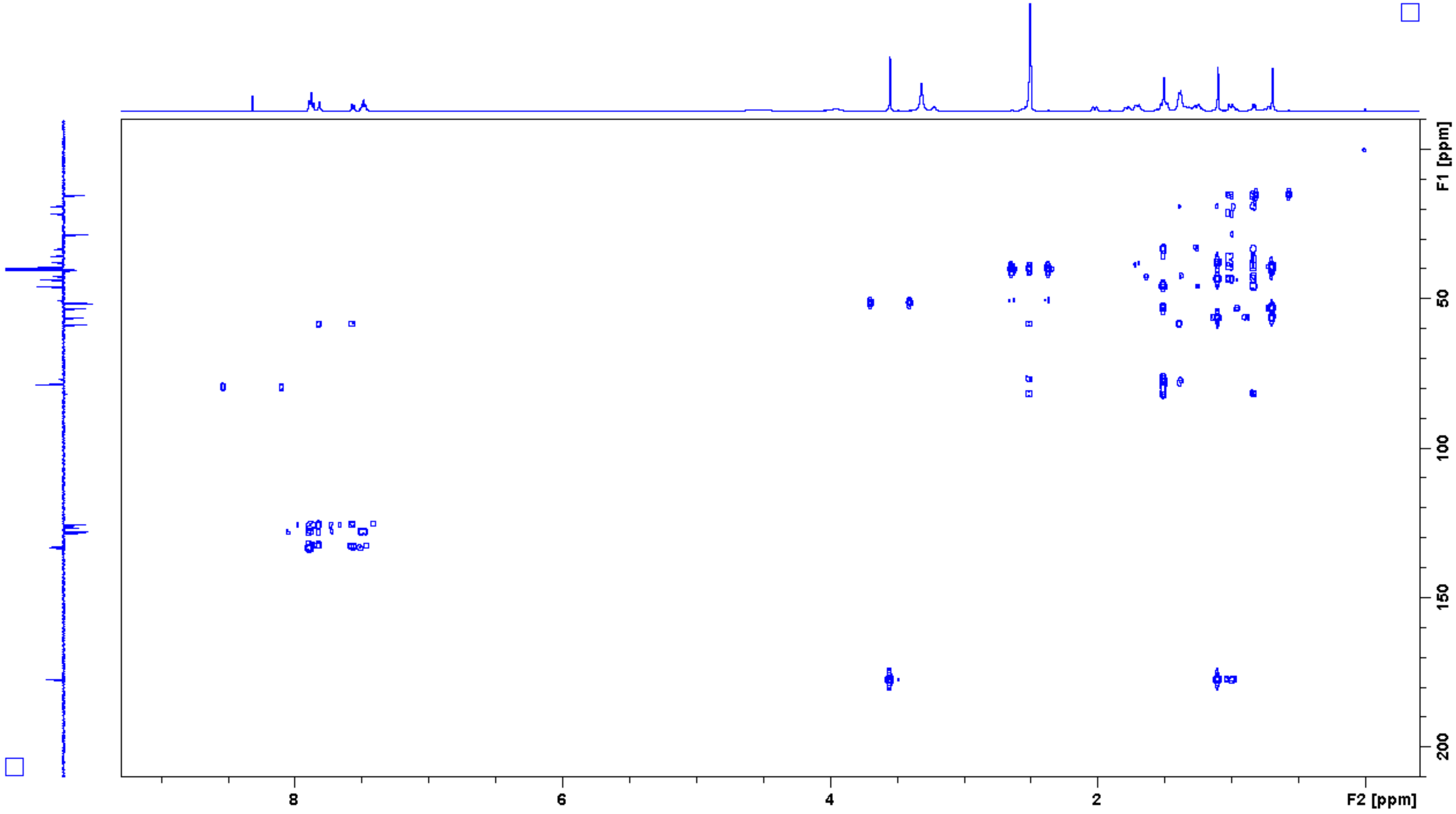

^{1}H-NMR of compound (4*R*,6a*R*,7*R*,8*R*,9*S*,11b*S*)-methyl 7,8,9-trihydroxy-4,11b-dimethyl-8-(((naphthalen-1-ylmethyl)amino)methyl)tetradecahydro-6a,9-methanocyclohepta[a]naphthalene-4-carboxylate **(14)**

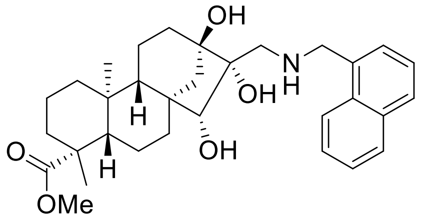

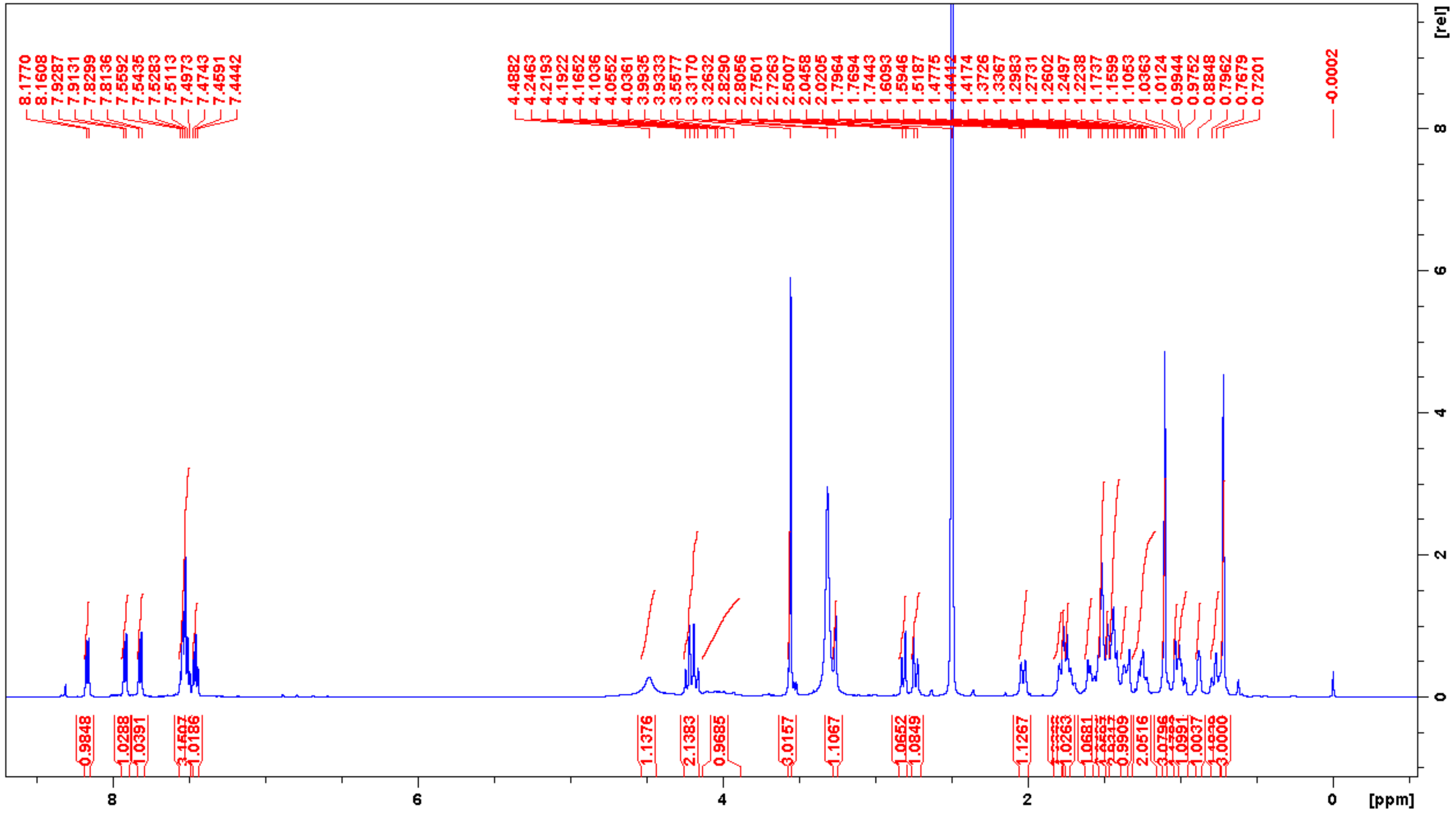

^{13}C-NMR of compound (4*R*,6a*R*,7*R*,8*R*,9*S*,11b*S*)-methyl 7,8,9-trihydroxy-4,11b-dimethyl-8-(((naphthalen-1-ylmethyl)amino)methyl)tetradecahydro-6a,9-methanocyclohepta[a]naphthalene-4-carboxylate **(14)**

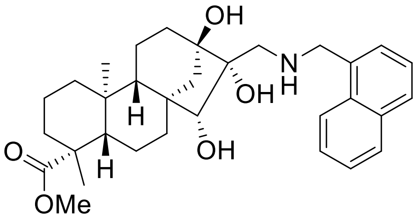

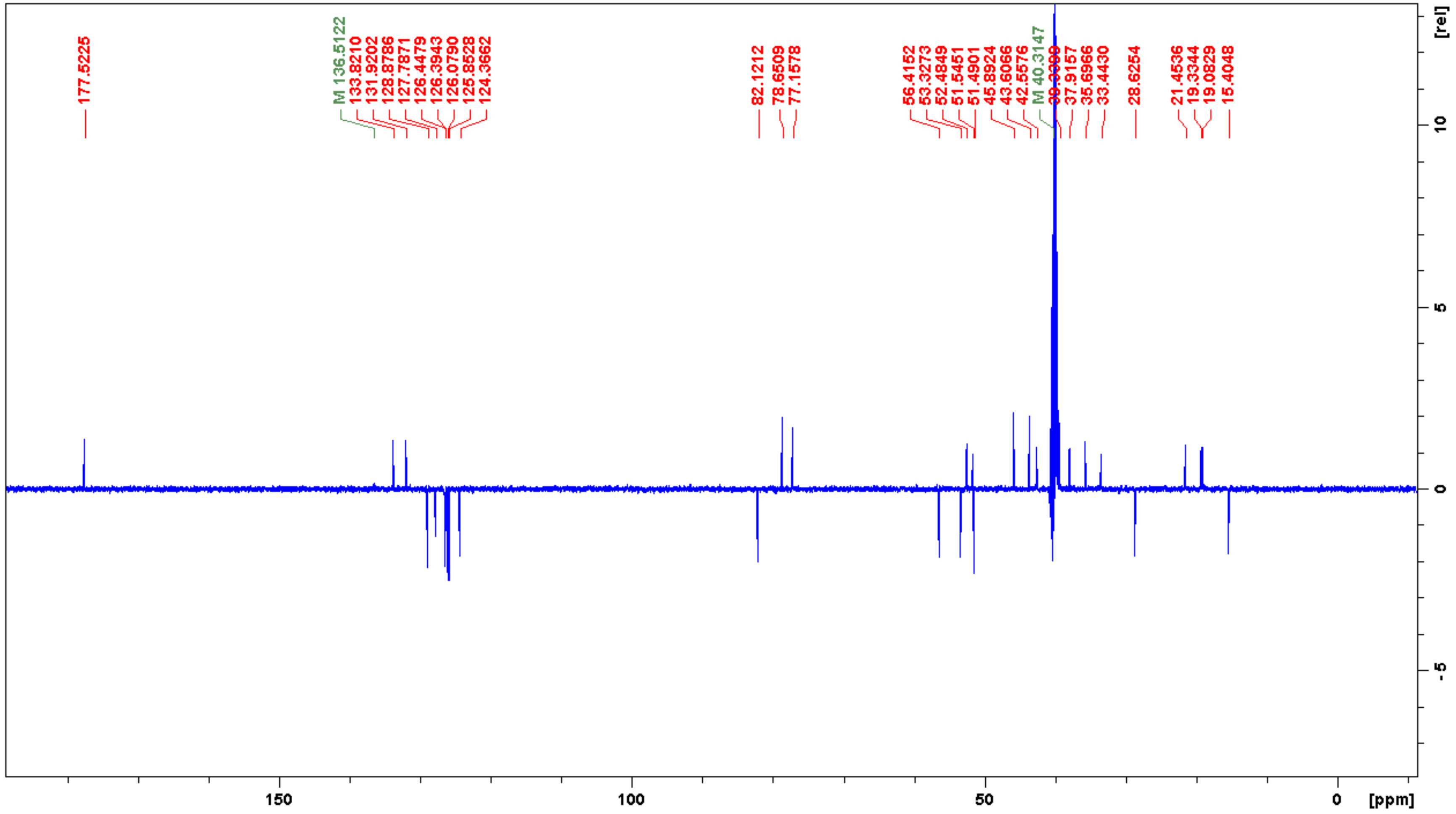

COSY of compound (4*R*,6a*R*,7*R*,8*R*,9*S*,11b*S*)-methyl 7,8,9-trihydroxy-4,11b-dimethyl-8-(((naphthalen-1-ylmethyl)amino)methyl)tetradecahydro-6a,9-methanocyclohepta[a]naphthalene-4-carboxylate **(14)**

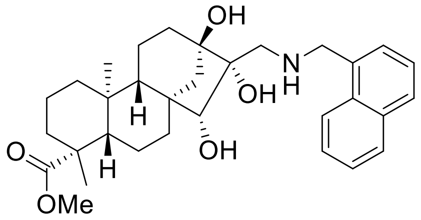

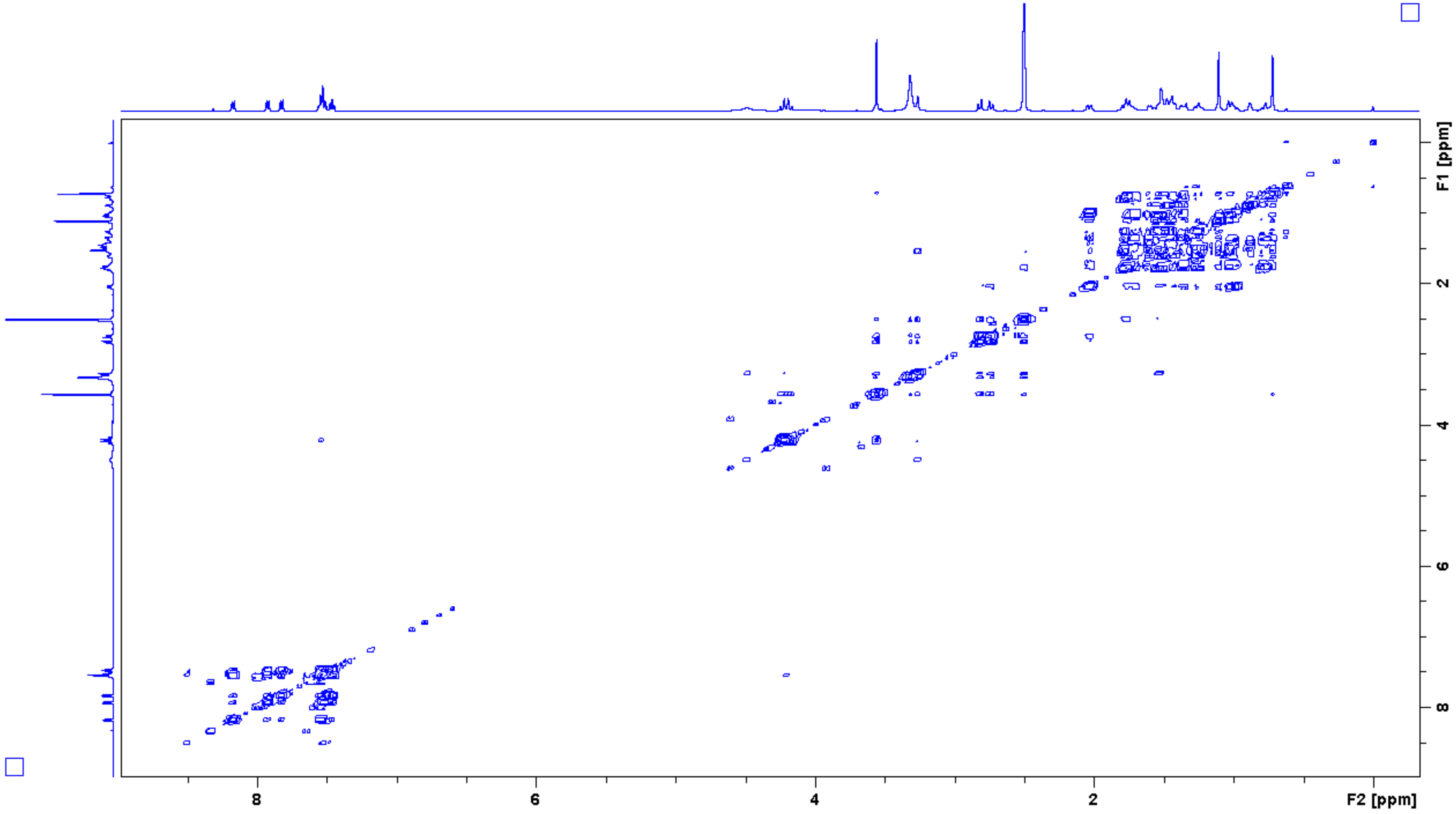

NOESY of compound (4*R*,6a*R*,7*R*,8*R*,9*S*,11b*S*)-methyl 7,8,9-trihydroxy-4,11b-dimethyl-8-(((naphthalen-1-ylmethyl)amino)methyl)tetradecahydro-6a,9-methanocyclohepta[a]naphthalene-4-carboxylate **(14)**

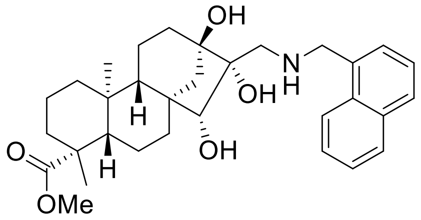

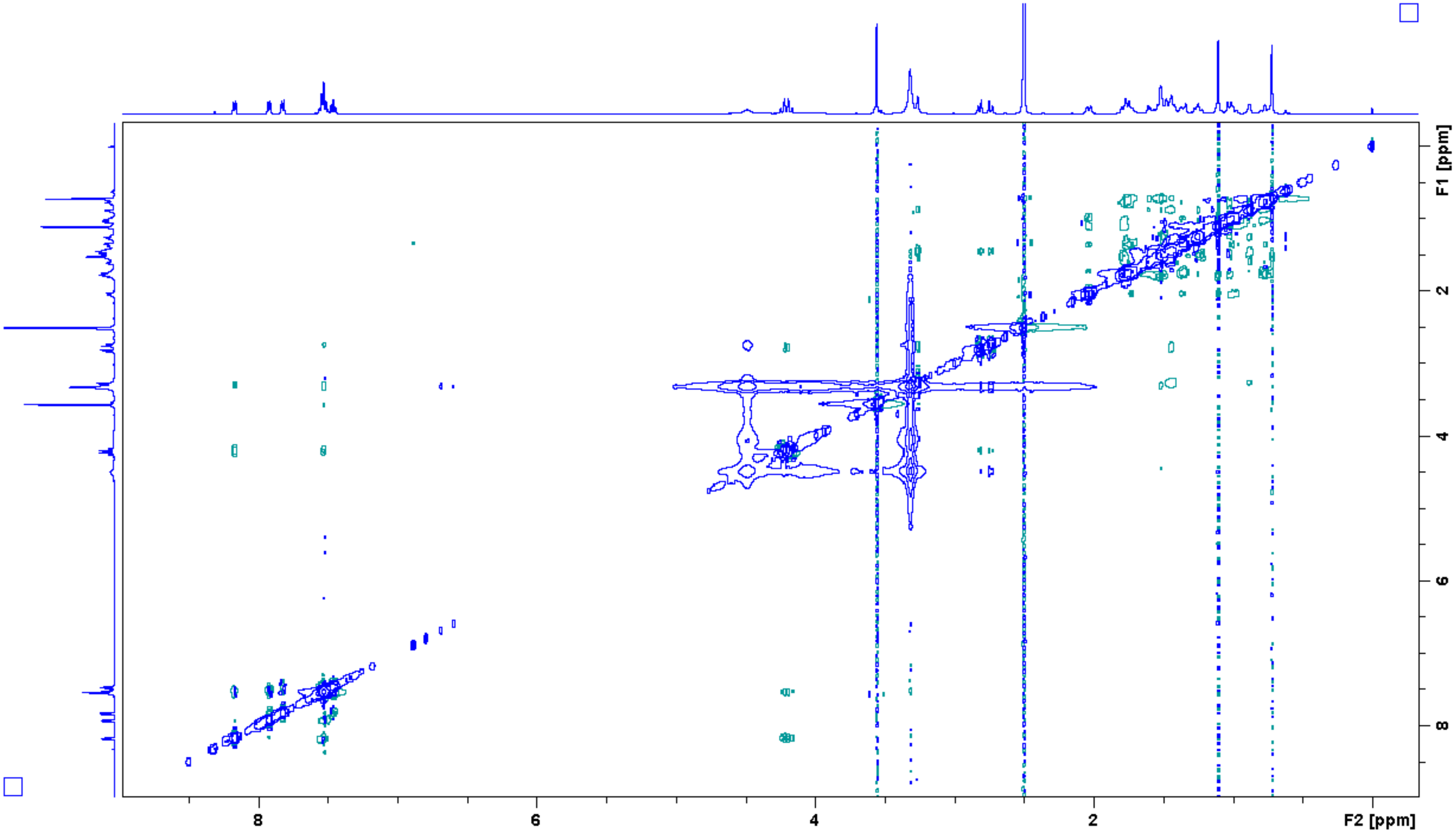

HSQC of compound (4*R*,6a*R*,7*R*,8*R*,9*S*,11b*S*)-methyl 7,8,9-trihydroxy-4,11b-dimethyl-8-(((naphthalen-1-ylmethyl)amino)methyl)tetradecahydro-6a,9-methanocyclohepta[a]naphthalene-4-carboxylate **(14)**

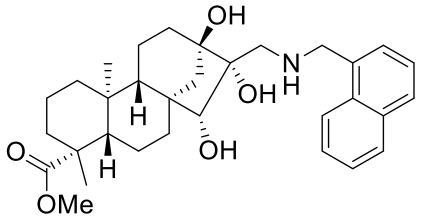

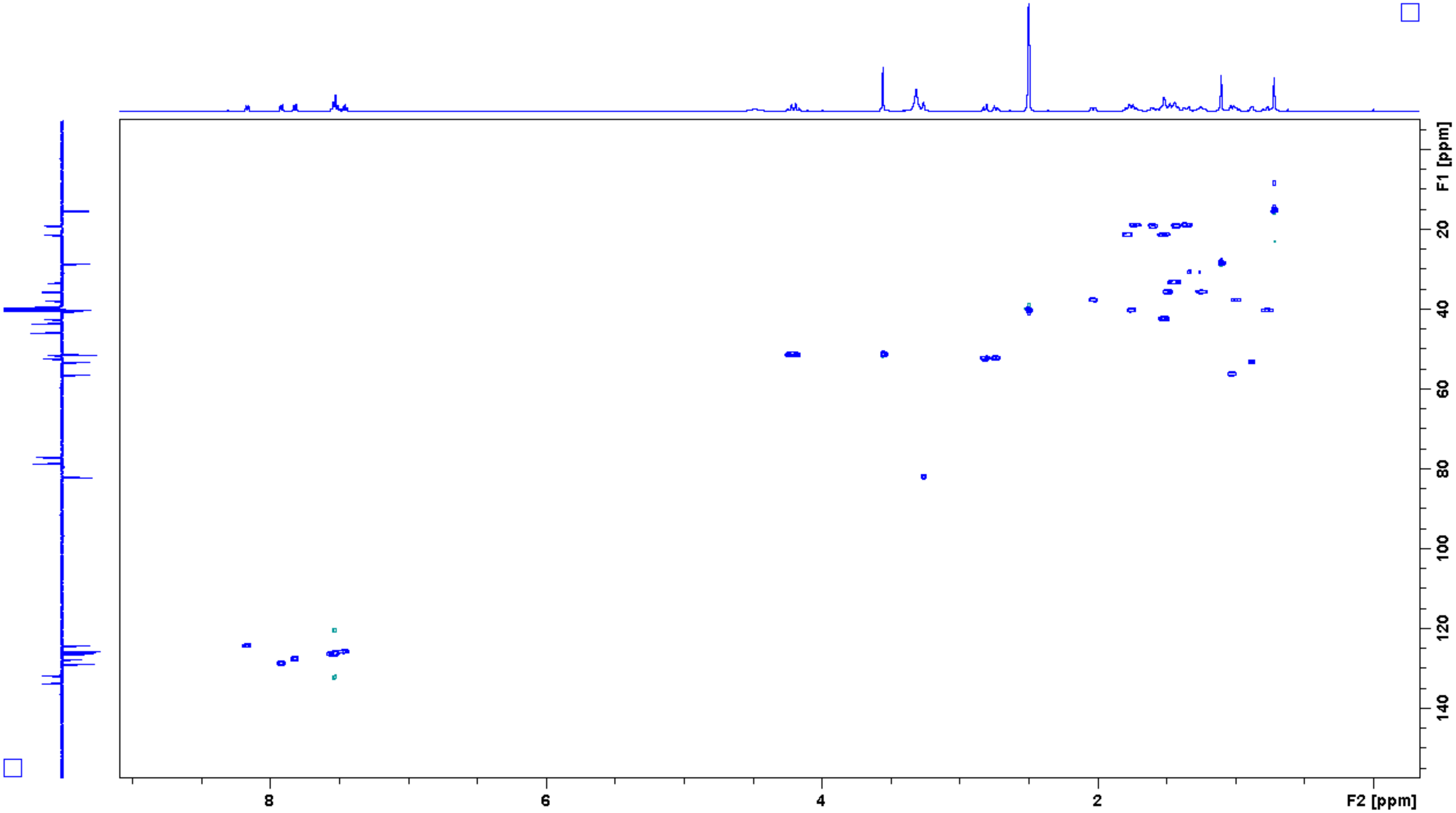

HMBC of compound(4*R*,6a*R*,7*R*,8*R*,9*S*,11b*S*)-methyl 7,8,9-trihydroxy-4,11b-dimethyl-8-(((naphthalen-1-ylmethyl)amino)methyl)tetradecahydro-6a,9-methanocyclohepta[a]naphthalene-4-carboxylate **(14)**

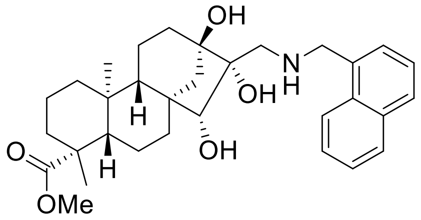

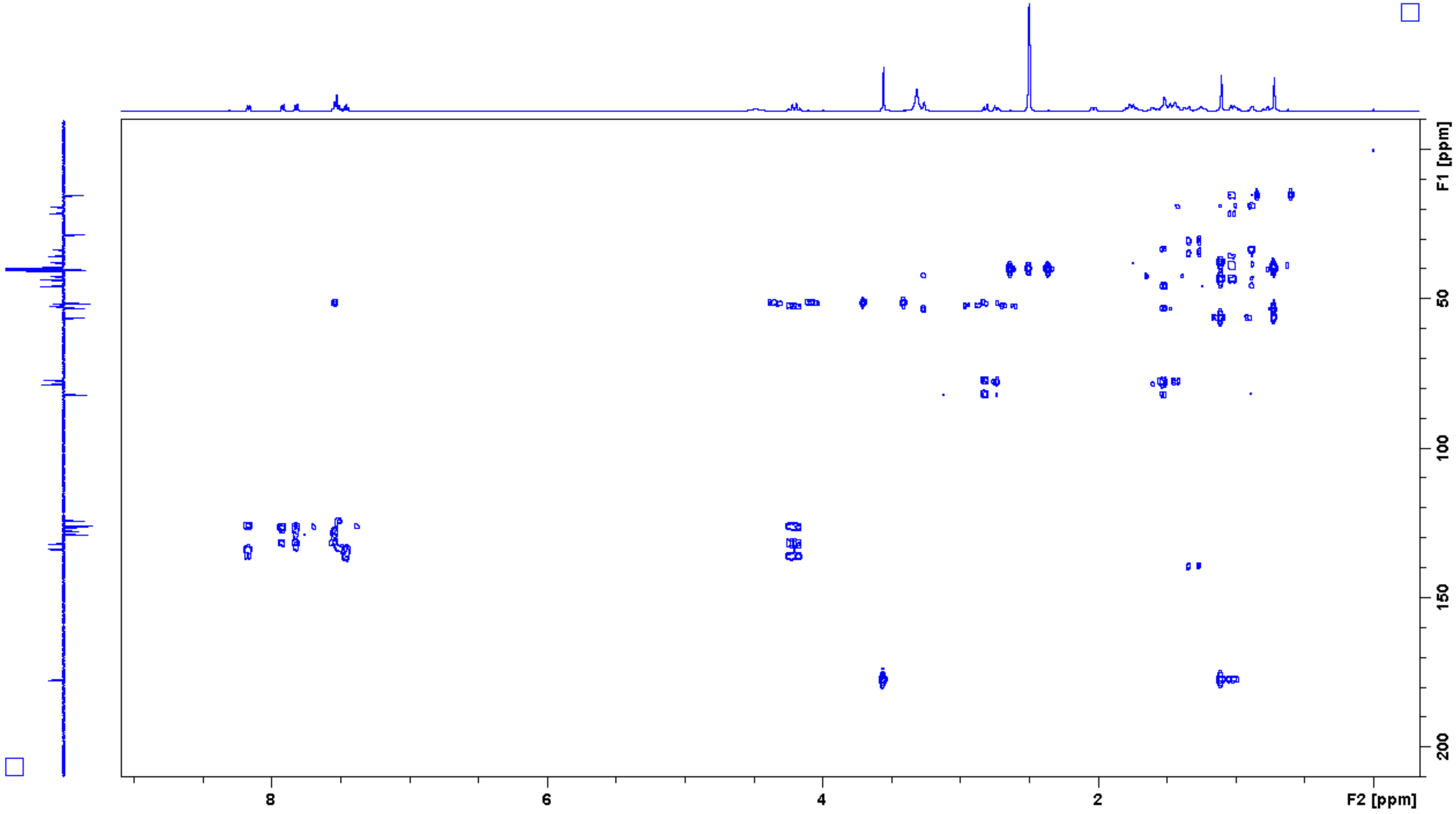

^{1}H-NMR of compound (4*R*,6a*R*,7*R*,8*R*,9*S*,11b*S*)-methyl 7,8,9-trihydroxy-4,11b-dimethyl-8-((((*R*)-1-phenylpropyl)amino)methyl)tetradecahydro-6a,9-methanocyclohepta[a]naphthalene-4-carboxylate **(15)**

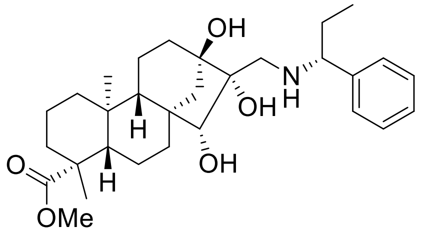

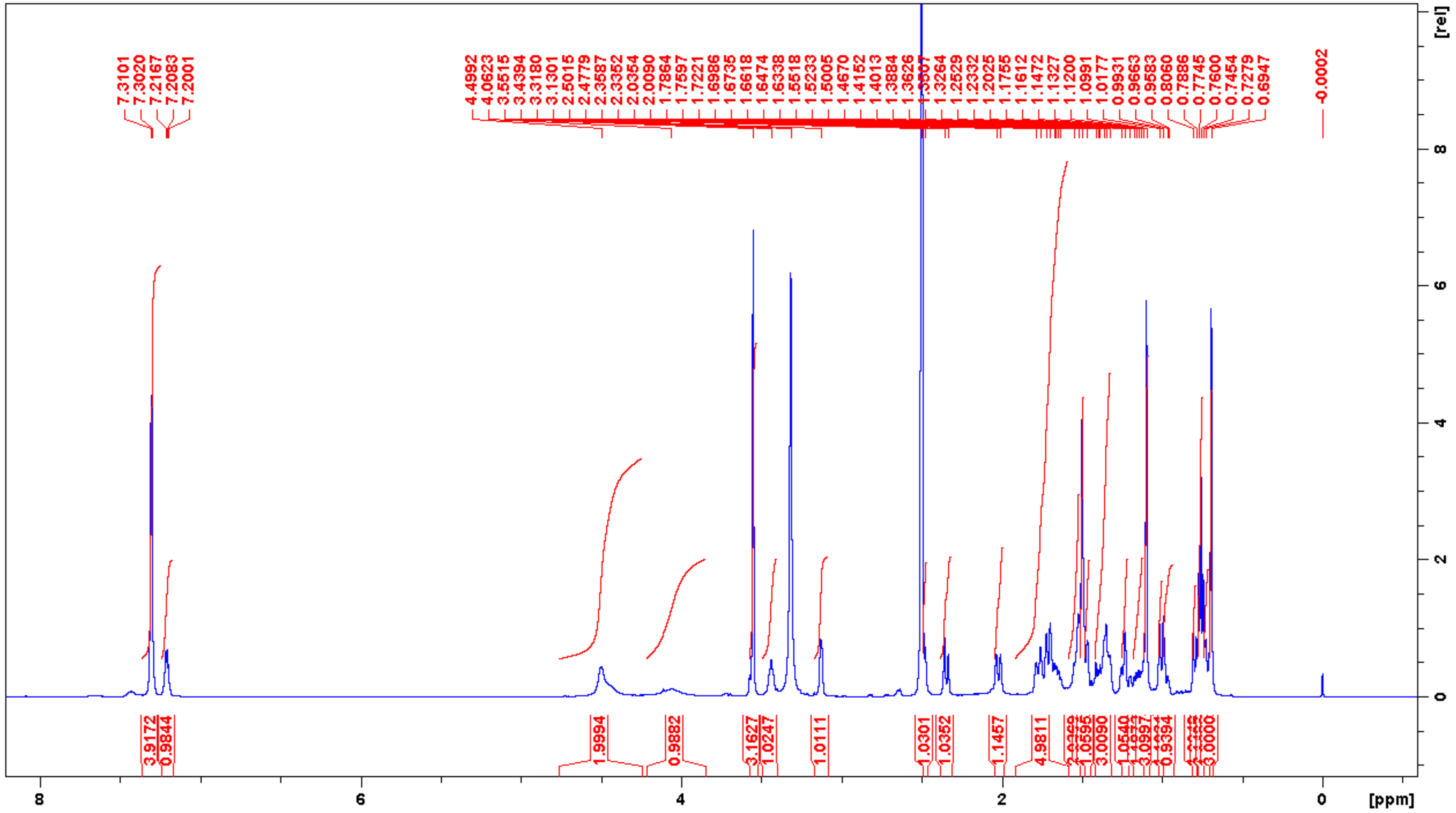

^{13}C-NMR of compound (4*R*,6a*R*,7*R*,8*R*,9*S*,11b*S*)-methyl 7,8,9-trihydroxy-4,11b-dimethyl-8-((((*R*)-1-phenylpropyl)amino)methyl)tetradecahydro-6a,9-methanocyclohepta[a]naphthalene-4-carboxylate **(15)**

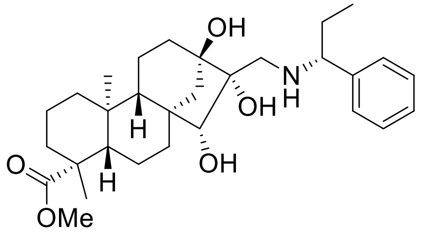

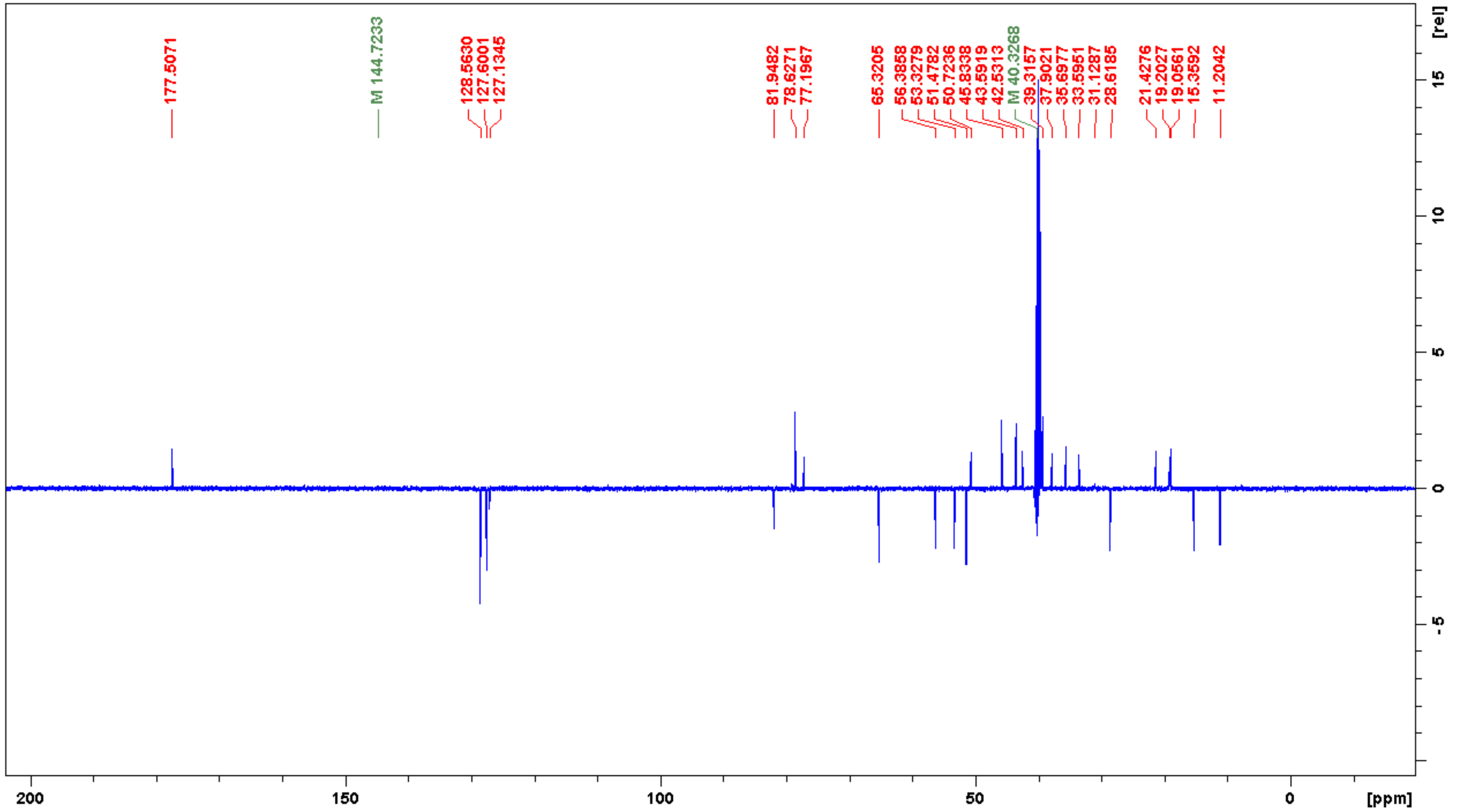

COSY of compound (4*R*,6a*R*,7*R*,8*R*,9*S*,11b*S*)-methyl 7,8,9-trihydroxy-4,11b-dimethyl-8-((((*R*)-1-phenylpropyl)amino)methyl)tetradecahydro-6a,9-methanocyclohepta[a]naphthalene-4-carboxylate **(15)**

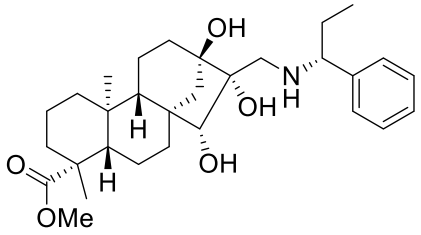

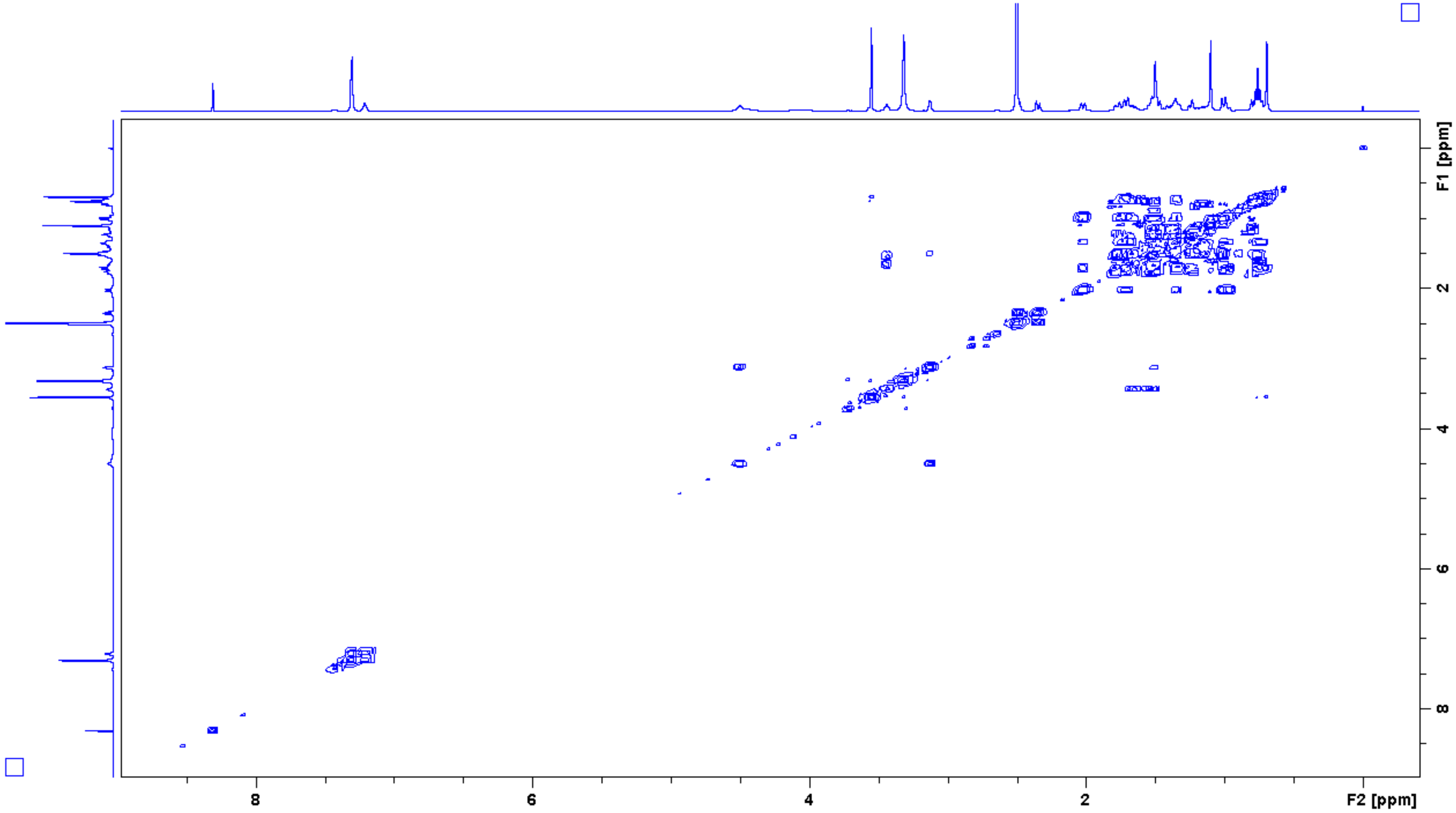

NOESY of compound (4*R*,6a*R*,7*R*,8*R*,9*S*,11b*S*)-methyl 7,8,9-trihydroxy-4,11b-dimethyl-8-((((*R*)-1-phenylpropyl)amino)methyl)tetradecahydro-6a,9-methanocyclohepta[a]naphthalene-4-carboxylate **(15)**

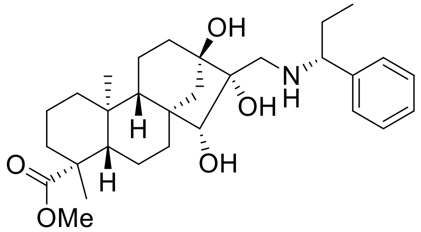

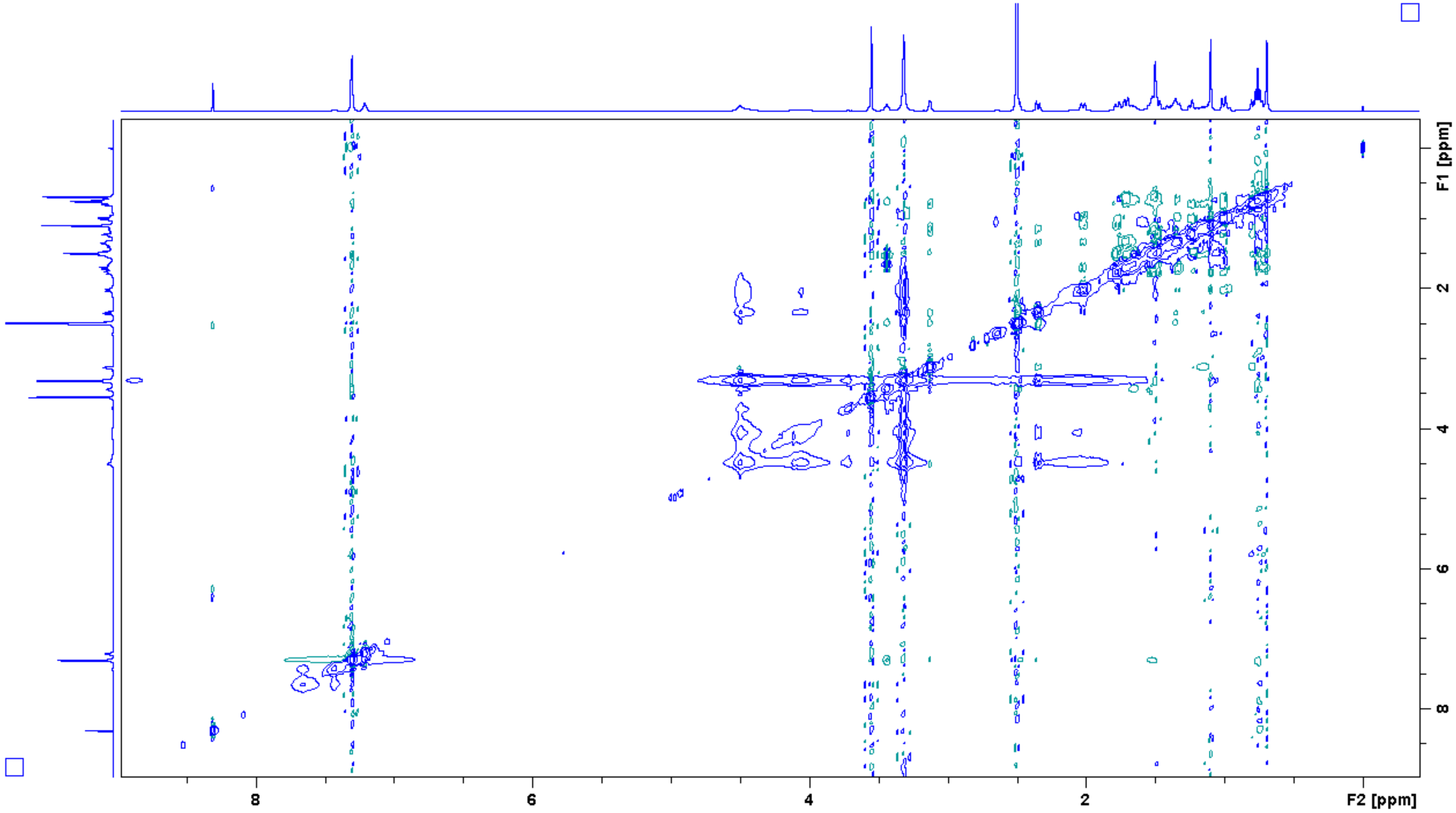

HSQC of compound (4*R*,6a*R*,7*R*,8*R*,9*S*,11b*S*)-methyl 7,8,9-trihydroxy-4,11b-dimethyl-8-((((*R*)-1-phenylpropyl)amino)methyl)tetradecahydro-6a,9-methanocyclohepta[a]naphthalene-4-carboxylate **(15)**

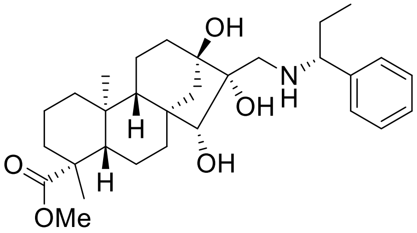

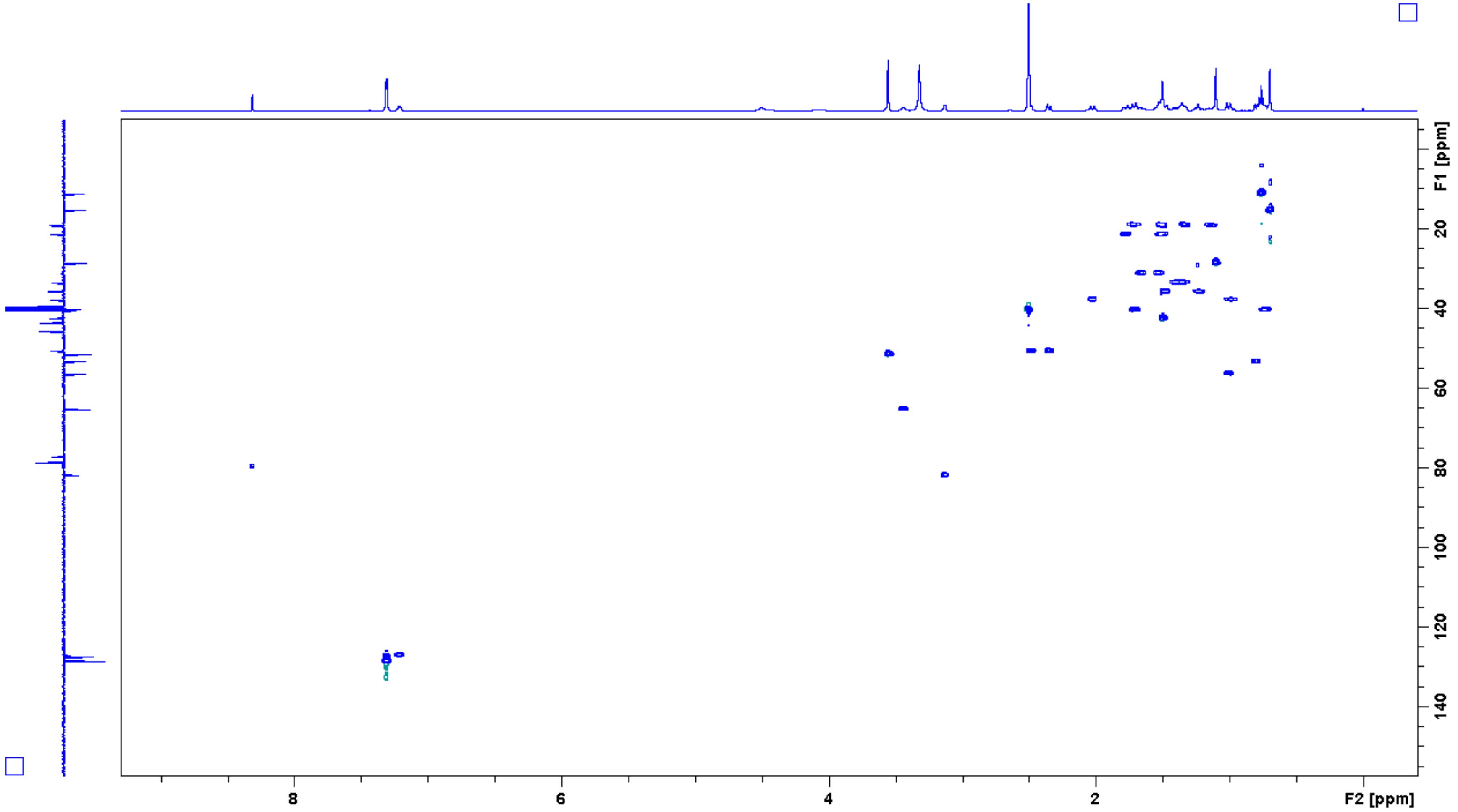

HMBC of compound (4*R*,6a*R*,7*R*,8*R*,9*S*,11b*S*)-methyl 7,8,9-trihydroxy-4,11b-dimethyl-8-((((*R*)-1-phenylpropyl)amino)methyl)tetradecahydro-6a,9-methanocyclohepta[a]naphthalene-4-carboxylate **(15)**

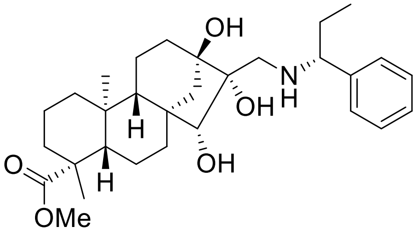

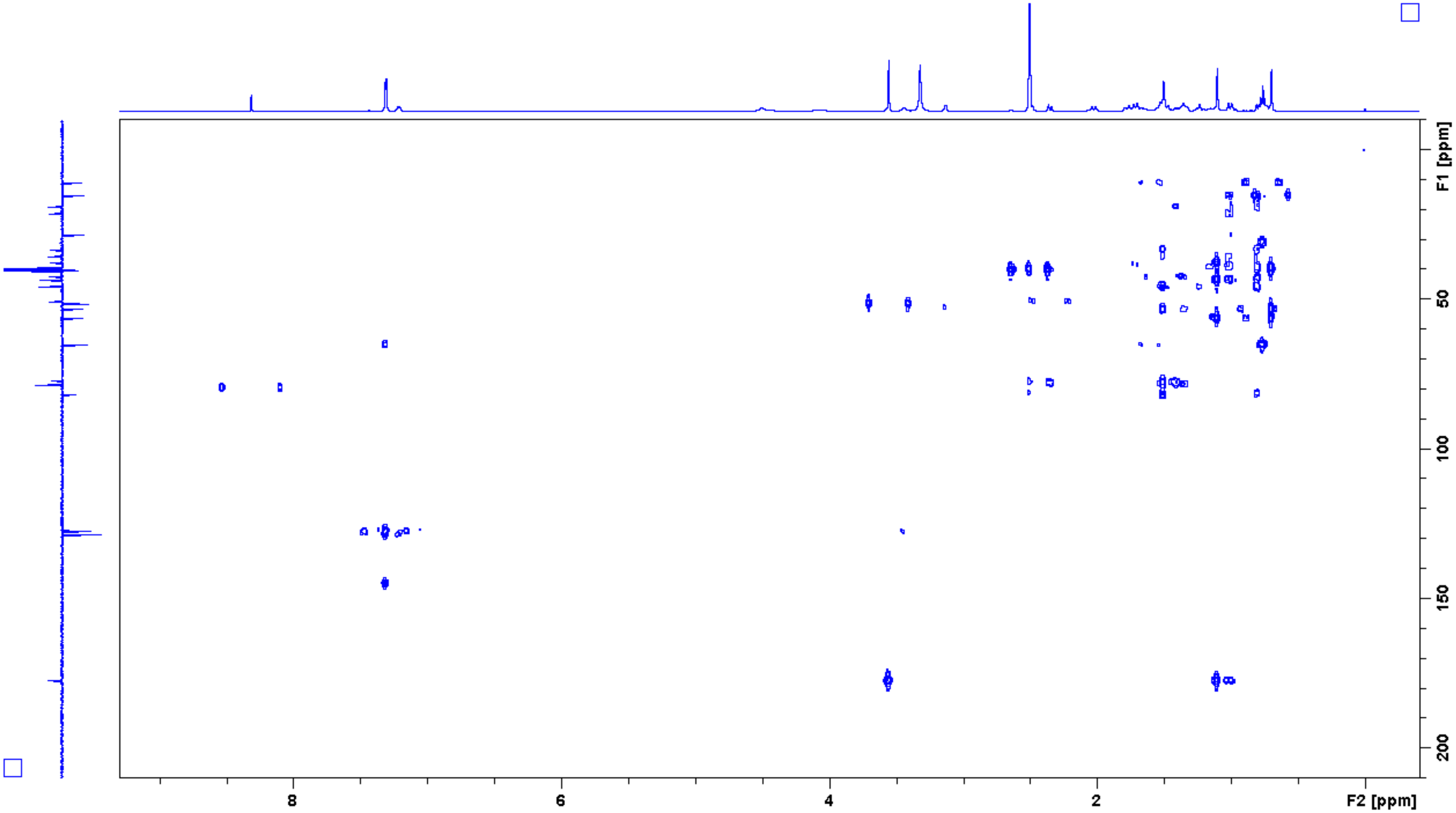

^{1}H-NMR of compound (4*R*,6a*R*,7*R*,8*R*,9*S*,11b*S*)-methyl 7,8,9-trihydroxy-4,11b-dimethyl-8-((((*S*)-1-phenylpropyl)amino)methyl)tetradecahydro-6a,9-methanocyclohepta[a]naphthalene-4-carboxylate **(16)**

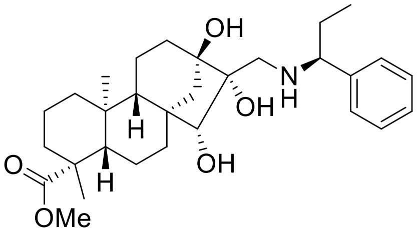

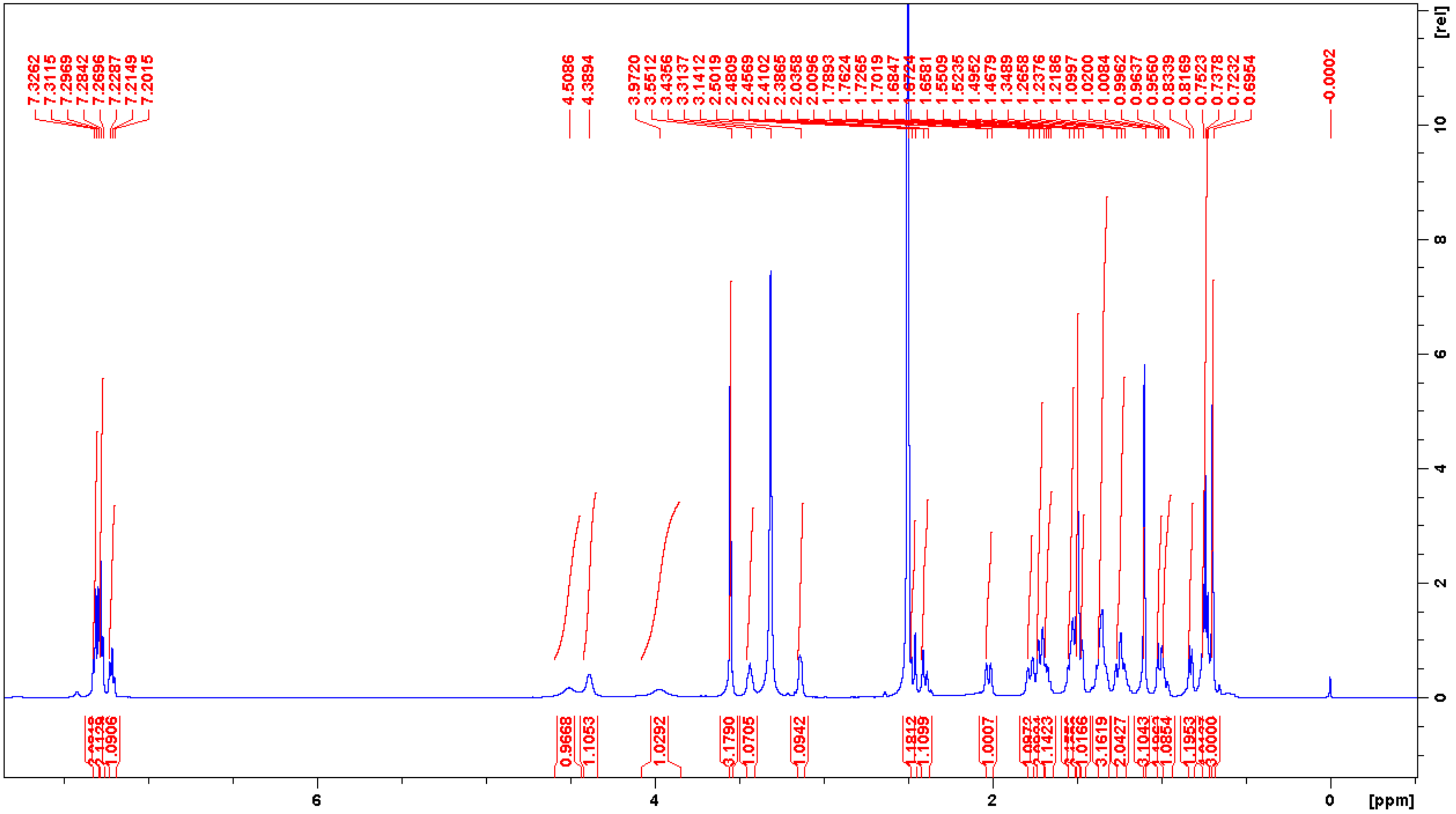

^{13}C-NMR of compound (4*R*,6a*R*,7*R*,8*R*,9*S*,11b*S*)-methyl 7,8,9-trihydroxy-4,11b-dimethyl-8-((((*S*)-1-phenylpropyl)amino)methyl)tetradecahydro-6a,9-methanocyclohepta[a]naphthalene-4-carboxylate **(16)**

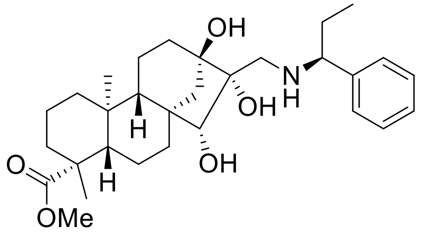

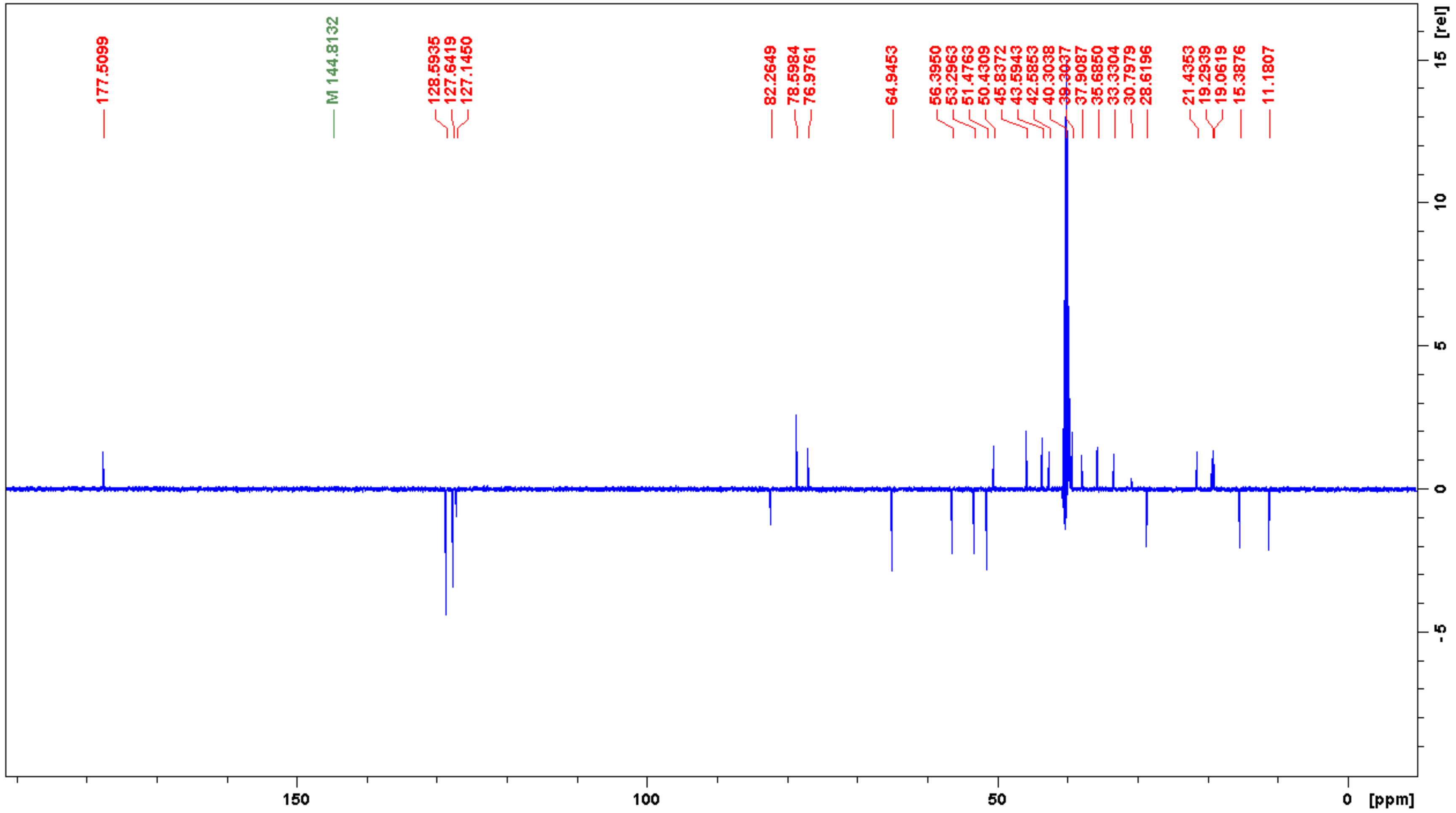

COSY of compound (4*R*,6a*R*,7*R*,8*R*,9*S*,11b*S*)-methyl 7,8,9-trihydroxy-4,11b-dimethyl-8-((((*S*)-1-phenylpropyl)amino)methyl)tetradecahydro-6a,9-methanocyclohepta[a]naphthalene-4-carboxylate **(16)**

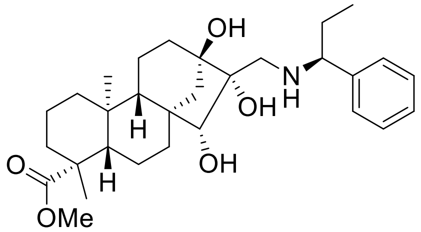

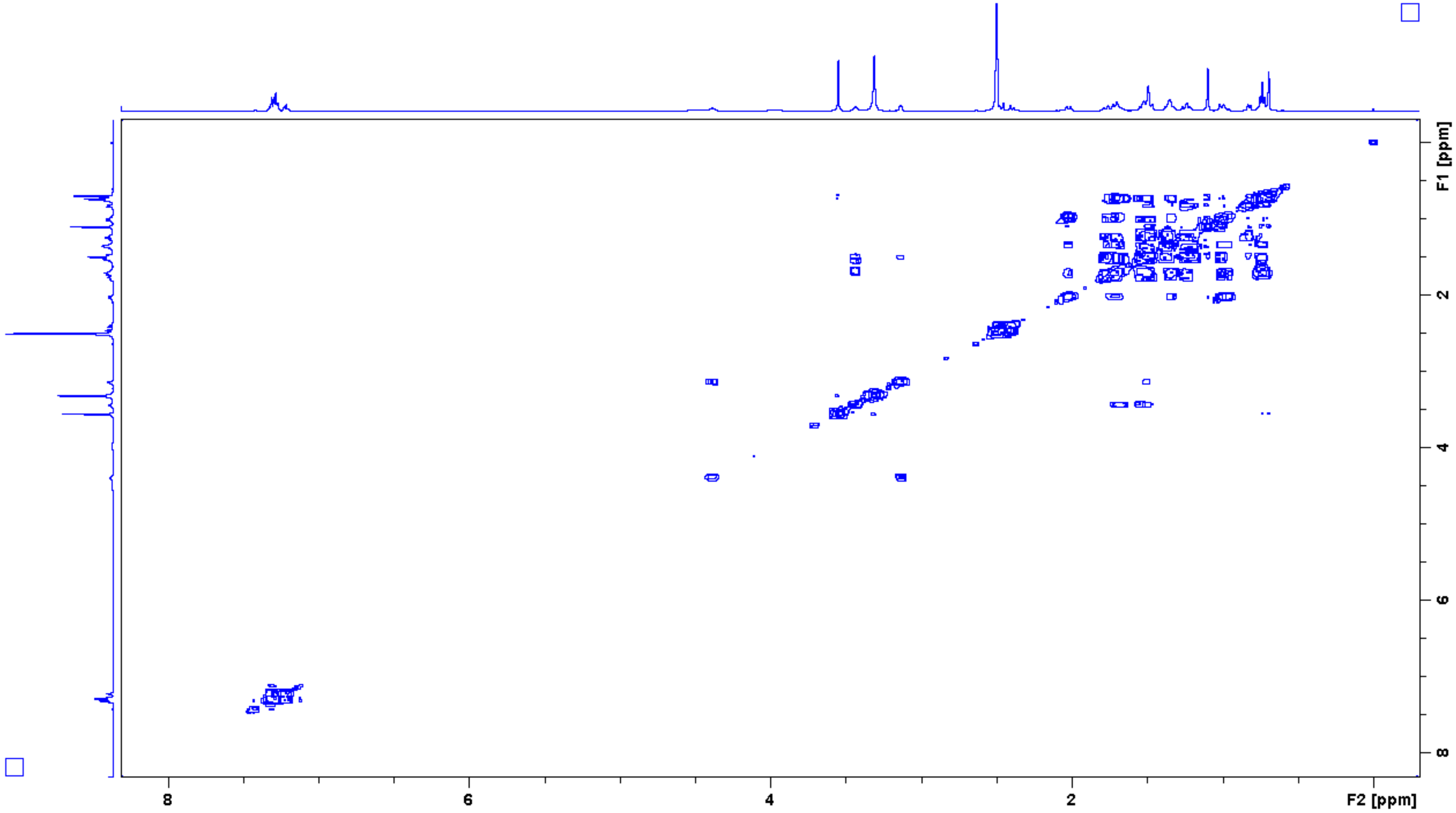

NOESY of compound (4*R*,6a*R*,7*R*,8*R*,9*S*,11b*S*)-methyl 7,8,9-trihydroxy-4,11b-dimethyl-8-((((*S*)-1-phenylpropyl)amino)methyl)tetradecahydro-6a,9-methanocyclohepta[a]naphthalene-4-carboxylate **(16)**

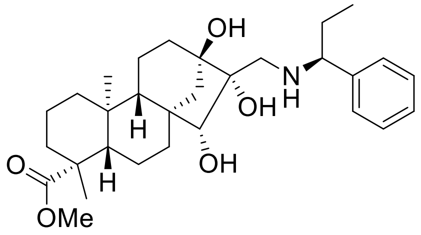

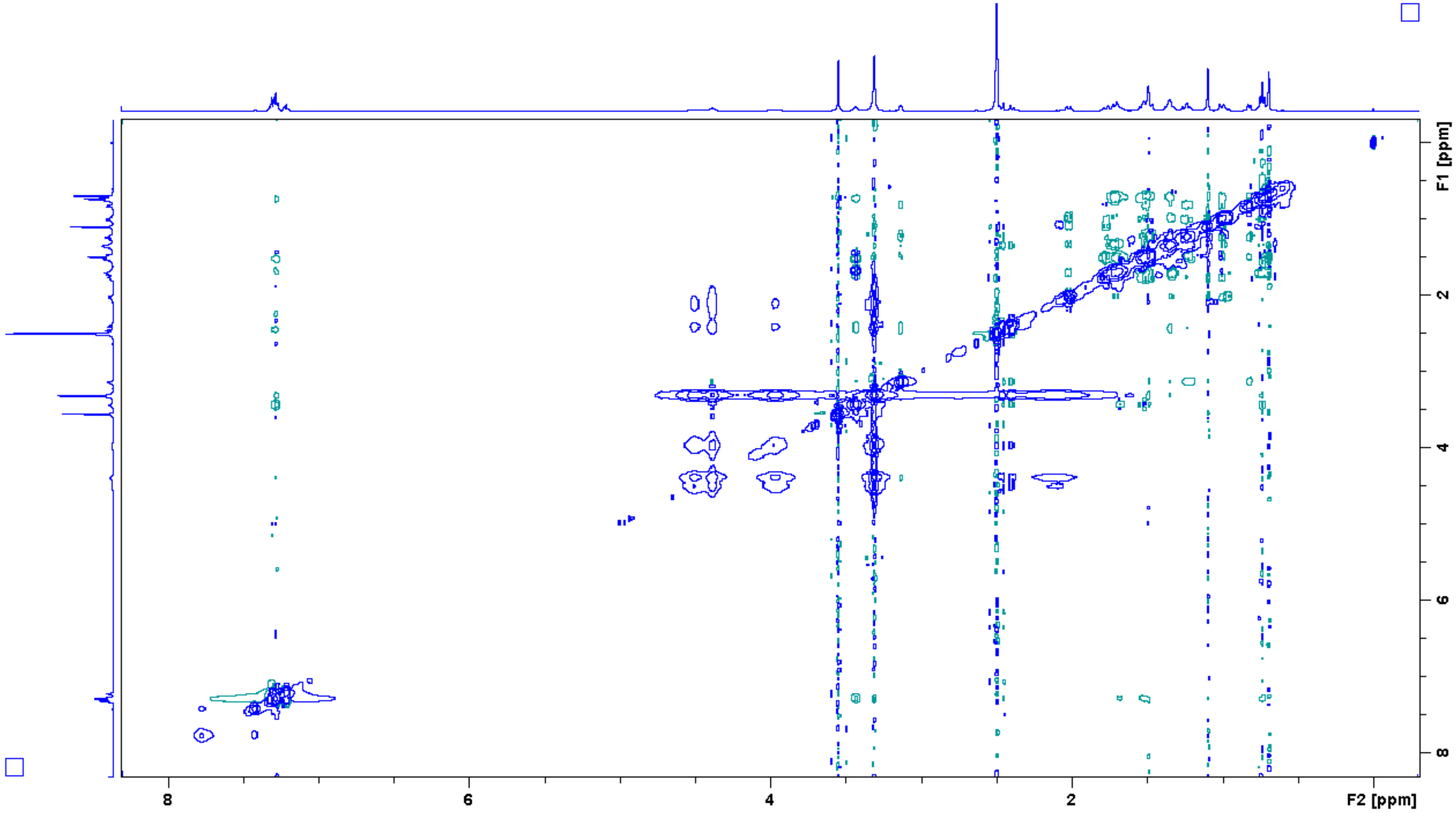

HSQC of compound (4*R*,6a*R*,7*R*,8*R*,9*S*,11b*S*)-methyl 7,8,9-trihydroxy-4,11b-dimethyl-8-((((*S*)-1-phenylpropyl)amino)methyl)tetradecahydro-6a,9-methanocyclohepta[a]naphthalene-4-carboxylate **(16)**

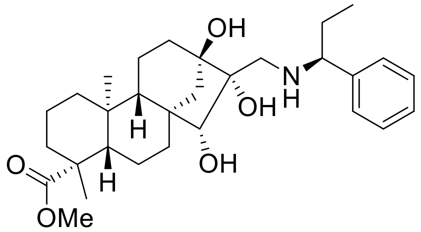

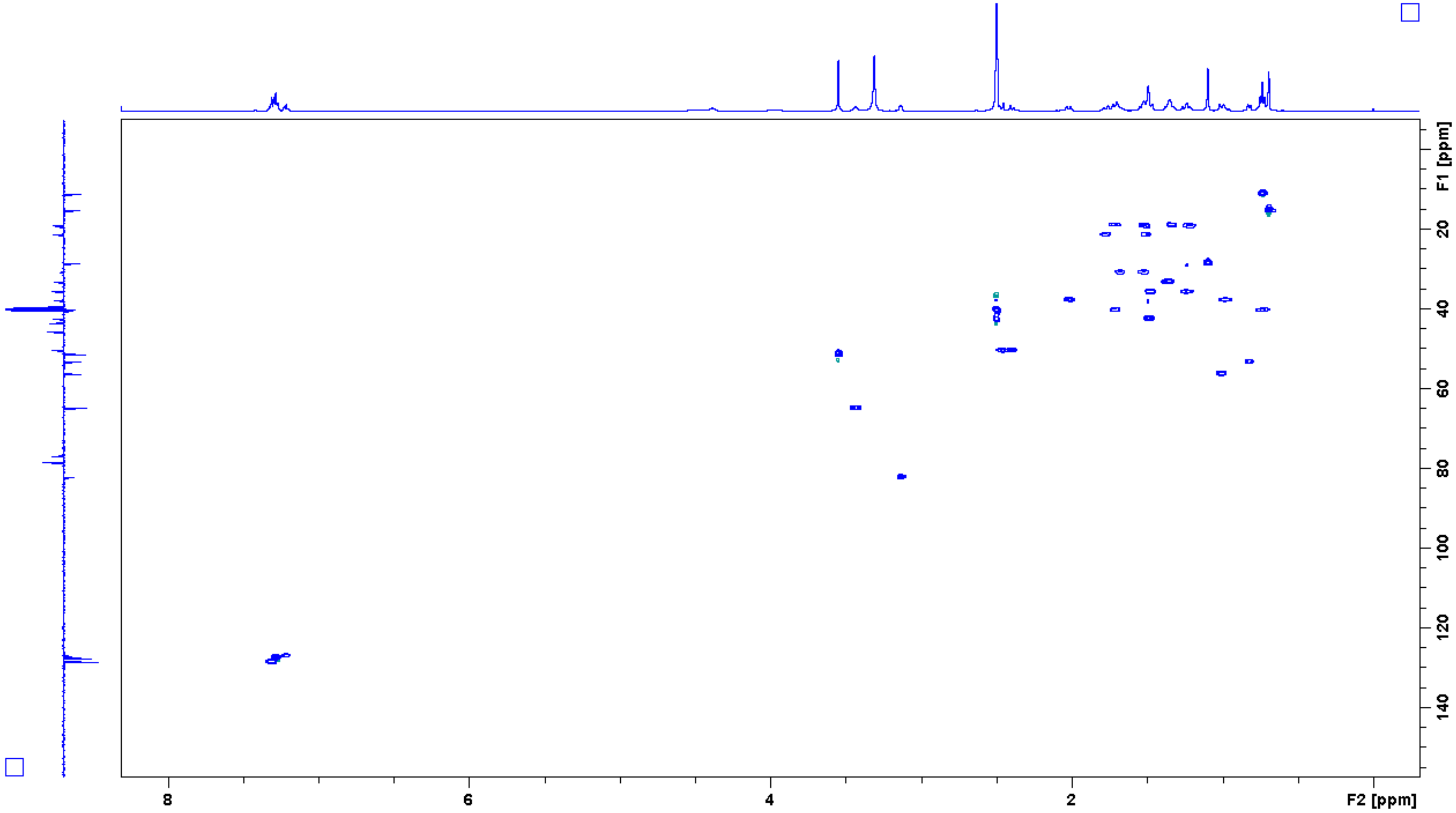

HMBC of compound (4*R*,6a*R*,7*R*,8*R*,9*S*,11b*S*)-methyl 7,8,9-trihydroxy-4,11b-dimethyl-8-((((*S*)-1-phenylpropyl)amino)methyl)tetradecahydro-6a,9-methanocyclohepta[a]naphthalene-4-carboxylate **(16)**

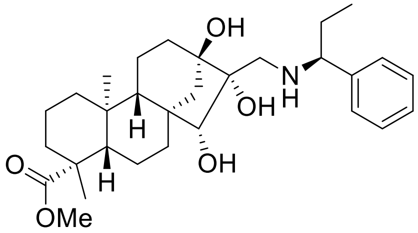

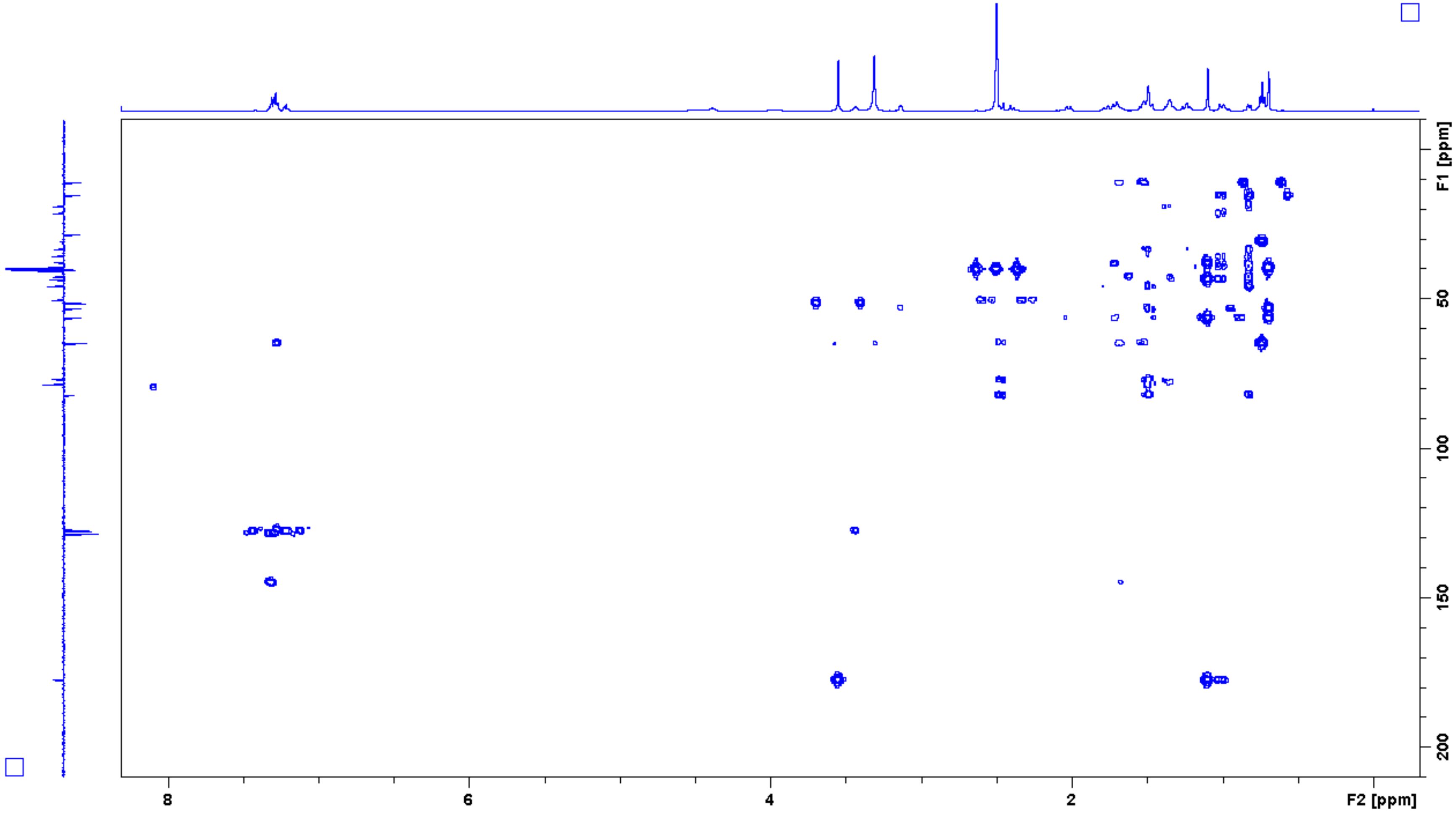

^{1}H-NMR of compound (4*R*,6a*R*,7*R*,8*R*,9*S*,11b*S*)-methyl 8-(aminomethyl)-7,8,9-trihydroxy-4,11b-dimethyltetradecahydro-6a,9-methanocyclohepta[a]naphthalene-4-carboxylate **(17)**

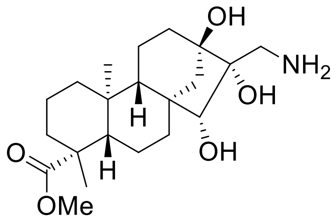

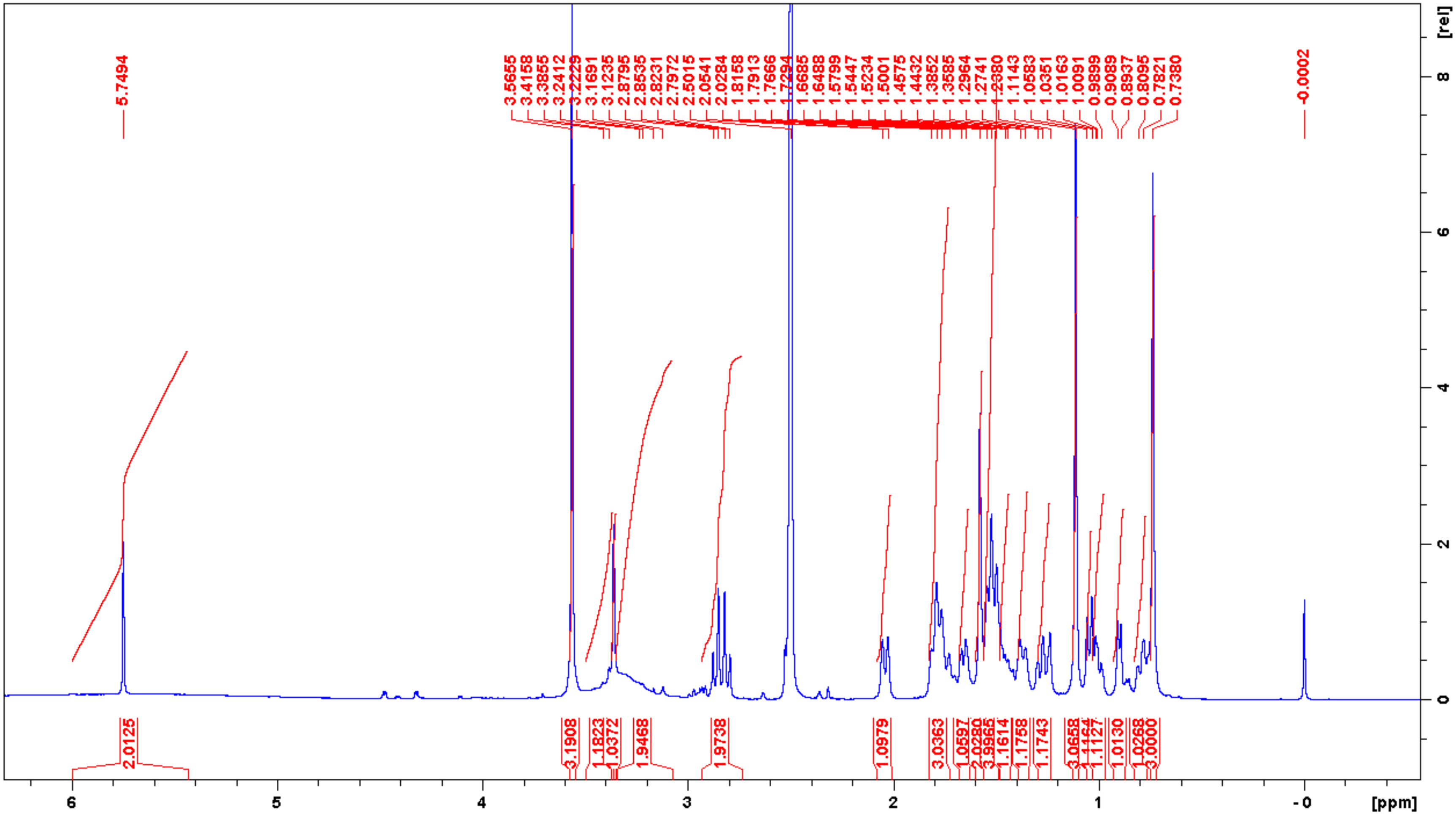

^{13}C-NMR of compound (4*R*,6a*R*,7*R*,8*R*,9*S*,11b*S*)-methyl 8-(aminomethyl)-7,8,9-trihydroxy-4,11b-dimethyltetradecahydro-6a,9-methanocyclohepta[a]naphthalene-4-carboxylate **(17)**

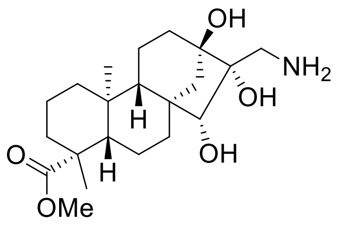

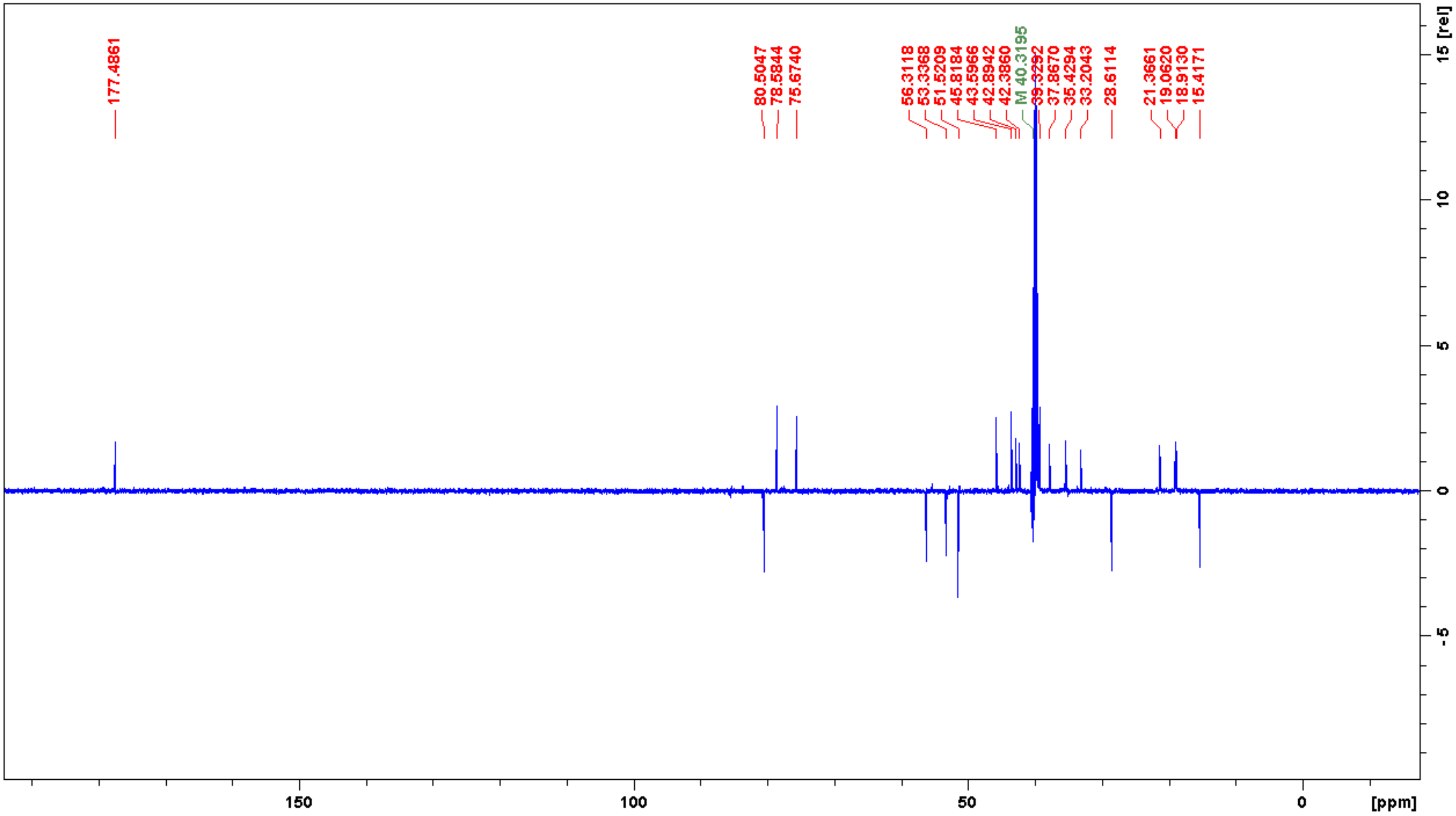

COSY of compound (4*R*,6a*R*,7*R*,8*R*,9*S*,11b*S*)-methyl 8-(aminomethyl)-7,8,9-trihydroxy-4,11b-dimethyltetradecahydro-6a,9-methanocyclohepta[a]naphthalene-4-carboxylate **(17)**

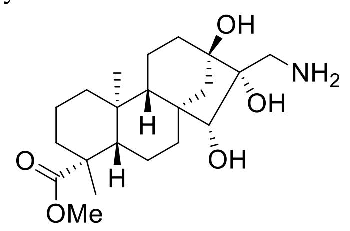

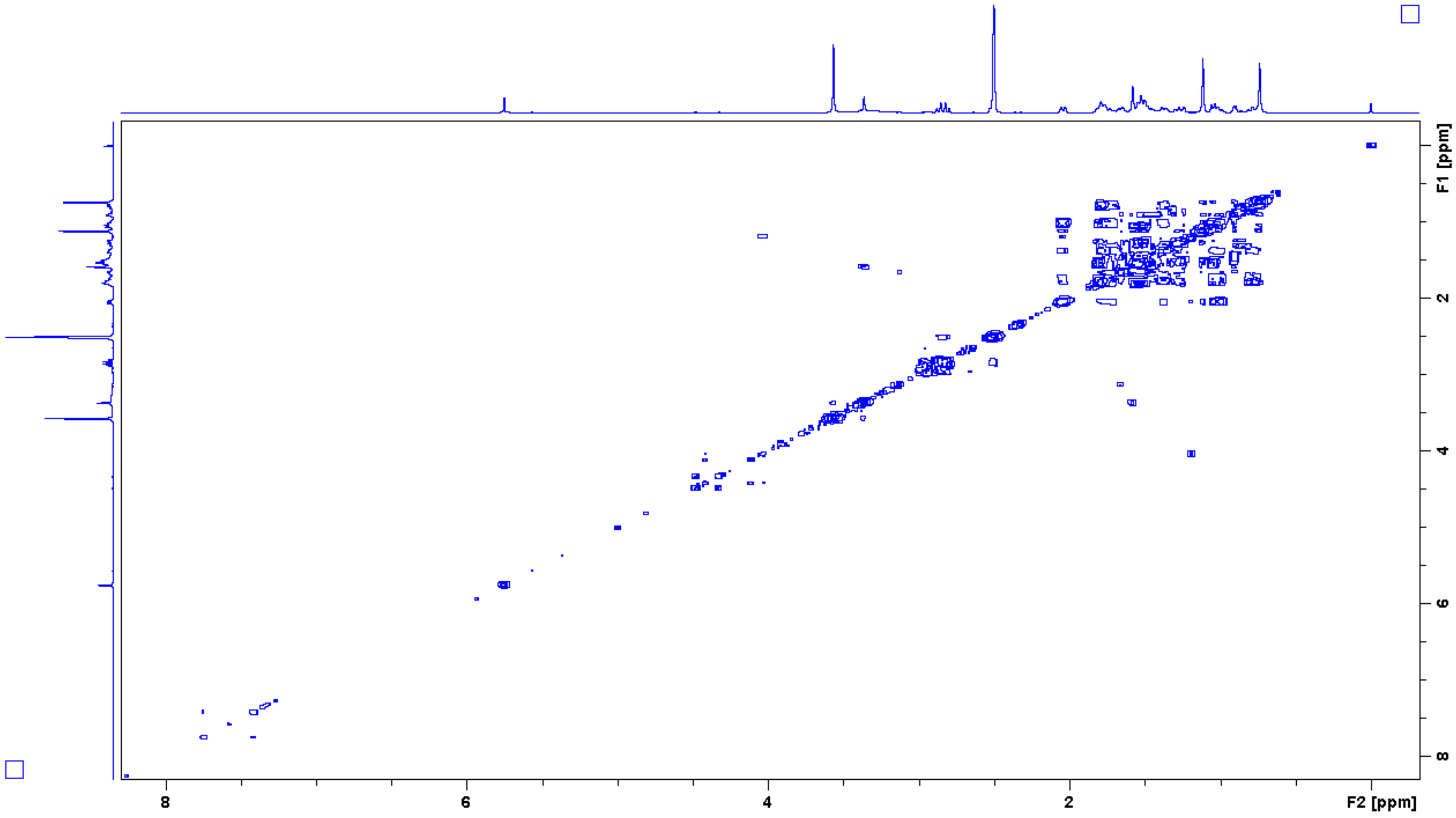

NOESY of compound (4*R*,6a*R*,7*R*,8*R*,9*S*,11b*S*)-methyl 8-(aminomethyl)-7,8,9-trihydroxy-4,11b-dimethyltetradecahydro-6a,9-methanocyclohepta[a]naphthalene-4-carboxylate **(17)**

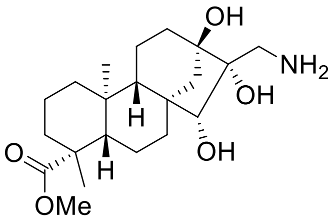

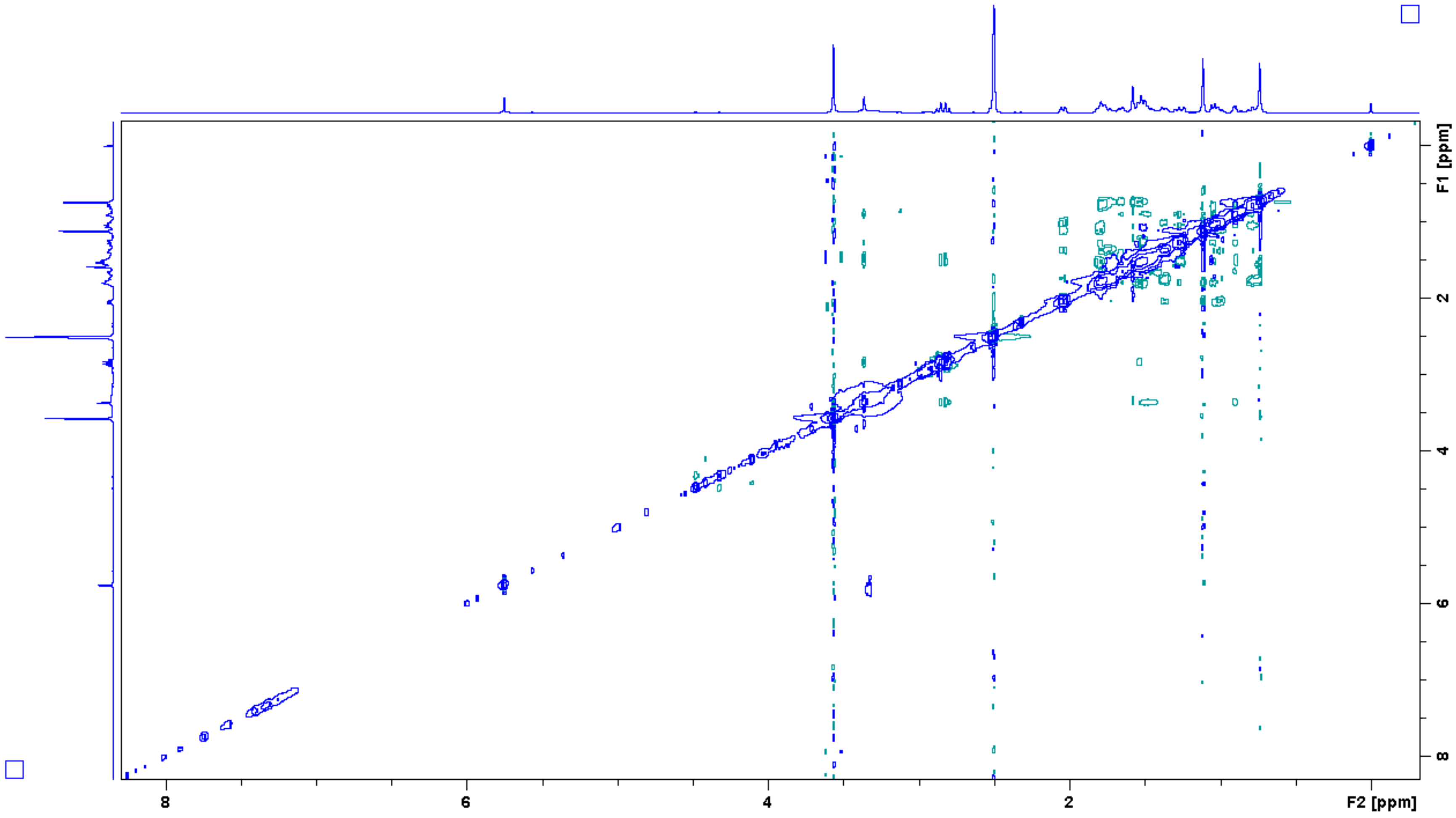

HSQC of compound (4*R*,6a*R*,7*R*,8*R*,9*S*,11b*S*)-methyl 8-(aminomethyl)-7,8,9-trihydroxy-4,11b-dimethyltetradecahydro-6a,9-methanocyclohepta[a]naphthalene-4-carboxylate **(17)**

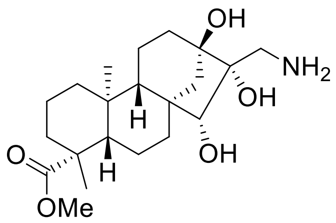

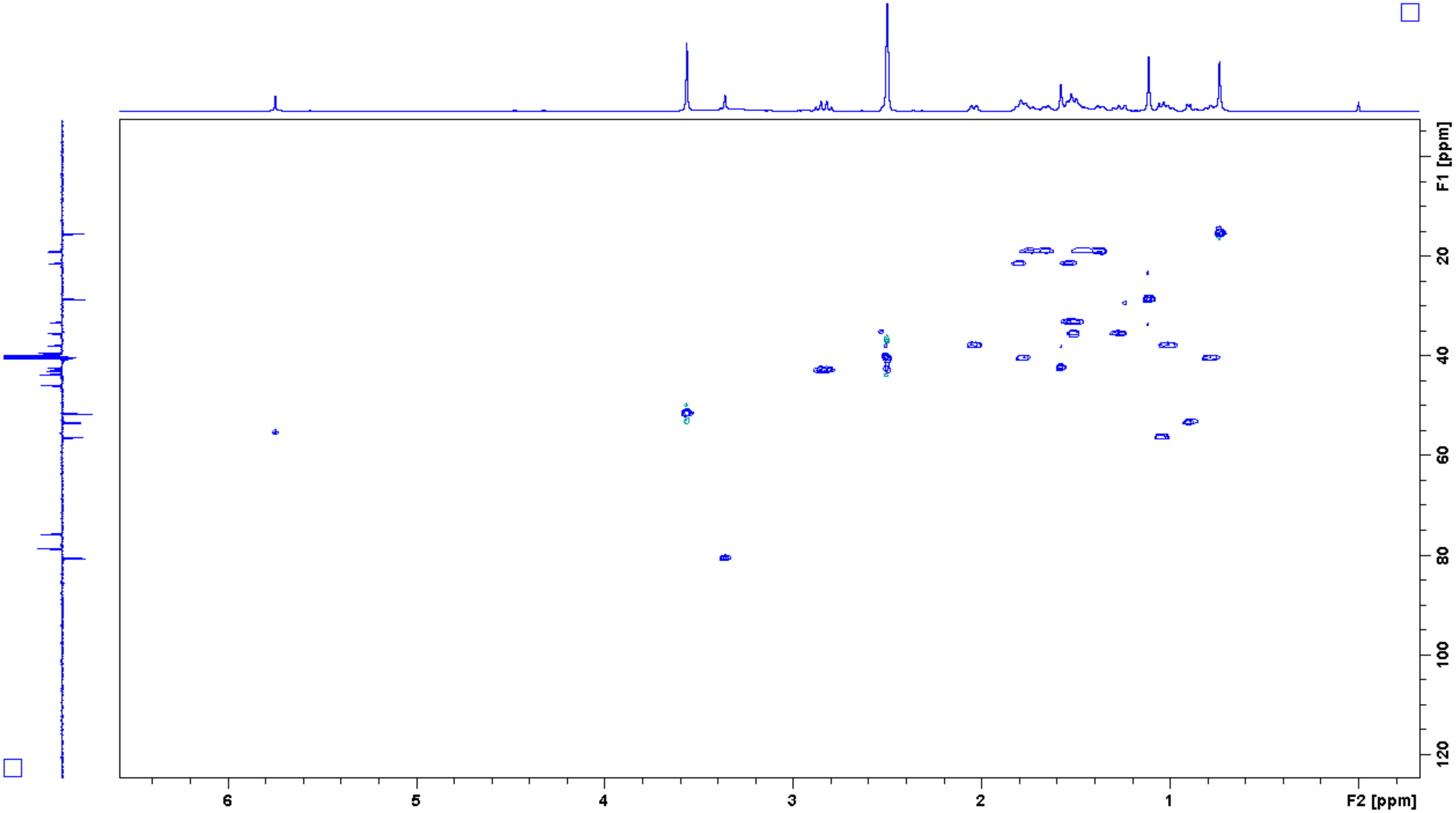

HMBC of compound (4*R*,6a*R*,7*R*,8*R*,9*S*,11b*S*)-methyl 8-(aminomethyl)-7,8,9-trihydroxy-4,11b-dimethyltetradecahydro-6a,9-methanocyclohepta[a]naphthalene-4-carboxylate **(17)**

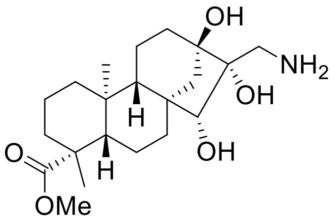

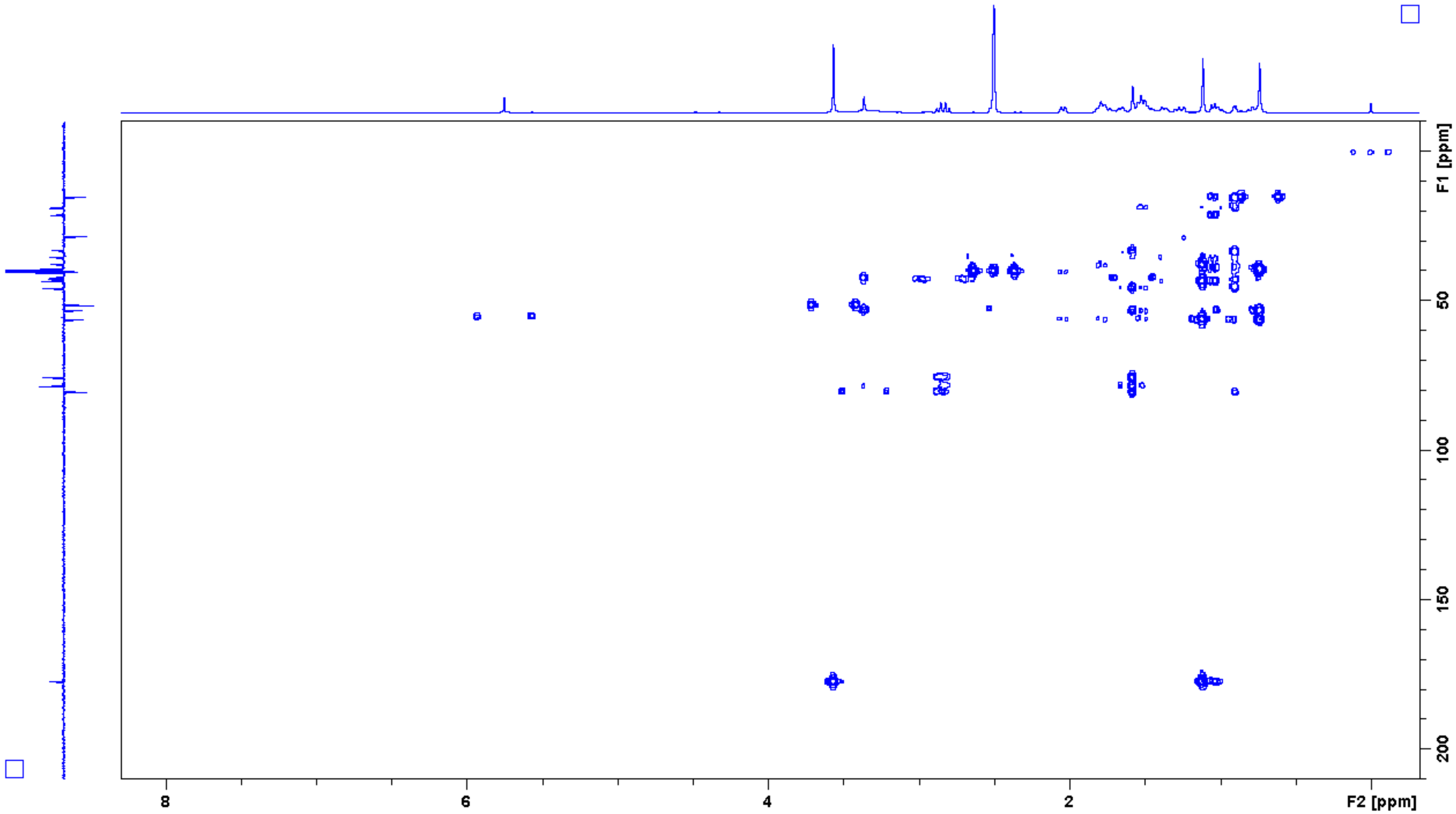

^{1}H-NMR of compound (4*R*,5'*R*,6a*R*,7*R*,9*S*,11b*S*)-methyl 3'-benzyl-7,9-dihydroxy-4,11b-dimethyldodecahydro-1H-spiro[6a,9-methanocyclohepta[a]naphthalene-8,5'-oxazolidine]-4-carboxylate **(18)**

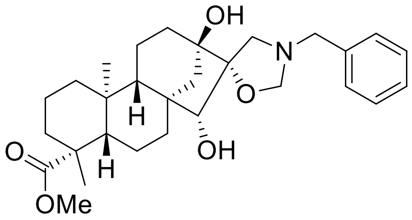

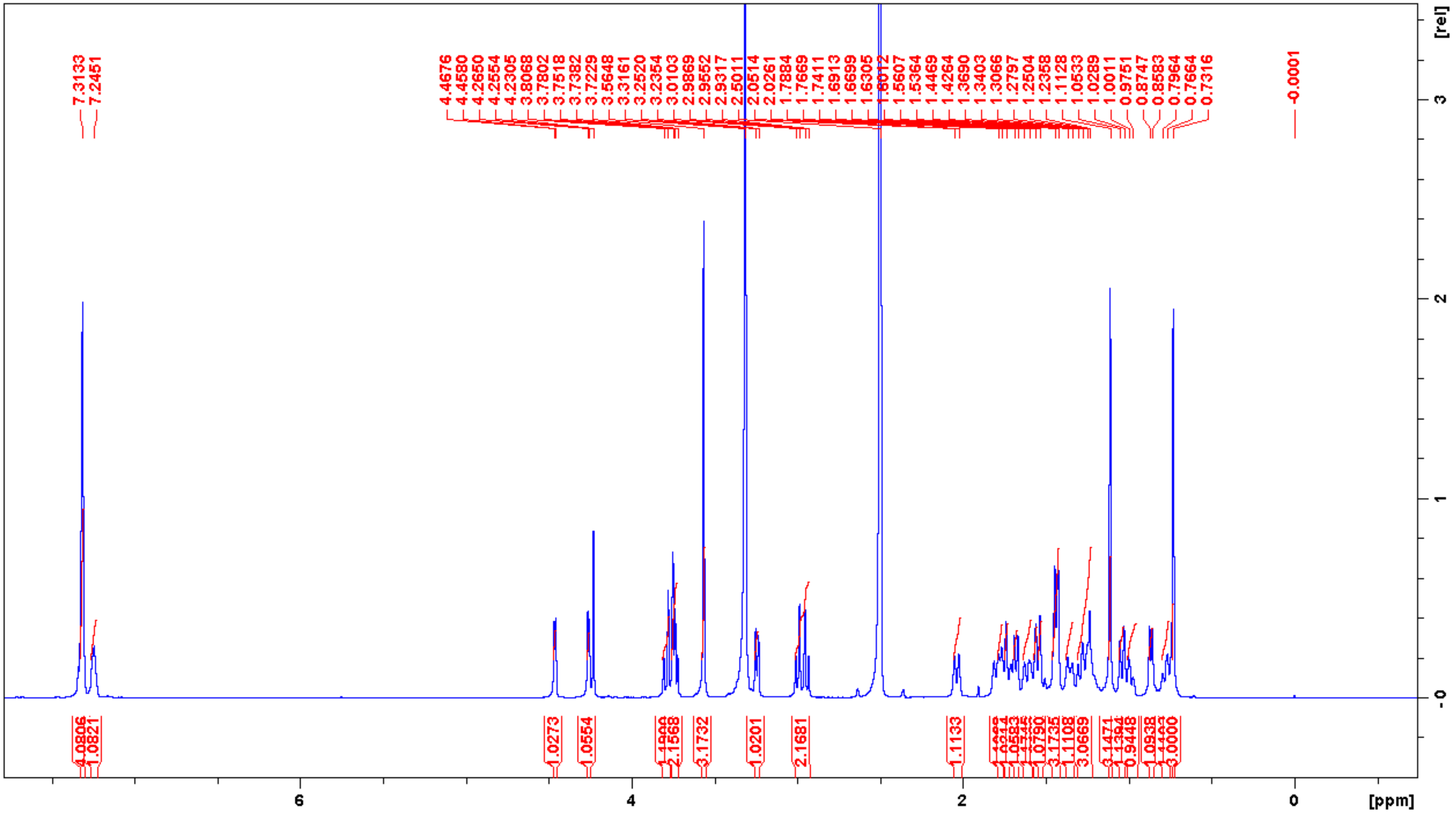

^{13}C-NMR of compound (4*R*,5'*R*,6a*R*,7*R*,9*S*,11b*S*)-methyl 3'-benzyl-7,9-dihydroxy-4,11b-dimethyldodecahydro-1H-spiro[6a,9-methanocyclohepta[a]naphthalene-8,5'-oxazolidine]-4-carboxylate **(18)**

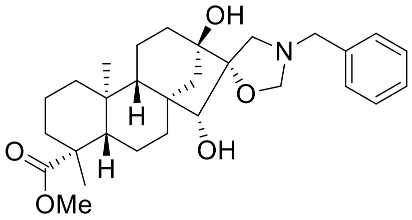

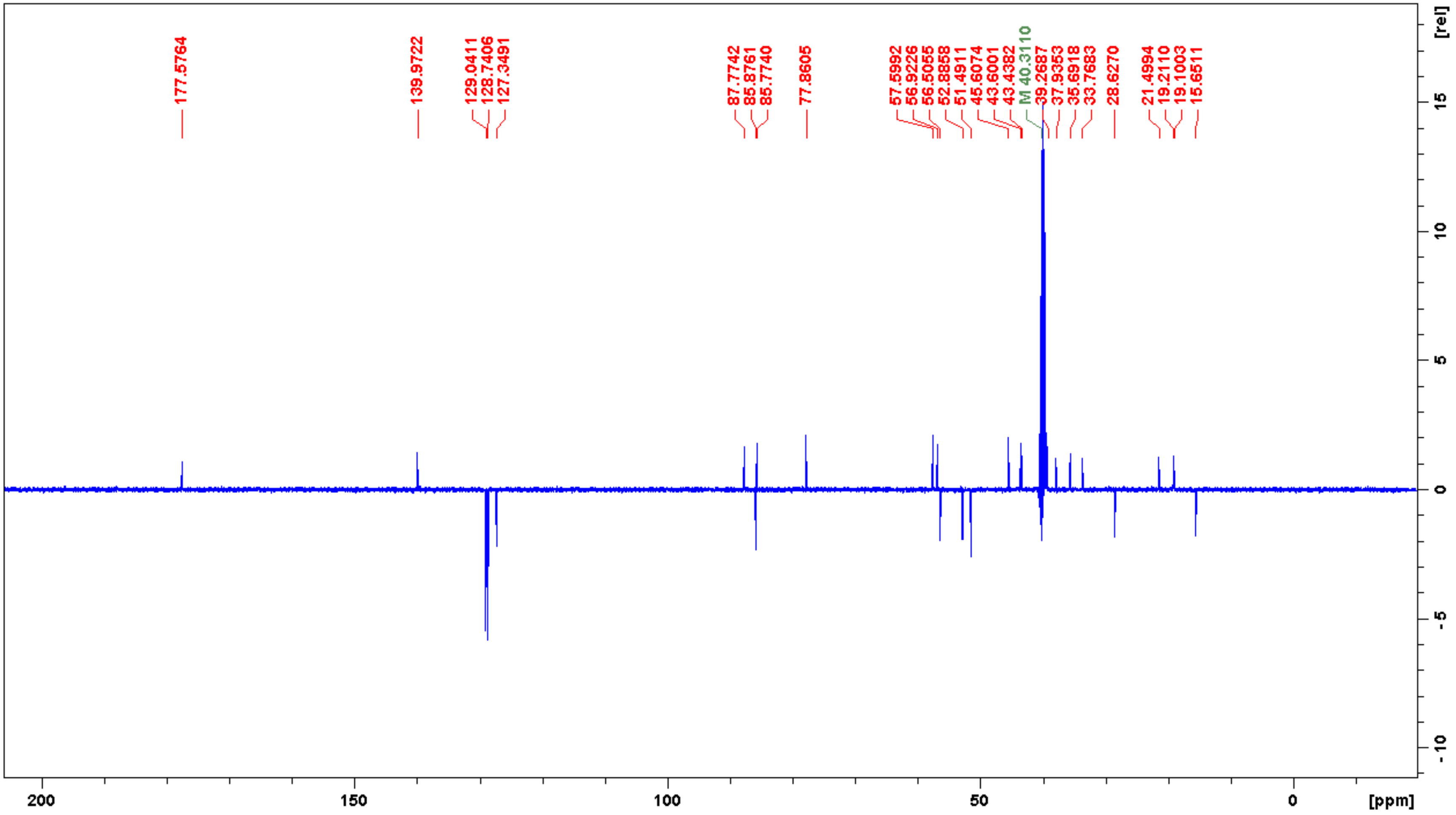

COSY of compound (4*R*,5'*R*,6a*R*,7*R*,9*S*,11b*S*)-methyl 3'-benzyl-7,9-dihydroxy-4,11b-dimethyldodecahydro-1H-spiro[6a,9-methanocyclohepta[a]naphthalene-8,5'-oxazolidine]-4-carboxylate **(18)**

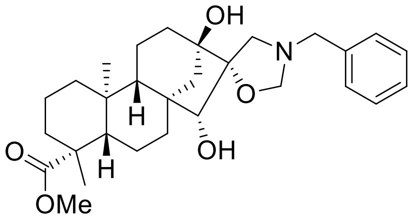

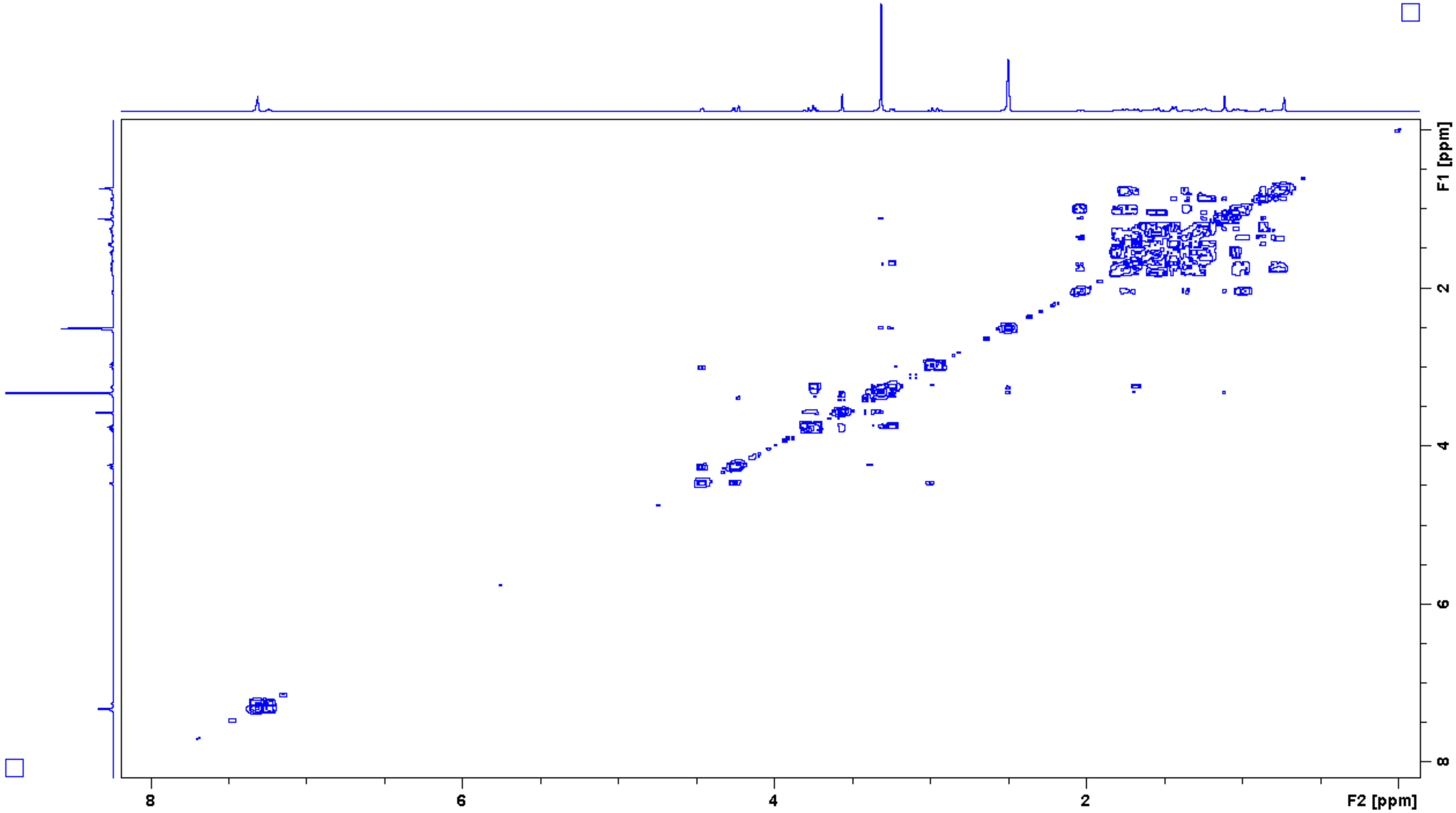

NOESY of compound (4*R*,5'*R*,6a*R*,7*R*,9*S*,11b*S*)-methyl 3'-benzyl-7,9-dihydroxy-4,11b-dimethyldodecahydro-1H-spiro[6a,9-methanocyclohepta[a]naphthalene-8,5'-oxazolidine]-4-carboxylate **(18)**

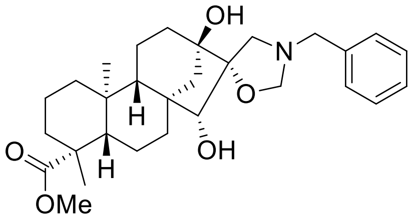

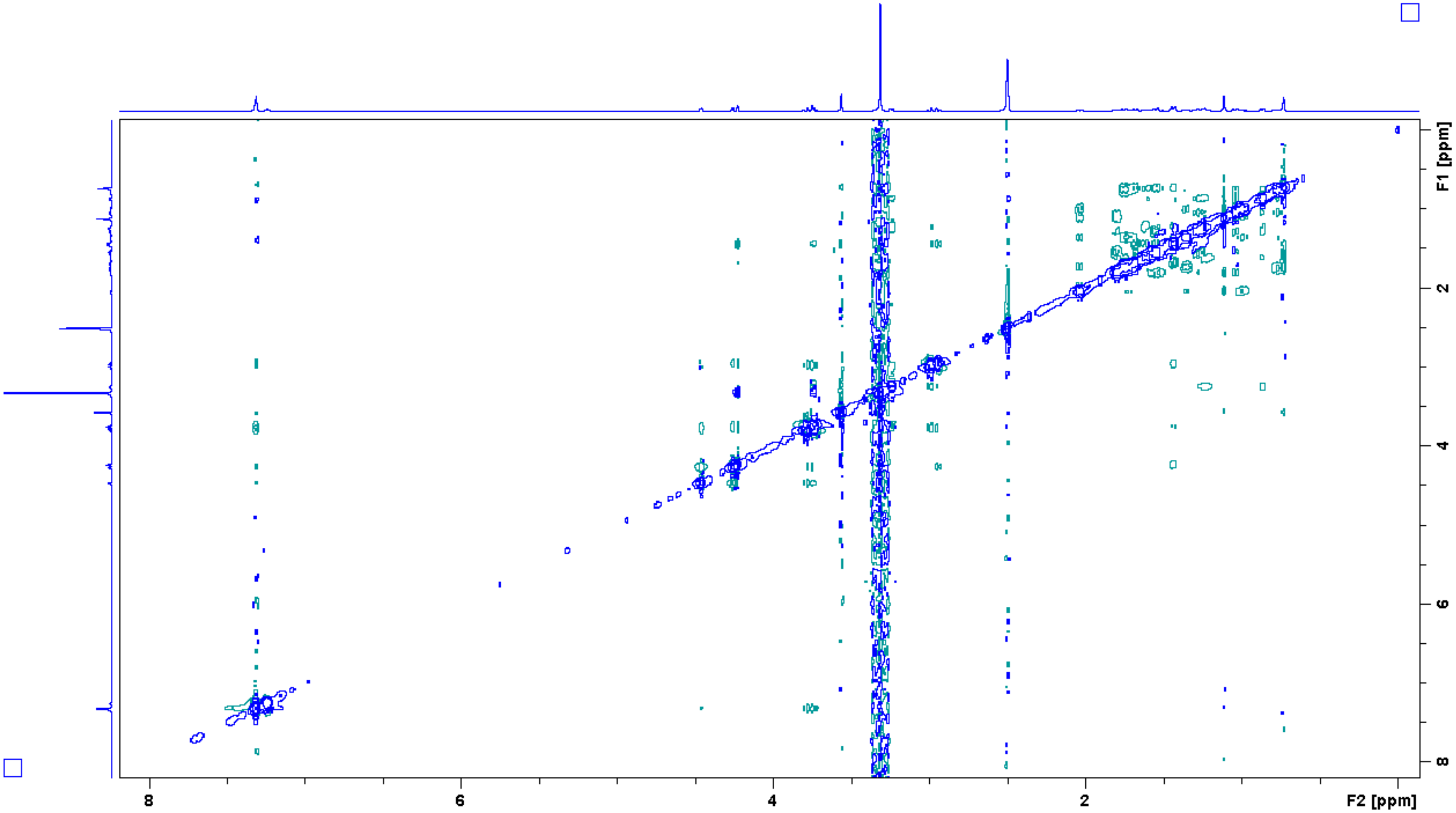

HSQC of compound (4*R*,5'*R*,6a*R*,7*R*,9*S*,11b*S*)-methyl 3'-benzyl-7,9-dihydroxy-4,11b-dimethyldodecahydro-1H-spiro[6a,9-methanocyclohepta[a]naphthalene-8,5'-oxazolidine]-4-carboxylate **(18)**

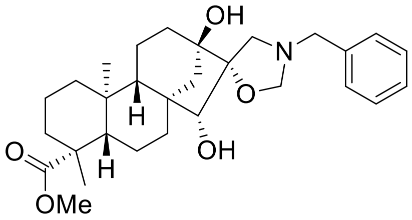

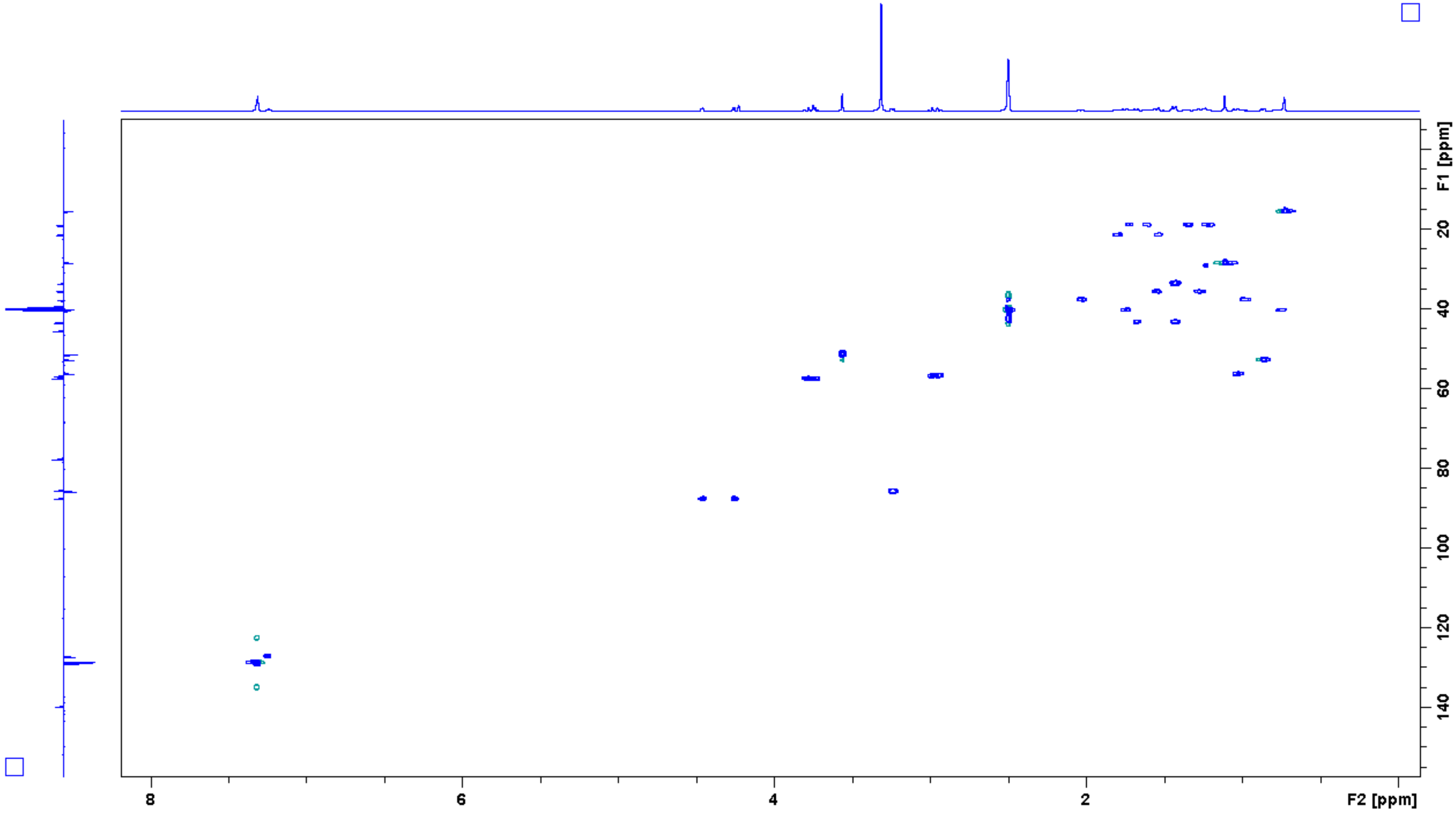

HMBC of compound (4*R*,5'*R*,6a*R*,7*R*,9*S*,11b*S*)-methyl 3'-benzyl-7,9-dihydroxy-4,11b-dimethyldodecahydro-1H-spiro[6a,9-methanocyclohepta[a]naphthalene-8,5'-oxazolidine]-4-carboxylate **(18)**

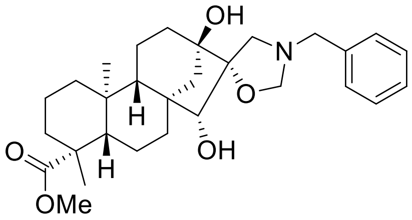

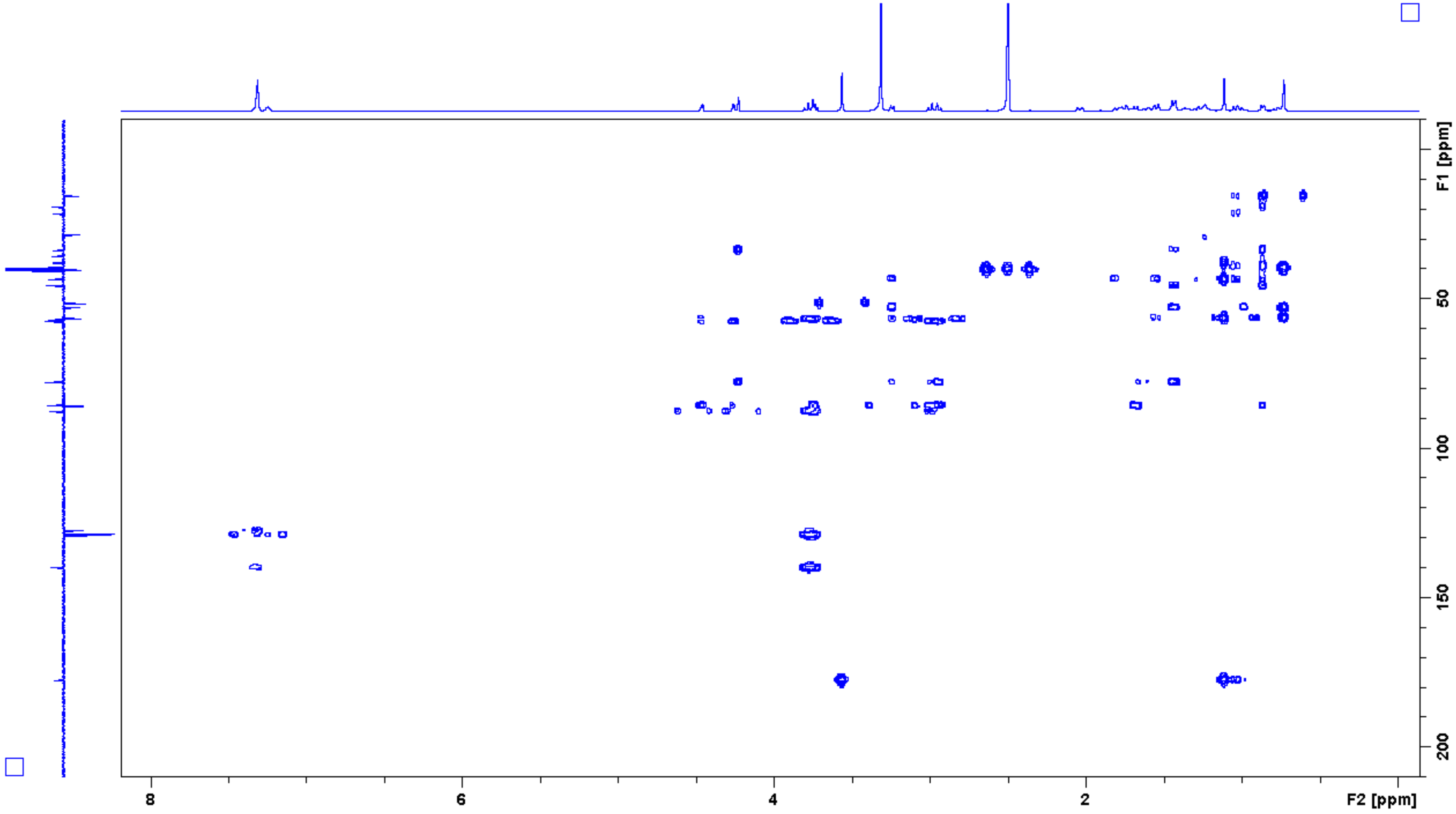

HRMS of compound (4*R*,6a*R*,7*R*,9*S*,11b*S*)-methyl 7,9-dihydroxy-4,11b-dimethyl-8-methylenetetradecahydro-6a,9-methanocyclohepta[a]naphthalene-4-carboxylate (**3**)

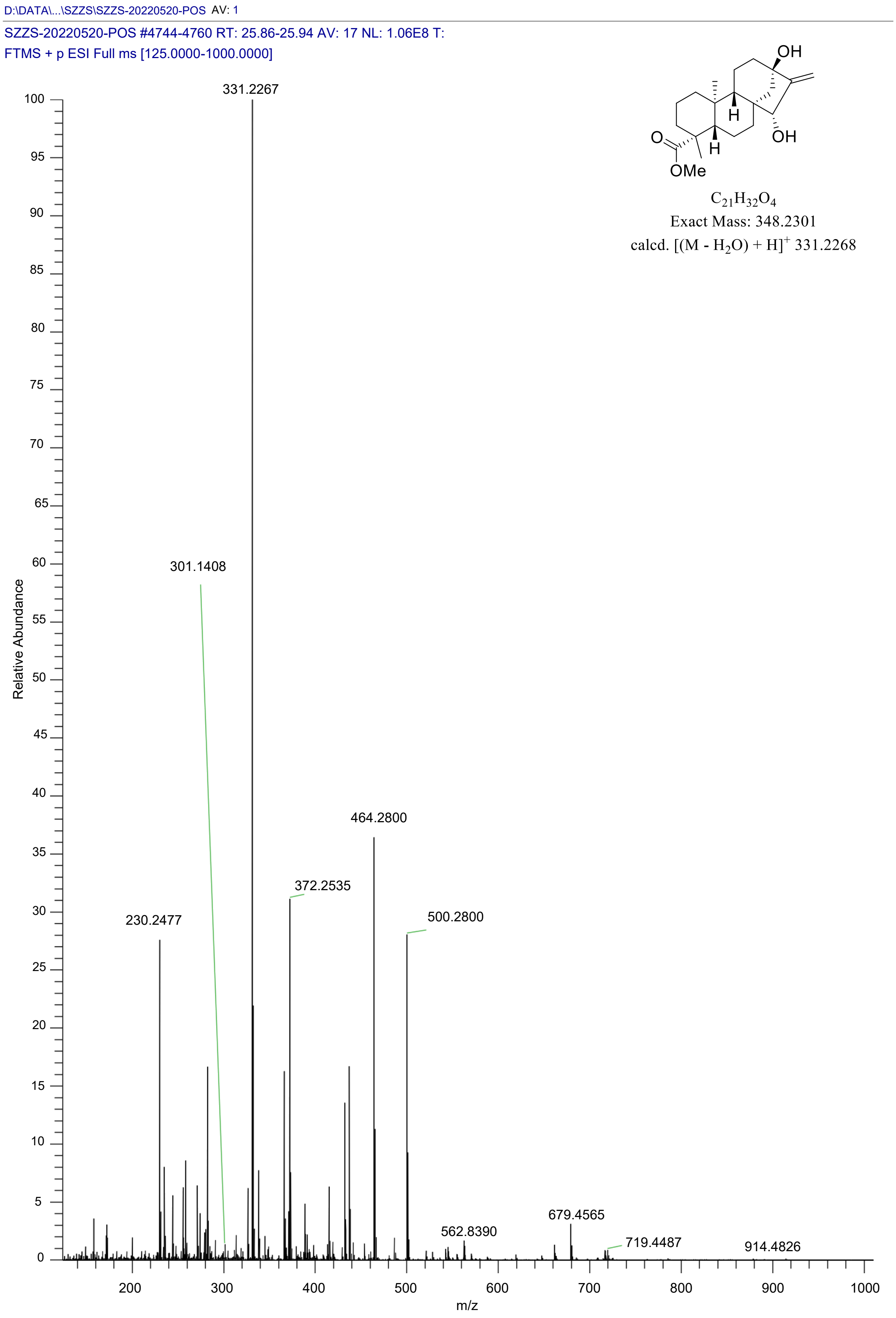

$C_{21}H_{32}O_4$
Exact Mass: 348.2301
calcd. $[(M - H_2O) + H]^+$ 331.2268

HRMS of compound (2'*R*,4*R*,6a*R*,7*R*,9*S*,11b*S*)-methyl 7,9-dihydroxy-4,11b-dimethyldodecahydro-1H-spiro[6a,9-methanocyclohepta[a]naphthalene-8,2'-oxirane]-4-carboxylate (**4b**)

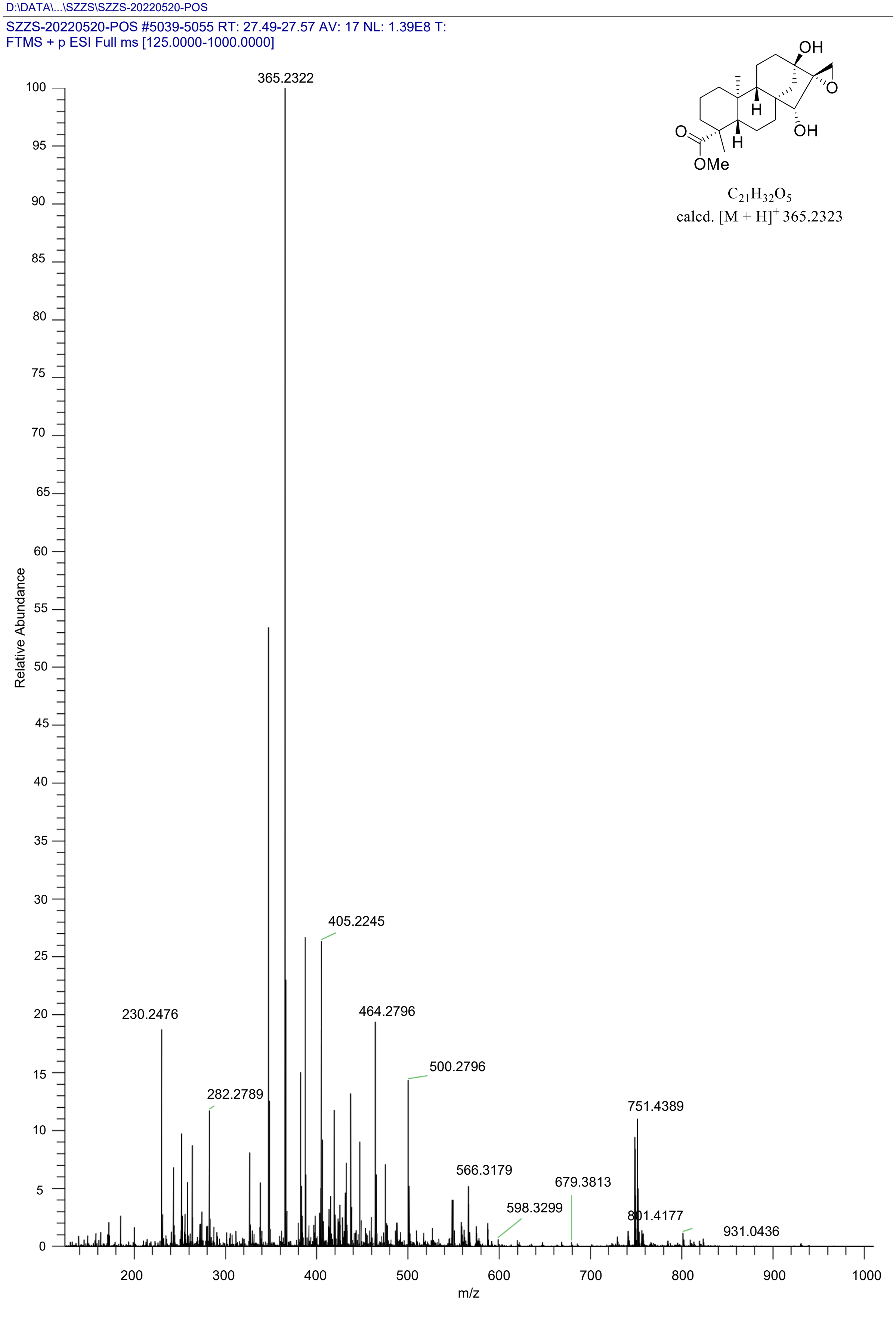

$C_{21}H_{32}O_5$

calcd. $[M + H]^+$ 365.2323

HRMS of compound (4*R*,6a*R*,7*R*,8*R*,9*S*,11b*S*)-methyl 8-((benzylamino)methyl)-7,8,9-trihydroxy-4,11b-dimethyltetradecahydro-6a,9-methanocyclohepta[a]naphthalene-4-carboxylate (**5**)

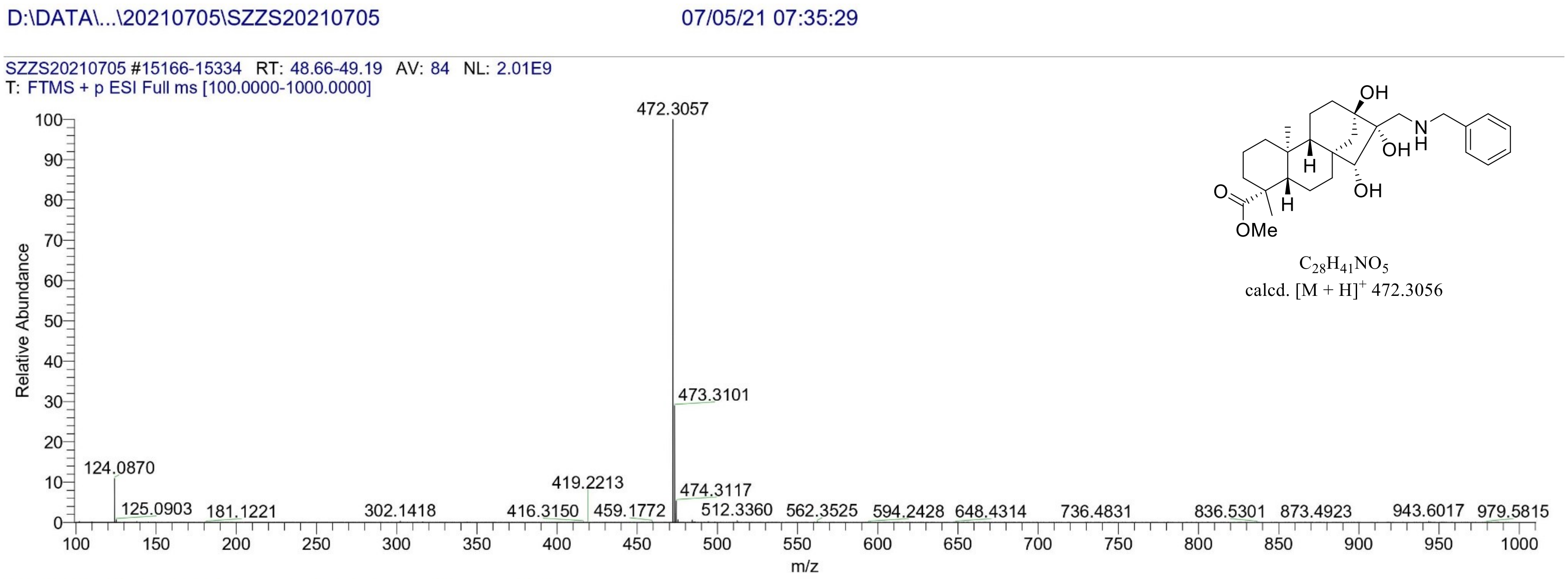

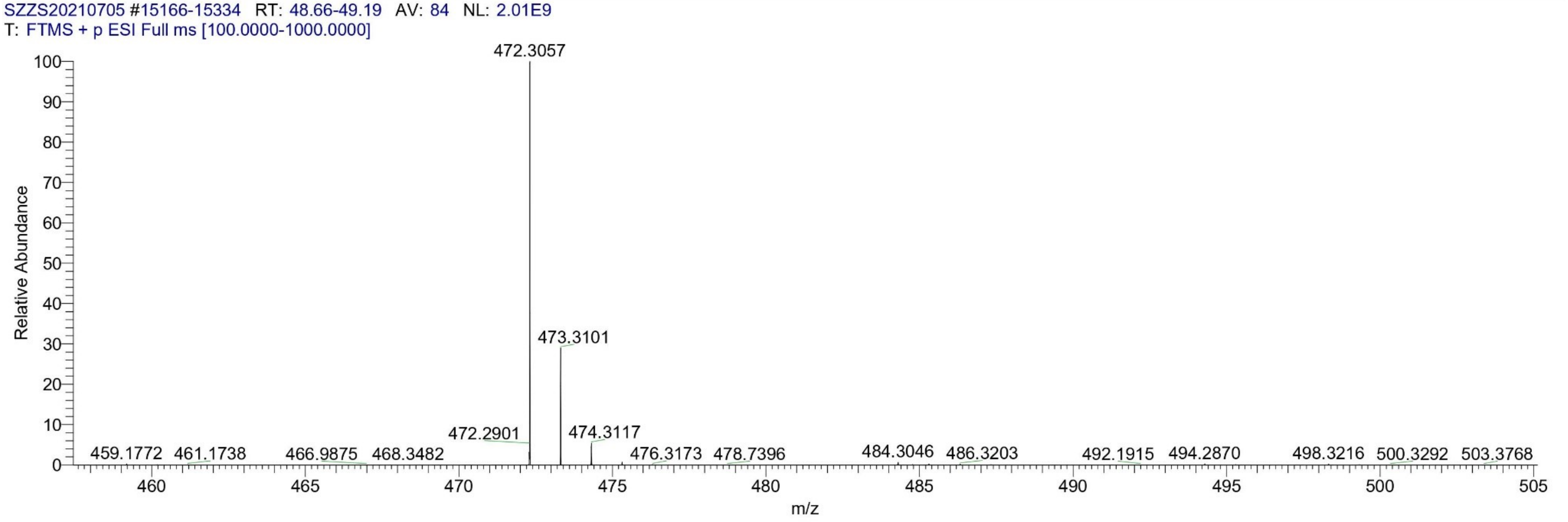

HRMS of compound (4*R*,6a*R*,7*R*,8*R*,9*S*,11b*S*)-methyl 8-((benzyl(methyl)amino)methyl)-7,8,9-trihydroxy-4,11b-dimethyltetradecahydro-6a,9-methanocyclohepta[a]naphthalene-4-carboxylate (**6**)

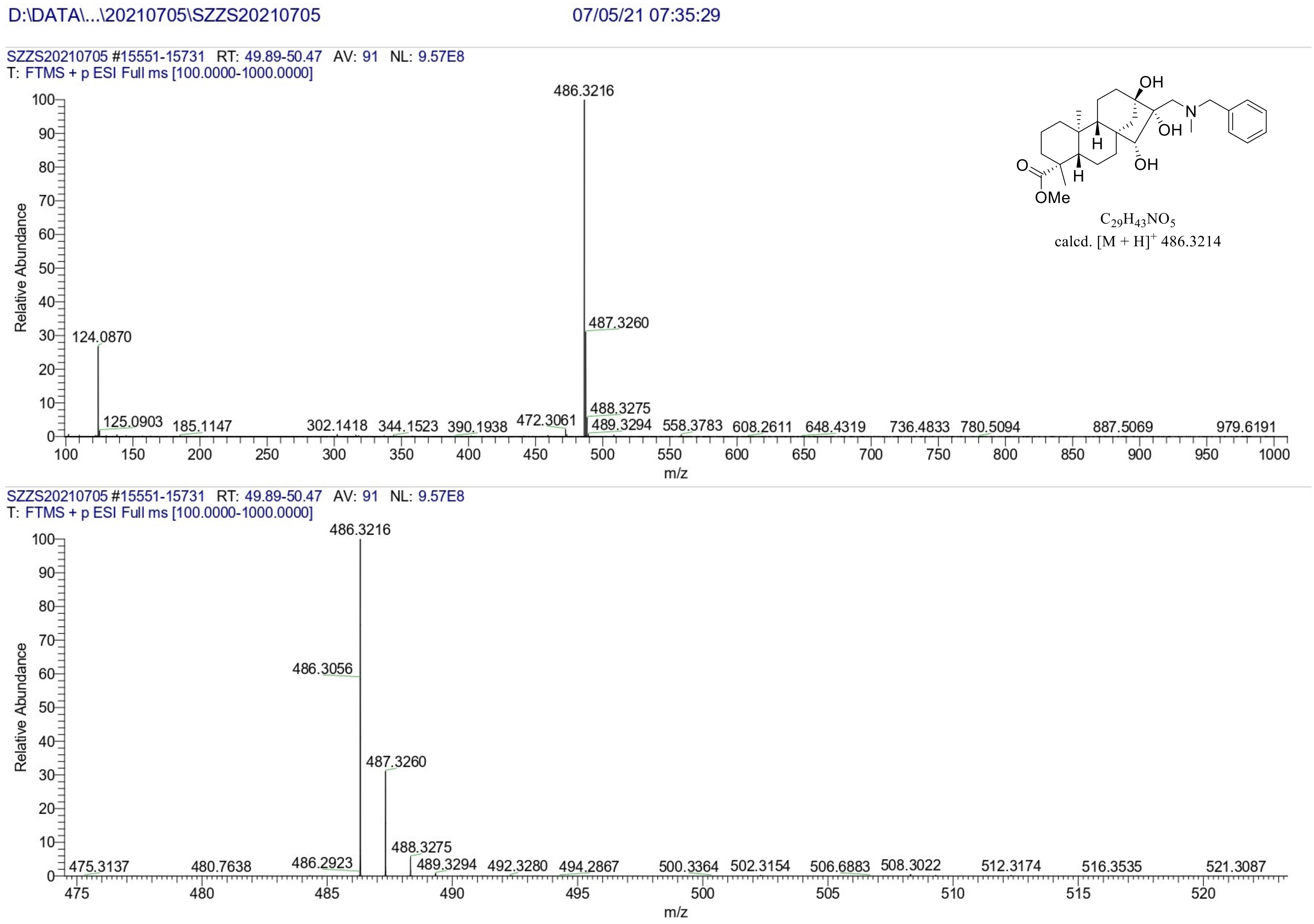

HRMS of compound (4*R*,6a*R*,7*R*,8*R*,9*S*,11b*S*)-methyl 7,8,9-trihydroxy-4,11b-dimethyl-8-((((*R*)-1-phenylethyl)amino)methyl)tetradecahydro-6a,9-methanocyclohepta[a]naphthalene-4-carboxylate (**7**)

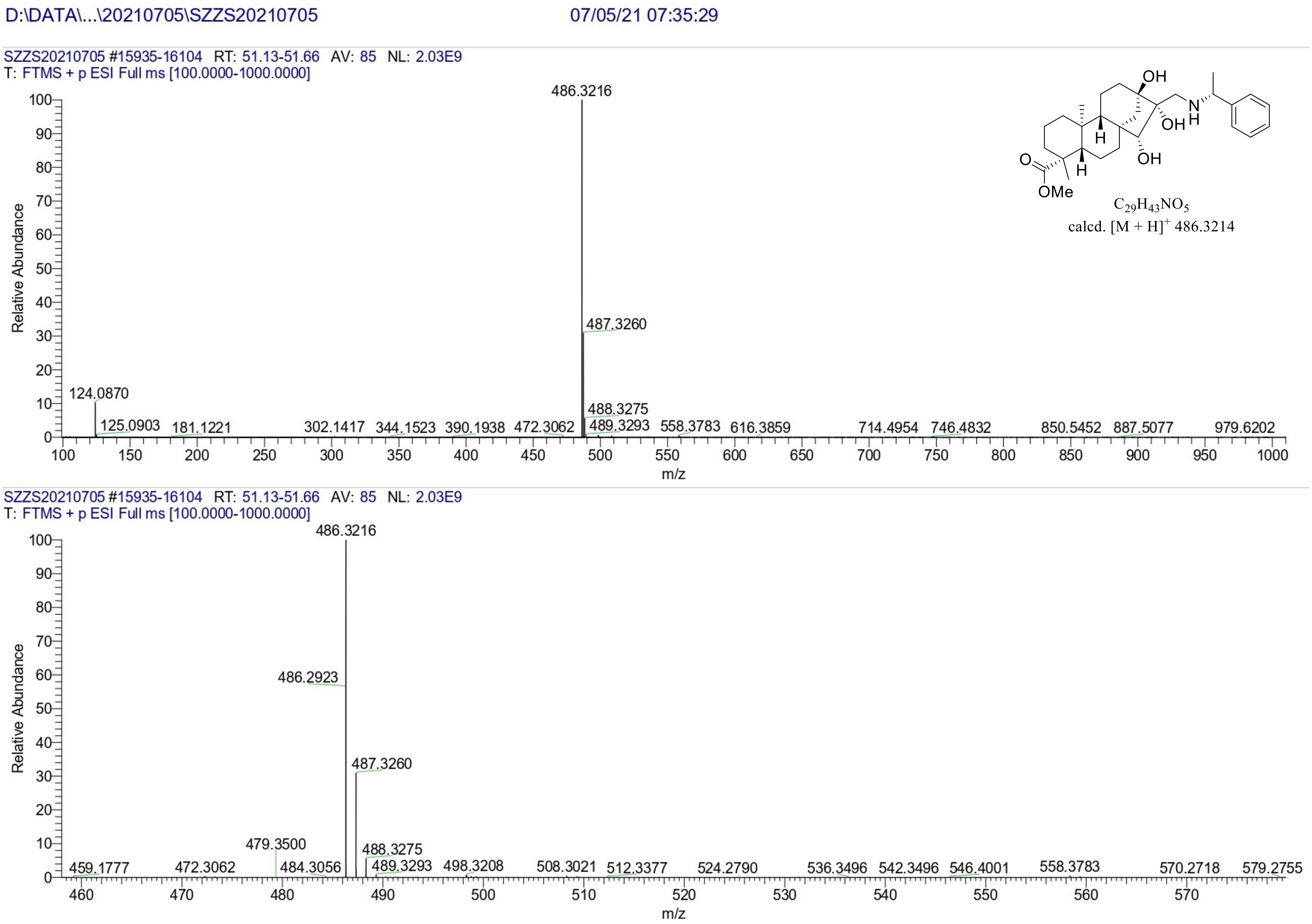

HRMS of compound (4*R*,6a*R*,7*R*,8*R*,9*S*,11b*S*)-methyl 7,8,9-trihydroxy-4,11b-dimethyl-8-((((*S*)-1-phenylethyl)amino)methyl)tetradecahydro-6a,9-methanocyclohepta[a]naphthalene-4-carboxylate (**8**)

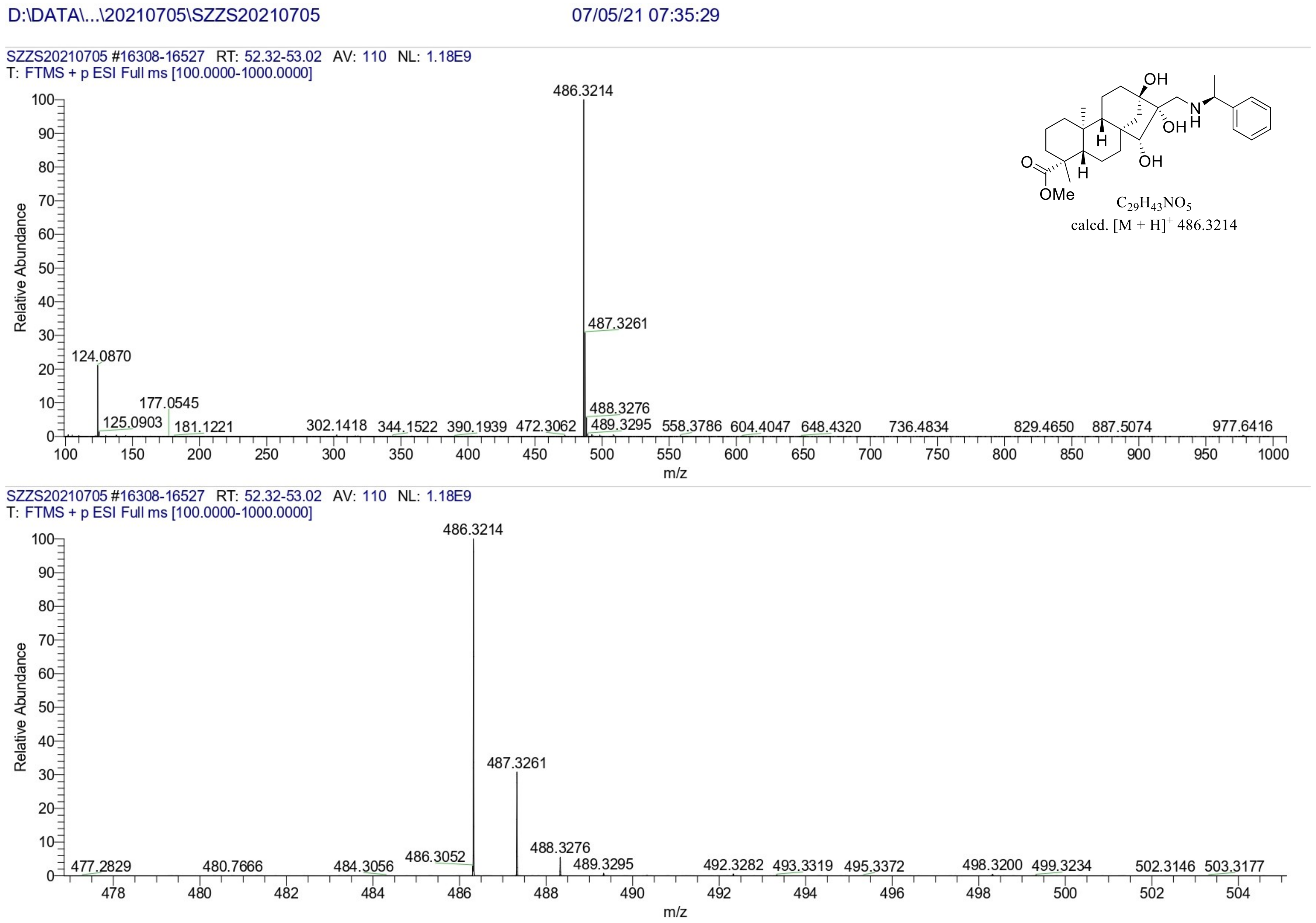

HRMS of compound (4*R*,6a*R*,7*R*,8*R*,9*S*,11b*S*)-methyl 8-(((4-fluorobenzyl)amino)methyl)-7,8,9-trihydroxy-4,11b-dimethyltetradecahydro-6a,9-methanocyclohepta[a]naphthalene-4-carboxylate (**9**)

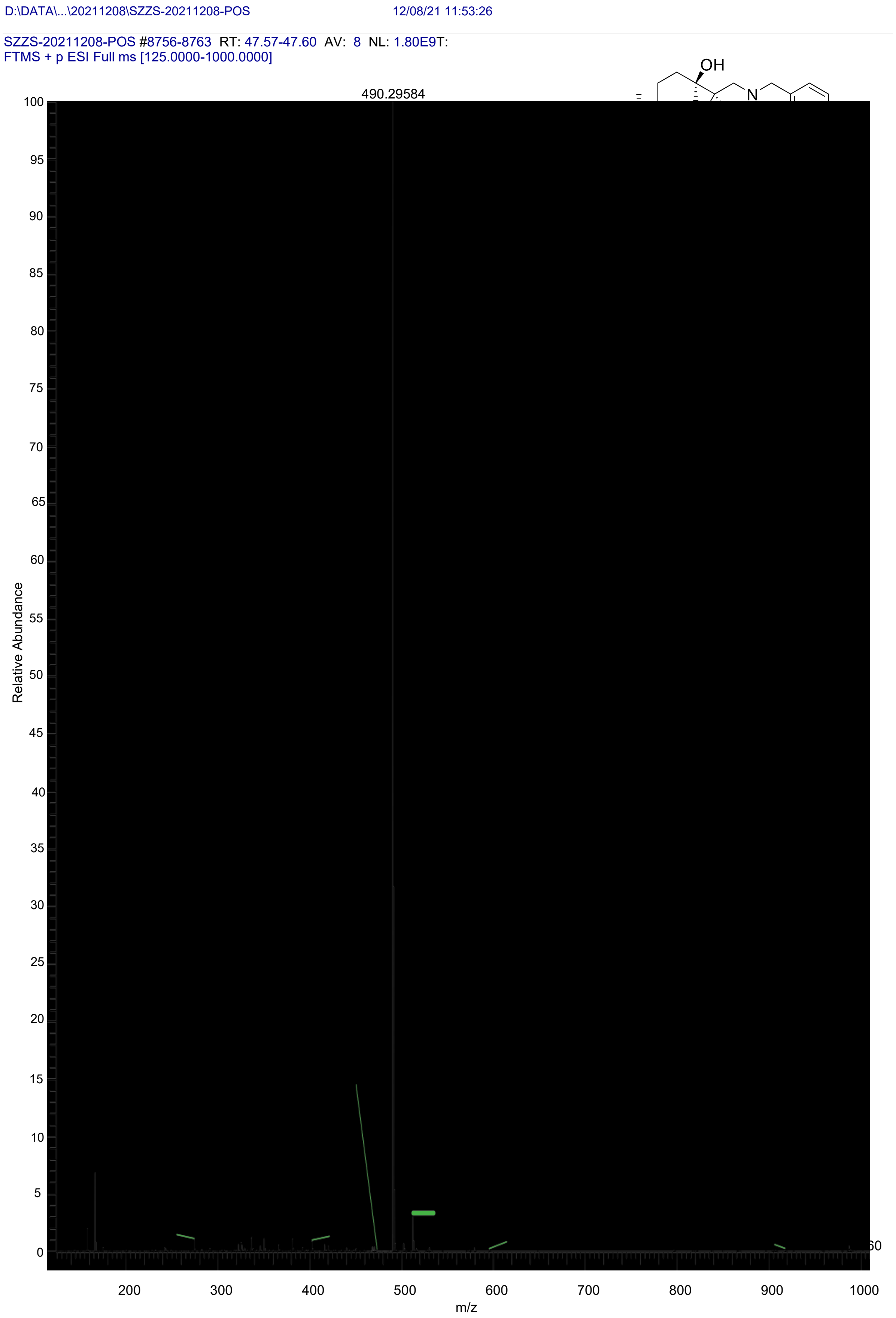

HRMS of compound (4*R*,6a*R*,7*R*,8*R*,9*S*,11b*S*)-methyl 7,8,9-trihydroxy-8-(((4-methoxybenzyl)amino)methyl)-4,11b-dimethyltetradecahydro-6a,9-methanocyclohepta[a]naphthalene-4-carboxylate (**10**)

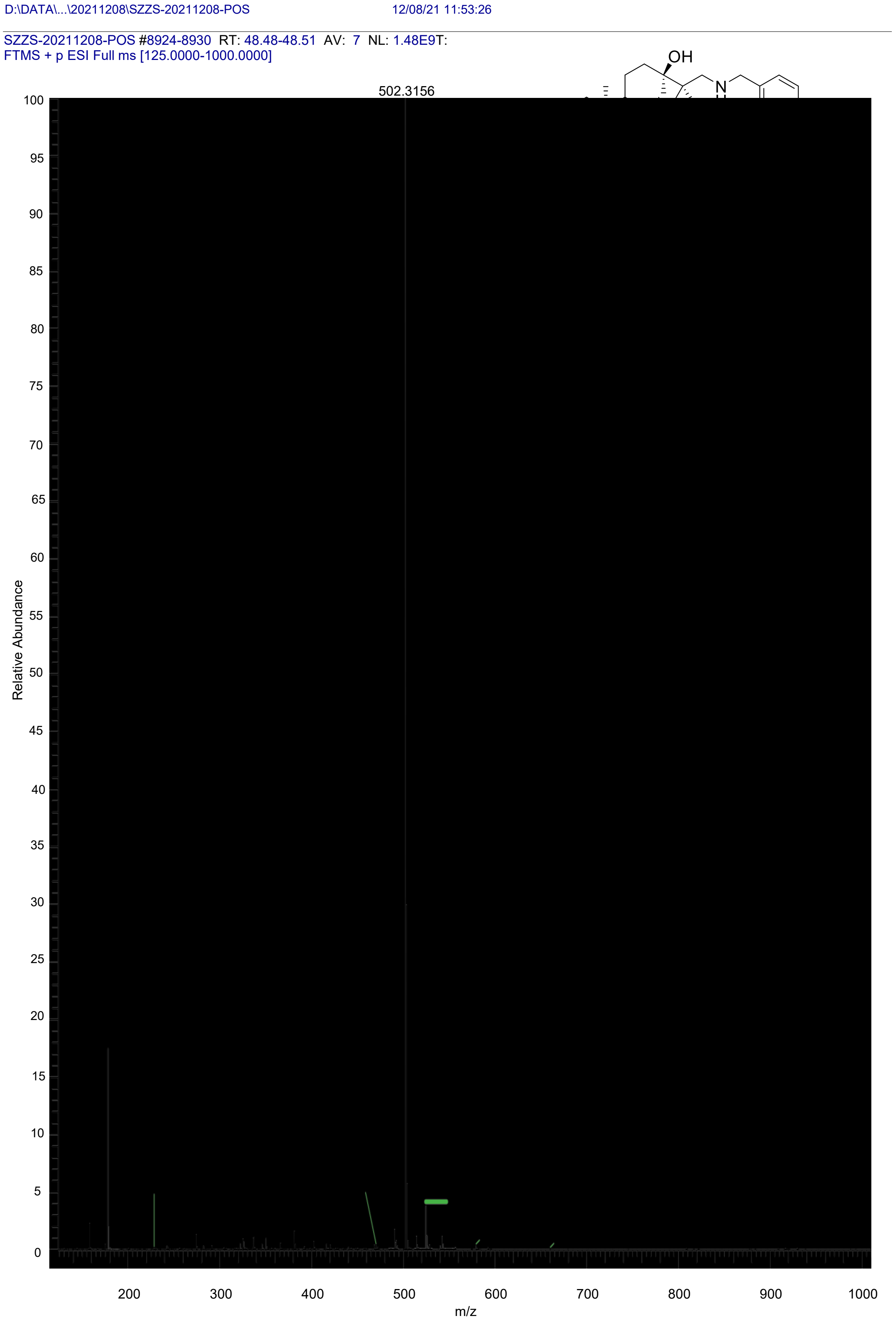

HRMS of compound (4*R*,4a*S*,6a*R*,7*R*,8*R*,11a*S*,11b*S*)-methyl 8-((((*R*)-1-(4-fluorophenyl)ethyl)amino)methyl)-7,8,9-trihydroxy-4,11b-dimethyltetradecahydro-6a,9-methanocyclohepta[a]naphthalene-4-carboxylate (**11**)

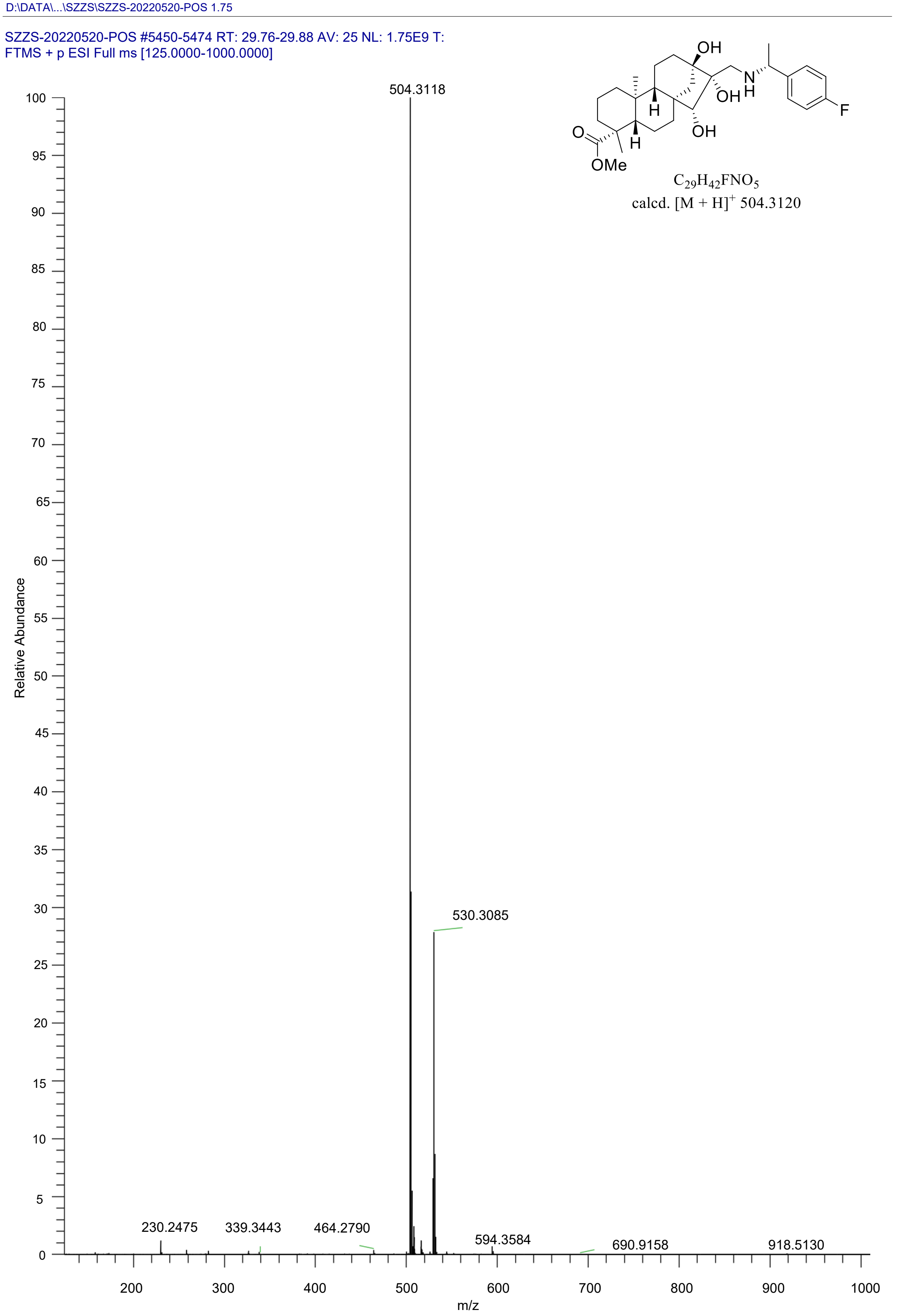

HRMS of compound (4*R*,6a*R*,7*R*,8*R*,9*S*,11b*S*)-methyl 7,8,9-trihydroxy-4,11b-dimethyl-8-((((*R*)-1-(naphthalen-2-yl)ethyl)amino)methyl)tetradecahydro-6a,9-methanocyclohepta[a]naphthalene-4-carboxylate (**12**)

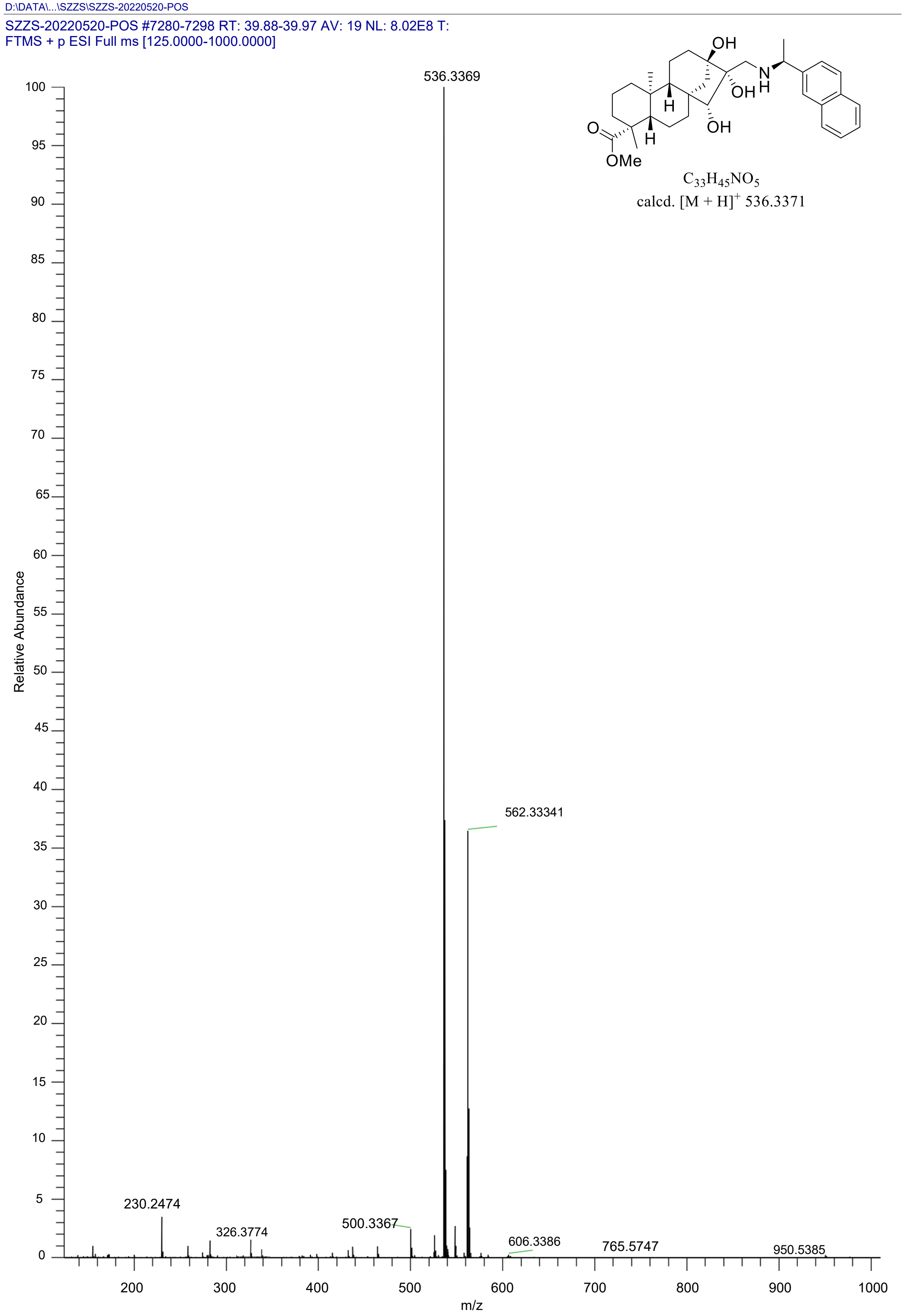

HRMS of compound (4*R*,6a*R*,7*R*,8*R*,9*S*,11b*S*)-methyl 7,8,9-trihydroxy-4,11b-dimethyl-8-((((*S*)-1-(naphthalen-2-yl)ethyl)amino)methyl)tetradecahydro-6a,9-methanocyclohepta[a]naphthalene-4-carboxylate (**13**)

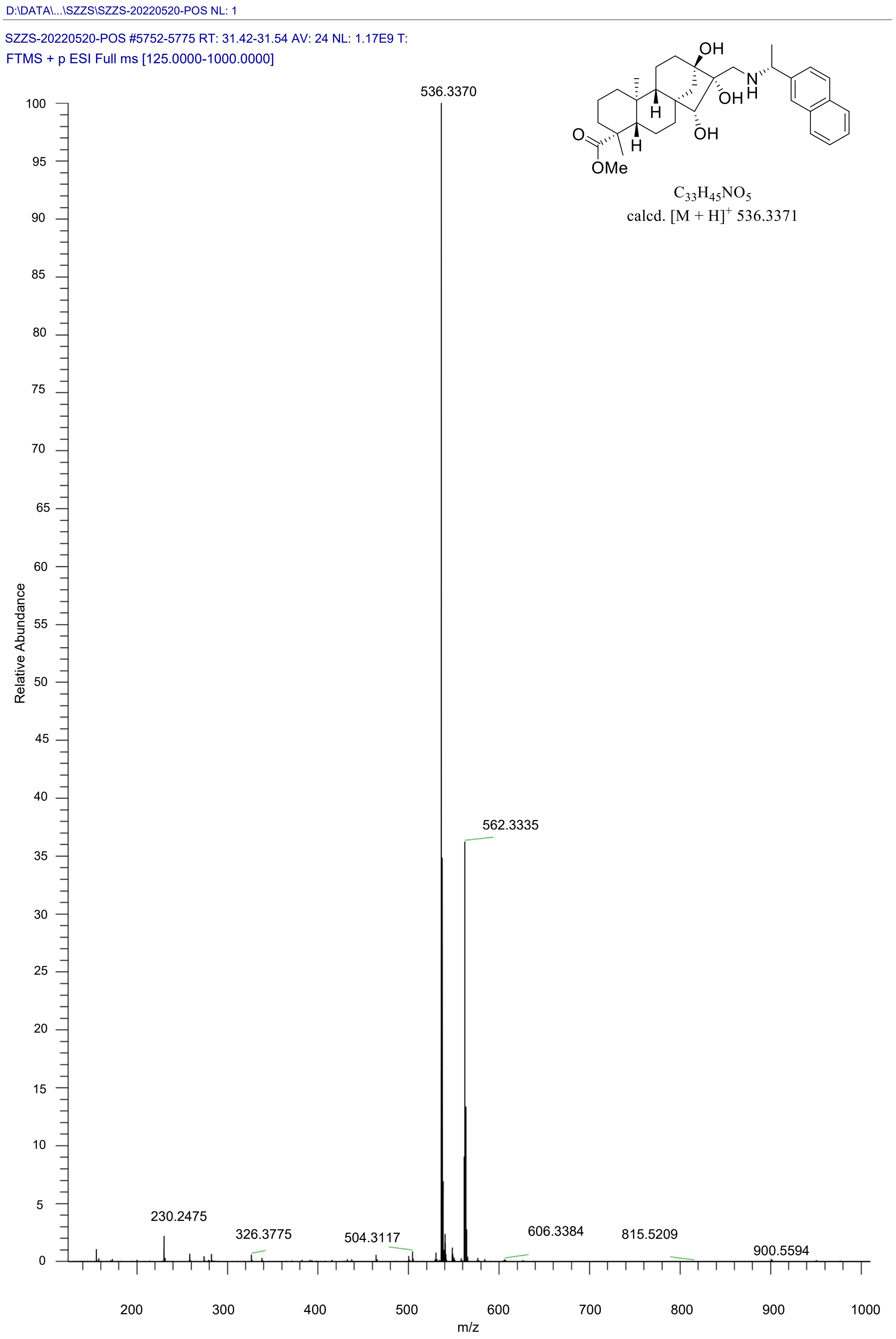

HRMS of compound (4*R*,6a*R*,7*R*,8*R*,9*S*,11b*S*)-methyl 7,8,9-trihydroxy-4,11b-dimethyl-8-(((naphthalen-1-ylmethyl)amino)methyl)tetradecahydro-6a,9-methanocyclohepta[a]naphthalene-4-carboxylate (**14**)

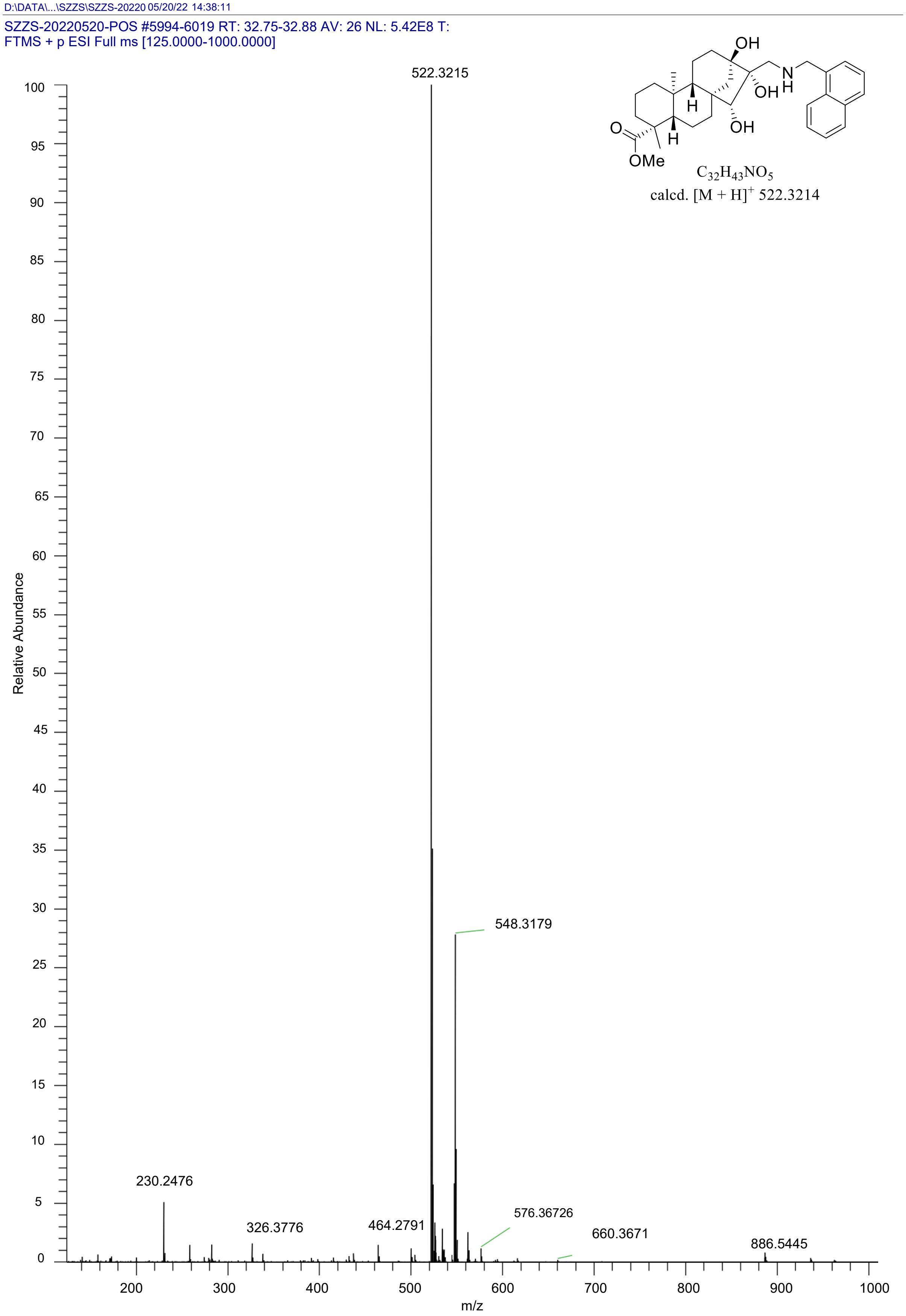

HRMS of compound (4*R*,6a*R*,7*R*,8*R*,9*S*,11b*S*)-methyl 7,8,9-trihydroxy-4,11b-dimethyl-8-((((*R*)-1-phenylpropyl)amino)methyl)tetradecahydro-6a,9-methanocyclohepta[a]naphthalene-4-carboxylate (**15**)

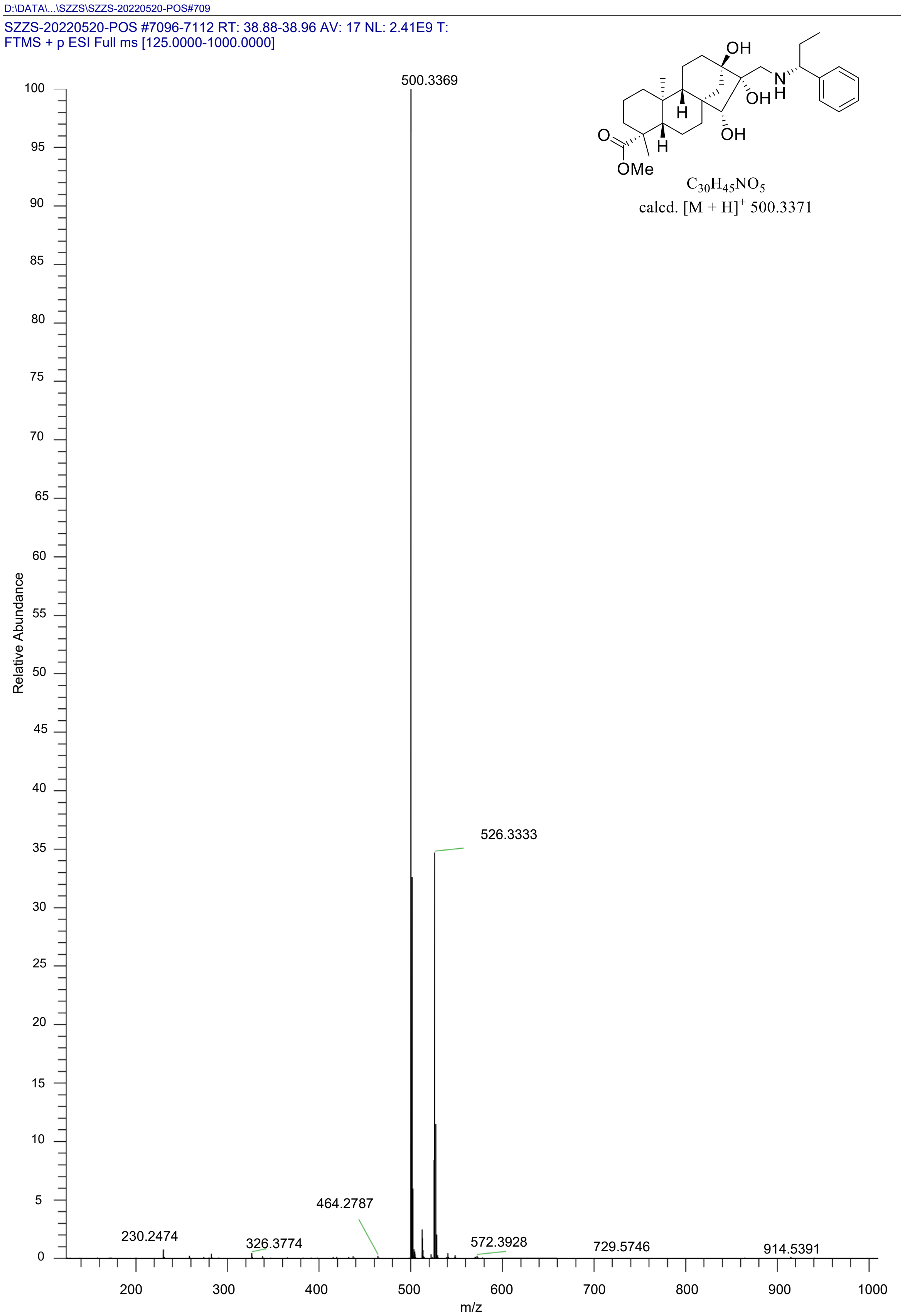

HRMS of compound (4*R*,6a*R*,7*R*,8*R*,9*S*,11b*S*)-methyl 7,8,9-trihydroxy-4,11b-dimethyl-8-((((*S*)-1-phenylpropyl)amino)methyl)tetradecahydro-6a,9-methanocyclohepta[a]naphthalene-4-carboxylate (**16**)

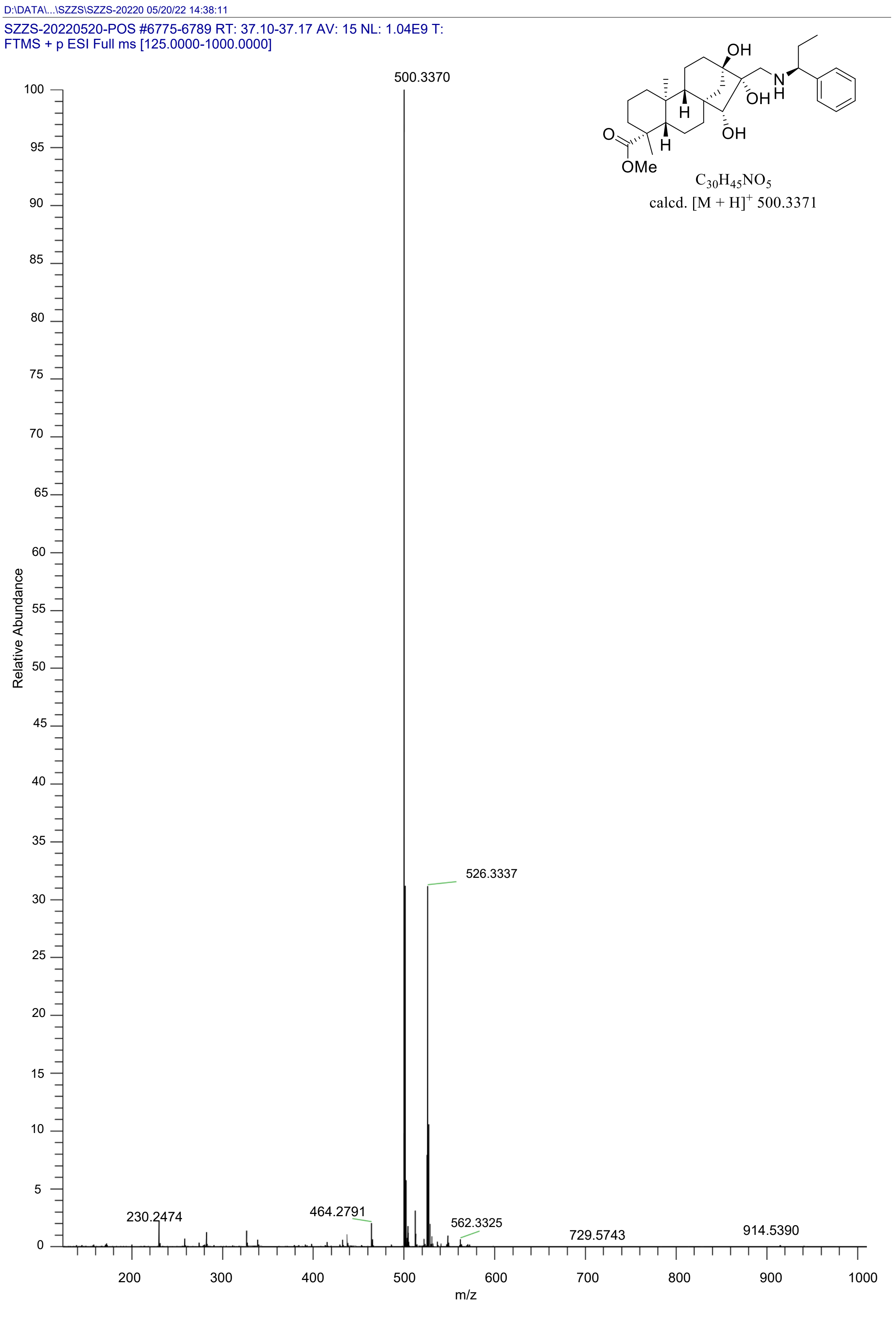

HRMS of compound (4*R*,6a*R*,7*R*,8*R*,9*S*,11b*S*)-methyl 8-(aminomethyl)-7,8,9-trihydroxy-4,11b-dimethyltetradecahydro-6a,9-methanocyclohepta[a]naphthalene-4-carboxylate (**17**)

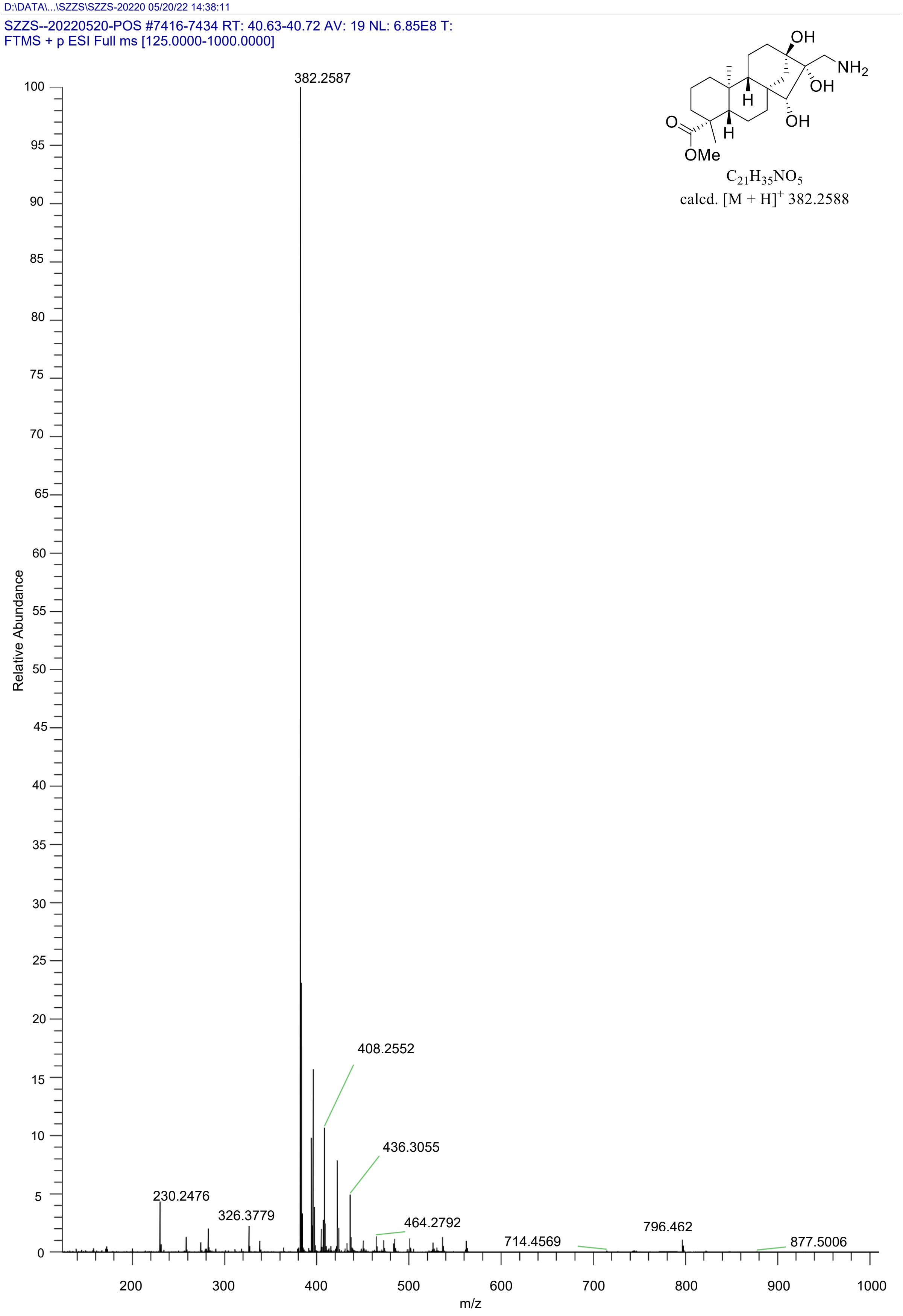

$C_{21}H_{35}NO_5$
calcd. $[M + H]^+$ 382.2588

HRMS of compound (4*R*,5'*R*,6a*R*,7*R*,9*S*,11b*S*)-methyl 3'-benzyl-7,9-dihydroxy-4,11b-dimethyldodecahydro-1H-spiro[6a,9-methanocyclohepta[a]naphthalene-8,5'-oxazolidine]-4-carboxylate (**18**)

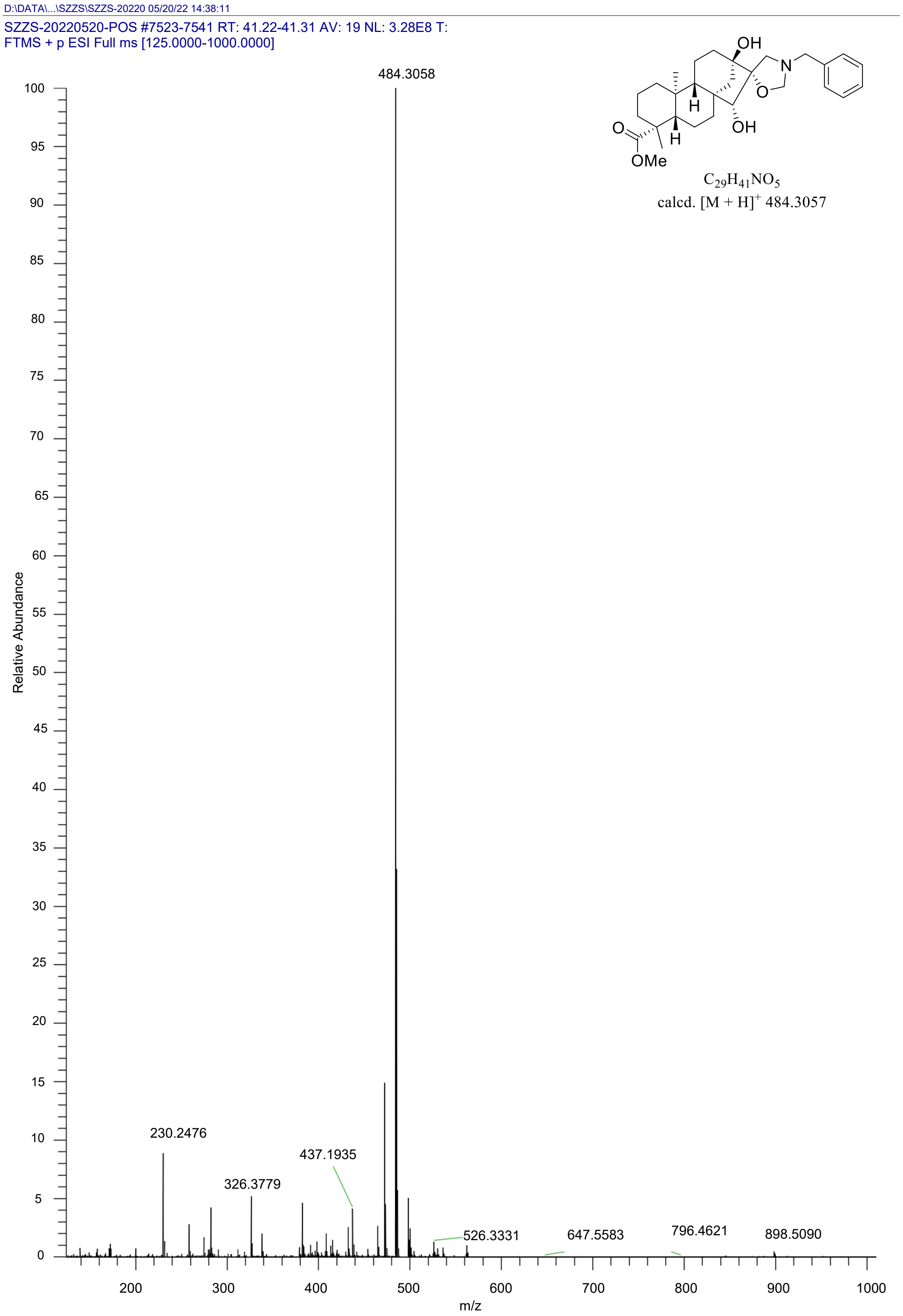

Supplement: Supplementary file 1 [file ijms-24-01121-s001.zip › ijms-2134986-supplementary.pdf]
